# Targeting Highly Reactive Oxygen Species (hROS) for Prodrug Activation through a Cascade Reaction with Kinetic Tunability (CReKT) to Effect Linker Cleavage

**DOI:** 10.1021/acs.jmedchem.5c03627

**Published:** 2026-03-16

**Authors:** Shameer M. Kondengadan, Shubham Bansal, Wen Lu, Manjusha Roy Choudhury, Binghe Wang

**Affiliations:** Department of Chemistry and Center for Diagnostics and Therapeutics, 1373 Georgia State University, Atlanta, Georgia 30301, United States

## Abstract

Most ROS-sensitive cleavable linkers rely on their broad reactivity toward all ROS. However, individual ROS have very specialized functions in various pathologies. For example, HOCl/OCl^–^ is primarily produced in response to infection and/or inflammation by certain immune cells that express myeloperoxidase (MPO). We herein describe a novel HOCl/OCl^–^-selective prodrug approach through an oxidation-initiated Cascade Reaction with Kinetic Tunability (CReKT) for drug release. Specifically, HOCl/OCl^–^ oxidation of a phenylthioether is used to trigger prodrug activation via enhancing the nucleophilicity of the S-connected carbon for condensation-based payload release. The reactivity of the S-connected carbon is further augmented by tethering to an electron-withdrawing group (EWG) and by creating synergy with proximity effects. Tunability of release kinetics can be achieved by varying the EWG, substitution on the phenyl ring, and entropic factors. This approach offers new tools and sets a new direction in designing species-selective ROS-sensitive prodrugs.

## Graphical abstract

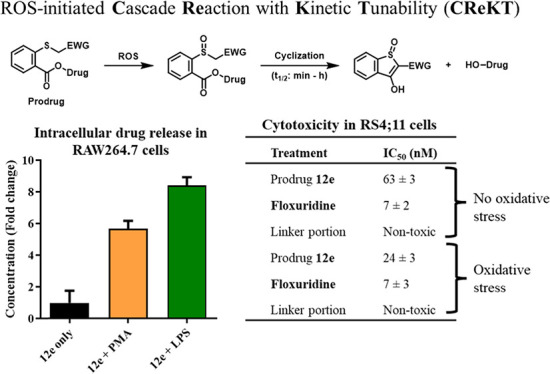

## 1 Introduction

It is well-known that there is a strong association between the overproduction of reactive oxygen species (ROS) and a large number of pathologies.^1–6^ It should be noted that ROS represents a collection of species with different reactivities and functions including hydrogen peroxide (H_2_O_2,_ the most abundant and stable), hypochlorous acid/hypochlorite (HOCl/OCl^–^, the second most abundant and stable), superoxide anion (O_2_^–.^, short-lived), hydroxyl radicals (OH·, react with almost everything in the cell), peroxynitrite (ONOO^–^, short-lived), and singlet oxygen species (^1^O_2_, short-lived), among other forms.^7,8^ These ROS are formed as products of various redox reactions in different cellular compartments such as mitochondria, cytoplasm, endoplasmic reticulum, and peroxisomes.^9,10^ Naturally, there have been intense interests in developing ROS-sensitive linker chemistry for targeted delivery of therapeutic and imaging agents.^3,10–12^ Along this line, the boronate and selenide groups are the most widely used ROS-sensitive triggers (Figure 1).^13–17^ Boronate-based approaches take advantage of its highly reactive nature toward ROS for the generation of a highly unstable intermediate, which spontaneously collapses to release the drug payload.^18^ For example, one most widely used method is the oxidation of an aryl boronate to a phenolic hydroxyl group for drug payload release through a 1,6-elimination reaction, leading to the formation of a quinone methide moiety (Figure 1A-i).^14,15,19^ Additionally, boronate oxidation has been used to release payload through β-elimination triggered by the oxidation of the boryl allyloxy (BAO) ether moiety (Figure 1A-ii).^16,20^ The use of a selenide moiety also takes advantage of its highly reactive nature toward ROS for the formation of selenium oxide, which can easily undergo an elimination reaction for prodrug activation (Figure 1A-iii).^17^ Both functional groups can be activated by all ROS including the most abundant and least reactive hydrogen peroxide.^18,21–24^ For example, the calculated first *t*_1/2_ of hydrogen peroxide reaction with boronate and selenide is 1.3 h and 35 min at 100 μM each, respectively.^18,24^ Even a stabilized version of a boron-based ROS-sensitive group, the diazaborine group, has a second-order rate constant of about 0.422 M^–1^ s^–1^ for H_2_O_2_, giving a first calculated *t*_1/2_ of 6.6 h at 100 μM each.^25^ Excellent progress has been made using such pan-ROS-sensitive activation chemistry. Furthermore, such progress has also led to the recognition that there is a need for species-selective linker chemistry as well. Furthermore, boronates are strong electrophiles, which present other problems.^26^ Finding ROS-sensitive chemistry without the use of a strong electrophile is expected to be an advantage.

**Figure 1:**
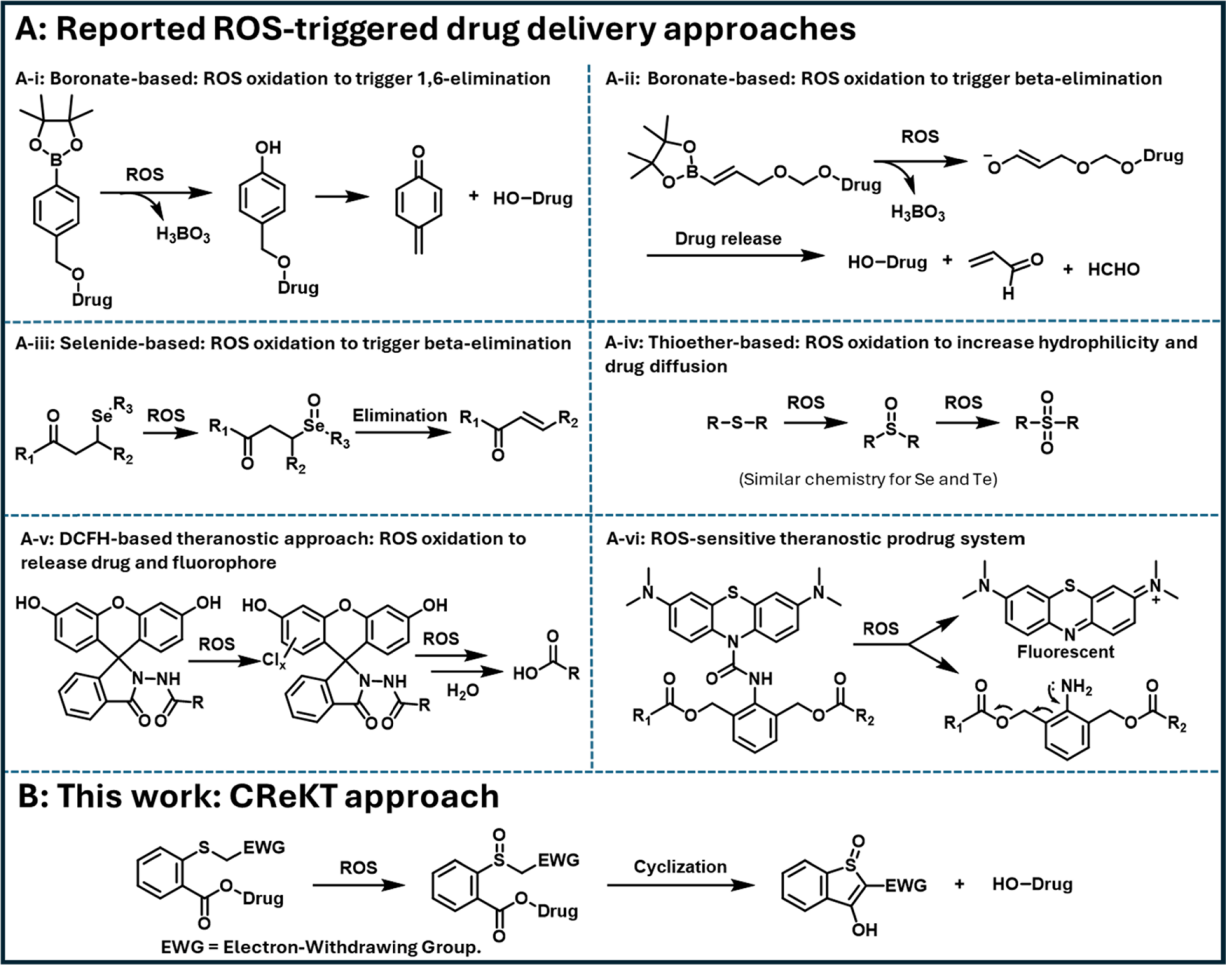
ROS-triggered drug delivery approaches. (A) Prodrug activation chemistry of reported ROS-triggered drug delivery approaches. (B) Prodrug activation chemistry of our CReKT approach.

At this point, it needs to be noted that among all the ROS, H_2_O_2_ and OCl^–^ are the only two that are stable enough to accumulate to some level of abundance (from low nanomolars to high micromolars) for various general drug delivery applications.^27^ Many ROS are too reactive and too short-lived to be at a high enough concentration or selectivity for prodrug activation applications.^28^ This analysis is based on the fact that most drugs function at mid-nanomolar–high-micromolar concentrations with some exceptions such as digitalis.^29–31^ Therefore, ROS that do not provide this level of accumulation or production would not be able to activate a sufficient amount/concentration of the intended prodrugs for therapeutic applications. For example, most one-electron oxidants are short-lived (commonly *t*_1/2_: 10^–6^–10^–9^ s) including OH^•^, O_2_^–•^, alkoxyl radical (RO^•^), nitrogen dioxide (NO_2_^•^), and alkyl peroxyl (ROO^•^).^32,33^ Among them, OH^•^ reacts with almost everything instantaneously upon formation, affording no meaningful selectivity.^34^ O_2_^–•^ dismutates with an ultrafast rate constant of 5 × 10^5^ and 5 × 10^9^ M^–1^ s^–1^ in the absence and presence of superoxide dismutase (SOD), respectively.^35^ This means that any other reactions of O_2_^**.**^^–^ must outperform the kinetics of SOD or spontaneous dismutation itself to be kinetically relevant. The formation of peroxynitrite (ONOO^–^) from NO· and O_2_^**.**^^–^ is the only reaction that could match this kinetic requirement (*k* = 1.9 × 10^10^ M^–1^ s^–1^).^36^ Even the two-electron peroxynitrite (ONOO^–^) only has a lifetime of 1 s in pure aqueous solution at pH 7.4 and 10–20 ms in the biological system.^37^ All these analyses lead to the point that only H_2_O_2_ and hypochlorous acid/hypochlorite (HOCl/OCl^–^) accumulate to a high enough concentration for prodrug activation purposes.^8^

In this study, we are interested in developing ROS-sensitive prodrug linker chemistry that is kinetically stable in the presence of the least reactive ROS, hydrogen peroxide, and can be quickly activated by highly reactive oxygen species (hROS) such as HOCl/OCl^–^ for several reasons. First, there have been many H_2_O_2_-sensitive linkers reported that serve the purpose of research tools very well. Second, H_2_O_2_ is ubiquitous and can exist in micromolar concentrations under frequently encountered pathophysiological conditions. For example, most human urine samples were found to contain 3.5–100 μM of H_2_O_2_ in several studies_._ ^27,38–40^ Similarly, fresh breast milk was found to contain >20 μM of H_2_O_2_.^41,42^ In blood plasma, the concentration of H_2_O_2_ is normally in the low micromolar range but can reach 50 μM during systemic inflammation.^43^ These concentrations are consistent with the essential physiological roles of H_2_O_2_ and yet are high enough to activate the most commonly used boronate-based ROS-sensitive prodrugs (rate constant: ∼2 M^–1^ s^–1^) to a therapeutically relevant level (nanomolar to micromolar).^27,44^ Third, the cytosolic concentration of H_2_O_2_ is known to be lower than the extracellular concentration by hundreds of fold^45^ and to be in the nanomolar range.^45–48^ The concentration difference between the extracellular matrix and mitochondrial matrix has been reported to be even greater at over 1500-fold.^49^ Such concentration differences negatively affect intracellular targeting if the focus is on H_2_O_2_. Fourth, our body is frequently exposed to external H_2_O_2_ at fairly high levels, which needs to be considered in analyzing selective prodrug activation.^40,41^ For example, many commonly consumed beverages including tea and instant coffee have been reported to contain more than 100 μM of H_2_O_2._ ^40^ Fifth, many “antioxidants” including epigallocatechin gallate, ascorbate, and gallic acid lead to H_2_O_2_ production, sometimes in stoichiometric amounts.^50,51^ In animal model studies, *i.v.* administration of vitamin C at typical human pharmacologic doses led to formation of 20 μM of H_2_O_2_.^51^ Such results foretell challenges in targeting diseases using a H_2_O_2_-triggering mechanism. Sixth and very importantly, HOCl/OCl^–^ is the second most abundant ROS^27^ and primarily produced in response to pathological conditions such as infection and/or inflammation by immune cells of myeloblast origin.^52–54^ Such cells include neutrophils and monocytes that express myeloperoxidase (MPO), leading to the formation of OCl^–^ from H_2_O_2_.^52–56^ Neutrophil and macrophage infiltrate into solid tumor and inflamed sites,^52–56^ making them ideal targets for hROS-sensitive drug delivery. It should be noted that hypochlorous acid has a p*K*_a_ of ∼7.5. Therefore, almost 50% of hypochlorous acid is in the pronated (HOCl) form and the rest in the deprotonated (OCl^–^) form at around cytosolic pH. We have used OCl^–^ as a representative form in our description.

There have been a large number of publications on hROS-sensitive imaging work including the use of DCFH for sensing hypochlorite (Figure 1A-v).^57^ There have also been efforts in employing similar chemistry for developing theragnostic agents with much success (Figure 1A-v,vi).^58–60^ However, the development of hROS-sensitive drug delivery chemistry still lags far behind with a strong need for new chemistry. In this study, we take a fundamentally different approach in achieving hROS sensitivity by bringing synergy among several factors to afford prodrug stability and kinetic tunability. Herein, we discuss our approach for selective activation through an OCl^–^-initiated Cascade Reaction with Kinetic Tunability (CReKT, for easy communication) for drug release and our experimental findings (Figure 1B).

## 2 Results and Discussion

### 2.1 The Design

In designing a new hROS-sensitive prodrug approach, we strive to avoid relying on the use of a highly reactive functional group to generate an intermediate that would spontaneously collapse to achieve prodrug activation (e.g., Figure 1A-i). Instead, we desire to use ROS oxidation as a way to leverage the reactivity of a tethered neighboring group to the point that the subsequent bond-breaking reaction is feasible under ambient conditions, especially when the subsequent reaction is facilitated by entropic factors. In doing so, we turn to what is normally considered a noncleavable hROS-sensitive group, a thioether, which is chemically stable.^13^ Specifically, OCl^–^ oxidation of phenylthioether is used to create a cleavable event via enhancing the nucleophilicity of the S-connected carbon for condensation-based payload release (Figure 1B). The reactivity of the S-connected carbon is further augmented by being tethered to an electron-withdrawing group (EWG) and creating synergy with proximity effects. Tunability of release kinetics can be achieved by varying the EWG, substitution on the phenyl ring, and entropic factors. Using this approach, we aim for the following features: (1) stable prodrugs, (2) selectivity for hypochlorite activation, (3) tunability in release kinetics, and (4) free of labile metal- or boron-based groups. This approach of generating synergy from various factors sets a new direction in designing species-selective ROS-sensitive prodrugs and allows us to convert a “noncleavable”^13^ ROS-sensitive thioether group to a “cleavable” one. As such, the resulting prodrugs are highly stable and allow for independently tunable ROS sensitivity and drug release kinetics.

We should note that, first, thioether oxidation to sulfoxide has been widely used in ROS-sensitive polymer-based delivery of untethered drugs,^13,61–64^ demonstrating a key feasibility component (Figure 1A-iv). Second, both thioether and sulfoxide are chemically stable and widely used functional groups in drug design, demonstrating compatibility in pharmaceutical applications.^65^ Third, the introduction of an electron-withdrawing sulfoxide group is known to decrease the p*K*_a_ of a methylene group by at least 3 units relative to thioether and to decrease the p*K*_a_ of the α-proton of a ketone carbonyl group by as much as 10 units,^66^ allowing for manipulation of the nucleophilicity of the α-carbon for prodrug activation.^67,68^ This point is discussed in a later section. Fourth, introducing entropic factors is known to significantly facilitate nucleophilic condensation or substitution reactions to allow for feasibility under near-physiological conditions.^69,70^ For all of these reasons, we have designed a novel linker chemistry relying on this ROS-initiated CReKT chemistry (Figure 1B).

The design principle requires an appropriately positioned ester group in a way that a latent nucleophile (e.g., the α-carbon of an EWG) is in an entropically favorable position for condensation-based ester cleavage and thus release of the payload (Figure 1B). Further, the same α-carbon is attached to a ROS-sensitive thioether group. The design is such that there should be a significant and meaningful difference in the reactivity of the latent nucleophile before and after ROS oxidation of the thioether group to sulfoxide, allowing oxidation-initiated cyclization. Various options of EWGs can be used to tune electronic and steric factors for controlling the reaction kinetics and for optimizing the physicochemical properties. A proper design requires an analysis of the p*K*_a_ of the α-proton under different scenarios. For selecting the EWGs, we started with an amide, ester, ketone, trifluoromethyl, and cyano group as their Hammet constant^71^ is in the range of 0.32–0.66 and the p*K*_a_ of their respective α-proton is about 26,^72^ 21,^66^ 19,^66^ and 21 ^73^ for an amide, ester, ketone, and cyano group, respectively. Literature precedents have shown that tethering a second EWG such as a ketone or a sulfoxide in the α-position could lower the p*K*_a_ by as much as 15 units.^66,74^ We reasoned that such a wide latitude in tuning the p*K*_a_ of the α-proton should allow us to optimize the system for the appropriate nucleophilicity of the α-position for condensation under near-physiological conditions. Therefore, we designed a system (Figure 2) that affords an entropically favorable five-membered ring after the condensation reaction for the intended cleavage.^75^ Further, we use an aryl ring to allow for easy tunability of the electronic properties of the thioether moiety should that be needed and for easy introduction of other functional groups for conjugation and optimization of physicochemical property in later work. The compounds of the initial design are aimed at a demonstration of their feasibility. For this study, we chose to start with five functional groups as our EWGs: an amide, ester, ketone, −CF_3_, or cyano group (Figure 2).

**Figure 2:**
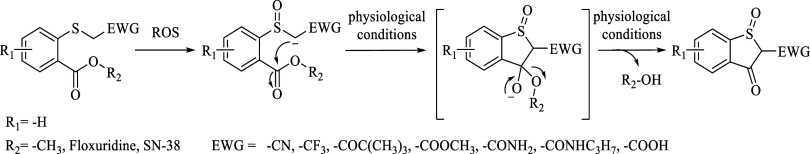
Design of ROS-triggered release via CReKT chemistry.

### 2.2 Synthesis of Model Compounds

The synthesis of the designed model compounds is shown in Scheme 1. Briefly, methyl salicylate was reacted with different alkyl halides (**2a**–**2g**) in the presence of triethylamine at room temperature to obtain thioethers (**3a**–**3g**), which were converted to the corresponding sulfoxides **4a**–**4g** by oxidation with meta-chloroperbenzoic acid (*m*-CPBA). For use as controls and standards, the cyclized products (**5a**–**5g, 6a**–**6g**) were synthesized by using triethylamine in a mixture of methanol and water.

**Scheme 1:**
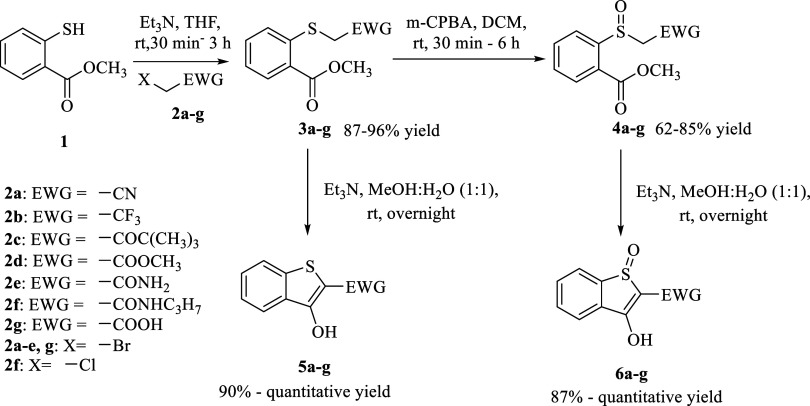
Synthesis of Model Compounds for Cyclization Kinetic Studies

### 2.3 Synthesis of the Floxuridine Prodrug (Flox-CReKT) and SN-38 Prodrug (SN-38-CReKT)

For assessing the feasibility of the prodrug design, we chose two molecules: floxuridine (**Flox**), an FDA-approved anticancer drug, and **SN-38**, the active ingredient of the FDA-approved (pro)­drug irinotecan.^76,77^ Activation of irinotecan to the active principal **SN-38** is known to be inefficient, with 2–5% activation by carboxylesterase and butyrylcholinesterase.^78,79^ This low efficiency in irinotecan activation has led to a high level of interest in developing alternative delivery approaches.^80^ The synthesis of these prodrugs followed similar strategies to those of the model compounds (Scheme 2). Briefly, thiosalicylic acid was reacted with different alkyl halides **2** (**a**, **d**, **e**, **f**) using triethylamine at room temperature to obtain compound **8** (**a**, **d**, **e**, **f**). Then, compound **8** was conjugated with TBDMS protected **Flox** using EDC to yield **11 (a, d, e**, and **f)**. Then the final prodrug **12** (**a**, **d**, **e**, and **f**) was obtained after removing the TBDMS group. The **SN-38** prodrug was synthesized in a similar fashion (Scheme S1).

**Scheme 2:**
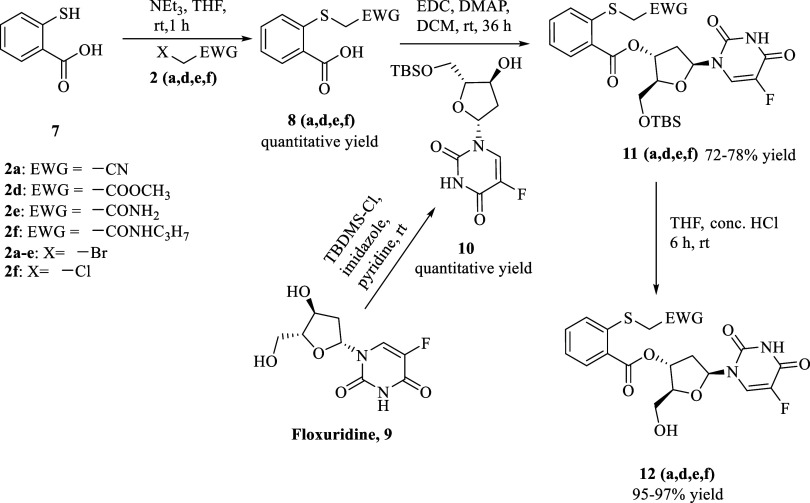
Synthesis of ROS-Sensitive Flox-CReKT Prodrugs

### 2.4 Determination of Cyclization Kinetics

The basic premise of this ROS-sensitive prodrug strategy relies on two properties: (1) the substantial rate enhancement of the cyclization/condensation reaction after oxidation and (2) the ability for ROS to oxidize a thioether to sulfoxide rapidly, which we have recently extensively studied.^44^ To examine the first aspect, we studied the rate of cyclization of the thioethers (**3a**–**3g**) and sulfoxides (**4a**–**4g**) using RP-HPLC (Figure 3 and Figures S1–S5). Briefly, 200 μM thioether or sulfoxide was incubated in PBS (containing 2% DMSO) at 37 °C. Aliquots of the reaction solution were sampled at designated time points for examination of the reaction kinetic profiles. As representative examples, the cases of thioether-sulfoxide pairs **3** *a* **/4a** (EWG: −CN), **3** *e* **/4e** (EWG: −CONH_2_), and **3** *f* **/4f** (EWG: −CONHC_3_H_7_) are described (Figure 3). Figure 3 shows HPLC chromatograms showing the time-dependent disappearance of the starting material (**3/4**) and the appearance of the cyclized product (**5/6**). For example, in Figure 3 and Figure S1, the HPLC chromatograms of thioether **3a** (EWG: −CN) showed a sharp peak at 10 min, which gradually decreased with the formation of the cyclized product (**5a,** retention time (RT) of 10.5 min). In the case of thioether **3a**, the cyclization *t*_1/2_ was determined to be 5 h, and its sulfoxide analog **4a** cyclized with a *t*_1/2_ of 40 min. The large separation of *t*_1/2_ is in line with our central idea of using oxidation to facilitate subsequent cyclization and demonstrates the initial proof of principle. As a subsequent step, we were interested in the ability to tune the reaction kinetics and the magnitude of the reaction rate separation between the thioether and sulfoxide analogs. We are especially interested in searching for systems that offer a high degree of separation in reaction kinetics before and after thioether oxidation.

**Figure 3:**
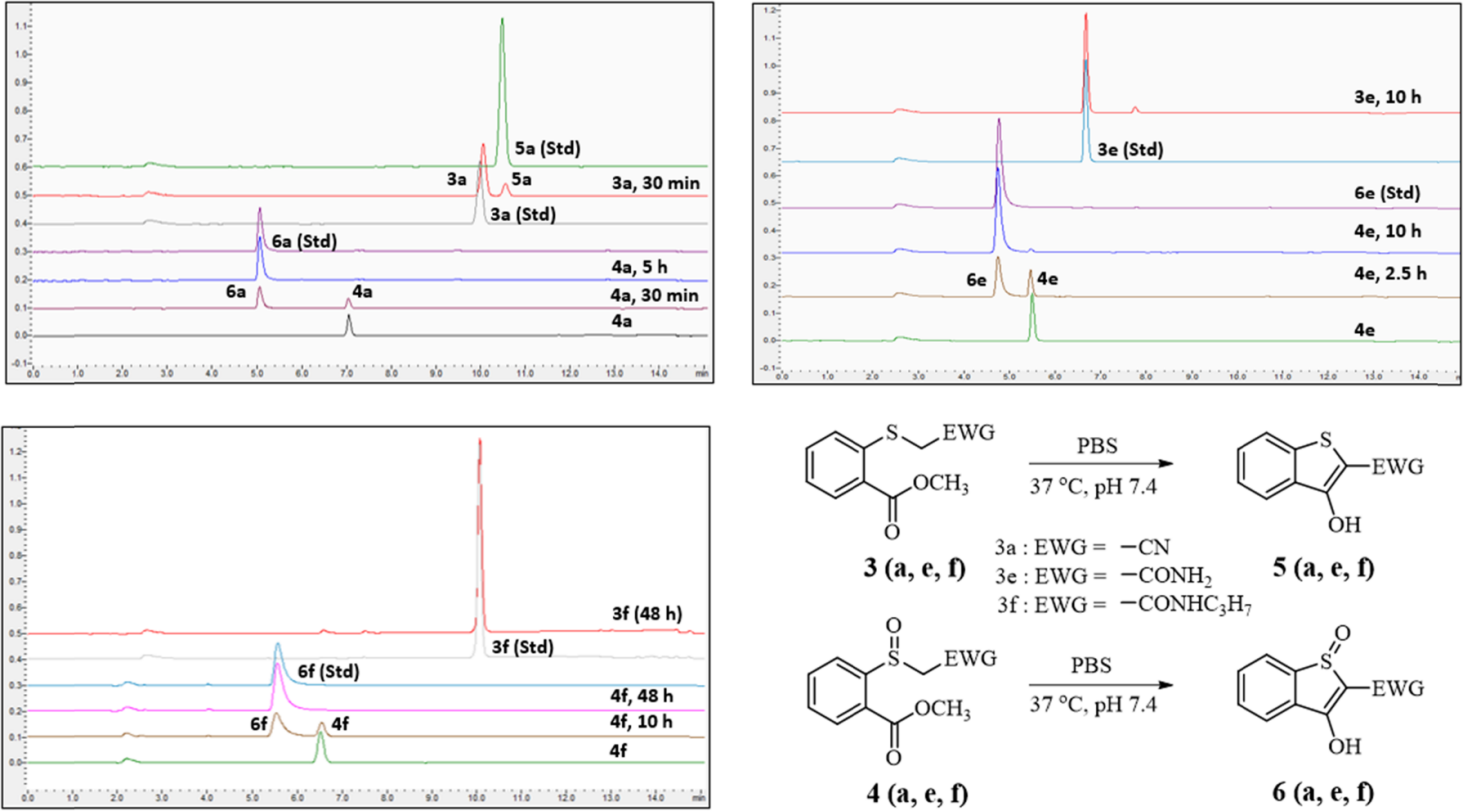
Cyclization kinetics of model thioethers and sulfoxides in near-physiological conditions. A 200 μM compound is incubated in PBS/DMSO (98:2) at 37 °C and pH 7.4.

Next, we examined compounds with different EWGs, including −CF_3_, −COC­(CH_3_)_3_, −COOCH_3_, −COOH, and −CN and two amides (−CONH_2_ and −CONHC_3_H_7_), and compared the cyclization kinetics between the thioether and sulfoxide analogs (Figures S1–S5). These selections cover a wide range of groups with varying characteristics including “enolizable” groups (ketones, ester, and amides), “nonenolizable” EWGs (−CF_3_, −CN), and an ionizable group (−COOH). This limited set of data for the intended feasibility study provides a wealth of information. As a summary, Table 1 lists the *t*_1/2_ values for all of the analogs. First, the idea of creating large separations of cyclization rate before and after oxidation by ROS oxidation of a thioether was indeed accomplished. This is readily apparent from the data for the thioether-sulfoxide pairs **3** *a* **/4a** (EWG = −CN) (*t*_1/2_: 5 h vs 40 min), **3** *c* **/4c** (EWG = −COC­(CH_3_)_3_) (*t*_1/2_: 10 h vs 90 min), and **3** *d* **/4d** (EWG = −COOCH_3_) (*t*_1/2_: 6 h vs 40 min) (Table 1 and Figures S1–S5). Such half-life separations of hours between the thioether and sulfoxide should allow for differential drug release by ROS-mediated oxidation. Second, excitingly, a near-complete “turn on” effect was achieved when the EWG is an amide (**3e** and **3f**). For example, thioether **3e** allowed only about 2% drug release within 12 h, which is very slow and has very little practical meaning in terms of drug delivery, whereas the corresponding sulfoxide (**4e**) rapidly cyclized with a *t*_1/2_ of about 130 min, providing an excellent scenario for prodrug activation (Figure 3). Third, simply having an EWG tethered to the carbon bearing the thioether is not sufficient to afford the desired cyclization effects after oxidation. For example, the thioether-sulfoxide pair **3** *b* **/4b** (−CF_3_) did not lead to cyclization for either the thioether or the sulfoxide analogs. Conceivably, this could be due to the lack of an “enolizable” group. However, the thioether-sulfoxide pair with a nitrile group (**3** *a* **/4a**) is not enolizable either and yet seems to afford ready cyclization of the sulfoxide compound. Further, when the EWG is a carboxyl group (**3** *g* **/4g**), cyclization did not happen (Table 1). This is easy to understand since deprotonation of the α-position of carboxylate is not readily achievable under near-physiological conditions.^81^ Although there is a general trend of faster cyclization with an EWG group of a higher Hammett constant^71^ (σ*p*) (Table 1), there are observations that indicate controlling factors beyond the Hammett constant and whether the EWG is enolizable. For example, both **3e** and **3f** have an amide group and should have similar Hammett constants,^71^ and yet they have very different cyclization kinetics (Table 1). Further, **3a** and **3d** have quite different Hammett constants,^71^ and yet their release rates are similar (Table 1). All of these suggest that the p*K*_a_ of the S-tethered position is not singularly responsible for the final outcome. A larger data set might be able to allow for a more detailed analysis of other controlling factors including steric, electronic, nucleophilicity, and structural idiosyncrasy (e.g., hydrogen bond and conjugation).

**Table 1:** Cyclization Kinetics of the Model Compounds

compound number	EWG	*t*_1/2_ (thioether) (3a–g)	*t*_1/2_ (sulfoxide) (4a–g)	Hammett constant
3/4a	–CN	5 h	40 min	0.66
**3/4b**	–CF_3_	>48 h	>48 h	0.54
**3/4c**	–COC(CH_3_)_3_	10 h	90 min	0.32
**3/4d**	–COOCH_3_	6 h	40 min	0.45
**3/4e**	–CONH_2_	>48 h	130 min	0.36
**3/4f**	CONHC_3_H_7_	no cyclization	7.5 h	
**3/4g**	–COOH	>48 h	>48 h	0.45

From the data described, one thing is certain: the cyclization-driven release of an alcohol from the sulfoxide analogue demonstrated proof of principle for the proposed ROS-sensitive prodrug system. Furthermore, the complete off–on switch from thioether to sulfoxide in the cases of **3/4e** and **3** ***/*** **4f** offers a solution to a critical challenge of existing ROS-triggered linker chemistry, i.e., premature cleavage and the consequent adverse effect in labile linker designs^14,82^ or sluggish release kinetics in highly stable systems.^83^ Specifically, thioether **3e** showed negligible cyclization (<2%) within 12 h, and its sulfoxide **4e** cyclized with a *t*_1/2_ of 130 min (Figure 3). In the case of thioether **3f**, it was completely stable under near-physiological conditions with no significant cyclization observed even after 5 days, and the sulfoxide **4f** cyclized with a *t*_1/2_ of 7.5 h (Figure 3). Overall, these analogues can be used as an off–on switch in ROS-sensitive drug delivery. Next, we assessed the ROS-triggered drug release profiles.

### 2.5 ROS-Triggered Drug Release

Encouraged by the cyclization kinetic results of the model compounds, we initiated drug release studies using drug conjugates. Specifically, we monitored drug release with and without ROS under physiological conditions.

For prodrug activation studies, hypochlorite was used as it is the second most abundant species^27,84–87^ with concentrations reported to be as high as ∼422 μM in liver injury induced by acetaminophen in mouse models.^27,88^ We have recently described with extensive experiments how thioether-based drug delivery systems are likely to be activated by hypochlorite.^44^ Specifically the reaction rate of the slowest thioether analog by hypochlorite was found to be in the range of 10^4^ M^–1^ s^–1^, which is faster than most of the reported click and biorthogonal reactions.^89^ Furthermore, we have described how commonly used solvents such as DMSO and organic buffers rapidly consume hypochlorite, leading to false-negative results and misidentification of ROS.^84,90^ Therefore, in our ROS-related studies, we avoided DMSO^84^ and used 1–2% DMF as cosolvent in PBS. Specifically, to study drug release after oxidation by NaOCl, 100 μM of Flox-CReKT **12a** was incubated with 150 μM sodium hypochlorite (NaOCl) in PBS (containing 1% DMF) under near-physiological conditions (Figure 4). The reaction progress was monitored by RP-HPLC, where 20 μL of the incubated mixture was sampled and analyzed at designated time points (Figure 4). Drug release *t*_1/2_ was calculated by plotting the peak area of **Flox** released against time (Figure 5a). As expected, addition of hypochlorite (150 μM) immediately oxidized thioethers **12a** to their corresponding sulfoxide (Figure 4), consistent with the known fast oxidation of thioether by NaOCl.^86,87^ The fast formation of the sulfoxide species indicates the rate-limiting step being cyclization, as designed. Specifically, oxidation of Flox-CReKT conjugate **12a** is designed to lead to sulfoxide analogue **13a**, which undergoes cyclization to release the **Flox** with the formation of the cyclized byproduct (**6a**) (Figure 4). Indeed, oxidation of **12a** led to the release of 90% **Flox** at the 20 min time point corresponding to a *t*_1/2_ of about 2.5 min at 37 °C and pH 7.4 (Figure 4). Prodrug **12a** with the same linker as model compound **4a** showed faster cyclization than **4a** (*t*_1/2_ of 40 min), indicating the likelihood for the payload to be a factor in influencing the release kinetics. In contrast, in the absence of any ROS, 100 μM Flox-CReKT **12a** released **Flox** with a *t*_1/2_ of around 5 h (Figures 4 and[Fig fig5]a), which is similar to that of model compound **3a** (Figure 3a). Similar drug release kinetics was observed with Flox-CReKT **12d** (Figures S6 and S7). Collectively, these two studies (drug release kinetics with ROS and without ROS) suggest that compound **12a** can be used as a ROS-triggered drug delivery system. Such kind of fast kinetics (*t*_1/2_ of about 2.5 min) is a significant improvement in comparison to existing ROS-sensitive drug delivery systems.^83^

**Figure 4:**
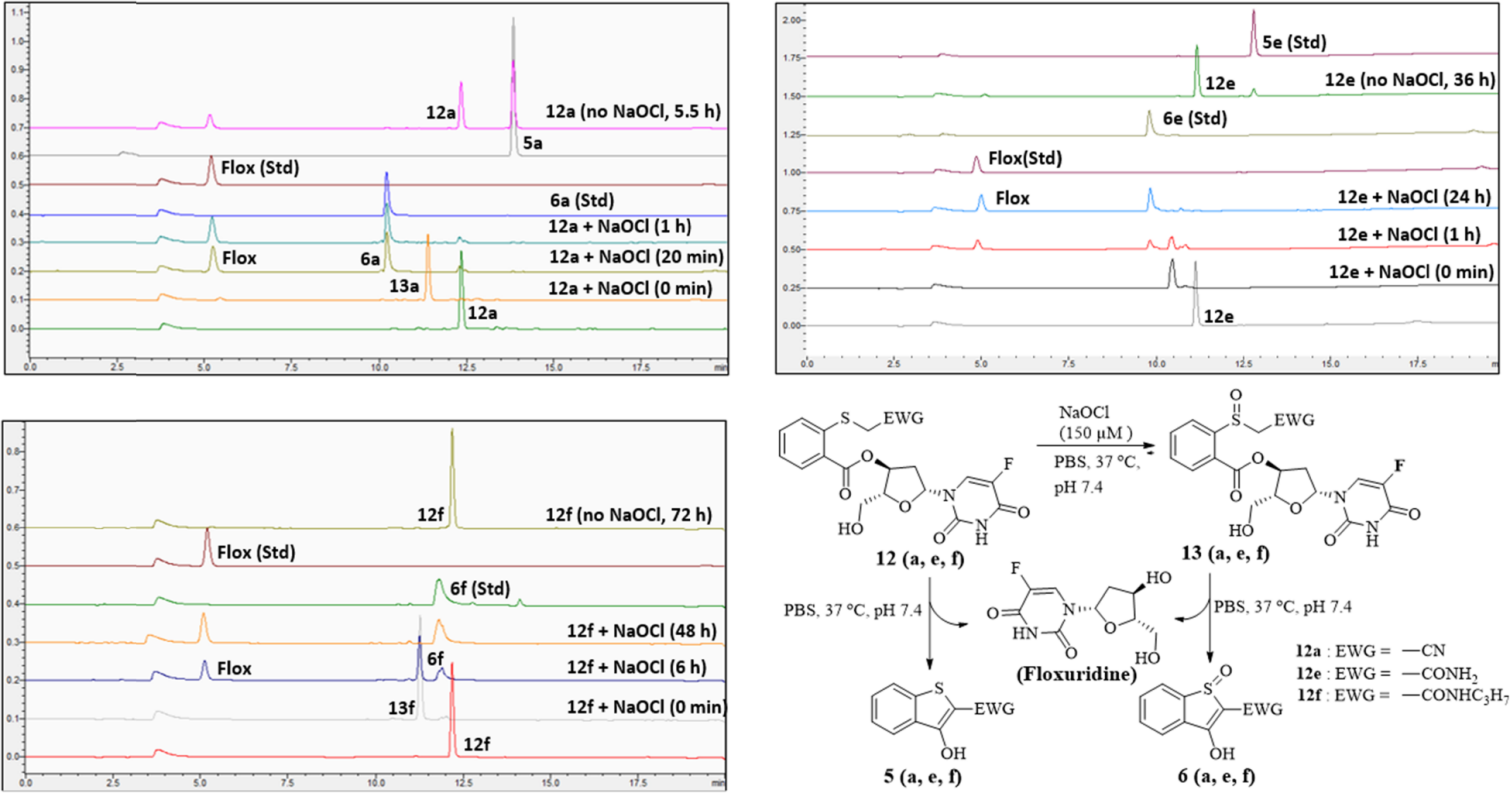
HPLC chromatogram showing the drug release kinetics of 100 μM flox-conjugates (**12a**, **12e**, and **12f**) in the presence or absence of 150 μM NaOCl in PBS (containing 1–2% DMF) at 37 °C and pH 7.4.

**Figure 5:**
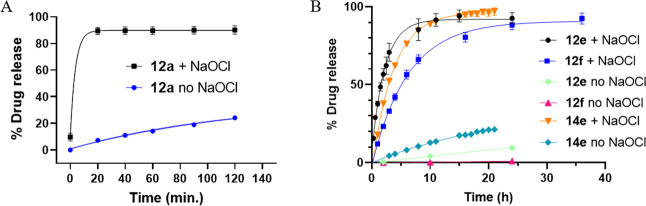
(A) Flox release from **12a** (100 μM) with and without NaOCl (150 μM) under near-physiological conditions (*n* = 3). (B) Flox release from **12e** and **12f** (100 μM) with NaOCl (150 μM) under near-physiological conditions (*n* = 3).

Next, we performed similar release studies with the highly stable Flox conjugates (Flox-CReKT, **12e** and **12f**) with an off–on ROS trigger. In the case of **12e**, the drug conjugate was highly stable in the absence of any ROS, with more than 93% of the conjugate remaining intact after 24 h. When Flox-CReKT (**12e;** 100 μM) was incubated with 150 μM NaOCl, it was rapidly oxidized to the corresponding sulfoxide **13e**, which then released **Flox** with a *t*_1/2_ of 1.5 h (Figure 5b). Compound **14e** having the same linker as **12e** released **SN-38** with a *t*_1/2_ of 2.7 h after oxidation, which is slightly longer than that of **12e**, further indicating the effect of payload on drug release kinetics. Further, **14e** without oxidation also released about 25% of **SN-38** within 24 h (Figures 5 and 6), consistent with the chemically labile nature of a phenolic ester. Such results also indicate the need to optimize specific designs to meet the need of specific applications instead of developing a “one size fits all’ prodrug system, at least in terms of release kinetics and stability. In the case of Flox-CReKT **12f**, the prodrug remained intact in the absence of ROS for up to 72 h (for the duration of the study). In contrast, **12f** released the drug with a *t*_1/2_ of 4.3 h after oxidation (Figures 4 and 5). As discussed earlier, the **Flox**-release rate is faster than that of the corresponding model sulfoxide compound (**6f,** *t*_1/2_ = 7.5 h), which is a desired improvement. Overall, CReKT **12e** and **12f** have shown excellent stability without ROS and fast drug release after oxidation by ROS. Collectively, the drug release studies with and without ROS suggest that CReKT **12e** and **12f** can be used for ROS triggered drug delivery.

**Figure 6:**
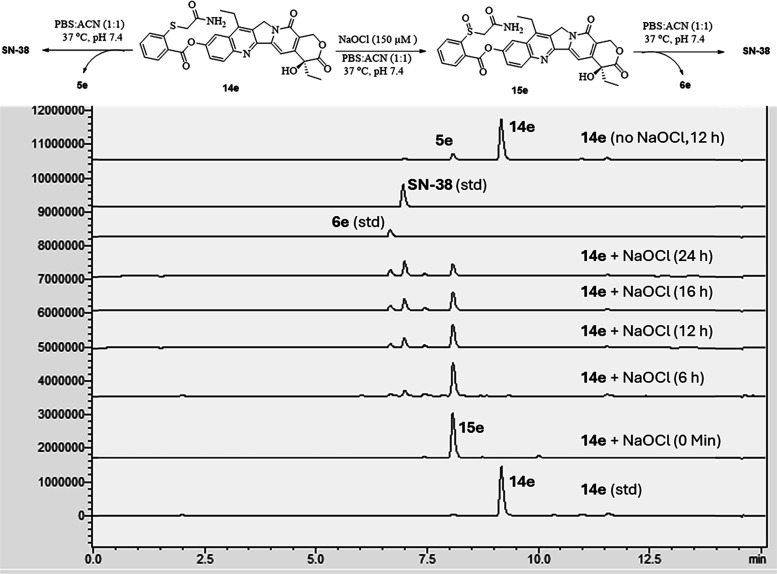
HPLC chromatograms showing drug release kinetics of 100 μM **SN-38** prodrug (**14e**) in the presence or absence of 150 μM NaOCl at 37 °C and pH 7.4.

With the success in demonstrating chemical feasibility, ROS-sensitive activation, and identification of specific structural scaffolds with sufficiently large separations of the release kinetics between the thioether and sulfoxide analogs, we next examined the feasibility of intracellular payload release.

### 2.6 Effect of pH on ROS-Triggered Drug Release

The designed CReKT linker showed the desired stability and drug release profile after oxidation by hROS. Next, we also examined the effect of pH on linker activation and drug release for the following reason.^91^ Cellular compartments can have slightly different pH. For example, the pH has been reported to be 7.2 for cytosol, 6–6.7 for the Golgi apparatus, 8.0 for mitochondria, 7.0 for peroxisomes, 5.5 for secretory granules, 6.3 for early endosomes, 5.5 in late endosomes, and 4.7 in lysosomes.^92^ We used the same protocol as discussed in the previous section of ROS-sensitive drug release to study the effect of pH. Briefly, we incubated 100 μM **12e** with or without 150 μM NaOCl in buffer at various pH (5.5, 6.5, 7.4, and 8.5) and found pH dependency of the cyclization kinetics, as expected (Figure 7). Specifically, the payload release half-life of **12e** was found to be 0.4 h at pH 8.5, 6 h at pH 6.5, 2.2 h at pH 7.4, and 48 h at pH 5.5 in the presence of 150 μM of NaOCl. In the absence of NaOCl, 42% release was found within 20 h at pH 8.5, while little release was found at other pH values (Figures S8–S12). Specifically, prodrug **12e** did not release any drug at pH 5.5 or 6.5 for up to 24 h without ROS (Figures S8–S12), as expected. These results indicate that the linker works as designed with acceleration in cyclization kinetics in line with deprotonation as a critical controlling factor, although other factors may come into play when comparing among different analogs as discussed earlier. Furthermore, we used either a citrate- or PBS-based buffer to test the effect of pH on the oxidation step. It should be noted that we have recently described OCl^–^ consumption by the organic buffers and solvents including DMSO, HEPES, MES, Tris, and ammonium acetate.^84,90^ Citrate can also react with OCl^–^, leading to the formation of alkyl hypochlorite that is still very reactive.^90^ This was the reason that we used citrate-based buffers for studies at pH 5.5 and 6.5 because other organic buffers could lead to false negatives for the reaction of **12e** with OCl^–^.^90^ Specifically, it was found that the oxidation by OCl^–^ was fast regardless of the pH (Figure 7). Overall, the results indicate that the oxidation step is feasible and not the rate-limiting step regardless of the pH and that the linker can release drug in the cytosolic pH with the desired release kinetics.

**Figure 7:**
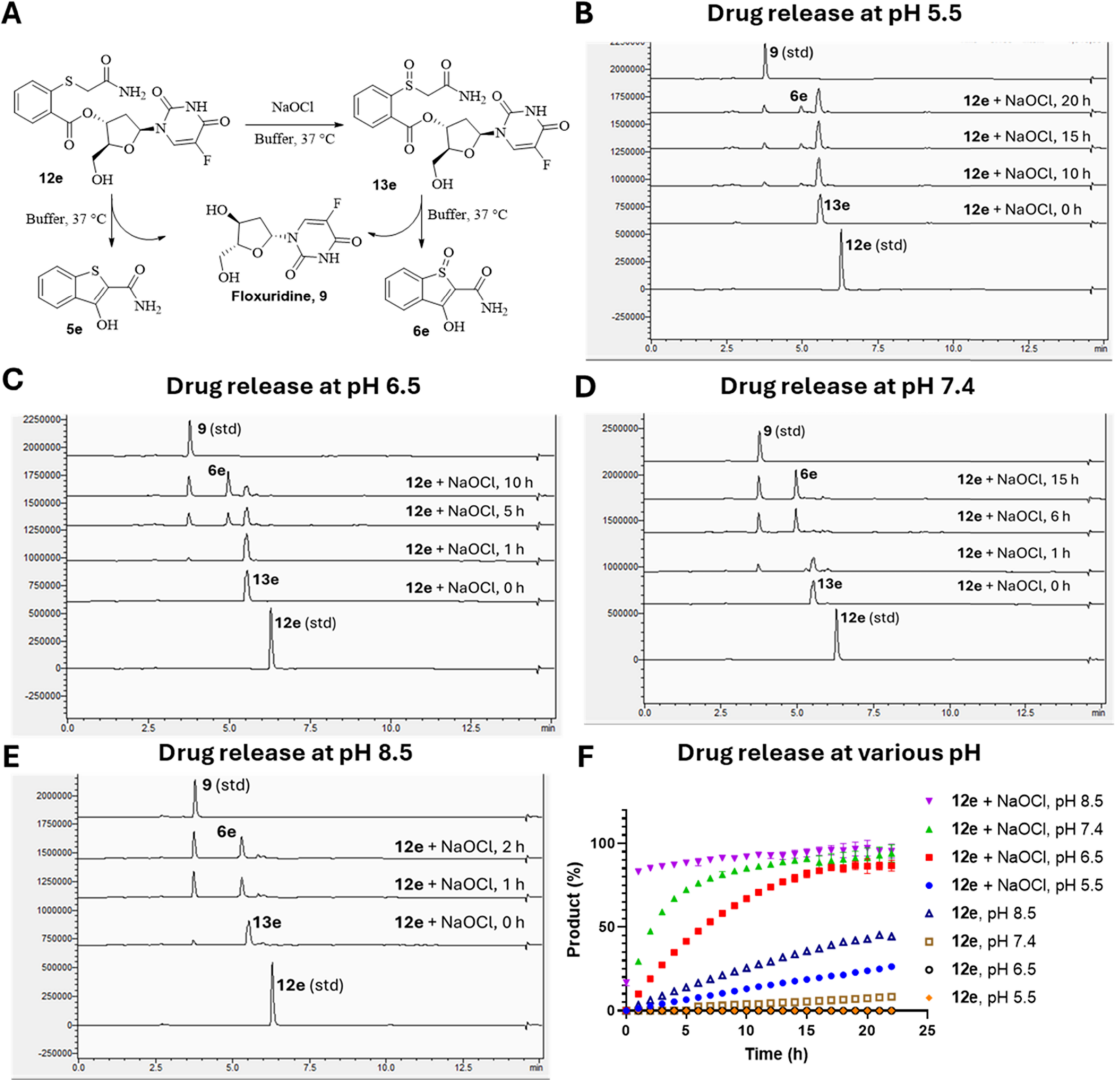
Effect of pH on the ROS-sensitive drug release. (A) General prodrug activation scheme. (B) Drug release from 100 μM **12e** and 150 μM NaOCl at pH 5.5 and 37 °C. (C) Drug release from 100 μM **12e** and 150 μM NaOCl at pH 6.5 and 37 °C. (D) Drug release from 100 μM **12e** and 150 μM NaOCl at pH 7.4 and 37 °C. (E) Drug release from 100 μM **12e** and 150 μM NaOCl at pH 8.5 and 37 °C. (F) Drug release curves from 100 μM **12e** and 150 μM NaOCl at various pH values at 37 °C.

### 2.7 Stability of Prodrug with Other Endogenous Molecules

A key feature needed for the targeted drug delivery is the activation of the prodrug by the desired mechanism. Along this line, we examined the stability of **12e** in the presence of H_2_O_2_, GSH, l-cysteine, and Boc-Lys-OH. Briefly, we incubated 100 μM of **12e** with H_2_O_2_ (1 mM), GSH (1 mM), l-cysteine (100 μM), or Boc-Lys-OH (100 μM) in PBS at pH 7.4 and 37 °C and found the prodrug **12e** to be stable in all such studies (Figure 8). Thioether is known to react sluggishly with H_2_O_2_. Recently, we have reported the second-order rate constant of phenyl thioether reaction with H_2_O_2_ to be in the range of 2.5 × 10^–3^ M^–1^ s^–1^,^44^ corresponding to an estimated *t*_1/2_ of ∼3 days. Indeed, the experimental results align with this half-life estimation (Figure 8). Overall, the results indicate that the prodrug cannot be activated by H_2_O_2_, thiol species, or an amino group, as designed.

**Figure 8:**
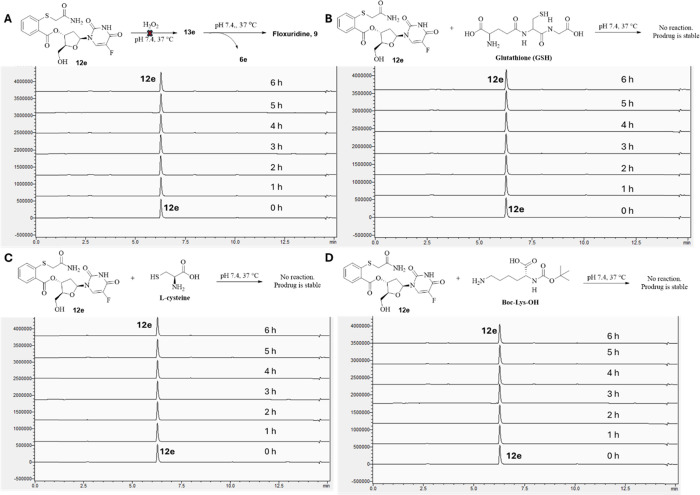
Stability of prodrug **12e** with other endogenous molecules. (A) **12e** (100 μM) with 1 mM H_2_O_2_ at pH 7.4 and 37 °C. (B) **12e** (100 μM) with 1 mM GSH at pH 7.4 and 37 °C. (C) **12e** (100 μM) with 100 μM l-cysteine at pH 7.4 and 37 °C. (D) **12e** (100 μM) with 100 μM Boc-Lys-OH at pH 7.4 and 37 °C.

### 2.8 Intracellular Drug Release under Oxidative Stress

Encouraged by the drug release studies in the buffer, we then studied the intracellular drug release in RAW264.7 (macrophage) cells under inflammatory conditions. This decision was made because of macrophages’ role as the frontline responders of inflammation and their role in the production of ROS.^93^ Because of the much higher level of complexity in the intracellular environment compared to solution-phase studies, we were careful in selecting the prodrug that showed the maximal release rate difference before and after oxidation to sulfoxide. Further, it is desirable to have fast release after conversion to the sulfoxide analogue for subsequent cytotoxicity studies. Because of the significant extent of drug release from the **SN-38** prodrug before ROS oxidation presumably due to the labile nature of a phenol ester group (Figure 5), we decided to use a **Flox** prodrug for the intracellular release studies.

For all these reasons, we chose Flox-CReKT (**12e**) for studies of intracellular release with and without stimulation for inflammatory responses, which are known to cause overproduction of ROS.^94,95^ Before studying ROS-sensitive release, we first examined the stability of our compounds in the presence of a representative esterase to eliminate direct hydrolysis as a mechanism for drug release. Briefly, Flox-CReKT (**12e**) was incubated with porcine liver esterase (PLE, 1 U/mL). No drug release via hydrolysis was observed in 2 h, the duration of the cell culture experiments (Figures S12 and S13). After that, we determined the intracellular drug release in RAW264.7 cells by incubating the prodrug with and without stimulants lipopolysaccharides (LPS) and phorbol 12-myristate 13-acetate (PMA) for their known ability to induce inflammatory responses and thus ROS production.^94,95^ Specifically, RAW264.7 cells were incubated with 50 μM CReKT **12e** in the presence of LPS (2 μg/mL) or PMA (2 μM). In the control experiments, RAW264.7 cells were incubated with 50 μM **12e** without a stimulant (PMA or LPS). After 2 h of incubation, the medium was discarded, and the cells were washed with cold water (3 × 3 mL) before centrifugation at 250*g* to give cell pellets for lysis with 200 μL of cold ACN. Then, the lysates were sonicated for 1 min at room temperature and centrifuged at 14,000 rpm for 10 min at 4 °C. 2’-Deoxyuridine (dURD, 50 μM) was used as an internal standard (Figures S14 and S15). The peak area ratio of **Flox** and the internal standard (dURD) from LCMS was used to build a standard curve for quantification (Figure S14). As expected, the drug release was observed to be higher upon stimulation (LPS or PMA). Briefly, **12e** showed 6-fold higher drug release with PMA stimulation and 8-fold higher drug release with LPS stimulation (Figure 9) when compared with the controls without stimulation. The intracellular drug release studies clearly demonstrate the key aspect of drug release and the utility of the designed CReKT system for ROS-triggered drug delivery in cell culture models.

**Figure 9:**
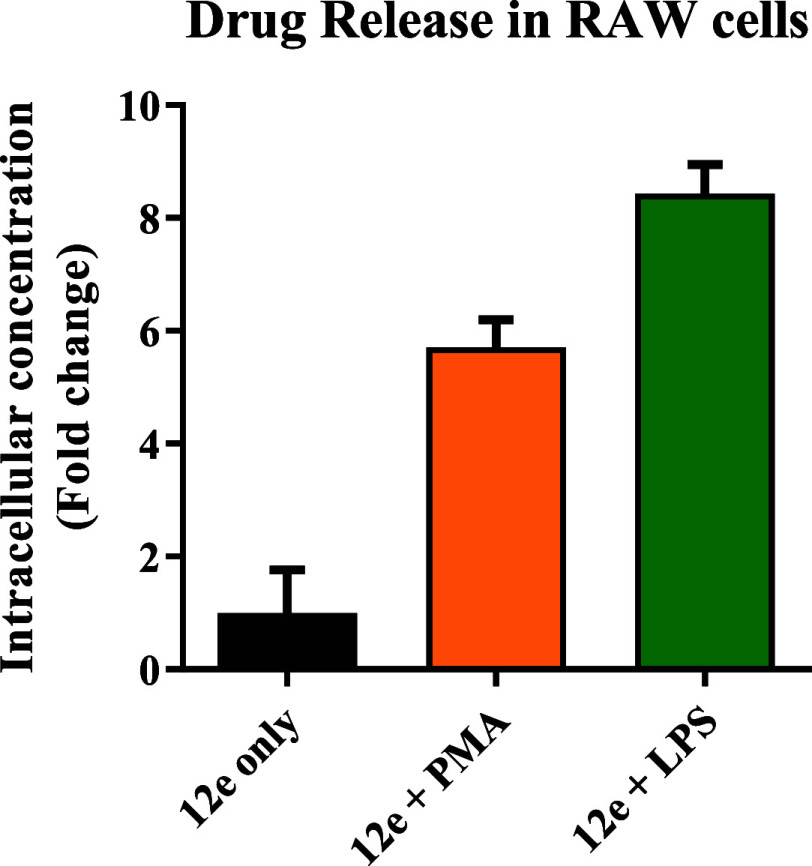
Intracellular drug release by CReKT **12e** in RAW264.7 cells.

### 2.9 Intracellular ROS Detection Using a Fluorescent Probe

To correlate the intracellular drug release, we also studied intracellular ROS formation upon stimulation with LPS or PMA or using RAW264.7 cells. We chose one of the widely used fluorescent probes, DCFH-DA, which is the diester version of DCFH capable of cellular penetration.^96–98^ Subsequent hydrolysis of the ester groups would lead to DCFH, which is not readily cell membrane permeable and thus is trapped intracellularly.^99^ It should be noted that DCFH reacts rapidly with hypochlorite^90^ or a species with similar reactivity.^100^ On the other hand, DCFH does not react with H_2_O_2_.^84,101^ Specifically, DCFH reacts with hypochlorite to generate a fluorescent product with a second-order rate constant of 580 ± 18 M^–1^ s^–1^.^90^ With such a fast reaction, one can assume that the majority of the ROS detected has similar or higher reactivity compared with hypochlorite.^84^ Similar to the intracellular payload release studies, we used RAW264.7 cells for intracellular ROS determination work. Briefly, 10 μM of DCFH-DA was incubated with and without LPS/PMA. Specifically, three groups were tested with the first being 10 μM of DCFH-DA only, the second being 10 μM of DCFH-DA and 2 μg/mL of LPS, and the third being 10 μM of DCFH-DA and 2 μM of PMA. The cells were incubated for 4 h before fluorescence measurements using a plate reader (λ_ex_ 495 nm and λ_em_ 530 nm). Indeed, a significantly higher fluorescence intensity was found when cells were stimulated with PMA (Figure 10). However, when incubated with LPS, the probe showed only a marginal increase in fluorescent intensity (Figure 10). Similar differences in the ROS production have been observed with hydroethidine (dihydroethidium) or DCFH-DA probe in RAW264.7 cells as well.^102,103^ In analyzing the results, we should note that fluorescence turn-on of the DCFH probe requires oxidation of a C–H bond instead of thioether oxidation, as in the prodrug activation process. The second-order rate constant of thioether oxidation by NaOCl (10^8^ M^–1^ s^–1^)^86,87^ is faster by 6 orders of magnitude than DCFH oxidation (580 ± 18 M^–1^ s^–1^),^90^ which could explain the significantly higher drug release after LPS stimulation even though DCFH only detected a marginal increase (Figure 10). Overall, the results are consistent with the results from intracellular drug release, with the conclusion that PMA/LPS causes elevated ROS production and increases drug release.

**Figure 10:**
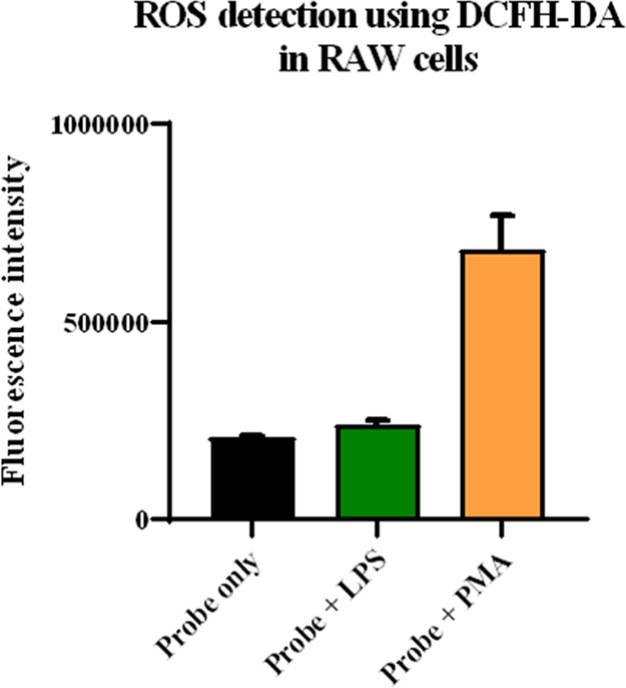
Intracellular ROS detection using DCFH-DA.

### 2.10 Biological Activity

The success in achieving ROS-triggered intracellular drug release encouraged us to initiate cytotoxicity studies using the same prodrug, Flox-CReKT (**12e**) (Figure 9), in RS4;11 cells (lymphoblast B-cells) with and without a stimulant. RS4;11 was chosen because of prior literature studies of **Flox** toxicity in leukemia cells.^104–106^ Our studies indeed confirm that RS4;11 is a very good cell line to use for the study. The active principal and the cyclized compound (**5e**) after drug release were used as positive and negative controls, respectively. As expected, the prodrug **12e** showed an IC_50_ value (63 nM) that is 9-fold higher than that of **Flox** itself (7 nM) (Table 2 and Figure 11). Such results agree with the design principle that the prodrug is in an inactive form or has reduced potency. Further, it is possible that **12e** experienced a basal level of activation via hydrolysis, as can be seen from our HPLC studies (Figures 4 and 5).

**Table 2:** IC_50_ Values of **12** *e* **/12f/Floxuridine/3** *e* **/3f** with or without H_2_O_2_

treatment	IC_50_ (nM)
**12e**	63 ± 3
**12e** + 25 μM H_2_O_2_	24 ± 3
**12f**	197 ± 3
**12f** + 25 μM H_2_O_2_	202 ± 3
**floxuridine**	7 ± 2
**floxuridine** + 25 μM H_2_O_2_	7 ± 3
**3e**	>10 μM
**3e** + H_2_O_2_	>10 μM
**3f**	>10 μM
**3f** + H_2_O_2_	>10 μM

**Figure 11:**
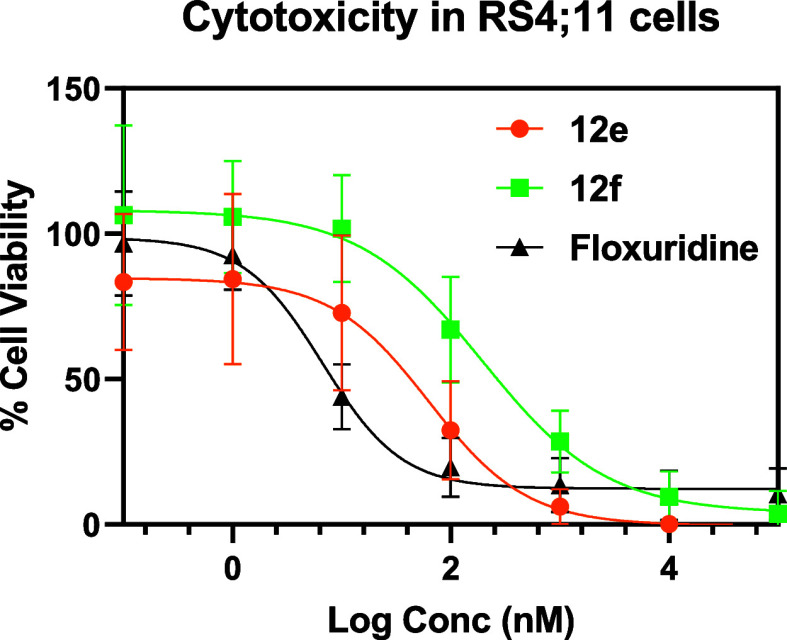
Dose–response curves of prodrug **12e**–**f** and **floxuridine**.

In literature studies of stimulating cancer cells to create oxidative stress and produce hROS, H_2_O_2_ is the most commonly used. For example, a recent systematic analysis of H_2_O_2_ usage for oxidative stress in the literature found that more than 84% of papers have used more than 100 μM H_2_O_2_ based on the first 112 relevant papers from a PubMed search.^107^ We should note that H_2_O_2_ at the tested concentrations cannot oxidize thioether because of the sluggish second-order rate constant.^44^ Therefore, we do not expect more than minimal direct oxidation of prodrug **12e** by H_2_O_2._ The expectation was that H_2_O_2_ stimulation would lead to the production of hROS.^108,109^ When **12e** was incubated with RS4;11 cells stimulated by 25 μM H_2_O_2_, the IC_50_ value improved from 63 to 24 nM (Table 2 and Figure S16). Such results indicate the formation of hROS, leading to prodrug activation, as designed. We should also note that prodrug **12f** did not show similar responses (Table 2 and Figure S16). We attribute such results to the slow drug release kinetics of **12f**, which is further exacerbated by the slow kinetics of its phosphorylation by kinase.^110^ Such results explain the differences in the potency under the stressed conditions. The negative controls **3e** and **3f** with or without H_2_O_2_ did not show any cytotoxicity (IC_50_ higher than 10 μM) (Table 2 and Figures S17 and S18), indicating that the linker portion of the prodrug does not have any contribution to the IC_50_ of prodrugs **12e** and **12f**. Overall, the results indicate that the prodrug approach works as designed.

## 3 Conclusions

Overall, we have demonstrated the feasibility of a novel type of ROS-sensitive prodrug via a ROS-initiated Cascade Reaction with Kinetic Tunability (CReKT). The basic design idea is to move away from relying purely on the reactivity with ROS for drug release. Instead, we focus on creating synergy among three factors: ROS-mediated oxidation, modulation of the reactivity of a neighboring group, and a proximity-driven reaction. In doing so, we were able to use a stable “noncleavable” thioether group to create a “cleavable” mechanism for drug release and design prodrugs that separate ROS sensitivity from drug release kinetics, allowing for stable prodrugs with kinetic tunability.

Solution-phase studies with different EWGs including the ketone, amide, ester, cyano, and trifluoromethyl groups demonstrated the general applicability of the design concept. Furthermore, the “turn-on” effects of ROS oxidation vary depending on the EWG used, leaving much room for kinetic tunability. In ROS-sensitive release studies, rapid oxidation was observed in solution by hypochlorite (not H_2_O_2_) and in cell culture after stimulation with LPS, PMA, and H_2_O_2_, which are known to lead to the generation of highly reactive ROS. In intracellular release studies, cellular stimulation by LPS and PMA was shown to significantly facilitate drug release, as designed. In efficacy studies, we chose a prodrug of **Flox** (**12e**) with maximal separation for the release rate before and after oxidation. Further, **Flox** is a known anticancer agent and has been extensively studied for its effects in leukemia cells. Stimulation of RS4;11 cells with H_2_O_2_ was shown to significantly increase the cytotoxicity of the prodrug (**12e**), in agreement with ROS-mediated drug release. We further studied hROS generation upon stimulation using LPS or PMA in RAW264.7 using a fluorescent probe (DCFH-DA) that is intracellularly trapped and does not react with H_2_O_2._ Fluorescence results clearly demonstrated the generation of highly reactive ROS, which is consistent with literature results, the proposed mechanism of CReKT prodrug activation, the observed intracellular drug release, and the enhanced cytotoxicity of prodrug **12e** upon stimulation.

Overall, all of the results clearly demonstrate proof of principle of the CReKT prodrug chemistry, which offers broad application potential, prodrug stability, and kinetic tunability. Further, the same concept could be extended to other ROS-sensitive fluorescent probe or prodrug designs, allowing for the use of stable prodrugs without the need for highly reactive boronate (an electrophile) or selenide functional groups. It should be emphasized again that hypochlorite production is limited to certain immune cells. Other cell types include solid tumor (benign or malignant) cells that do not express MPO, which is needed for hypochlorite production. In animal models, hypochlorite in cancer and inflamed sites comes from the infiltration by neutrophils and macrophages. However, such experiments are difficult to simulate in a cell culture. Therefore, care is needed in designing appropriate cell-culture experiments and selecting appropriate cytotoxic payloads.

## 4 Experimental Section

### 4.1 Materials and Methods

All reagents and solvents were reagent grade and purchased from Sigma-Aldrich (Massachusetts, USA) or Oakwood Products, Inc. (South Carolina, USA). Column chromatography was carried out using silica gel (Sorbent Technologies (Georgia, USA) 230–400 mesh). RS4;11 cells and RAW264.7 cells were purchased from ATCC (Virginia, USA). FBS was purchased from Corning (New York, USA). TLC analyses were conducted on silica gel plates (Sorbent Technologies (Georgia, USA) Silica XHL TLC plates w/UV254). ^1^H NMR (400 MHz) and ^13^C NMR (100 MHz) spectra were recorded on a Bruker Avance 400 MHz NMR spectrometer in deuterated solvent from Oakwood Products, Inc. (South Carolina, USA). Chemical shifts were reported as δ values (ppm). TMS (δ = 0.00 ppm) or residual peaks of the deuterated solvent were used as the internal reference. Mass spectrometric analyses were conducted by the Georgia State University Mass Spectrometry Facilities. The milligram scale quantities were weighed on a C-33 microbalance (CAHN Instruments Inc., California, USA). HPLC was performed on a Shimadzu LC-20AT or Agilent HPLC system. Column: C18, 5 μm, 4.6 × 150 mm; detector: DAD monitored at 254 nm. All compounds are >95% pure by HPLC (Figures S19–S21).

The mobile phase used was made of A: H_2_O (0.1% TFA) and B: ACN (0.1% TFA). Gradient methods used were as follows: (method A) 95% B, 0–10 min; 95% B, 10–12 min; 5% B, 12–12.1 min; 5% B, 12.1–15 min; (method B) 30–50% B, 0–10 min; 50% B, 10–12 min; 30% B, 12–12.1 min; 30% B, 12.1–15 min; and (method C) 4% B, 0–5.1 min; 4–95% B, 5.1–15 min; 95% B, 15–17.5 min; 4% B, 17.5–17.6 min; 4% B, 17.6–20 min.

### 4.2 HPLC studies

#### 4.2.1 Cyclization Studies of Model Compounds

First, 10 mM stock solutions of compound **3** or **4** were prepared in DMSO. Then, 10 μL was taken from the stock solution and added to 980 μL of PBS at pH 7.4. The resultant solution was kept at 37 °C. At different time points, 20 μL was taken from the reaction mixture and injected into HPLC. Either gradient method A or B was used to monitor the reaction. The reaction mixture had a final concentration of 200 μM in PBS with 2% DMSO.

#### 4.2.2 Release Studies of Drug-Conjugated Compounds

To monitor drug release in the absence of ROS, the same procedure of the model compound was followed with a small change of stock solution solvent. For drug conjugates **12,** DMF was used to prepare the stock solution. Briefly, a 10 mM stock solution of the drug-conjugated compound was prepared in DMF. Then, 10 μL was taken from the stock solution and added to 990 μL of the buffer. The resultant solution was kept at 37 °C. At different time points, 20 μL was taken from the reaction mixture and injected into HPLC. Gradient method C was used to monitor the floxuridine release. The reaction mixture had a final concentration of 100 μM in DMF/PBS (1:99) under near-physiological conditions.

To monitor the drug release in the presence of ROS, drug conjugate **12** was incubated with ROS under near-physiological conditions. First, a 10 mM stock solution of the drug conjugate was prepared in DMF. Then, 10 μL was taken from the stock solution and added to 975 μL of the buffer. Then, 15 μL of 10 mM ROS solution was added to the solution. The resultant solution was kept at 37 °C. At different time points, 20 μL was taken from the reaction mixture and injected into HPLC. Gradient method C was used to monitor the floxuridine release. The reaction mixture had a final concentration of 100 μM of compound and 150 μM of ROS in PBS with 1% DMF under near-physiological conditions.

#### 4.2.3 Stability of the Drug-Conjugated Compound with Endogenous Metabolites

Briefly, a 10 mM stock solution of **12e** was prepared in DMF. Then, 50 mM GSH, 10 mM l-cysteine, or 50 mM Boc-Lys-OH was prepared in H_2_O.

##### 4.2.3.1 Stability with H_2_O_2_

A 10 μL portion was taken from the **12e** stock solution and added to 890 μL of the buffer. Then, 100 μL of a 10 mM H_2_O_2_ solution was added to the buffer solution. The resultant solution was kept at 37 °C. At different time points, 20 μL was taken from the reaction mixture and injected into HPLC. Gradient method A was used to monitor the floxuridine release. The reaction mixture had a final concentration of 100 μM compound and 1 mM H_2_O_2_ in PBS with 1% DMF at pH 7.4 and 37 °C.

##### 4.2.3.2 Stability with GSH

A 10 μL portion was taken from the **12e** stock solution and added to 970 μL of the buffer. Then, 20 μL of 50 mM GSH solution was added to the buffer solution. The resultant solution was kept at 37 °C. At different time points, 20 μL was taken from the reaction mixture and injected into HPLC. Gradient method A was used to monitor the floxuridine release. The reaction mixture had a final concentration of 100 μM of compound and 1 mM of GSH in PBS with 1% DMF at pH 7.4 and 37 °C.

##### 4.2.3.3 Stability with l-Cysteine

A 10 μL portion was taken from the **12e** stock solution and added to 980 μL of the buffer. Then, 10 μL of 10 mM l-cysteine solution was added to the buffer solution. The resultant solution was kept at 37 °C. At different time points, 20 μL was taken from the reaction mixture and injected into HPLC. Gradient method A was used to monitor the floxuridine release. The reaction mixture had a final concentration of 100 μM of compound and 100 μM of l-cysteine in PBS with 1% DMF at pH 7.4 and 37 °C.

##### 4.2.3.4 Stability with Boc-Lys-OH

A 10 μL portion was taken from the **12e** stock solution and added to 988 μL of the buffer. Then, 2 μL of a 10 mM Boc-Lys-OH solution was added to the buffer solution. The resultant solution was kept at 37 °C. At different time points, 20 μL was taken from the reaction mixture and injected into HPLC. Gradient method A was used to monitor the floxuridine release. The reaction mixture had a final concentration of 100 μM compound and 100 μM Boc-Lys-OH in PBS with 1% DMF at pH 7.4 and 37 °C.

### 4.3 Quantification of Floxuridine in RAW264.7 Cells under Inflamed Conditions Using LCMS

#### 4.3.1 Stock Solutions and Working Solution of Calibration Standards

Stock solutions of the internal standard (2′-deoxyuridine) and floxuridine were prepared at 1 mM concentration in 50% ACN. The internal standard working solution was diluted from internal standard stock solution with 50% MeOH (0.1% FA) at 50 μM concentration. Floxuridine working solutions were diluted from stock solution with internal standard working solution (50 μM) to make concentrations of 100 nM, 1 μM, 5 μM, 10 μM, 50 μM, and 100 μM. Stock solutions and working solutions were stored at 4 °C.

#### 4.3.2 LC Conditions

LC analyses were performed on a Waters Xevo TQ-s micro-Mass Spectrometer (Waters Corporate, Milford, MA) equipped with a Waters Acquity UPLC H-Class Plus LC system using a Phenomenex Gemini NX-C18 100 × 3 mm, 3 μm column. Mobile phase A was water with 0.1% formic acid, and mobile phase B was acetonitrile with 0.1% formic acid. The mobile phase was 1% B and held for 10 min. The flow rate was 200 μL/min. The injection volumes of standards and samples were 5 μL.

#### 4.3.3 MS Conditions

Ionization and detection of analytes were performed using an ion spray interface and in negative ionization mode. The instrument operation parameters were optimized as follows: capillary voltage of −4.0 kV, desolvation temperature of 350 °C, and a source temperature of 120 °C. Nitrogen was used as nebulizing and drying gas on pressures of 10 and 650 psi, respectively. The optimized MS parameters are reported in Table S1.

#### 4.3.4 Sample Preparations

RAW264.7 cells were seeded in a 100 mm Petri dish and were allowed to achieve over 80% confluency before the experiment. After that, the cells were treated with 50 μM compound for 2 h in 10 mL of DMEM. After the incubation, the culture medium was discarded, and the cells were washed with ice-cold water (3 × 3 mL). Further, 1 mL of cold water was added to the dish, and the cells’ monolayer was scraped gently using a cell spatula. Afterward, the cell suspended solution was collected in a 1.5 mL Eppendorf tube and centrifuged at 250*g* for 4 min at 4 °C. The supernatant was removed, and cell pellets were resuspended in 200 μL of cold ACN. The lysed cells were sonicated in the Eppendorf tube for 1 min at room temperature and then centrifuged at 14,000*g* for 10 min at 4 °C. Then, 199 μL of the supernatant was taken, and to that supernatant, 1 μL of the internal standard (2′-deoxyuridine) from 10 mM stock solution. The internal standard concentration in the sample was 50 μM, which was used to prepare the standard curve (Table S2 and Figures S14 and 15) Finally, 100 μL of this solution was taken in an HPLC vial containing an insert for the LCMS study.

### 4.4 Intracellular ROS Detection Using the Fluorescent Turn-On Probe

First, stock solutions were prepared by using DMF. DCFH-DA (10 mM) was prepared by dissolving 3.392 mg in 696 μL of DMF. PMA (10 mM) was prepared by dissolving 1.128 mg in 183 μL of DMF. LPS (2 μg/μL) was prepared by dissolving 1.610 mg in 805 μL of H_2_O.

Working solutions were prepared by dilution in DMEM (Fluorobright DMEM, containing 1% PNS). DCFH-DA (10 μM) was prepared by adding 4 μL of 10 mM DCFH-DA in 3.996 mL of media. DCFH-DA (10 μM) and LPS (2 μg/mL) were prepared by adding 4 μL of 10 mM DCFH-DA and 4 μL of 2 μg/μL of LPS in 3.994 mL of media. DCFH-DA (10 μM) and PMA (2 μM) were prepared by adding 4 μL of 10 mM DCFH-DA and 0.8 μL of 10 mM PMA in 3.995 mL of media. Simultaneously, a 96-well plate having RAW264.7 cells was prepared to have over 80% confluency (DMEM, containing 10% FBS and 1% PNS). From the 96-well plate having RAW264.7 cells, the medium was removed, and 200 μL of the probe solution with and without stimulant was added to each designated well. RAW264.7 cells were finally incubated in three groups. The first group had 10 μM DCFH-DA only, the second group had 10 μM of DCFH-DA and 2 μg/mL of LPS, and the third group had 10 μM of DCFH-DA and 2 μM of PMA. Subsequently, the plate was incubated 4 h before fluorescence measurements using a plate reader (λ_ex_ 495 nm and λ_em_ 530 nm). The experiment was conducted in triplicates.

### 4.5 Cytotoxicity Studies

To conduct this set of experiments, a 96-well plate (8 × 12) was used. First, RS4;11 cells were seeded in a 96-well plate 1 day before the experiment (RPMI-1640, containing 10% FBS and 1% PNS). Then, the cells were incubated with different concentrations of compounds with or without the stimulant. Specifically, the final concentrations of compounds were 0.1 pM–100 μM with or without H_2_O_2_ (25 μM) stimulation. The plate containing cells were incubated for 72 h at 37 °C with 5% CO_2_. The cell viability was tested using the CCK-8 assay. Specifically, after 72 h of incubation, 10 μL of the CCK-8 assay reagent was added to each well. Then, the plate was further incubated for 2 h. After that, the plate was gently shaken for 1 min, and then absorbance was recorded at 450 nm. The percentage cell viability was calculated using the following formula: cell viability (%) = ((*A*_450_ of treated wells – *A*_450_ of control wells)/(*A*_450_ of no treatment wells – *A*_450_ of control wells)) × 100. The experiment was conducted in triplicates. The IC_50_ values have been summarized in Table 2, and the dose–response curves are shown in Figure 8 and Figures S12–S14.

### 4.6 Chemistry

#### 4.6.1 General Synthesis for Compound **3**(**a**–**j**)

To a solution of methylthiosalicylate **1** (1 equiv) in 50 mL of THF, **2**(**a**–**j**) (1.1 equiv) and then triethylamine were added (1.1 equiv). The resulting reaction mixture was stirred at room temperature for 30 min. Then, the reaction mixture was concentrated *in vacuo*, dissolved in DCM, and washed with 1 N HCl and brine. The combined organic layers (3 × 50 mL) were dried over anhydrous Na_2_SO_4_ and concentrated *in vacuo*. The crude product was purified by column chromatography (silica gel and hexane/ethyl acetate) to afford the desired product.

#### 4.6.2 Methyl 2-((Cyanomethyl)­thio)­benzoate (**3a**)

Isolated yield 96%. ^1^H NMR (CDCl_3_): δ 8.05 (d, *J* = 7.7 Hz, 1H), 7.59 (t, *J* = 6.9 Hz, 1H), 7.43 (d, *J* = 7.9 Hz, 1H), 7.34 (t, *J* = 7.4 Hz, 1H), 3.94 (s, 3H), 3.74 (s, 2H). ^13^C NMR (CDCl_3_): δ 166.7, 137.0, 133.1, 131.6, 128.6, 126.8, 126.1, 77.4, 52.48, 18.5. HRMS calculated for C_10_H_9_NO_2_SNa [M + Na]^+^ 230.0252; found: 230.0585. Data match with the literature reports.^111^

#### 4.6.3 Methyl 2-((2,2,2-Trifluoroethyl)­thio)­benzoate­(**3b**)

Isolated yield 90%. ^1^H NMR (CDCl_3_): δ 7.96 (dd, *J* = 7.9, 0.9 Hz, 1H), 7.57–7.40 (m, 2H), 7.37–7.16 (m, 1H), 3.95 (s, 3H), 3.60 (q, *J* = 9.7 Hz, 2H). ^13^C NMR (CDCl_3_): δ 166.9, 137.1, 132.5, 131.2, 130.0, 128.4, 126.1, 126.9, 52.4, 35.9. HRMS calcd for C_10_H_10_F_3_O_2_S [M + H]^+^ 251.0354; found 251.0348. Data match with the literature reports.^112^

#### 4.6.4 Methyl 2-((3,3-Dimethyl-2-oxobutyl)­thio)­benzoate (**3c**)

Isolated yield 93%. ^1^H NMR (CDCl_3_): δ 7.89 (dd, *J* = 7.8, 1.4 Hz, 1H), 7.43–7.31 (m, 1H), 7.25 (d, *J* = 7.8 Hz, 1H), 7.17–7.07 (m, 1H), 3.95 (d, *J* = 10.0 Hz, 2H), 3.86 (s, 3H), 1.20 (s, 9H). ^13^C NMR (CDCl_3_): δ 209.3, 166.7, 140.1, 132.2, 131.0, 128.2, 126.6, 124.5, 52.0, 44.4, 38.4, 26.5. HRMS calcd for C_14_H_19_O_3_S [M + H]^+^ 267.1055; found 267.1057.

#### 4.6.5 Methyl 2-((2-Methoxy-2-oxoethyl)­thio)­benzoate (**3d**)

Isolated yield 96%. ^1^H NMR (CDCl_3_): δ 7.97 (d, *J* = 7.8 Hz, 1H), 7.45 (t, *J* = 7.7 Hz, 1H), 7.37 (d, *J* = 8.1 Hz, 1H), 7.19 (t, *J* = 7.5 Hz, 1H), 3.90 (s, 3H), 3.72 (d, *J* = 2.5 Hz, 5H). ^13^C NMR (CDCl_3_): δ 170.0, 166.8, 140.2, 132.8, 131.4, 127.8, 125.9, 124.8, 52.79, 52.30, 34.83. HRMS calcd for C_11_H_13_O_4_S [M + H]^+^ 241.0535; found 241.0531. Data match with the literature reports.^113^

#### 4.6.6 Methyl 2-((2-Amino-2-oxoethyl)­thio)­benzoate (**3e**)

Isolated yield 91%. ^1^H NMR (CD_3_OD): δ 7.96 (d, *J* = 7.8 Hz, 1H), 7.52 (t, *J* = 7.7 Hz, 1H), 7.44 (d, *J* = 8.1 Hz, 1H), 7.26 (t, *J* = 7.5 Hz, 1H), 3.91 (s, 3H), 3.72 (s, 2H). ^13^C NMR (CD_3_OD): δ 172.6, 166.9, 140.1, 132.4, 130.8, 127.8, 125.9, 124.4, 53.4, 51.2, 35.4. HRMS calcd for C_10_H_12_NO_3_S [M + H]^+^ 226.0538; found 226.0534.

#### 4.6.7 Methyl 2-((2-Oxo-2-(propylamino)­ethyl)­thio)­benzoate (**3f**)

Isolated yield 87%. ^1^H NMR (CDCl_3_): δ 8.03 (d, *J* = 7.6 Hz, 1H), 7.49 (t, *J* = 7.7 Hz, 1H), 7.24 (t, *J* = 7.1 Hz, 2H), 6.92 (s, 1H), 3.94 (d, *J* = 10.8 Hz, 3H), 3.68 (s, 2H), 3.19 (dd, *J* = 13.4, 6.7 Hz, 2H), 1.42 (dd, *J* = 14.5, 7.3 Hz, 2H), 0.76 (t, *J* = 7.4 Hz, 3H). ^13^C NMR (CDCl_3_): δ 167.6, 166.8, 139.4, 133.1, 131.5, 127.3, 125.4, 124.9, 52.3, 41.4, 36.3, 22.5, 11.1. HRMS calculated for C_13_H_18_NO_3_S [M + H]^+^ 268.1007; found 268.1015.

#### 4.6.8 2-((2-(Methoxycarbonyl)­phenyl)­thio)­acetic Acid (**3g**)

Isolated yield 94%. ^1^H NMR (CD_3_OD): δ 7.93 (dd, *J* = 7.8, 1.0 Hz, 1H), 7.48 (dt, *J* = 17.2, 7.9 Hz, 2H), 7.23 (t, *J* = 7.4 Hz, 1H), 3.89 (s, 3H), 3.78 (s, 2H). ^13^C NMR (CD_3_OD): δ 170.1, 165.3, 138.7, 130.8, 129.2, 126.2, 124.4, 122.7, 49.6, 46.7, 46.5, 46.2, 46.0, 45.8, 45.6, 45.4, 32.6. HRMS calculated for C_10_H_9_O_4_S [M-H]^+^ 225.0222; found 225.0238.

#### 4.6.9 General Synthesis of Compound **4**(**a**–**j**)

To a solution of compound **3** (1 equiv) in DCM, meta-chloroperoxybenzoic acid (0.9 equiv) was added, and the solution was stirred at room temperature for 4 h. The resulting reaction mixture was washed with saturated NaHCO_3_ and brine three times. The organic layer was dried over anhydrous Na_2_SO_4_ and concentrated *in vacuo*. The crude product was purified by column chromatography (silica gel, hexane/ethyl acetate) to afford the desired product.

#### 4.6.10 Methyl 2-((Cyanomethyl)­sulfinyl)­benzoate (**4a**)

Isolated yield 62%. ^1^H NMR (CDCl_3_) δ 8.32 (d, *J* = 7.9 Hz, 1H), 8.11 (d, *J* = 7.7 Hz, 1H), 7.89 (t, *J* = 7.7 Hz, 1H), 7.66 (t, *J* = 7.6 Hz, 1H), 4.06 (dd, *J* = 38.6, 16.3 Hz, 2H), 3.94 (s, 3H). ^13^C NMR (CDCl_3_) δ 166.1, 145.4, 134.3, 131.7, 131.0, 126.7, 125.9, 111.7, 53.1, 43.7. HRMS calculated for C_10_H_10_NO_3_S [M + H]^+^ 224.0381; found 224.0379.

#### 4.6.11 Methyl 2-((2,2,2-Trifluoroethyl)­sulfiny)­benzoate (**4b**)

Isolated yield 85%. ^1^H NMR (CDCl_3_) δ 8.37 (d, *J* = 8.0 Hz, 1H), 8.16 (d, *J* = 7.7 Hz, 1H), 7.90 (dd, *J* = 11.2, 4.2 Hz, 1H), 7.67 (t, *J* = 7.6 Hz, 1H), 4.00 (s, 3H), 3.86 (dq, *J* = 13.8, 10.0 Hz, 1H), 3.31 (dq, *J* = 13.9, 9.9 Hz, 1H). ^13^C NMR (CDCl_3_) δ 165.8, 146.7, 134.3, 131.2, 131.1, 126.6, 125.2, 125.0,123.9, 60.5, 53.0. HRMS calculated for C_10_H_10_F_3_O_3_S [M + H]^+^ 267.0303; found 267.0314.

#### 4.6.12 Methyl 2-((3,3-Dimethyl-2-oxobutyl)­sulfinyl)­benzoate (**4c**)

Isolated yield 74%. ^1^H NMR (CDCl_3_) δ 8.30 (d, *J* = 7.8 Hz, 1H), 8.14–7.99 (m, 1H), 7.82 (dd, *J* = 11.1, 4.2 Hz, 1H), 7.58 (dd, *J* = 11.0, 4.1 Hz, 1H), 4.25 (d, *J* = 15.3 Hz, 1H), 3.91 (s, 3H), 3.76 (d, *J* = 15.3 Hz, 1H), 1.16 (s, 9H). ^13^C NMR (CDCl_3_) δ 207.0, 165.8, 148.4, 133.9, 130.8, 130.4, 126.6, 125.2, 65.2, 52.9, 44.5, 25.9. HRMS calculated for C_14_H_19_O_4_S [M + H]^+^ 283.1004; found 283.1093.

#### 4.6.13 Methyl 2-((2-Methoxy-2-oxoethyl)­sulfinyl)­benzoate (**4d**)

Isolated yield 76%. ^1^H NMR (CDCl_3_) δ 8.11 (dd, *J* = 7.6, 0.9 Hz, 1H), 7.76–7.63 (m, 3H), 4.63 (s, 2H), 3.95 (s, 3H), 3.69 (s, 3H). ^13^C NMR (CDCl_3_) δ 166.0, 147.5, 134.1, 130.9, 130.9, 126.8, 125.2, 60.7, 53.0, 52.8. HRMS calculated for C_11_H_13_O_5_S [M + H]^+^ 257.0484; found 257.0462.

#### 4.6.14 Methyl 2-((2-Amino-2-oxoethyl)­sulfinyl)­benzoate (**4e**)

Isolated yield 66%. ^1^H NMR (DMSO-*d*_6_) δ 8.11 (dd, *J* = 15.0, 7.8 Hz, 2H), 7.95 (t, *J* = 7.6 Hz, 1H), 7.71 (t, *J* = 7.5 Hz, 1H), 7.53 (s, 1H), 7.34 (s, 1H), 3.94 (d, *J* = 13.8 Hz, 1H), 3.88 (s, 3H), 3.34 (d, *J* = 13.8 Hz, 1H). ^13^C NMR (DMSO-*d*_6_) δ 166.9, 165.5, 148.3, 134.5, 131.2, 126.8, 124.9, 62.8, 53.2. HRMS calculated for HRMS calculated for C_10_H_11_NO_4_SNa [M + Na]^+^ 264.0306; found 264.0297.

#### 4.6.15 Methyl 2-((2-Oxo-2-(propylamino)­ethyl)­sulfinyl)­benzoate (**4f**)

Isolated yield 72%. ^1^H NMR (CDCl_3_) δ 8.12 (dd, *J* = 15.2, 7.8 Hz, 2H), 7.78 (t, *J* = 7.5 Hz, 1H), 7.58 (t, *J* = 7.5 Hz, 1H), 6.99 (s, 1H), 3.93 (s, 3H), 3.87 (d, *J* = 13.9 Hz, 1H), 3.63 (d, *J* = 13.9 Hz, 1H), 3.14 (dd, *J* = 13.2, 6.7 Hz, 2H), 1.51 (dt, *J* = 14.5, 7.3 Hz, 2H), 0.91 (t, *J* = 7.4 Hz, 3H). ^13^C NMR (CDCl_3_) δ 165.7, 163.9, 145.7, 133.7, 131.2, 130.9, 126.9, 125.1, 57.6, 52.8, 41.4, 22.7, 11.5. HRMS calculated for C_13_H_18_NO_4_S [M + H]^+^ 284.0957; found 284.0959.

#### 4.6.16 2-((2-(Methoxycarbonyl)­phenyl)­sulfinyl)­acetic Acid (**4g**)

Isolated yield 81%. ^1^H NMR (CD_3_OD) δ 8.21 (dd, *J* = 15.2, 7.8 Hz, 2H), 7.94 (t, *J* = 7.6 Hz, 1H), 7.72 (t, *J* = 7.6 Hz, 1H), 4.21 (d, *J* = 14.6 Hz, 1H), 3.98 (d, *J* = 5.4 Hz, 3H), 3.55 (d, *J* = 14.6 Hz, 1H). ^13^C NMR (CD_3_OD) δ 169.3, 165.8, 146.2, 133.9, 130.8, 126.9, 124.4, 61.9, 52.0. HRMS calculated for C_10_H_9_O_5_S [M-H]^+^ 241.0171; found 241.0132.

#### 4.6.17 General Synthesis Scheme for Compounds **5**(**a**–**j**) and **6**(**a**–**j**)

To a solution of compound **3** (1 equiv) in MeOH/H_2_O (1:1), triethylamine (2 equiv) was added; the resulting solution was stirred at room temperature overnight. The resulting reaction mixture was extracted with ethyl acetate followed by washing with 1 N HCl and brine. The organic layer was dried over anhydrous Na_2_SO_4_ and concentrated. The crude product was purified by column chromatography (silica gel, DCM/methanol) to afford the desired product with almost quantitative yield.

#### 4.6.18 3-Hydroxybenzo­[*b*]­thiophene-2-carbonitrile (**5a**)

Isolated yield 90%.^1^H NMR (CD_3_OD) δ 7.91 (d, *J* = 8.1 Hz, 1H), 7.78 (d, *J* = 8.2 Hz, 1H), 7.54 (t, *J* = 7.6 Hz, 1H), 7.45 (t, *J* = 7.6 Hz, 1H). ^13^C NMR (CD_3_OD) δ 158.8, 138.6, 130.3, 128.4, 124.6, 122.6, 122.1, 113.5, 82.3. HRMS calculated for C_9_H_6_NOS [M + H]^+^ 176.0170; found 176.0178.

#### 4.6.19 1-(3-Hydroxybenzo­[*b*]­thiophen-2-yl)-2,2-dimethylpropan-1-one (**5c**)

Isolated yield 93%. ^1^H NMR (CDCl_3_) δ 13.50 (s, 1H), 8.00 (d, *J* = 8.1 Hz, 1H), 7.71 (d, *J* = 8.2 Hz, 1H), 7.60–7.44 (m, 1H), 7.40 (t, *J* = 7.3 Hz, 1H), 1.43 (s, 9H). ^13^C NMR (CDCl_3_) δ 205.8, 165.0, 139.4, 130.1, 124.6, 123.7, 122.7, 107.6, 43.8, 27.3. HRMS calculated for C_13_H_15_O_2_S [M + H]^+^ 235.0793; found 235.0788.

#### 4.6.20 Methyl 3-Hydroxybenzo­[*b*]­thiophene-2-carboxylate (**5d**)

Isolated yield, quantitative. ^1^H NMR (CDCl_3_) δ 10.06 (s, 1H), 7.87 (d, *J* = 8.0 Hz, 1H), 7.66 (d, *J* = 8.1 Hz, 1H), 7.43 (t, *J* = 7.6 Hz, 1H), 7.33 (t, *J* = 7.5 Hz, 1H), 3.88 (s, 3H). ^13^C NMR (CDCl_3_) δ 167.7, 159.6, 138.8, 130.4, 128.9, 124.4, 123.2, 123.0, 101.6, 52.2. HRMS calculated for C_10_H_9_O_3_S [M + H]^+^ 209.0272; found 209.0271. Data match with the literature reports.^113^

#### 4.6.21 3-Hydroxybenzo­[*b*]­thiophene-2-carboxamide (**5e**)

Isolated yield, quantitative. ^1^H NMR (acetone-*d*_6_) δ 12.56 (s, 1H), 7.91 (dd, *J* = 12.3, 8.1 Hz, 2H), 7.56 (t, *J* = 7.2 Hz, 1H), 7.48 (t, *J* = 7.5 Hz, 1H), 7.30 (s, 2H). 13C NMR (acetone-*d*_6_) δ 168.9, 158.9, 137.1, 131.0, 128.5, 124.6, 123.3, 122.3, 102.9. HRMS calculated for C_9_H_6_NO_2_S [M-H]^+^ 192.0119; found 192.0114.

#### 4.6.22 3-Hydroxy-*N*-propylbenzo­[*b*]­thiophene-2-carboxamide (**5f**)

Isolated yield 91%. ^1^H NMR (CDCl_3_) δ 11.87 (s, 1H), 7.96 (d, *J* = 7.9 Hz, 1H), 7.74 (d, *J* = 8.1 Hz, 1H), 7.51 (t, *J* = 7.5 Hz, 1H), 7.44 (t, *J* = 7.5 Hz, 1H), 5.57 (s, 1H), 3.45 (dd, *J* = 13.6, 6.6 Hz, 2H), 1.69 (dd, *J* = 14.6, 7.3 Hz, 2H), 1.03 (t, *J* = 7.4 Hz, 4H). ^13^C NMR (CDCl_3_) δ 166.9, 158.8, 136.0, 131.3, 128.4, 124.6, 123.0, 122.9, 102.2, 41.3, 22.9, 11.5. HRMS calculated for C_12_H_14_NO_2_S [M + H]^+^ 236.0745; found 236.0751.

#### 4.6.23 3-Hydroxycinnamonitrile 1-Oxide (**6a**)

Isolated yield 92%. ^1^H NMR (CD_3_OD) δ 7.82–7.78 (m, 1H), 7.72 (dd, *J* = 5.7, 2.7 Hz, 1H), 7.70–7.63 (m, 2H). ^13^C NMR (CD_3_OD) δ 181.5, 145.2, 135.6, 131.8, 125.4, 122.7, 118.8, 79.8, 53.4. HRMS calculated for C_9_H_6_NO_2_S [M + H]^+^ 192.0119; found 192.0121.

#### 4.6.24 1-(3-Hydroxy-1-oxidobenzo­[*b*]­thiophen-2-yl)-2,2-dimethylpropan-1-one (**6c**)

Isolated yield quantitative. ^1^H NMR (CDCl_3_) δ 16.35 (s, 1H), 7.99 (dd, *J* = 17.0, 7.6 Hz, 2H), 7.84 (dd, *J* = 10.9, 4.1 Hz, 1H), 7.73 (t, *J* = 7.5 Hz, 1H), 1.56 (s, 9H). ^13^C NMR (CDCl_3_) δ 199.1, 189.8, 148.0, 135.5, 132.6, 132.2, 127.0, 125.1, 41.4, 27.8. HRMS calculated C_13_H_15_O_3_S [M + H]^+^ 251.0742; found 251.0739.

#### 4.6.25 Methyl 3-Hydroxycinnamate 1-Oxide (**6d**)

Isolated yield 88%. ^1^H NMR (CDCl_3_) δ 8.11 (dd, *J* = 7.6, 0.9 Hz, 1H), 7.76–7.63 (m, 3H), 4.63 (s, 2H), 3.95 (s, 3H), 3.69 (s, 3H). ^13^C NMR (CDCl_3_) δ 172.4, 168.2, 145.7, 133.8, 132.4, 131.1, 127.0, 124.0, 109.7, 52.9. HRMS calculated for C_10_H_9_O_4_S [M + H]^+^ 225.0222; found 225.0208.

#### 4.6.26 3-Hydroxycinnamamide 1-Oxide (**6e**)

Isolated yield, quantitative.^1^H NMR (CD_3_OD) δ 8.07–7.87 (m, 2H), 7.62 (ddd, *J* = 8.0, 2.0, 1.0 Hz, 1H), 7.48 (t, *J* = 7.9 Hz, 1H). ^13^C NMR (101 MHz, CD_3_OD) δ 166.9, 134.1, 132.7, 132.5, 129.8, 129.2, 127.6. HRMS calculated for C_9_H_7_NO_3_SNa [M + Na]^+^ 232.0044; found 232.0052.

#### 4.6.27 3-Hydroxy-*N*-propylcinnamamide 1-Oxide (**6f**)

Isolated yield 87%. ^1^H NMR (CD_3_OD) δ 7.88–7.80 (m, 1H), 7.79–7.72 (m, 1H), 7.71–7.58 (m, 2H), 3.38 (t, *J* = 7.0 Hz, 2H), 1.71–1.55 (m, 2H), 1.05–0.94 (m, 3H). ^13^C NMR (CDCl_3_) δ 177.9, 167.5, 143.9, 137.6, 130.0, 131.4, 125.3, 123.0, 40.5, 22.7, 10.3. HRMS calculated for C_12_H_14_NO_3_S [M + H]^+^ 252.0616; found 252.0692.

#### 4.6.28 General Synthesis Scheme for Compound **8**

To a solution of compound **7** (1 equiv) in DCM/ACN (1:1), compound **2** (1 equiv) was added, and the resulting mixture was kept in an ice bath. Then, triethylamine (1 equiv) was added, and the resulting solution was stirred overnight and allowed to reach room temperature slowly. The resulting reaction mixture was concentrated under a vacuum. The product was extracted with DCM followed by washing with 1 N HCl and brine. The organic layer was dried over anhydrous Na_2_SO_4_ and concentrated. The crude product was taken to the next step without further purification.

#### 4.6.29 1-((2*R*,4*S*,5*R*)-5-(((*tert*-Butyldimethylsilyl)­oxy)­methyl)-4-hydroxytetrahydrofuran-2-yl)-5-fluoropyrimidine-2,4­(1*H*,3*H*)-dione (**10**)

The compound was synthesized by following the previously reported method.^114^

#### 4.6.30 (2*S*,3*R*,5*S*)-2-(((*tert*-Butyldimethylsilyl)­oxy)­methyl)-5-(5-fluoro-2,4-dioxo-3,4-dihydropyrimidin-1­(2*H*)-yl)­tetrahydrofuran-3-yl 2-((Cyanomethyl)­thio)­benzoate (**11a**)

Isolated yield 78%. ^1^H NMR (CDCl_3_) δ 8.95 (d, *J* = 4.5 Hz, 1H), 8.17–8.03 (m, 2H), 7.66 (t, *J* = 7.7 Hz, 1H), 7.46 (d, *J* = 8.0 Hz, 1H), 7.40 (t, *J* = 7.6 Hz, 1H), 6.55–6.44 (m, 1H), 5.54 (d, *J* = 5.7 Hz, 1H), 4.35 (s, 1H), 4.03 (q, *J* = 11.3 Hz, 2H), 3.77 (s, 2H), 2.68 (dd, *J* = 14.0, 5.5 Hz, 1H), 2.37–2.21 (m, 1H), 1.05–0.90 (m, 9H), 0.20 (s, 6H). ^13^C NMR (CDCl_3_) δ 165.6, 156.6 (d, *J* = 27.1 Hz), 148.8, 141.9, 139.5, 137.5, 133.8, 132.0, 127.5, 126.6, 126.2, 123.9, 116.0, 85.7, 63.9, 38.4, 18.4, −5.5. HRMS calculated for C_24_H_29_FN_3_O_6_SSi [M – H]^−^ = 534.1530, found 534.1527.

#### 4.6.31 ((2*R*,3*S*,5*R*)-3-((*tert*-Butyldimethylsilyl)­oxy)-5-(5-fluoro-2,4-dioxo-3,4-dihydropyrimidin-1­(2*H*)-yl)­tetrahydrofuran-2-yl)­methyl 2-((2-Methoxy-2-oxoethyl)­thio)­benzoate (**11d**)

Isolated yield 72%. ^1^H NMR (CDCl_3_) δ 9.81 (d, *J* = 4.5 Hz, 1H), 8.01 (d, *J* = 6.1 Hz, 1H), 7.95 (d, *J* = 7.7 Hz, 1H), 7.42 (t, *J* = 7.7 Hz, 1H), 7.29 (d, *J* = 8.1 Hz, 1H), 7.17 (dd, *J* = 14.2, 6.6 Hz, 1H), 6.46–6.37 (m, 1H), 5.41 (d, *J* = 5.7 Hz, 1H), 4.24 (s, 1H), 3.92 (dd, *J* = 25.5, 10.6 Hz, 2H), 3.66 (d, *J* = 6.6 Hz, 5H), 2.57 (dd, *J* = 14.0, 5.5 Hz, 1H), 2.22–2.10 (m, 1H), 0.85 (s, 9H), 0.08 (s, 6H). ^13^C NMR (CDCl_3_) δ 169.8, 165.7, 157.0 (d, *J* = 26.7 Hz), 149.2, 142.0, 140.7, 139.6, 133.4, 131.8, 126.5, 125.8, 124.9, 124.2, 123.8, 85.8 (d, *J* = 28.2 Hz), 76.7, 63.9, 52.8, 38.4, 34.6, 25.9, 18.3, −5.4, −5.5. HRMS calculated for C_25_H_34_FN_2_O_8_SSi [M + H]^+^ 569.1789; found 569.1813.

#### 4.6.32 ((2*R*,3*S*,5*R*)-3-((*tert*-Butyldimethylsilyl)­oxy)-5-(5-fluoro-2,4-dioxo-3,4-dihydropyrimidin-1­(2*H*)-yl)­tetrahydrofuran-2-yl)­methyl 2-((2-Amino-2-oxoethyl)­thio)­benzoate (**11e**)

Isolated yield 76%. ^1^H NMR (CDCl_3_) δ 10.73 (d, *J* = 4.0 Hz, 1H), 8.05 (t, *J* = 6.9 Hz, 2H), 7.51 (t, *J* = 7.5 Hz, 1H), 7.36–7.14 (m, 2H), 6.85 (s, 1H), 6.71 (s, 1H), 6.58–6.37 (m, 1H), 5.50 (d, *J* = 5.5 Hz, 1H), 4.33 (s, 1H), 4.03 (dt, *J* = 20.1, 9.0 Hz, 2H), 3.69 (s, 2H), 2.64 (dd, *J* = 13.8, 5.2 Hz, 1H), 2.40–2.16 (m, 1H), 0.92 (d, *J* = 15.2 Hz, 9H), 0.16 (s, 6H). ^13^C NMR (CDCl_3_) δ 171.7,171.3, 165.8, 157.4, 149.4, 142.0, 139.7, 133.8, 132.0, 126.3, 125.3,123.84, 85.7, 63.9, 60.5, 38.4, 35.8, 25.9, 18.3, −5.5. HRMS calculated for C_24_H_32_FN_3_O_7_SSiNa [M + Na]^+^ 576.6672; found 576.6675.

#### 4.6.33 (2*R*,3*S*,5*R*)-2-(((*tert*-Butyldimethylsilyl)­oxy)­methyl)-5-(5-fluoro-2,4-dioxo-3,4-dihydropyrimidin-1­(2*H*)-yl)­tetrahydrofuran-3-yl 2-((2-Oxo-2-(propylamino)­ethyl)­thio)­benzoate (**11f**)

Isolated yield 72%.^1^H NMR (CDCl_3_) δ 9.60 (d, *J* = 4.3 Hz, 1H), 8.10 (dd, *J* = 9.8, 7.1 Hz, 2H), 7.54 (t, *J* = 7.7 Hz, 1H), 7.28 (t, *J* = 8.7 Hz, 2H), 6.87 (t, *J* = 5.5 Hz, 1H), 6.59–6.42 (m, 1H), 5.53 (d, *J* = 5.6 Hz, 1H), 4.35 (s, 1H), 4.03 (q, *J* = 11.3 Hz, 2H), 3.70 (s, 2H), 3.21 dd, *J* = 13.3, 6.7 Hz, 2H), 2.68 (dd, *J* = 14.0, 5.4 Hz, 1H), 2.34–2.20 (m, 1H), 1.42 (dt, *J* = 14.4, 7.2 Hz, 2H), 0.93 (d, *J* = 24.2 Hz, 9H), 0.78 (t, *J* = 7.4 Hz, 3H), 0.19 (s, 6H). ^13^C NMR (CDCl_3_) δ 167.6, 165.8, 156.8 (d, *J* = 26.8 Hz), 149.1, 141.9, 140.0, 139.6, 133.8, 131.9, 126.1, 125.4, 125.1, 123.9, 85.7, 63.9, 41.5, 38.4, 36.1, 29.7, 25.9, 22.5, 18.3, 11.1, −5.5. HRMS calculated for C_27_H_38_FN_3_O_7_SSi [M + H]^+^ 596.2262; found 596.2239.

#### 4.6.34 (2*S*,3*R*,5*S*)-5-(5-Fluoro-2,4-dioxo-3,4-dihydropyrimidin-1­(2*H*)-yl)-2-(hydroxymethyl)­tetrahydrofuran-3-yl 2-((Cyanomethyl)­thio)­benzoate (**12a**)

Isolated yield 95%. ^1^H NMR (CDCl_3_) δ 10.50 (s, 1H), 8.30 (d, *J* = 7.1 Hz, 1H), 8.15 (d, *J* = 7.8 Hz, 1H), 7.70 (dd, *J* = 11.2, 4.2 Hz, 1H), 7.61 (d, *J* = 8.0 Hz, 1H), 7.41 (t, *J* = 7.5 Hz, 1H), 6.43 (t, *J* = 6.5 Hz, 1H), 5.63 (d, *J* = 5.9 Hz, 1H), 4.71 (s, 1H), 4.34 (d, *J* = 1.7 Hz, 1H), 4.15 (d, *J* = 6.4 Hz, 2H), 3.96 (dd, *J* = 26.6, 11.9 Hz, 2H), 2.55 (ddt, *J* = 14.4, 8.5, 7.3 Hz, 2H). ^13^C NMR (acetone-*d*_6_) δ 165.3, 156.8, 149.0, 141.8, 139.5, 138.3, 133.4, 131.8, 127.5, 125.8, 124.2, 116.9, 103.4, 85.4, 76.6, 61.9, 37.6, 17.0 HRMS calculated for C_18_H_14_FN_3_O_6_S [M – H]^+^ 420.0666; found 420.0683.

#### 4.6.35 5-(5-Fluoro-2,4-dioxo-3,4-dihydropyrimidin-1­(2*H*)-yl)-2-(hydroxymethyl)­tetrahydrofuran-3-yl 2-((2-Methoxy-2-oxoethyl)­thio)­benzoate (**12d**)

Isolated yield 96%. ^1^H NMR (acetone-*d*_6_) δ 8.19 (d, *J* = 7.1 Hz, 1H), 7.93 (d, *J* = 7.8 Hz, 1H), 7.45 (t, *J* = 7.7 Hz, 1H), 7.37 (d, *J* = 8.0 Hz, 1H), 7.18 (t, *J* = 7.5 Hz, 1H), 6.32 (t, *J* = 7.1 Hz, 1H), 5.50 (d, *J* = 5.9 Hz, 1H), 4.57 (s, 1H), 4.21 (s, 1H), 3.85 (d, *J* = 16.5 Hz, 2H), 3.76 (s, 2H), 3.57 (s, 3H), 2.50–2.37 (m, 2H). ^13^C NMR (acetone-*d*_6_) δ 170.4, 166.2, 157.5, 149.9, 142.6, 141.4, 140.3, 133.8, 132.2, 128.4, 127.2, 125.5 (d, *J* = 19.5 Hz), 125.0, 86.3 (d, *J* = 21.0 Hz), 77.2, 62.9, 52.7, 38.5, 34.9. HRMS calculated for C_19_H_18_FN_2_O_8_S [M – H]^−^ 453.0768 and found 453.0768.

#### 4.6.36 ((2*R*,3*S*,5*R*)-5-(5-Fluoro-2,4-dioxo-3,4-dihydropyrimidin-1­(2*H*)-yl)-3-hydroxytetrahydrofuran-2-yl)­methyl 2-((2-Amino-2-oxoethyl)­thio)­benzoate (**12e**)

Isolated yield 97%.^1^H NMR (DMSO*-d*_6_) δ 8.10 (d, *J* = 7.0 Hz, 1H), 7.96 (d, *J* = 7.7 Hz, 1H), 7.77 (s, 1H), 7.57 (t, *J* = 7.6 Hz, 1H), 7.47 (d, *J* = 8.1 Hz, 1H), 7.28 (t, *J* = 7.5 Hz, 1H), 7.22 (s, 1H), 6.29 (t, *J* = 6.9 Hz, 1H), 5.77 (s, 1H), 5.47 (s, 2H), 4.16 (s, 1H), 3.77–3.69 (m, 2H), 3.67 (s, 2H), 2.39 (d, *J* = 6.5 Hz, 2H). ^13^C NMR (DMSO*-d*_6_) δ 170.2, 165.5, 152.2, 141.3,141.6, 133.5, 131.5, 126.9, 126.4, 124.8,123.9, 85.1, 76.6, 67.5, 61.9, 55.4, 37.4, 35.9. HRMS calculated for C_18_H_17_FN_3_O_7_S [M – H]^−^ 438.4064 and found 438.4062.

#### 4.6.37 (2*R*,3*S*,5*R*)-5-(5-Fluoro-2,4-dioxo-3,4-dihydropyrimidin-1­(2*H*)-yl)-2-(hydroxymethyl)­tetrahydrofuran-3-yl 2-((2-Oxo-2-(propylamino)­ethyl)­thio)­benzoate (**12f**)

Isolated yield 96%. ^1^H NMR (DMSO-*d*_6_) δ 11.92 (d, *J* = 4.8 Hz, 1H), 8.27 (d, *J* = 7.1 Hz, 1H), 8.19 (s, 1H), 7.97 (d, *J* = 7.7 Hz, 1H), 7.55 (d, *J* = 7.6 Hz, 1H), 7.47 (d, *J* = 8.1 Hz, 1H), 7.28 (t, *J* = 7.5 Hz, 1H), 6.27 (s, 1H), 5.46 (d, *J* = 4.8 Hz, 1H), 4.20 (s, 1H), 3.73 (s, 1H), 3.68 (s, 2H), 3.38 (s, 2H), 3.02 (dd, *J* = 12.8, 6.5 Hz, 2H), 2.42 (d, *J* = 12.5 Hz, 2H), 1.40–1.32 (m, 2H), 0.80 (t, *J* = 7.4 Hz, 3H). ^13^C NMR (DMSO-*d*_6_) δ 167.9, 165.5, 157.5, 149.5, 141.7, 141.5, 139.4, 133.5, 131.6, 126.9, 126.5, 125.2, 124.9, 85.2, 76.3, 61.8, 61.2, 41.1, 29.6, 22.7, 11.8. HRMS calculated for C_21_H_25_FN_3_O_7_S [M + H]^+^ 482.1397; found 482.1388.

#### 4.6.38 (*S*)-4,11-Diethyl-4-hydroxy-3,14-dioxo-3,4,12,14-tetrahydro-1*H*-pyrano­[3′,4’:6,7]­indolizino­[1,2-*b*]­quinolin-9-yl 2-((2-Amino-2-oxoethyl)­thio)­benzoate (**14e**)

Isolated yield 62%. ^1^H NMR (CDCl_3_) δ 8.38 (dd, *J* = 7.8, 1.3 Hz, 1H), 8.30 (d, *J* = 9.2 Hz, 1H), 7.97 (d, *J* = 2.4 Hz, 1H), 7.75–7.58 (m, 3H), 7.39 (dd, *J* = 14.4, 7.5 Hz, 2H), 6.70 (s, 1H), 5.76 (d, *J* = 16.3 Hz, 1H), 5.46 (s, 1H), 5.36–5.25 (m, 3H), 3.78 (s, 1H), 3.71 (s, 2H), 3.19 (q, *J* = 7.6 Hz, 2H), 1.91 (ddt, *J* = 21.5, 14.1, 7.2 Hz, 2H), 1.42 (t, *J* = 7.7 Hz, 3H), 1.05 (t, *J* = 7.4 Hz, 3H). ^13^C NMR (CDCl_3_) δ: 173.9, 170.4, 164.6, 157.7, 152.1, 150.2, 149.6, 147.6, 146.8, 145.4, 141.0, 134.3, 132.4, 132.2, 127.5, 127.4, 125.7, 125.5, 125.2, 118.6, 114.9, 98.1, 72.8, 66.4, 49.4, 35.8, 31.6, 29.7, 23.2, 14.1, 7.8. HRMS calcd for C_31_H_28_N_3_O_7_S [M + H]^+^ 568.1648; found 586.1667.

## Supplementary Material





## Notes

Additional materials and methods and experimental details including HPLC traces and studies, LCMS study, cyclization studies, cytotoxicity studies, and NMR and HRMS spectrum of compounds (PDF)
Molecular formula strings (CSV)

## Abbreviations

CCK-8: Cell Counting Kit-8
CReKT: Cascade Reaction with Kinetic Tunability
flox: floxuridine
hROS: highly reactive oxygen species
*m*-CPBA: meta-chloroperbenzoic acid
MPO: myeloperoxidase
PBS: phosphate-buffered saline
SOD: superoxide dismutase (SOD)
