# Supplementary material for: Targeting Highly Reactive Oxygen Species (hROS) for Prodrug Activation through a Cascade Reaction with Kinetic Tunability (CReKT) to Effect Linker Cleavage
Source: J Med Chem. 2026 Mar 16;69(8):9196–214. doi: 10.1021/acs.jmedchem.5c03627 (PMC13126685; doi:10.1021/acs.jmedchem.5c03627)

Supporting Information

Targeting Highly Reactive Oxygen Species (hROS) for Prodrug Activation through a Cascade Reaction with Kinetic Tunability (CReKT) to Effect Linker Cleavage

Shameer M. Kondengadan,[#] Shubham Bansal,[#] Wen Lu, Manjusha Roy Choudhury, and Binghe Wang*

Department of Chemistry and Center for Diagnostics and Therapeutics, Georgia State University, Atlanta, Georgia 30301 USA

[#]SK and SB contributed equally to this work.

*Address correspondence to
Dr. Binghe Wang
Regents Professor and Dr. Frank Hannah Chair
Georgia Research Alliance Eminent Scholar
Department of Chemistry
Georgia State University
Atlanta, Georgia 30301
USA
wang@gsu.edu
Phone: 404-413-5544
ORCID: 0000-0002-2200-5270

Contents

1 Supporting figures and tables. 2
2 Cyclization kinetics of model compounds 2
2.1 Cyclization kinetics of 3a/4a 2
2.2 Cyclization kinetics of 3c/4c 3
2.3 Cyclization kinetics of 3d/4d 3
2.4 Cyclization kinetics of 3e/4e 4
2.5 Cyclization kinetics of 3f/4f 4
3 Cyclization and drug release studies using HPLC 5
4 Effect of pH on stability of prodrug 12e 6
5 Esterase Stability Studies 8
6 Quantification of floxuridine in RAW cells under inflamed conditions using LCMS 10
6.1 Standard curve 10
6.2 LCMS Chromatograph of floxuridine and internal standard (2'-deoxyuridine) 11
7 Cytotoxicity studies 12
8 HPLC traces of compounds 13
9 NMR spectra 16
10 MS Spectra 49

1 Supporting figures and tables.

Table S1. Optimized QQQ-MRM Parameters

Analyte	Precursor/product	CE(V)	CV (V)
Floxuridine	244.84/154.87	12	36
Internal standard (2'-deoxyuridine)	226.84/183.91	10	40

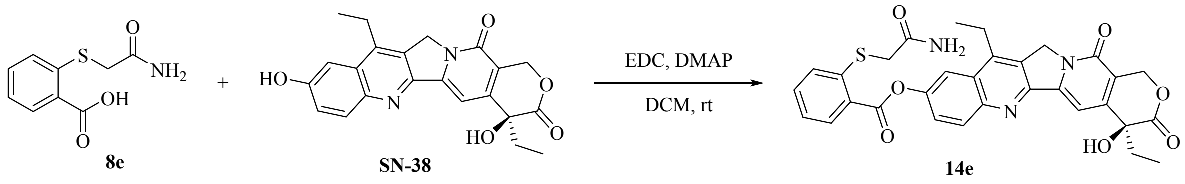

*Scheme S1. Synthesis of SN-38 prodrug **14e***

2 Cyclization kinetics of model compounds

2.1 Cyclization kinetics of 3a/4a

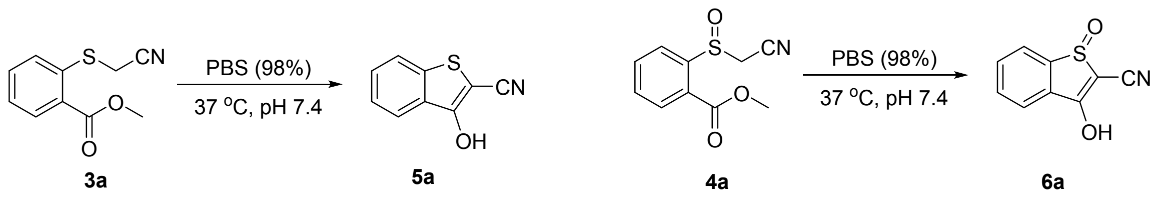

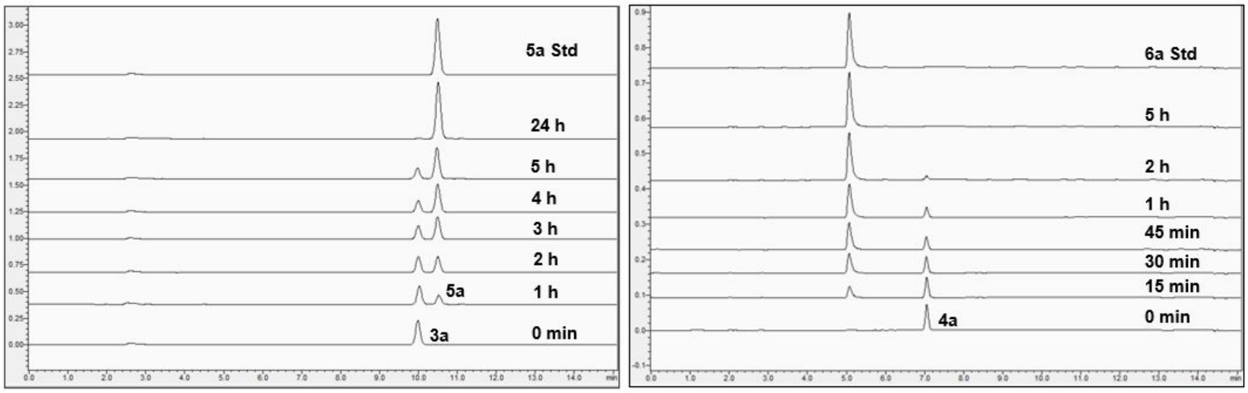

Figure S1. HPLC chromatograms showing the cyclization kinetics of thioether **3a** (a) and sulfoxide **4a** (b) under near physiological conditions (200 µM, DMSO:PBS (2:98)).

2.2 Cyclization kinetics of 3c/4c

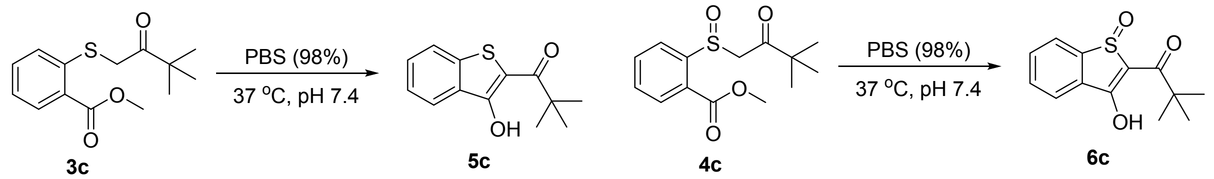

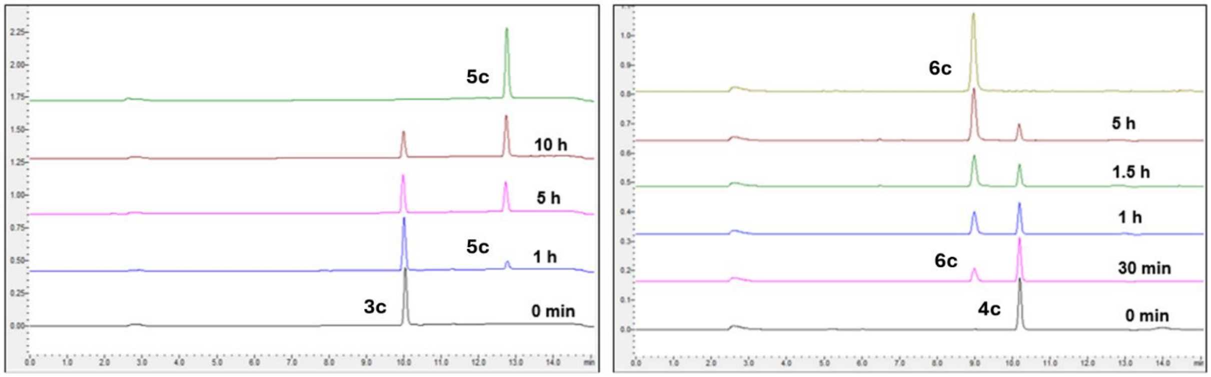

Figure S2. HPLC chromatograms showing the cyclization kinetics of thioether **3c** (a) and sulfoxide **4c** (b) under near physiological conditions (200 μM, DMSO:PBS (2:98)).

2.3 Cyclization kinetics of 3d/4d

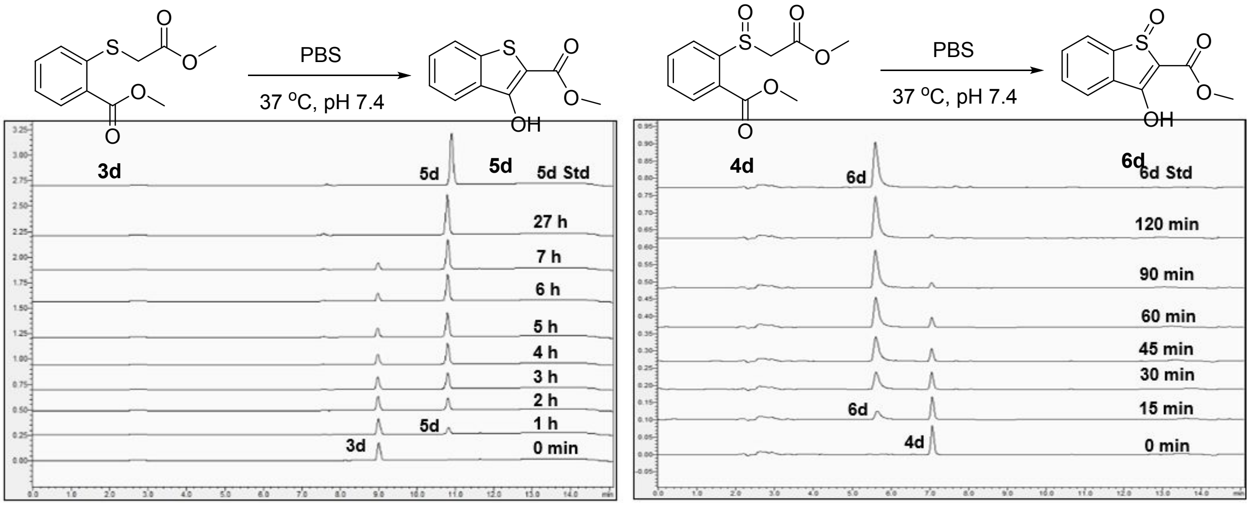

Figure S3. HPLC chromatograms showing the cyclization kinetics of thioether **3d** (a) and sulfoxide **4d** (b) under near physiological conditions (200 μM, DMSO:PBS (2:98)).

2.4 Cyclization kinetics of 3e/4e

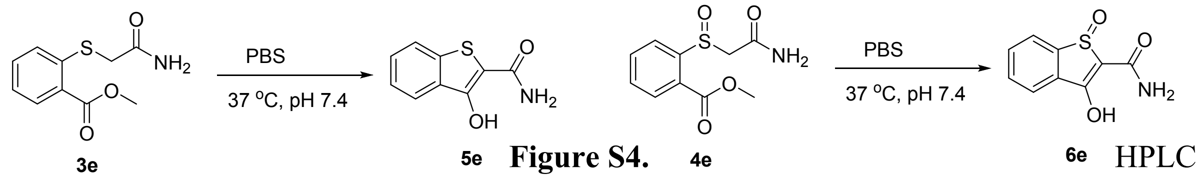

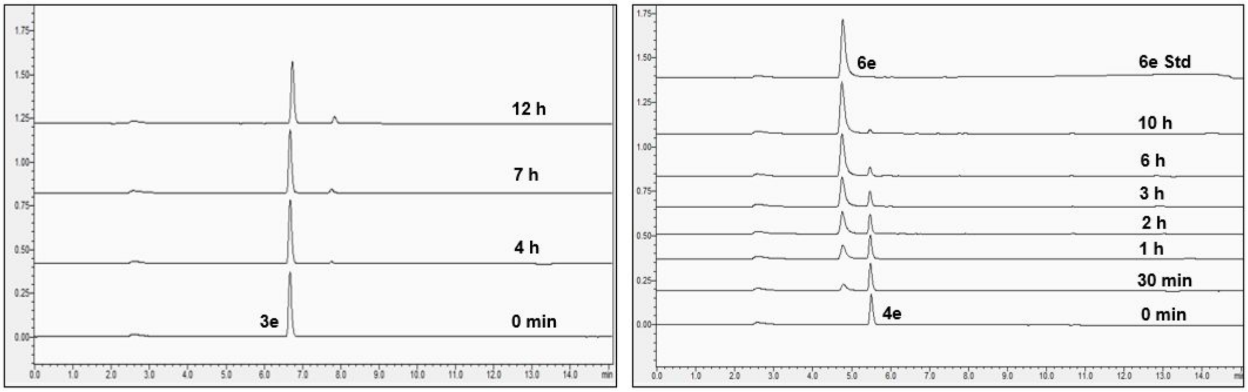

Figure S4. HPLC chromatograms showing the cyclization kinetics of thioether **3e** (a) and sulfoxide **4e** (b) under near physiological conditions (200 μM, DMSO:PBS (2:98)).

2.5 Cyclization kinetics of 3f/4f

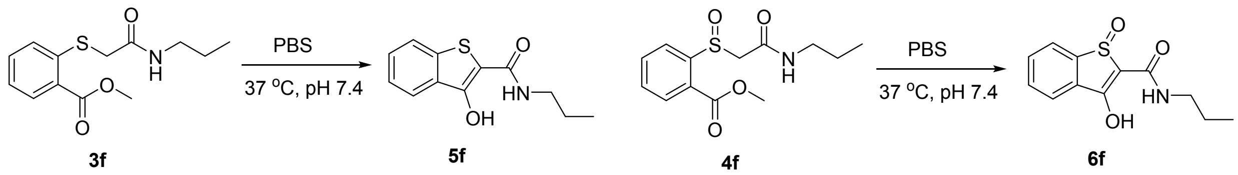

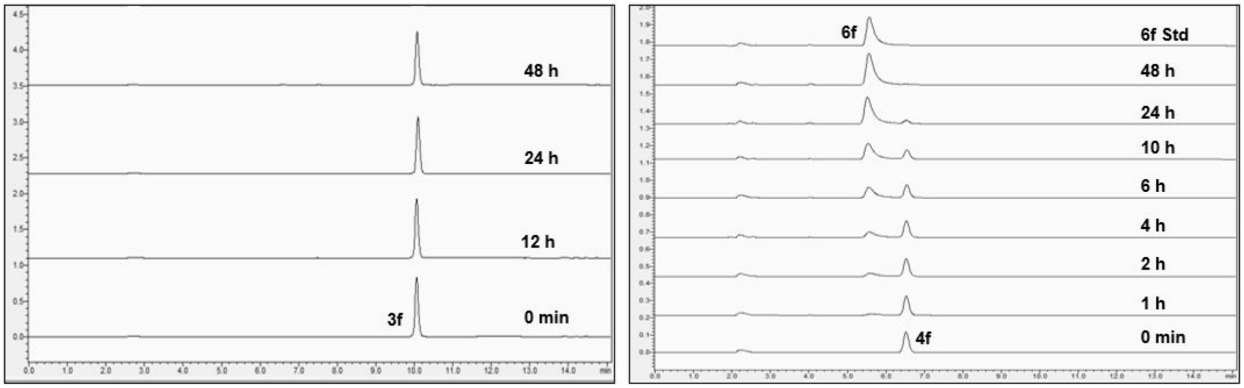

Figure S5: HPLC chromatograms showing the cyclization kinetics of thioether **3f** (a) and sulfoxide **4f** (b) under near physiological conditions (200 µM, DMSO:PBS (2:98)).

3 Cyclization and drug release studies using HPLC

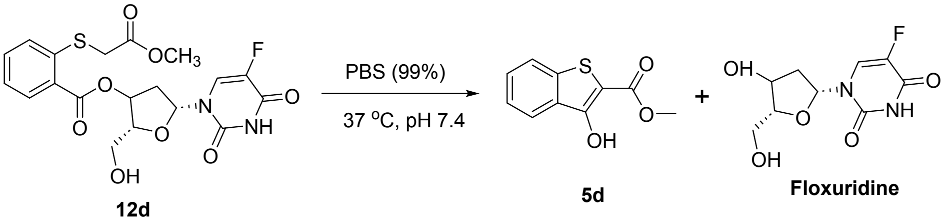

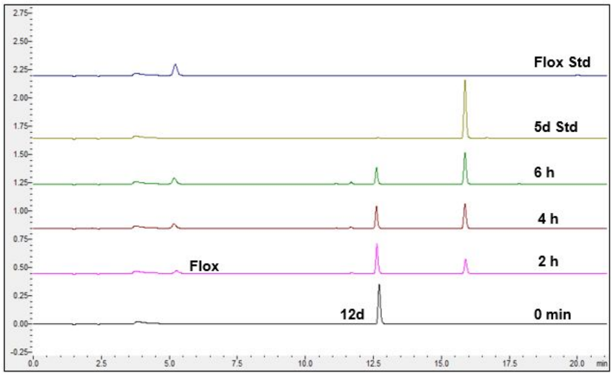

Figure S6: HPLC chromatograms showing the cyclization kinetics of flox-CReKT **12d** (100 µM) in the absence of ROS under near physiological conditions (100 µM, DMF:PBS (1:99)).

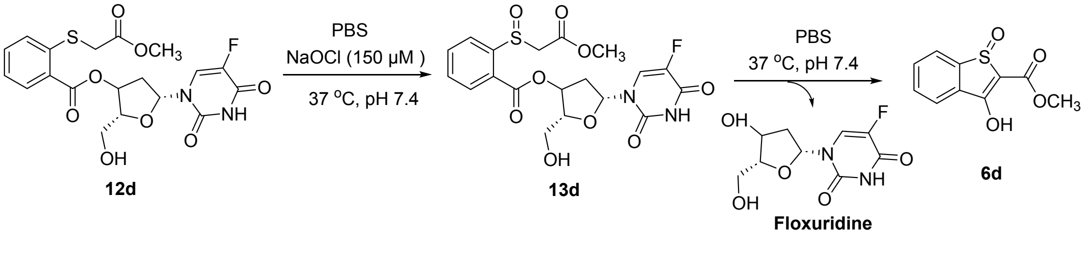

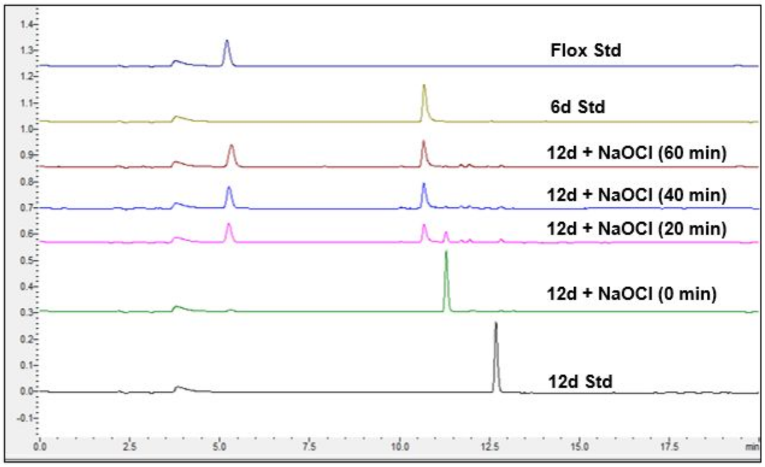

Figure S7: HPLC chromatograms showing the cyclisation kinetics of flox-CReKT **12d (**100 µM**)** in presence of NaOCl (150 µM) under near physiological conditions (DMF:PBS (1:99)).

4 Effect of pH on stability of prodrug 12e

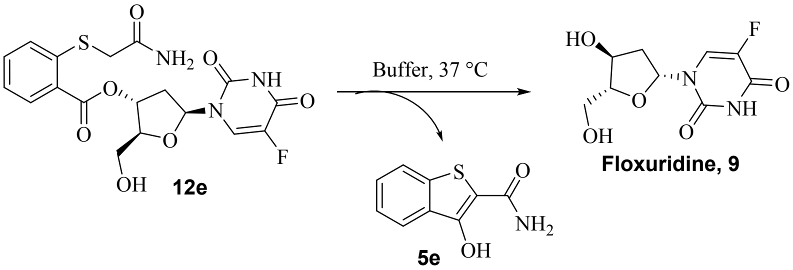

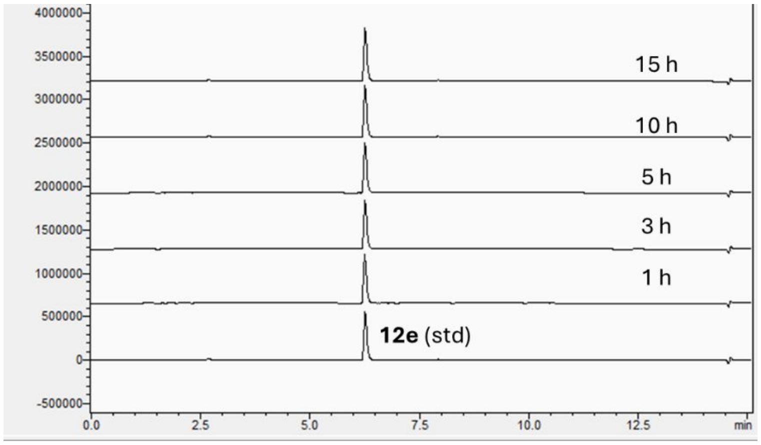

Figure S8: HPLC chromatograms showing the stability of **12d (**100 µM**)** in pH 5.5 and 37 °C.

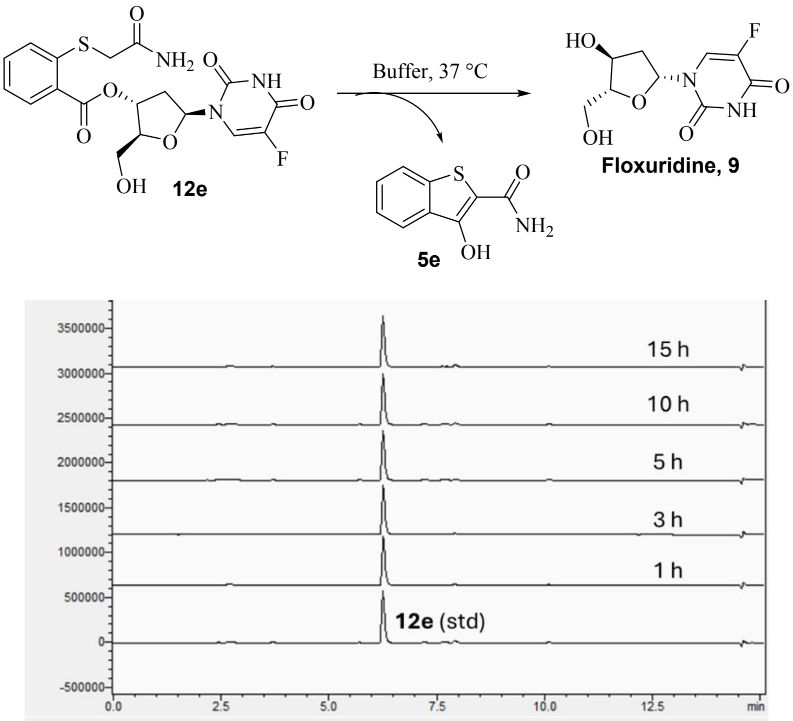

Figure S9: HPLC chromatograms showing the stability of **12d (**100 μM**)** in pH 6.5 and 37 °C.

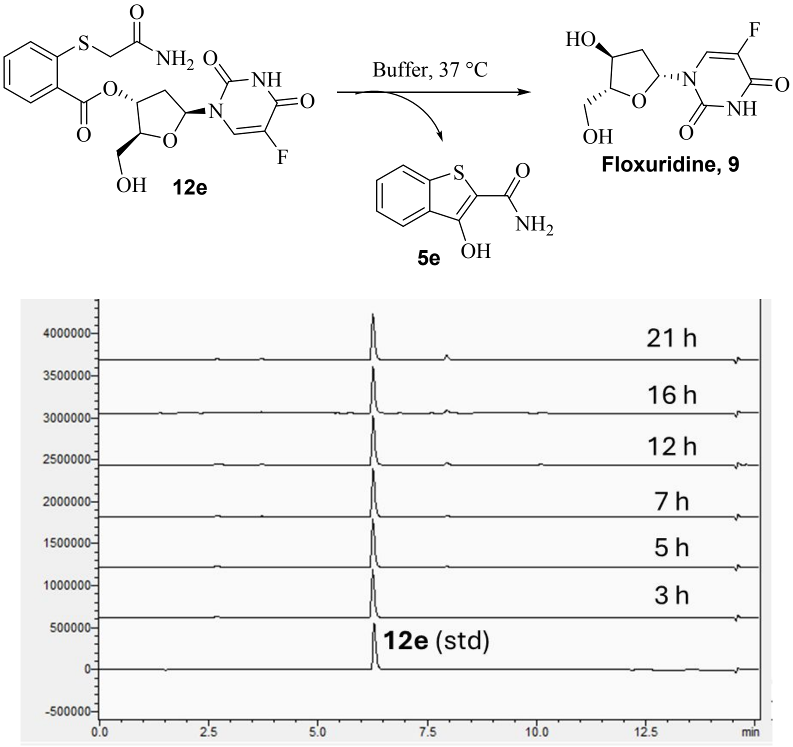

Figure S10: HPLC chromatograms showing the stability of **12d (**100 μM**)** in pH 7.4 and 37 °C.

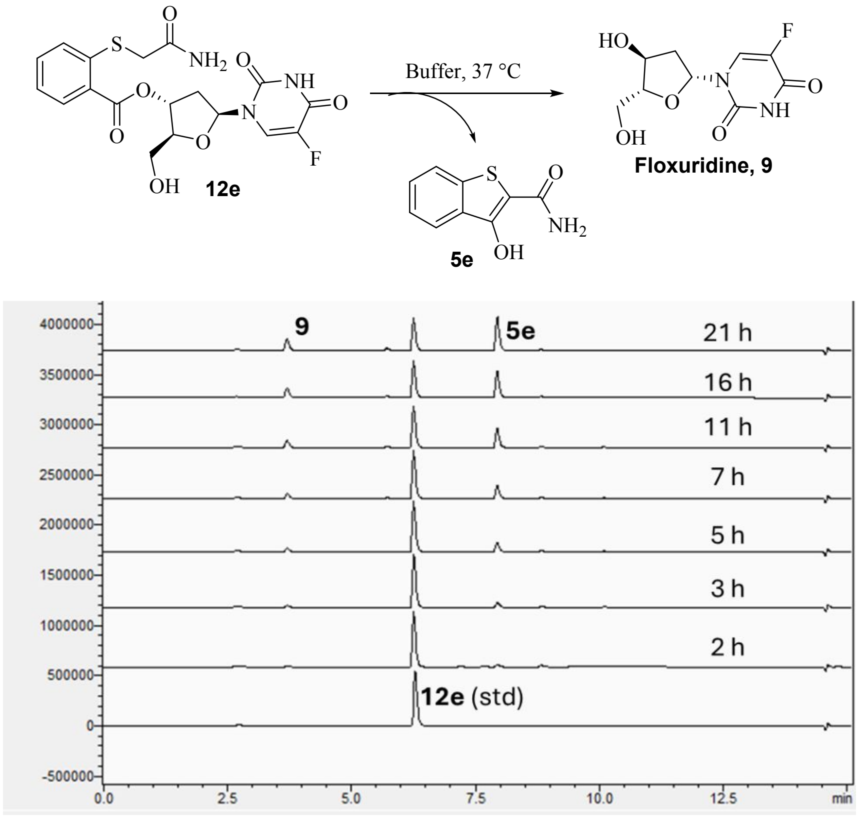

Figure S11: HPLC chromatograms showing the stability of **12d (**100 µM**)** in pH 7.4 and 37 °C.

5 Esterase Stability Studies

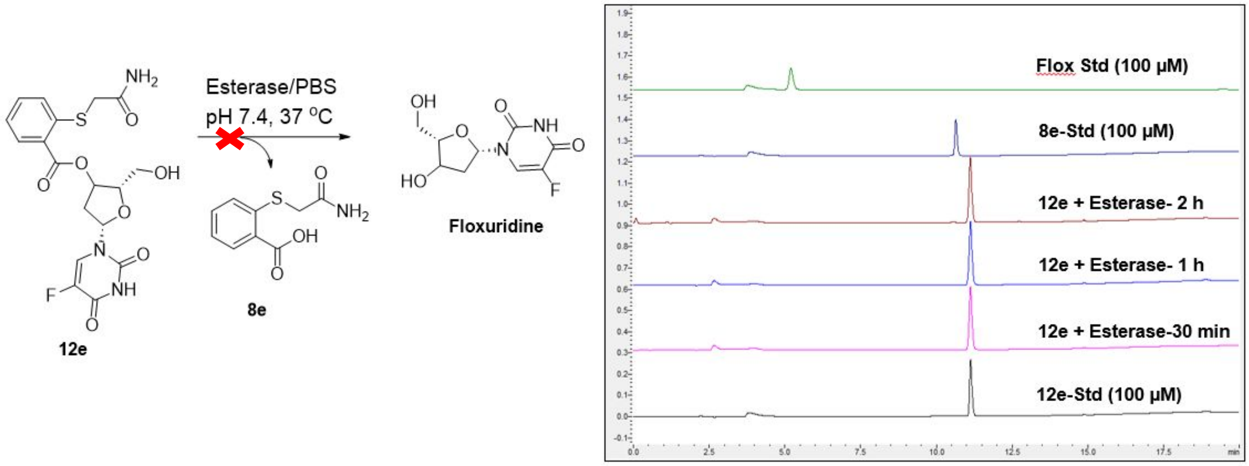

Figure S12. Stability studies of **12e** (100 µM) in the presence of porcine liver esterase (1 U/mL) under near physiological condition (PBS, 37°C, pH 7.4). Compound **3d** was a positive control (Figure S13).

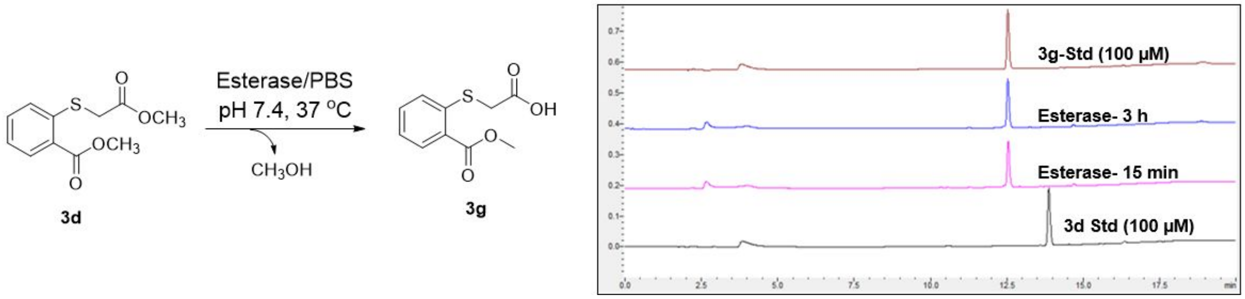

Figure S13: Stability study of **3d** (100 μM) in the presence of porcine liver esterase (1 U/mL) in near physiological condition (PBS, 37°C, pH 7.4).

6 Quantification of floxuridine in RAW cells under inflamed conditions using LCMS

6.1 Standard curve

Table S2. Peak area of floxuridine and internal standard (2'-deoxyuridine) at different concentrations

Conc (µM)	Floxuridine	Internal standard (2'-deoxyuridine)	Ratio
**0.1**	726	42979	0.017
**1**	5097	42241	0.12
**5**	24756	43362	0.57
**10**	49526	40382	1.23
**20**	108154	40458	2.67
**50**	268829	39664	6.78
**100**	579422	43562	13.30

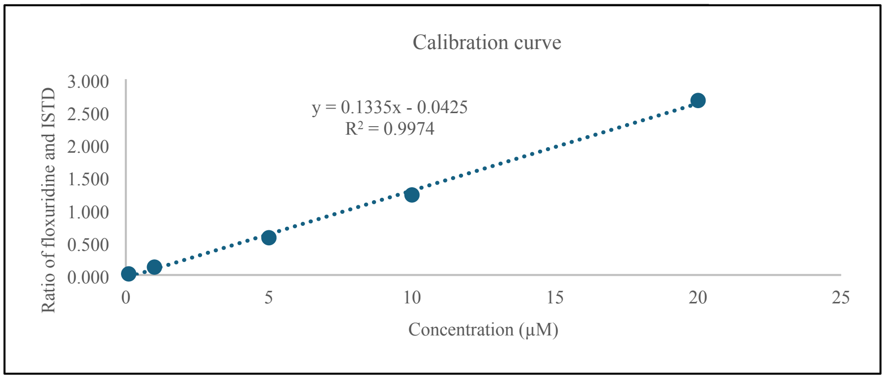

Figure S14. Calibration curve for quantification of floxuridine using LCMS.

6.2 LCMS Chromatograph of floxuridine and internal standard (2'-deoxyuridine)

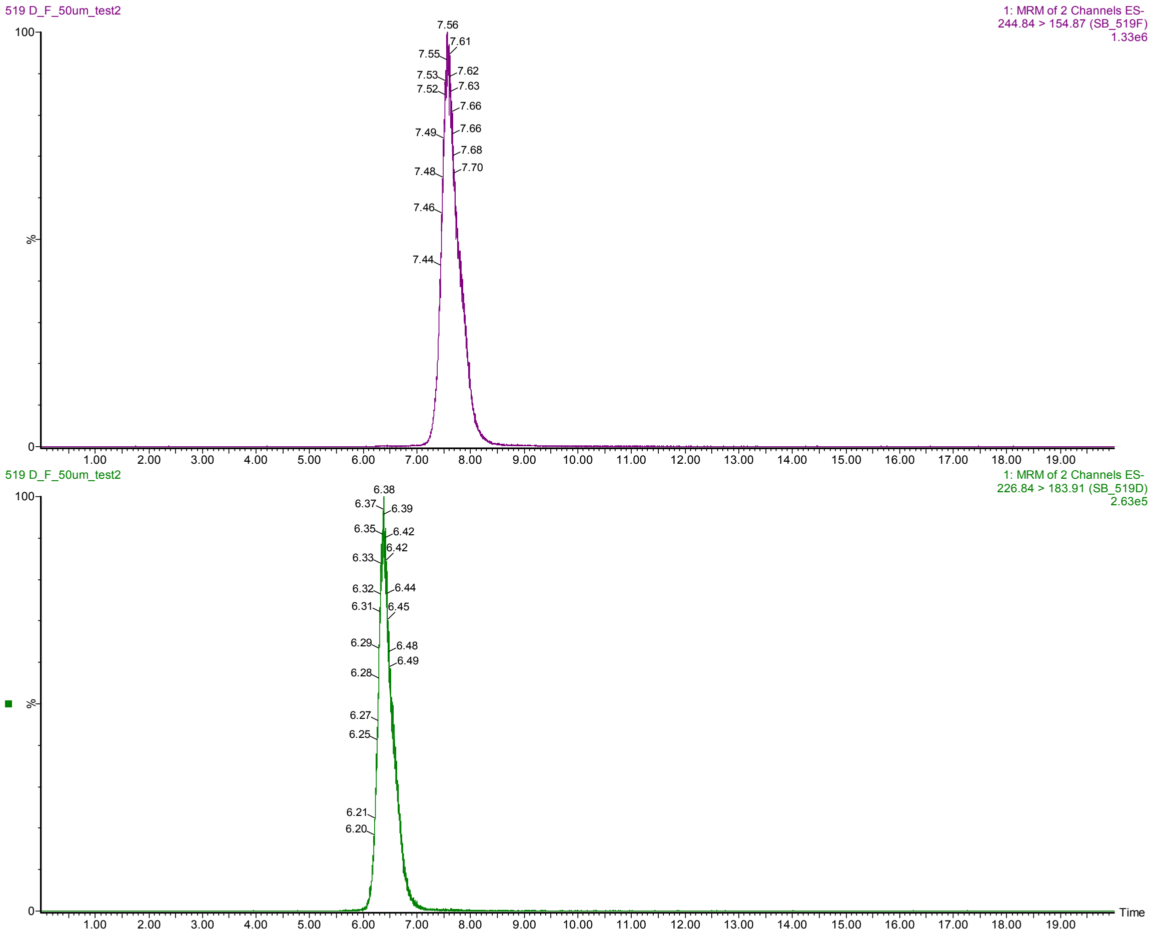

Figure S15. LCMS Chromatograph of floxuridine and internal standard (2'-deoxyuridine).

7 Cytotoxicity studies

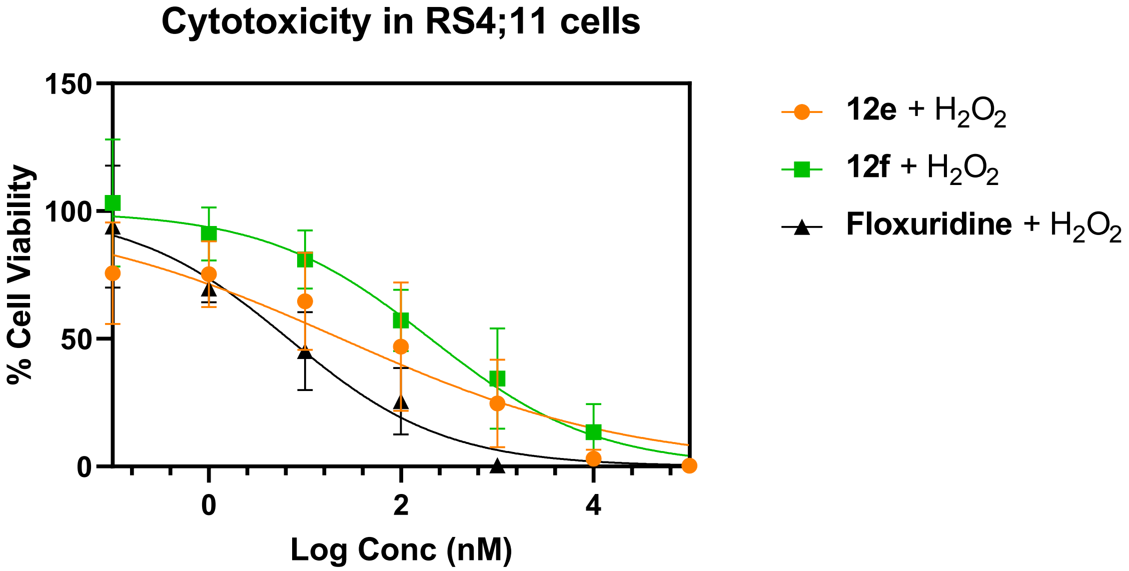

Figure S16. Dose response curves of **12e-f** with H_2O_2.

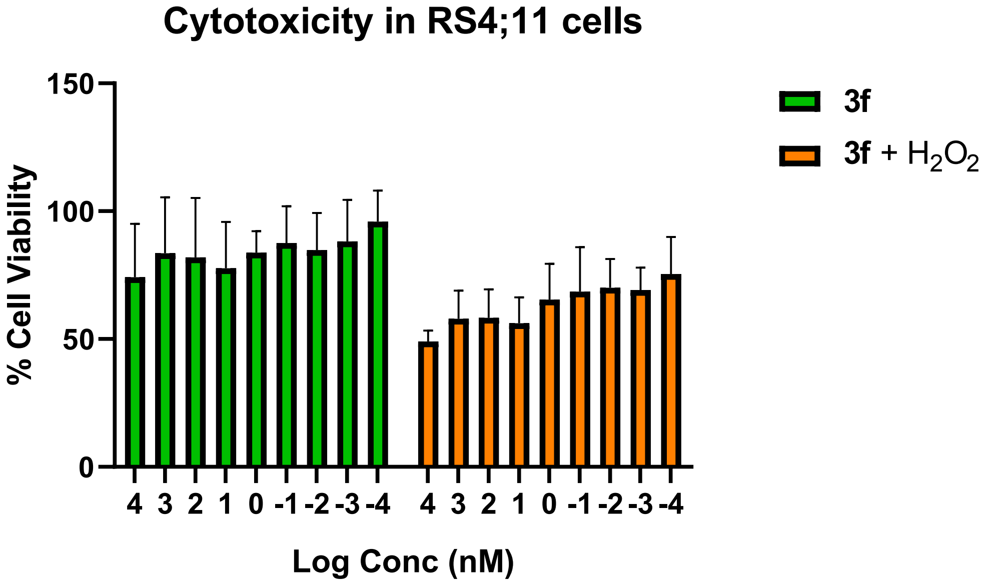

Figure S17. Cytotoxicity studies of **3f** with and without H_2O_2.

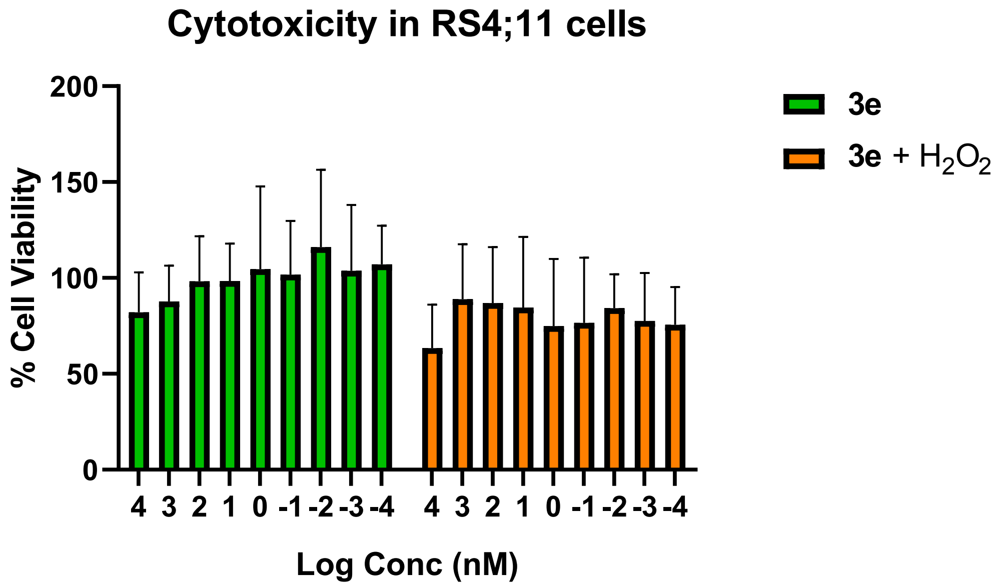

.

Figure S18. Cytotoxicity studies of **3e** with and without H_2O_2.

8 HPLC traces of compounds

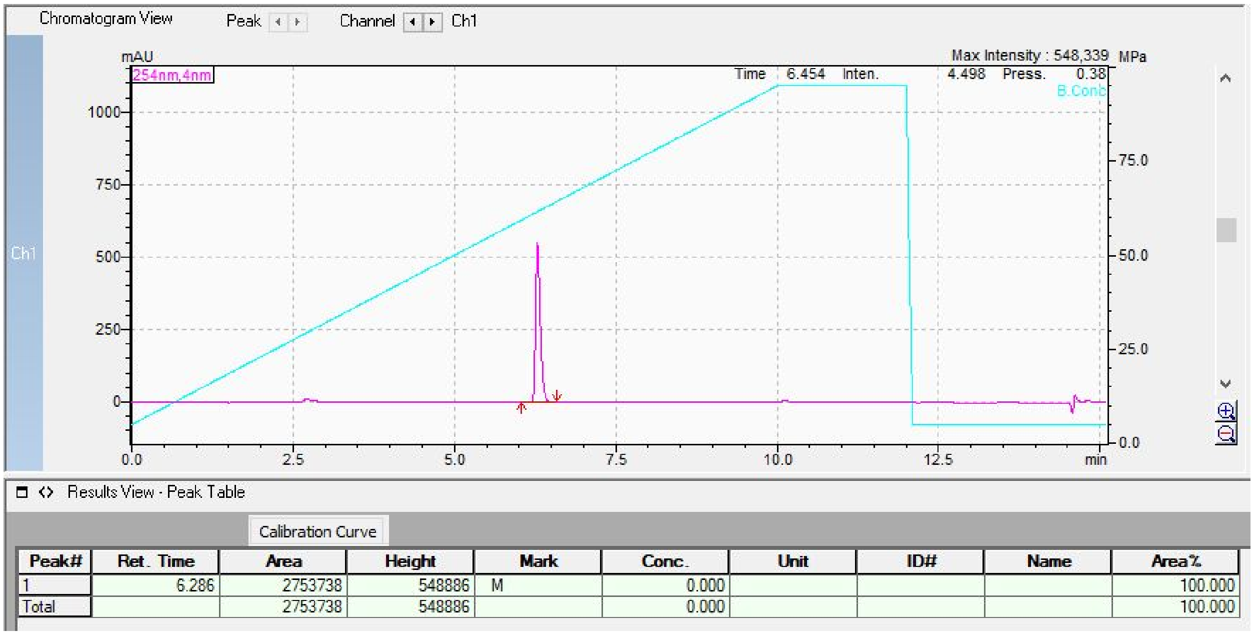

Figure S19. HPLC trace of compound **12e** (100 µM).

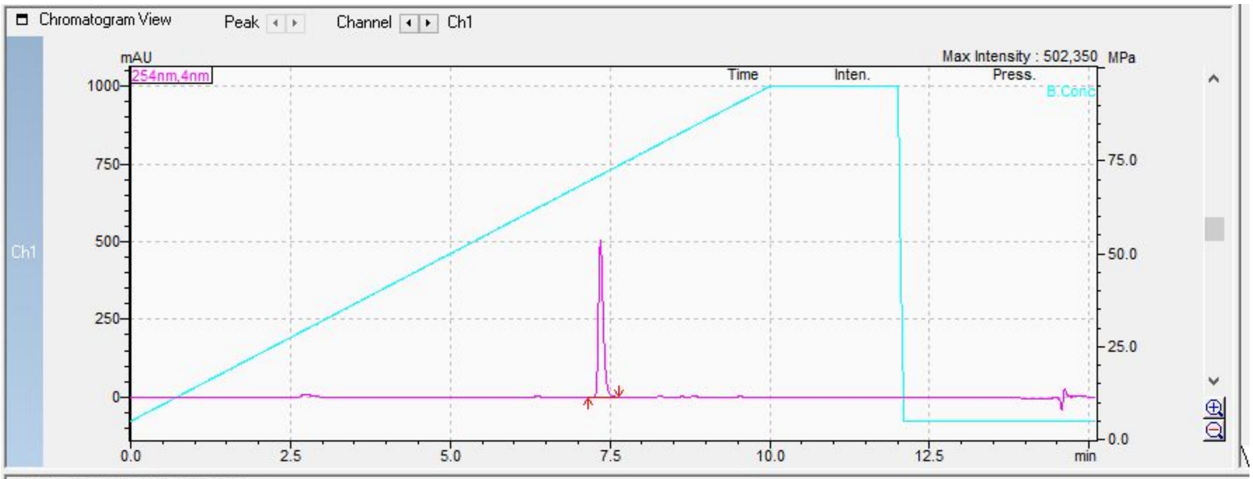

Results View - Peak Table

Calibration Curve

Peak#	Ret. Time	Area	Height	Mark	Conc.	Unit	ID#	Name	Area%
1	7.351	2539954	502259	M	0.000				100.000
Total		2539954	502259		0.000				100.000

Figure S19. HPLC trace of compound **12f** (100 µM).

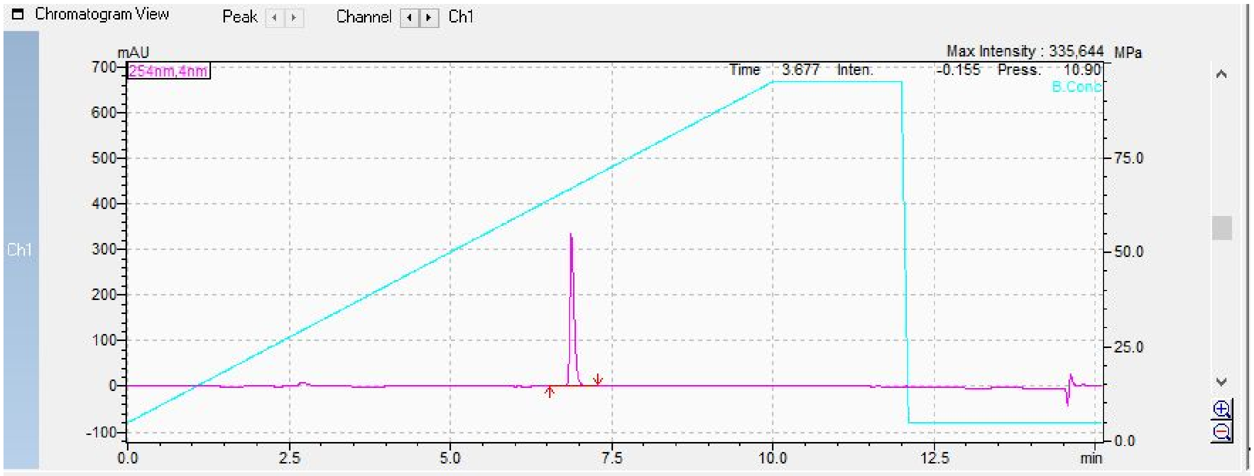

Results View - Peak Table

Peak Table

Group Calibration Curve

Peak#	Ret. Time	Area	Height	Mark	Conc.	Unit	ID#	Name	Area%
1	6.882	1621258	335362	M	0.000				100.000
Total		1621258	335362		0.000				100.000

Figure S20. HPLC trace of compound **3e** (100 µM).

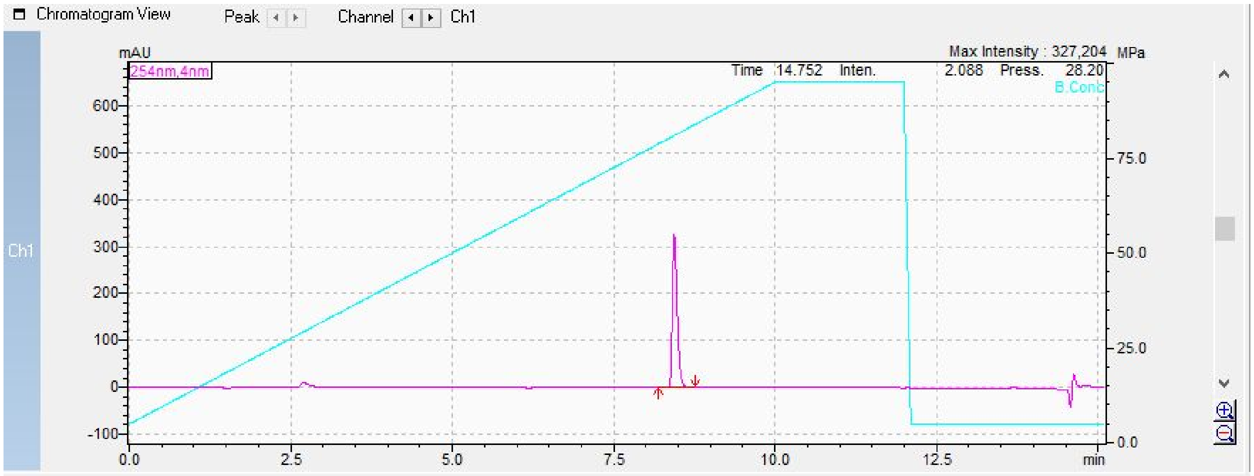

Results View - Peak Table

Peak Table | Group | Calibration Curve

Peak#	Ret. Time	Area	Height	Mark	Conc.	Unit	ID#	Name	Area%
1	8.440	1643099	326912	M	0.000				100.000
Total		1643099	326912		0.000				100.000

Figure S21. HPLC trace of compound **3f** (100 µM).

9 NMR spectra

Compound 3a

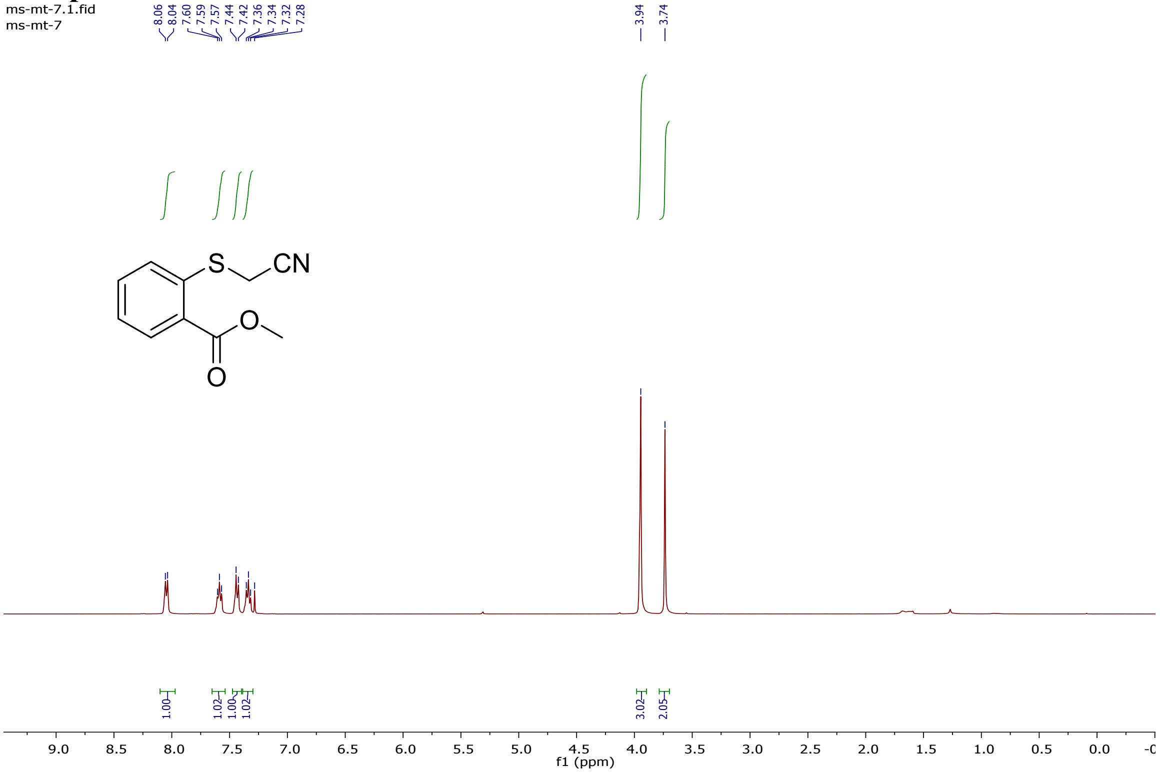

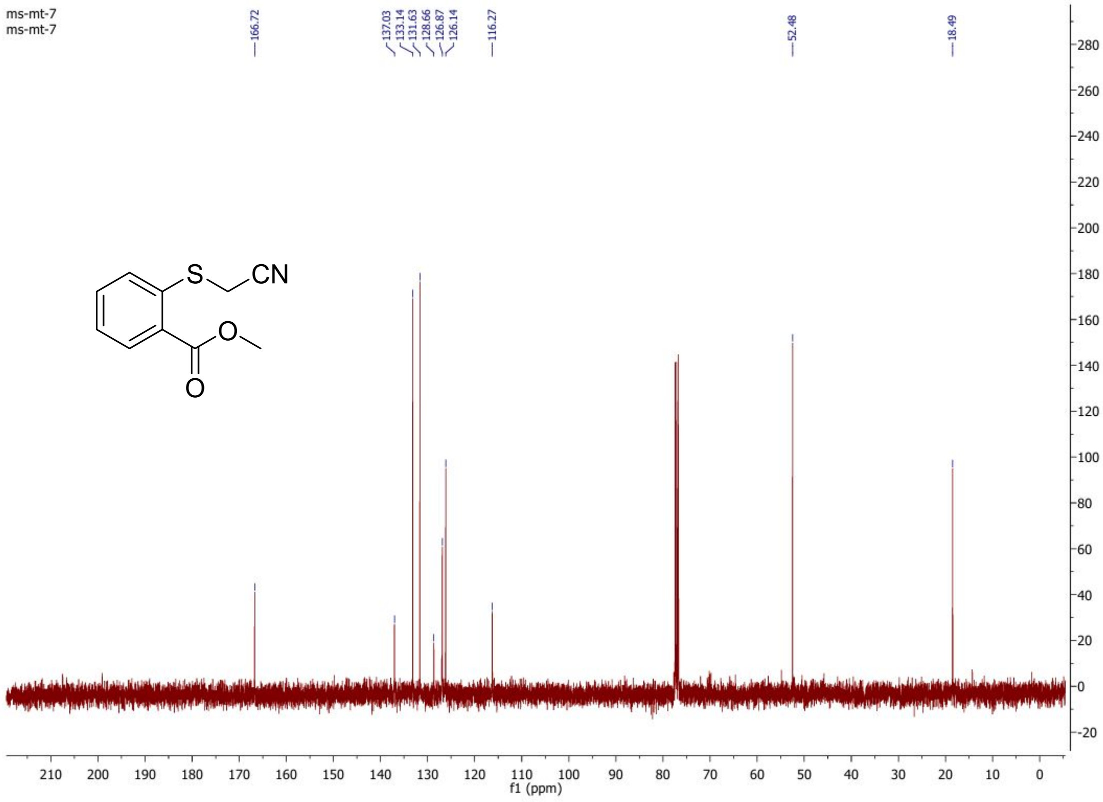

Compound 4a

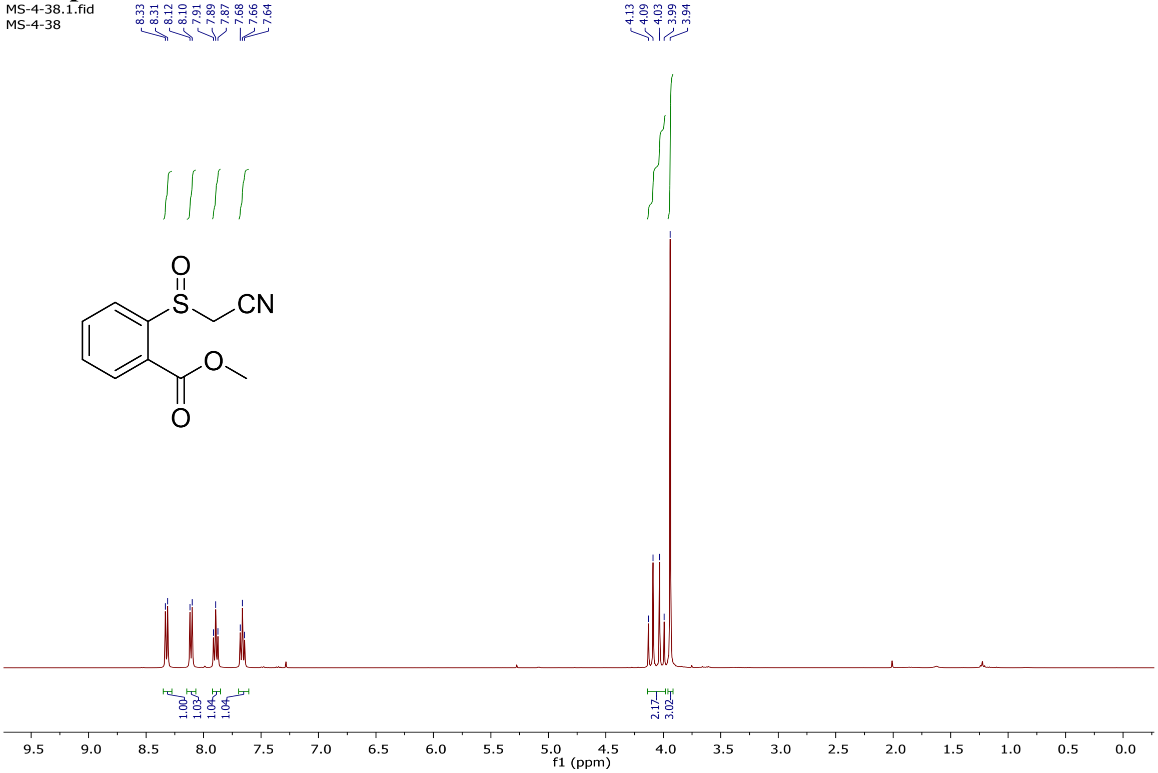
MS-4-38.1.fid
MS-4-38
8.33
8.31
8.12
8.10
7.91
7.89
7.87
7.68
7.66
7.64
4.13
4.09
4.03
3.99
3.94
O
S
CN
O
O
1.00
1.03
1.04
1.04
2.17
3.02
9.5 9.0 8.5 8.0 7.5 7.0 6.5 6.0 5.5 5.0 4.5 4.0 3.5 3.0 2.5 2.0 1.5 1.0 0.5 0.0
f1 (ppm)

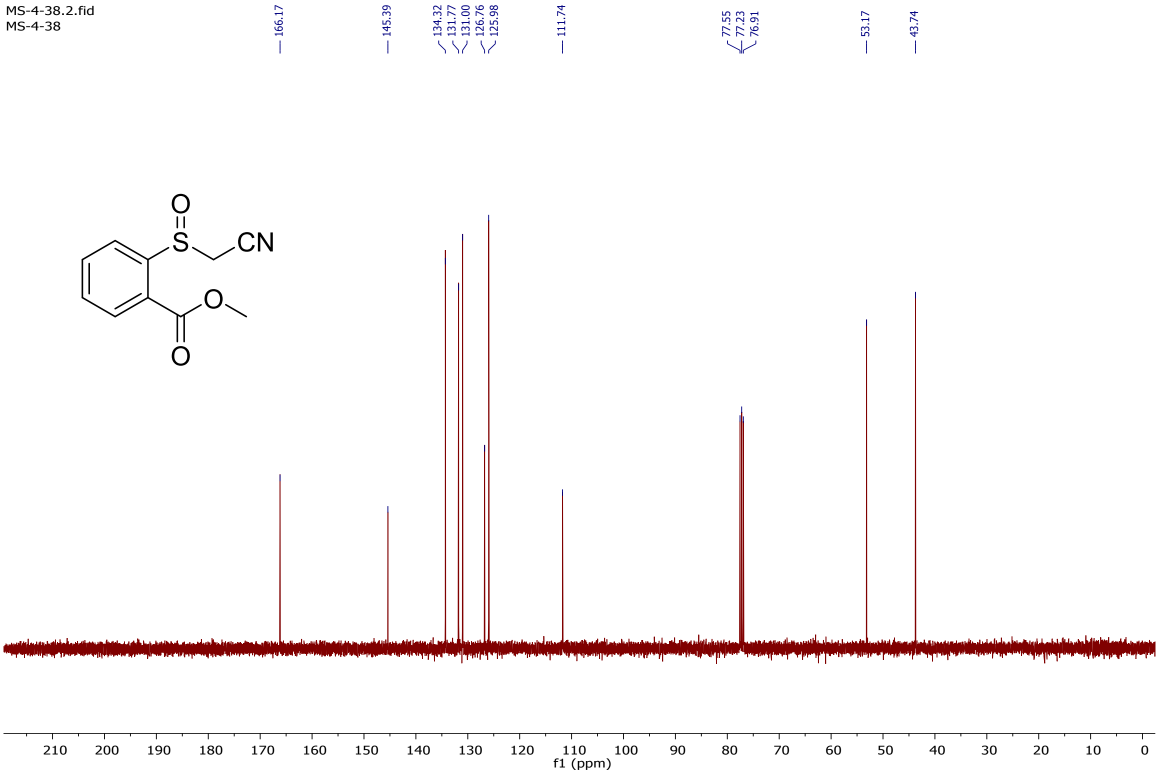
MS-4-38.2.fid
MS-4-38
166.17
145.39
134.32
131.77
131.00
126.76
125.98
111.74
77.55
77.23
76.91
53.17
43.74
O
S
CN
O
O
210 200 190 180 170 160 150 140 130 120 110 100 90 80 70 60 50 40 30 20 10 0
f1 (ppm)

Compound 5a

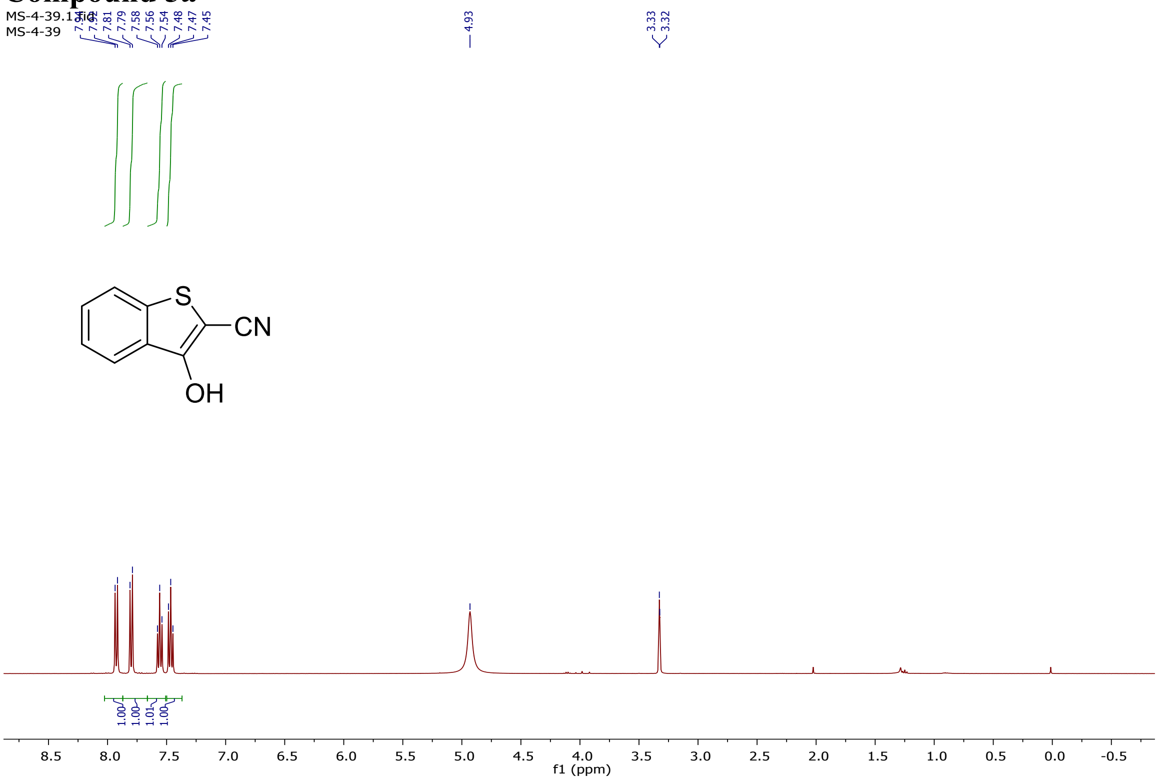
MS-4-39.1.fid
MS-4-39
4.93
3.33
3.32
CN
OH
S
1.00
1.00
1.01
1.00
f1 (ppm)

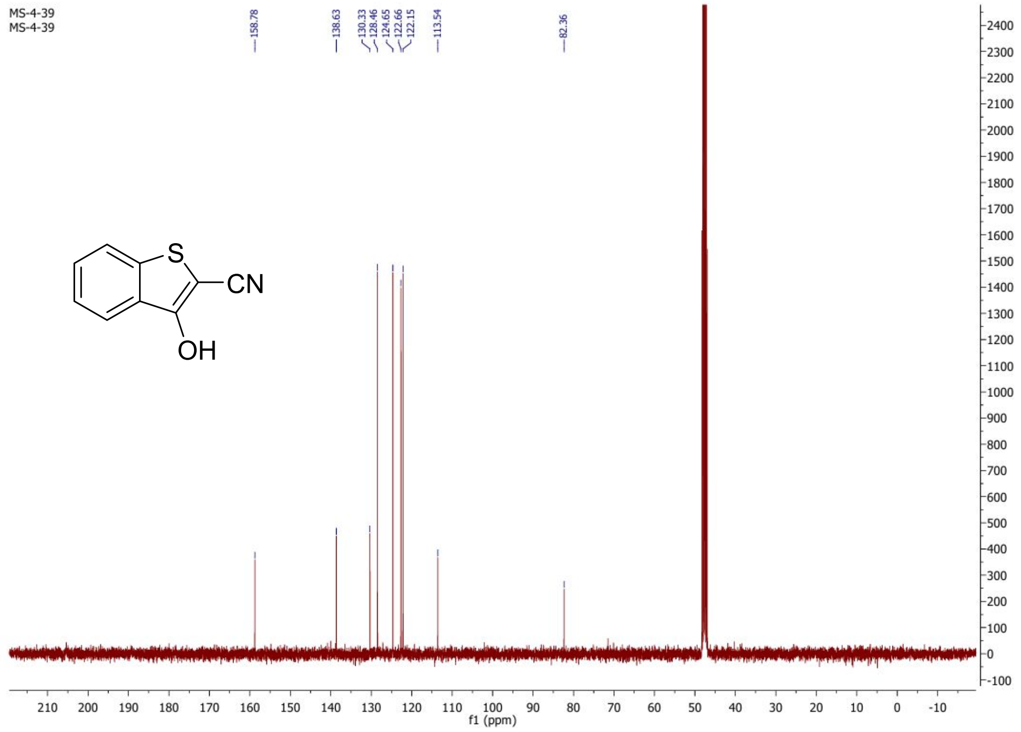
MS-4-39
MS-4-39
158.78
138.63
130.33
128.46
124.65
122.66
122.15
113.54
82.36
CN
OH
S
f1 (ppm)

Compound 6a

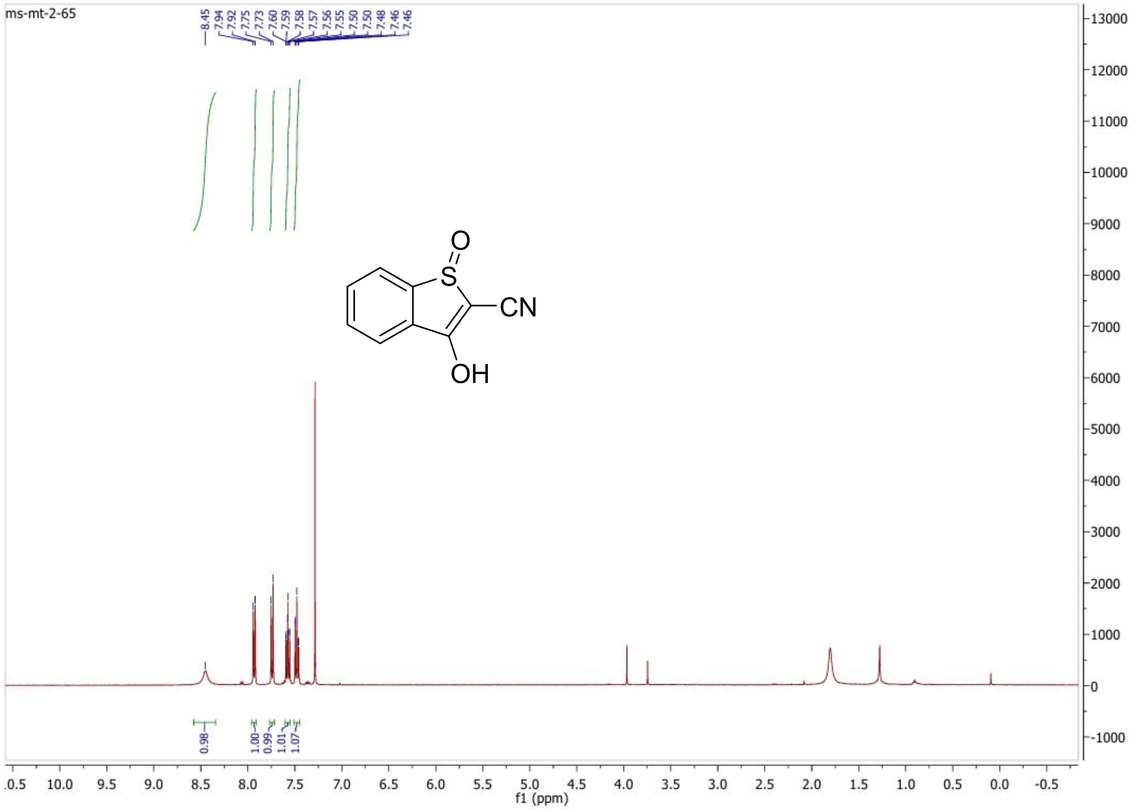
ms-mt-2-65
8.45
7.94
7.92
7.75
7.73
7.60
7.59
7.58
7.57
7.56
7.55
7.50
7.50
7.48
7.46
7.46
O
S
CN
OH
0.98
1.00
0.99
1.01
1.07
f1 (ppm)

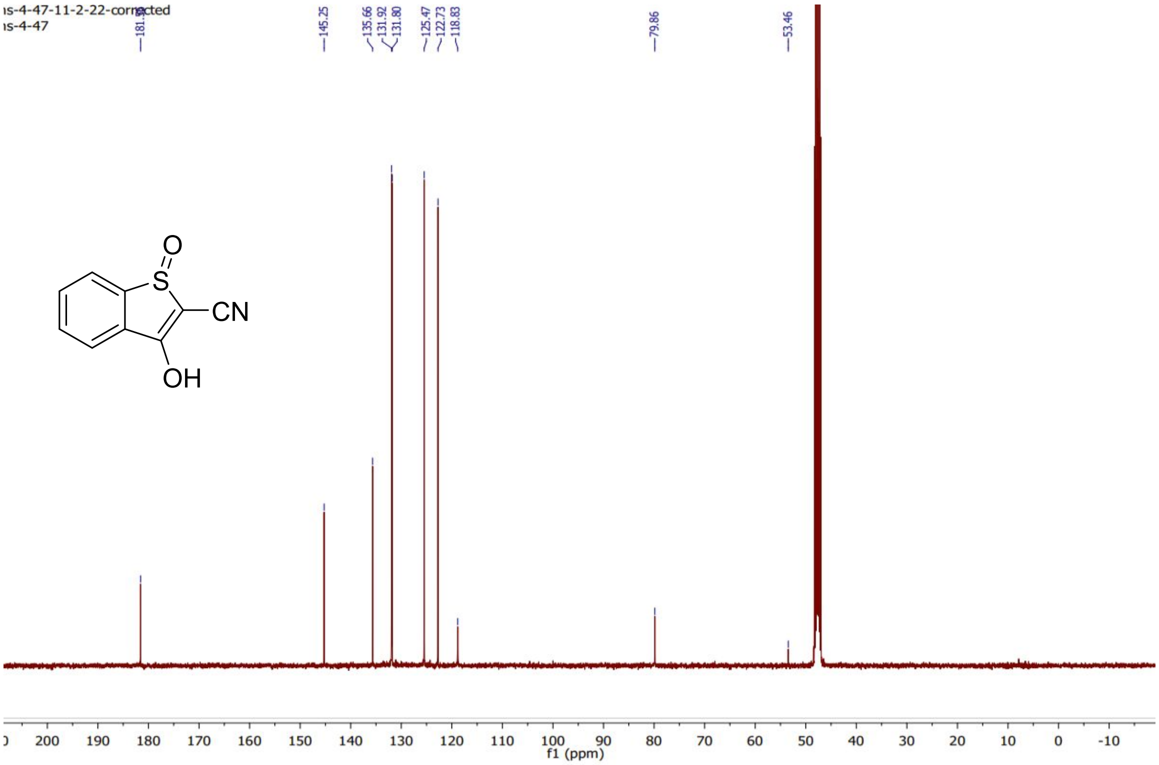
ms-4-47-11-2-22-corrected
ms-4-47
145.25
135.66
131.92
131.80
125.47
122.73
118.83
79.86
53.46
O
S
CN
OH
f1 (ppm)

Compound 3b

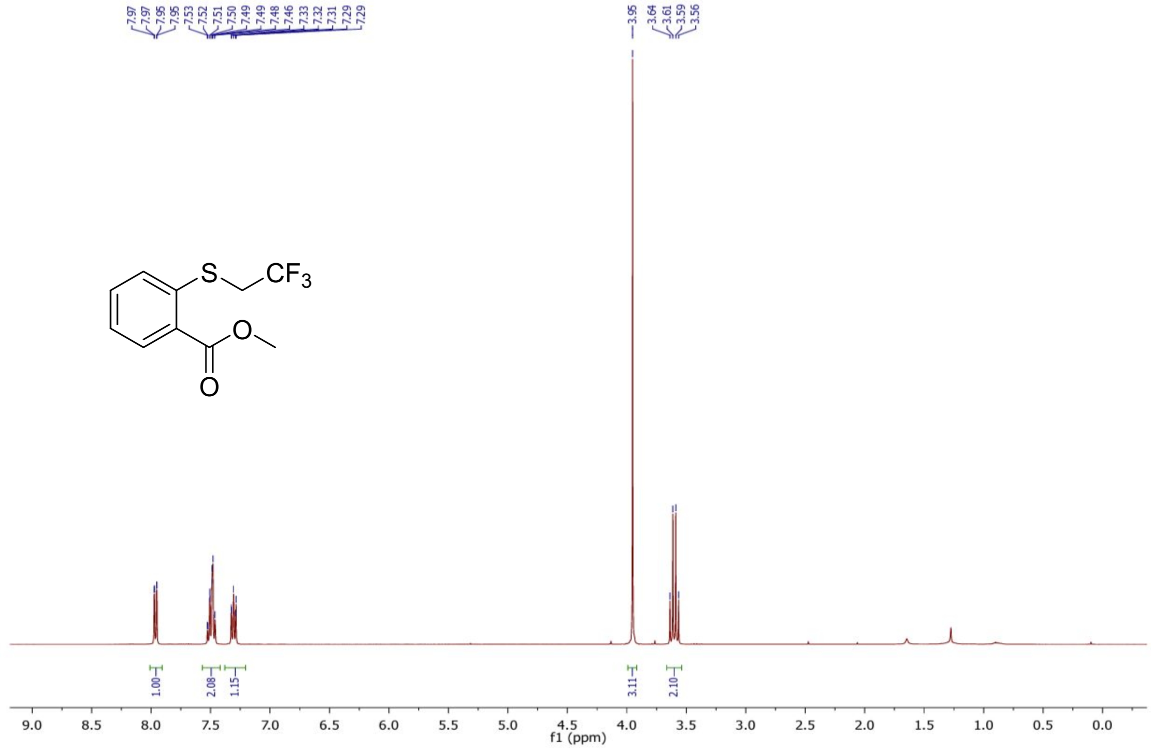

7.97 7.97 7.95 7.95 7.53 7.52 7.51 7.50 7.49 7.49 7.48 7.46 7.33 7.32 7.31 7.29 7.29
3.95
3.64 3.61 3.59 3.56
S
CF3
O
O
1.00
2.08
1.15
3.11
2.10
9.0 8.5 8.0 7.5 7.0 6.5 6.0 5.5 5.0 4.5 4.0 3.5 3.0 2.5 2.0 1.5 1.0 0.5 0.0
f1 (ppm)

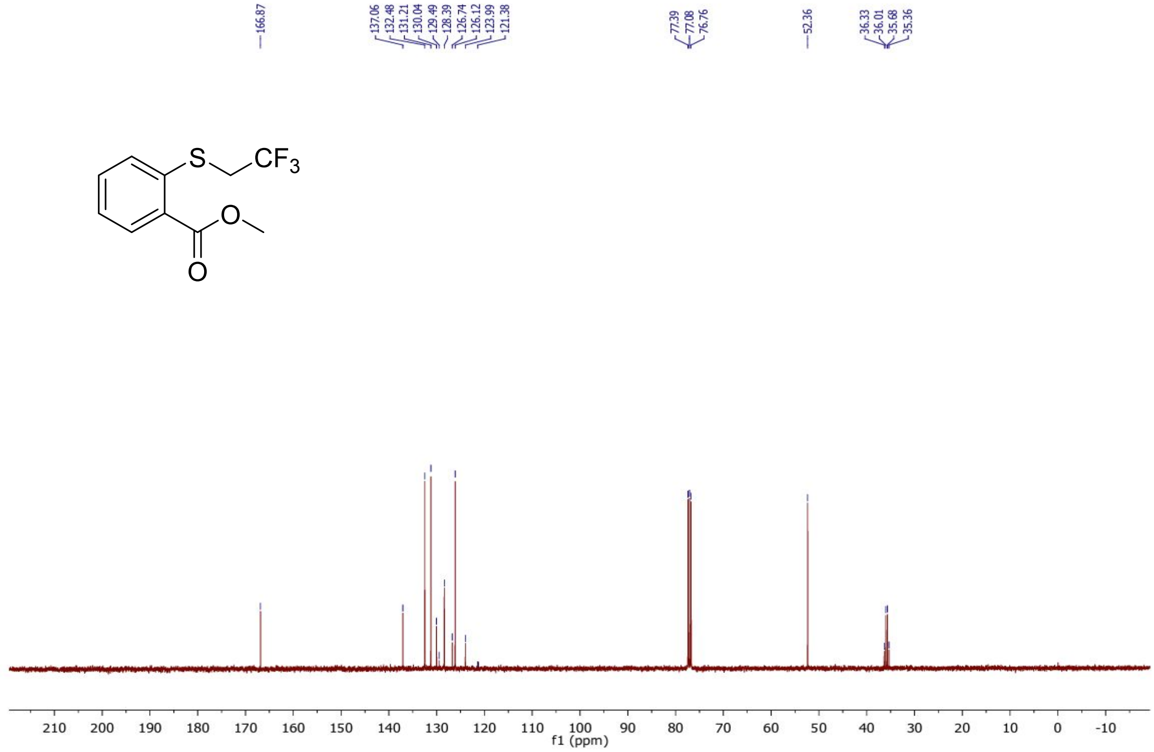

166.87
137.06 132.48 131.21 130.04 129.49 128.39 126.74 126.12 123.99 121.38
77.39 77.08 76.76
52.36
36.33 36.01 35.68 35.36
S
CF3
O
O
210 200 190 180 170 160 150 140 130 120 110 100 90 80 70 60 50 40 30 20 10 0 -10
f1 (ppm)

Compound 4b

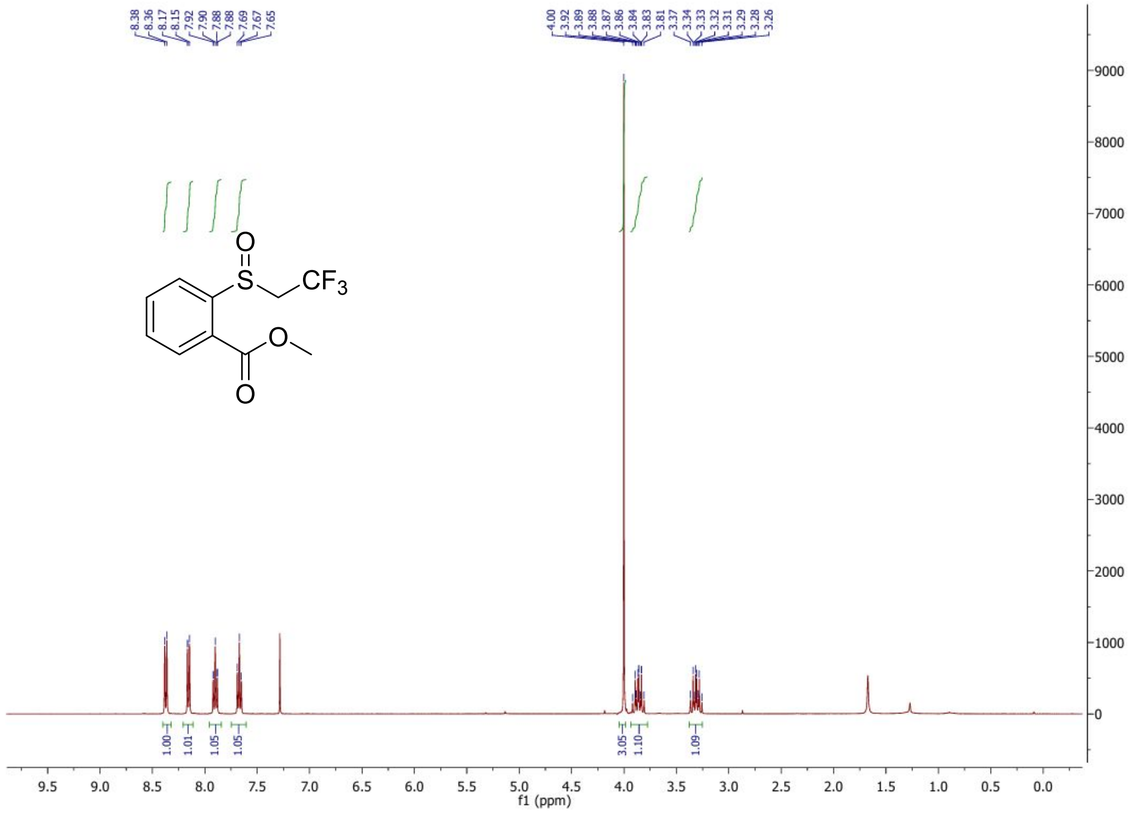

8.38
8.36
8.17
8.15
7.92
7.90
7.88
7.88
7.69
7.67
7.65
4.00
3.92
3.89
3.88
3.87
3.86
3.84
3.83
3.81
3.37
3.34
3.33
3.32
3.31
3.29
3.28
3.26
1.00
1.01
1.05
1.05
3.05
1.10
1.09
f1 (ppm)

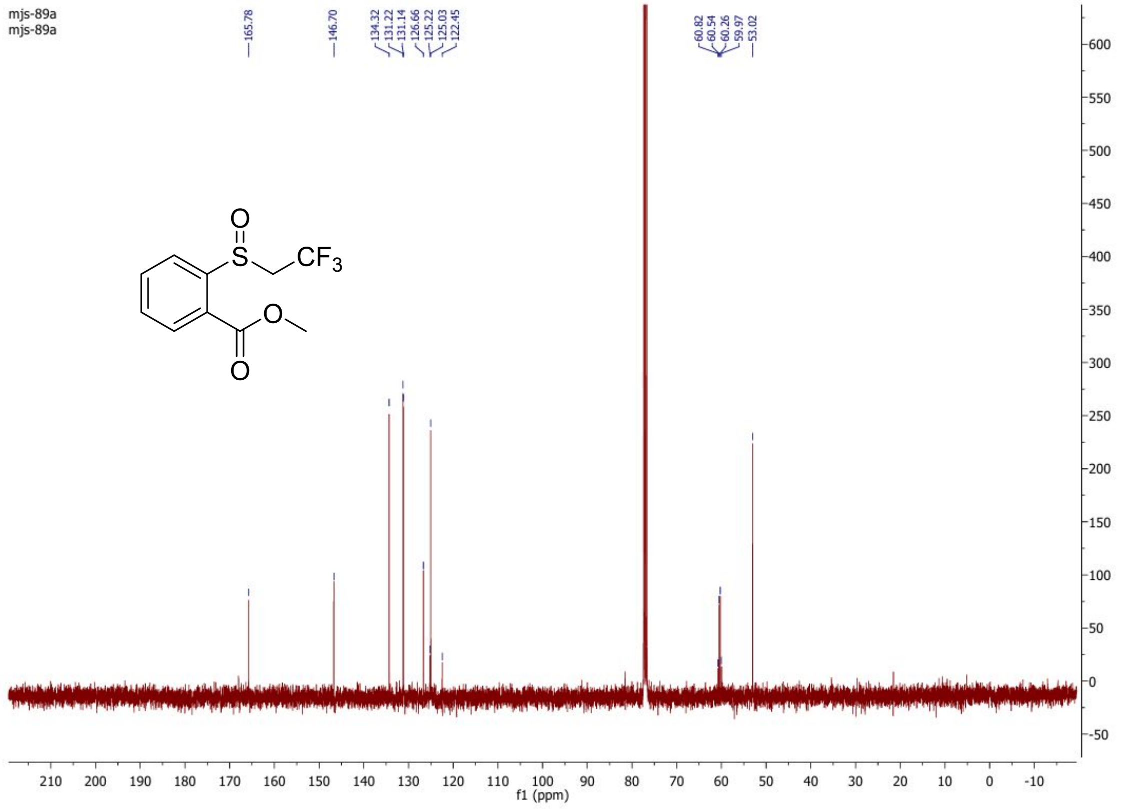

mjs-89a
mjs-89a
165.78
146.70
134.32
131.22
131.14
126.66
125.22
125.03
122.45
60.82
60.54
60.26
59.97
53.02
f1 (ppm)

Compound 3c

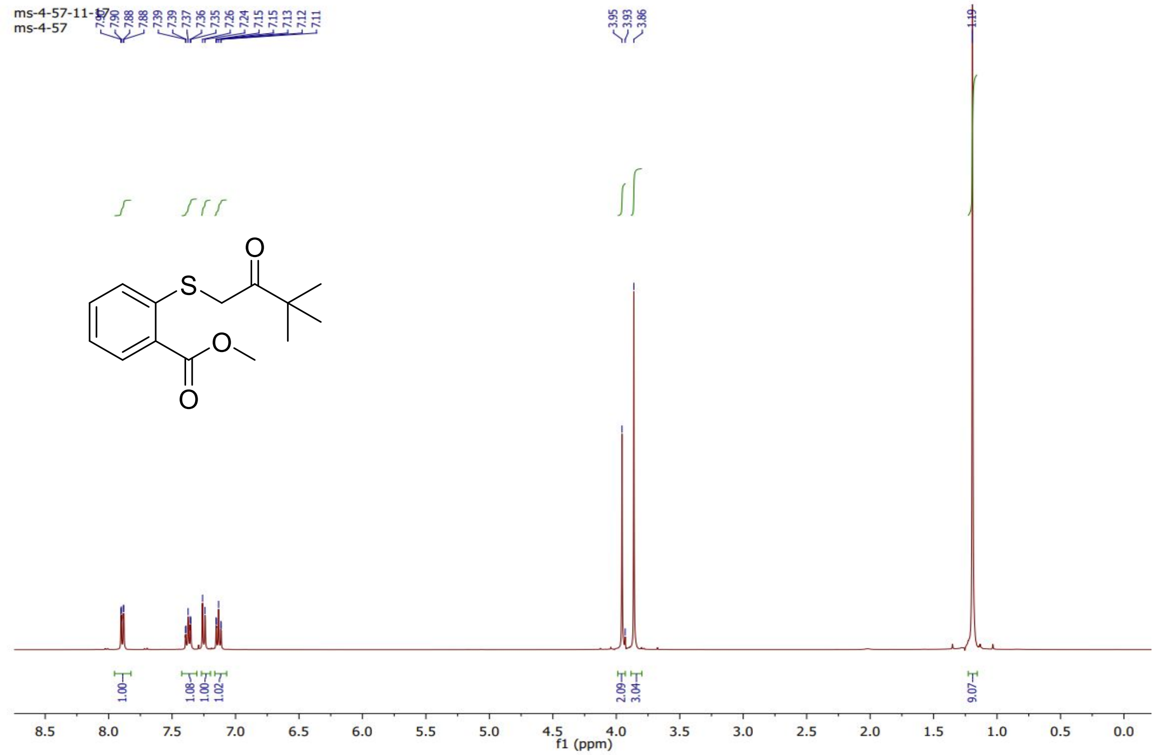
ms-4-57-11-17
ms-4-57
7.90
7.88
7.39
7.39
7.37
7.36
7.35
7.26
7.24
7.15
7.15
7.13
7.12
7.11
3.95
3.93
3.86
1.19
O
S
O
O
1.00
1.08
1.00
1.02
2.09
3.04
9.07
8.5 8.0 7.5 7.0 6.5 6.0 5.5 5.0 4.5 4.0 3.5 3.0 2.5 2.0 1.5 1.0 0.5 0.0
f1 (ppm)

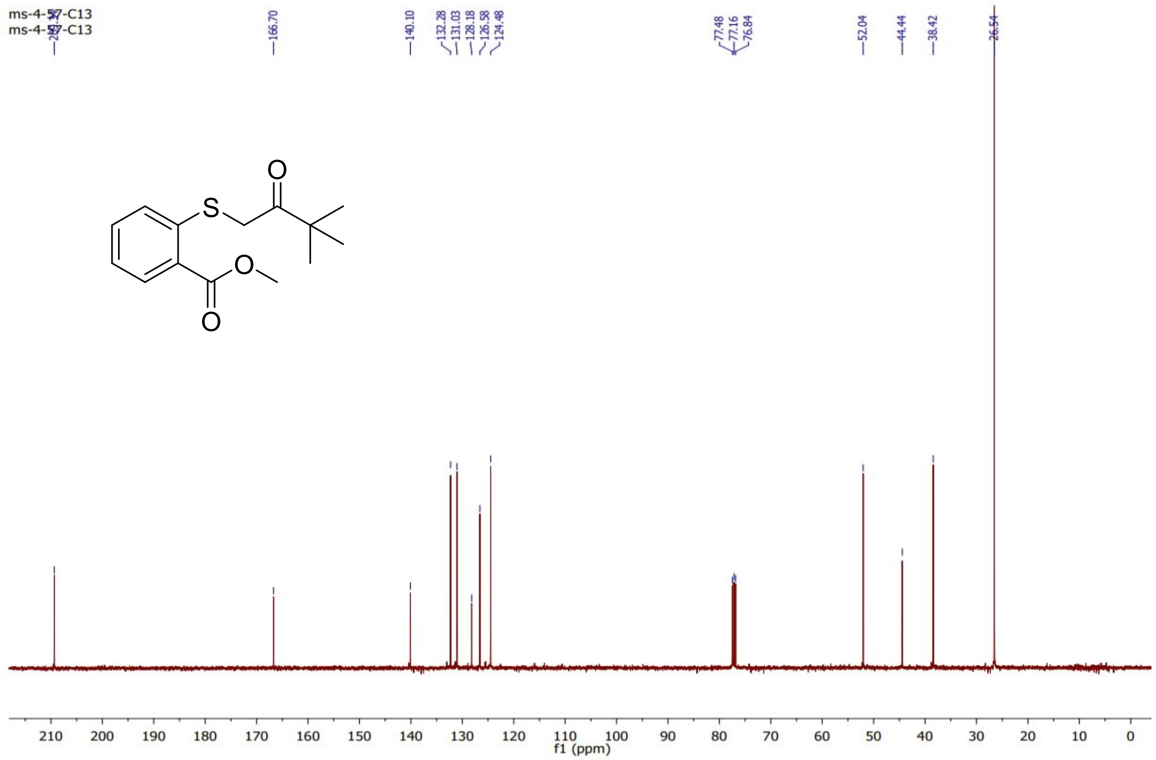
ms-4-57-C13
ms-4-57-C13
166.70
140.10
132.28
131.03
128.18
126.58
124.48
77.48
77.16
76.84
52.04
44.44
38.42
26.54
O
S
O
O
210 200 190 180 170 160 150 140 130 120 110 100 90 80 70 60 50 40 30 20 10 0
f1 (ppm)

Compound 4c

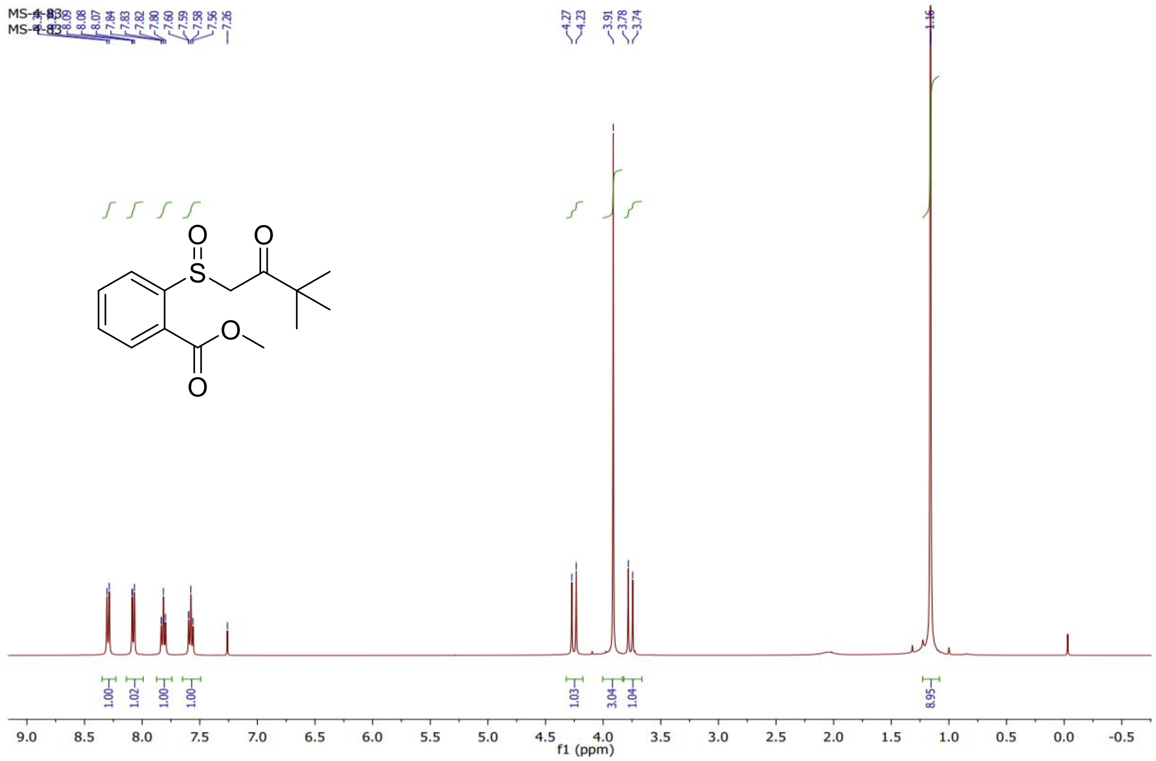
MS-4-83
MS-4-83
8.09
8.08
8.07
7.84
7.83
7.82
7.80
7.60
7.59
7.58
7.56
7.26
4.27
4.23
3.91
3.78
3.74
1.16
1.00
1.02
1.00
1.00
1.03
3.04
1.04
8.95
f1 (ppm)

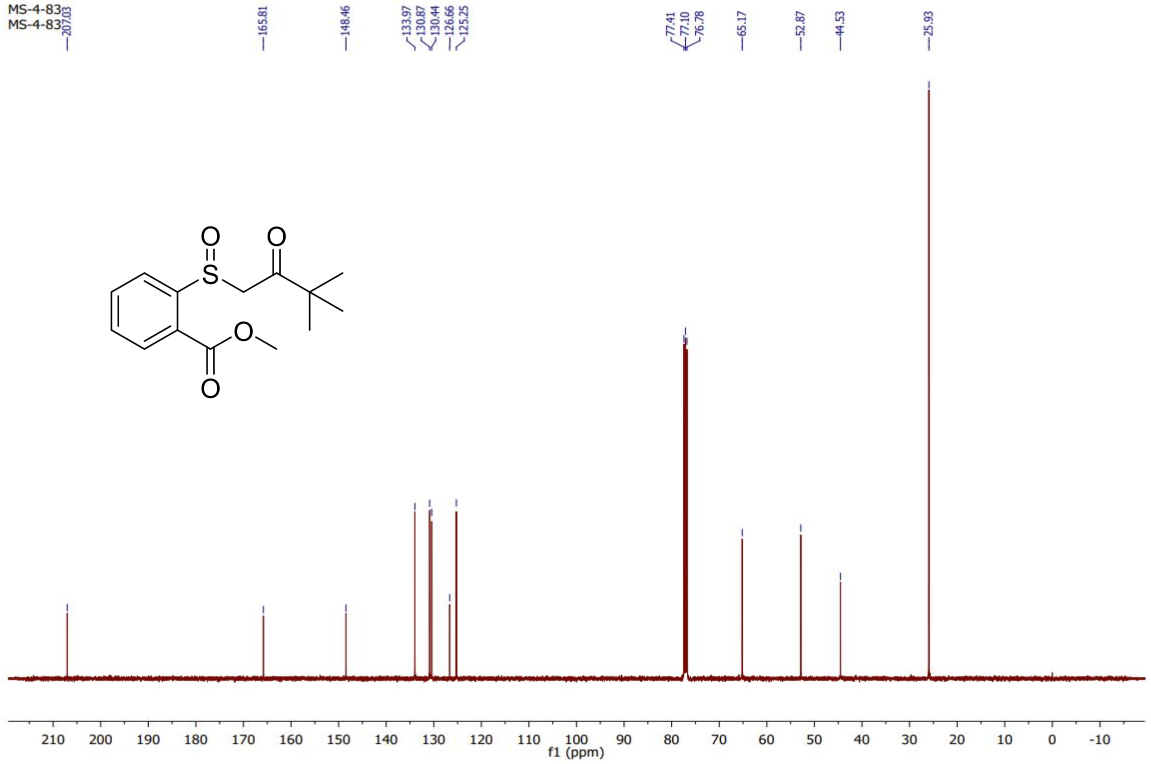
MS-4-83
MS-4-83
207.03
165.81
148.46
133.97
130.87
130.44
126.66
125.25
77.41
77.10
76.78
65.17
52.87
44.53
25.93
f1 (ppm)

Compound 5c

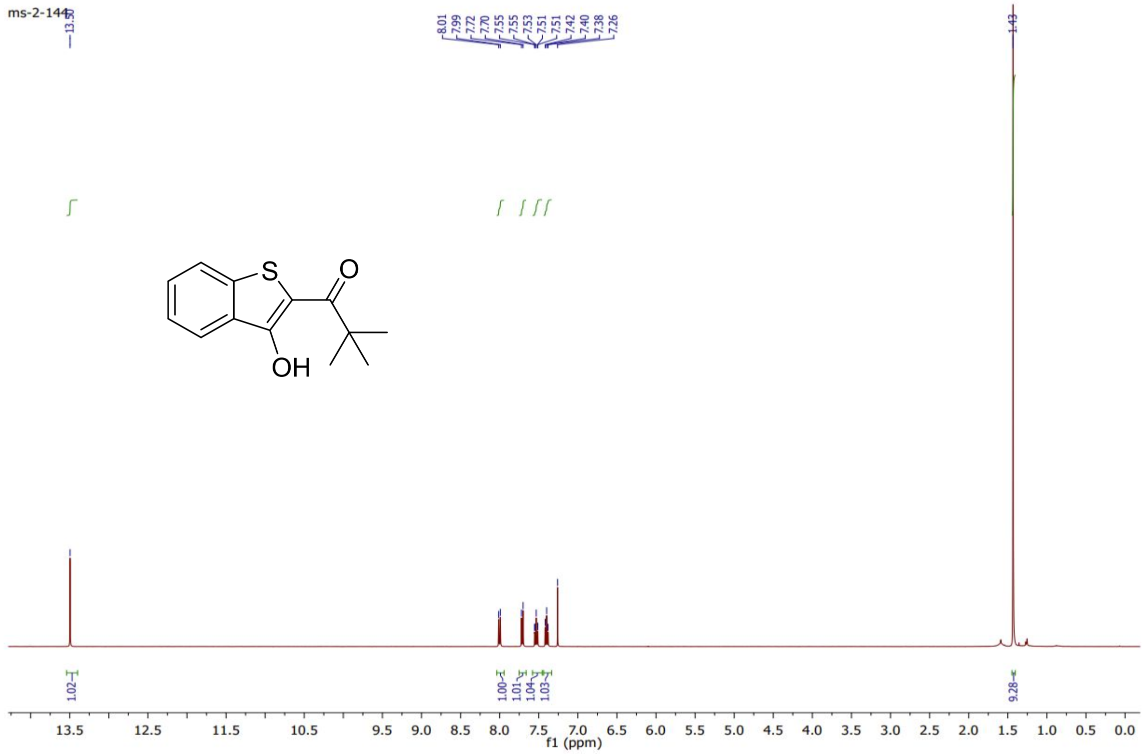
ms-2-144
13.50
8.01 7.99 7.72 7.70 7.55 7.55 7.53 7.51 7.51 7.42 7.40 7.38 7.26
1.43
S
O
OH
1.02
1.00 1.01 1.04 1.03
9.28
f1 (ppm)

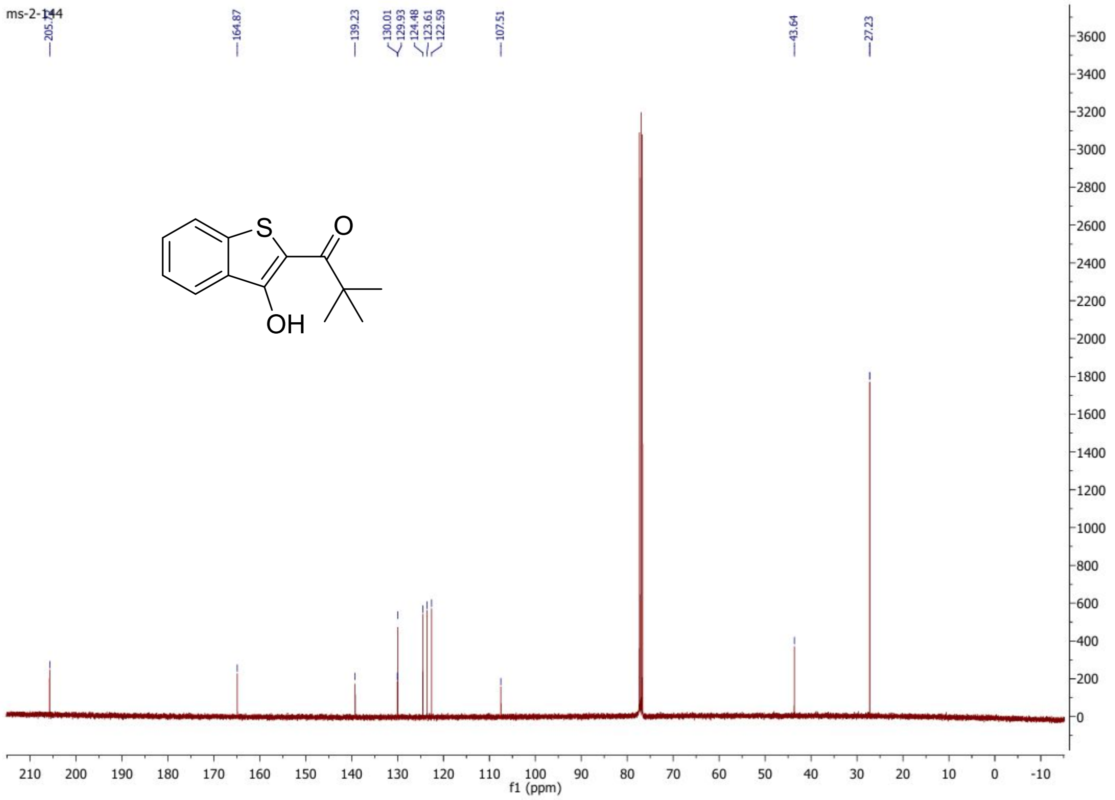
ms-2-144
164.87
139.23
130.01 129.93 124.48 123.61 122.59
107.51
43.64
27.23
S
O
OH
f1 (ppm)

Compound 6c

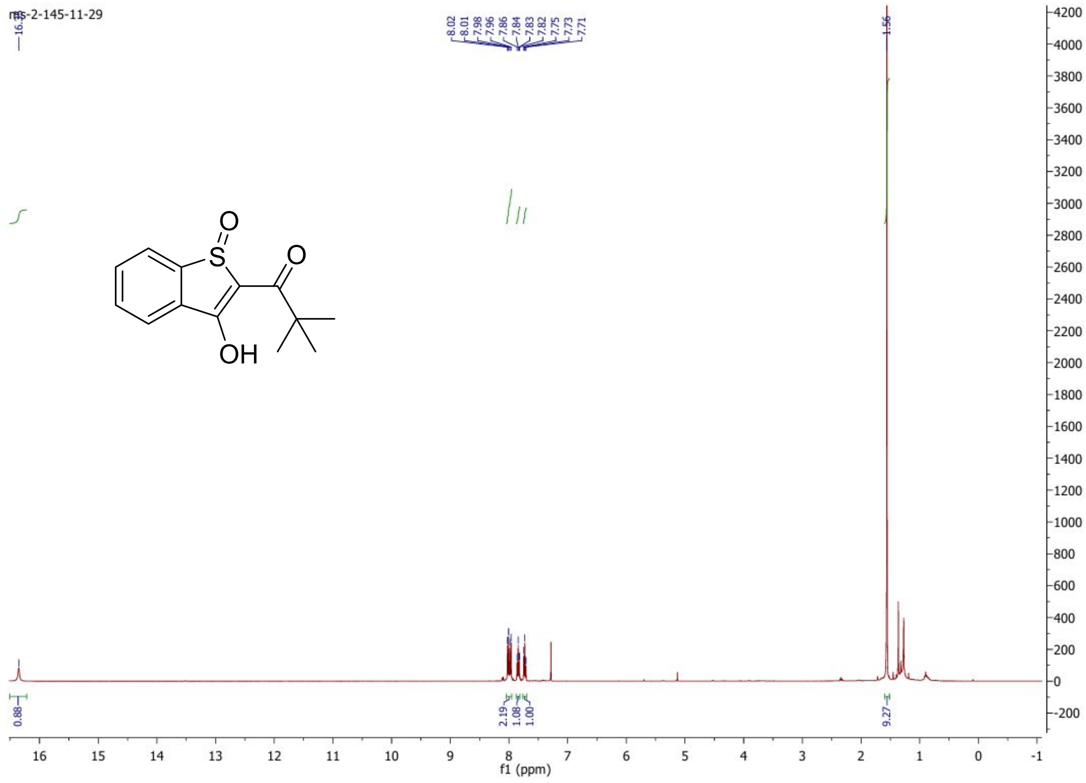

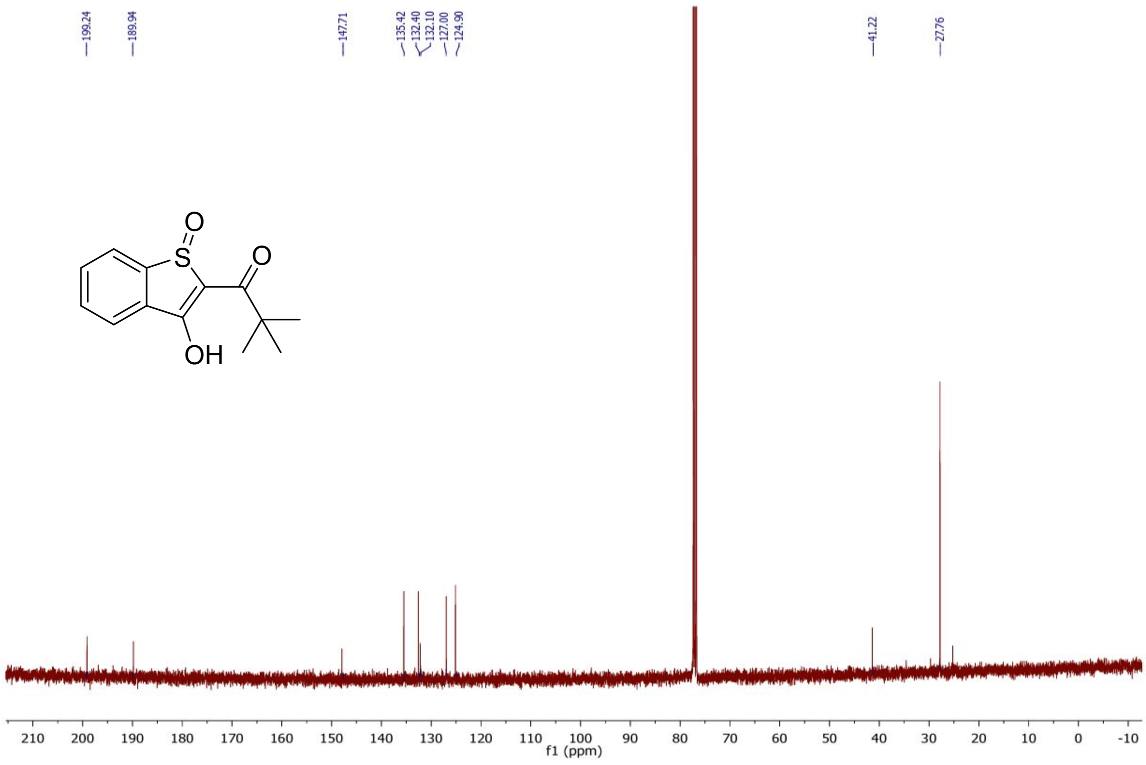

Compound 3d

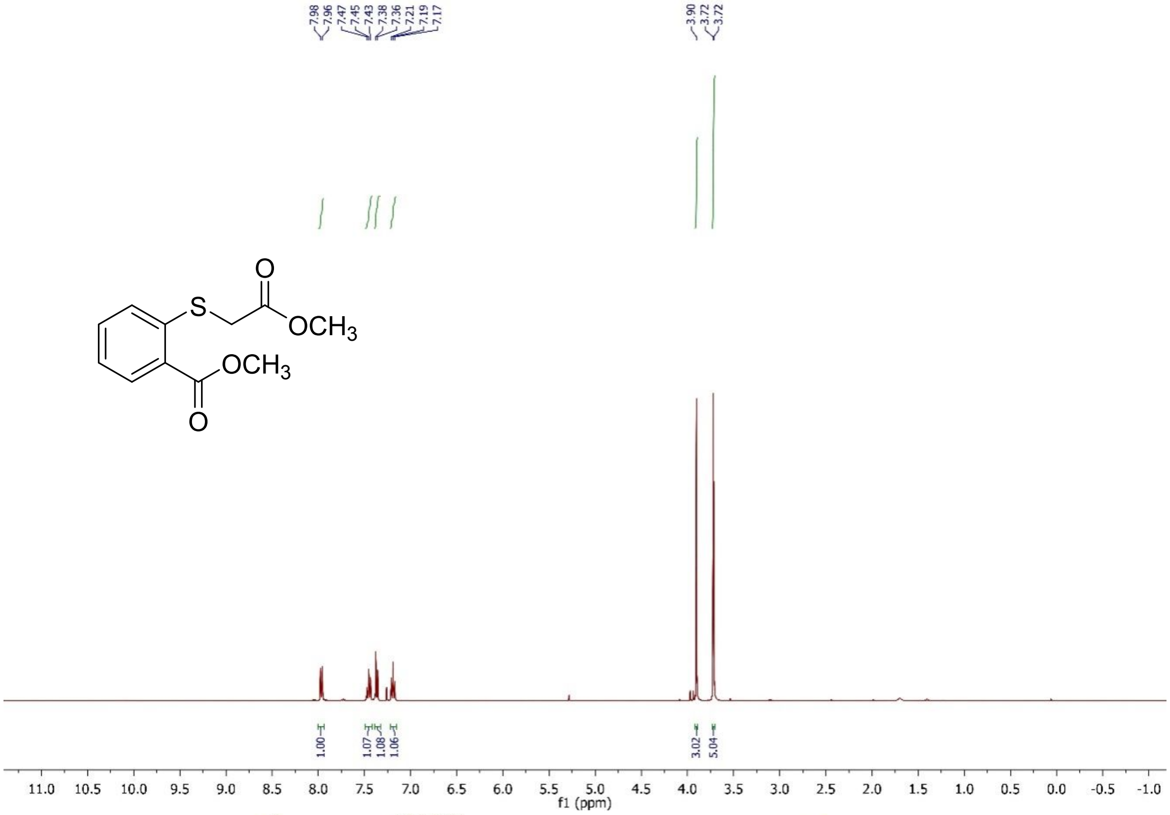
7.98
7.96
7.47
7.45
7.43
7.38
7.36
7.21
7.19
7.17
3.90
3.72
3.72
O
S
OCH3
OCH3
O
1.00
1.07
1.08
1.06
3.02
5.04
11.0 10.5 10.0 9.5 9.0 8.5 8.0 7.5 7.0 6.5 6.0 5.5 5.0 4.5 4.0 3.5 3.0 2.5 2.0 1.5 1.0 0.5 0.0 -0.5 -1.0
f1 (ppm)

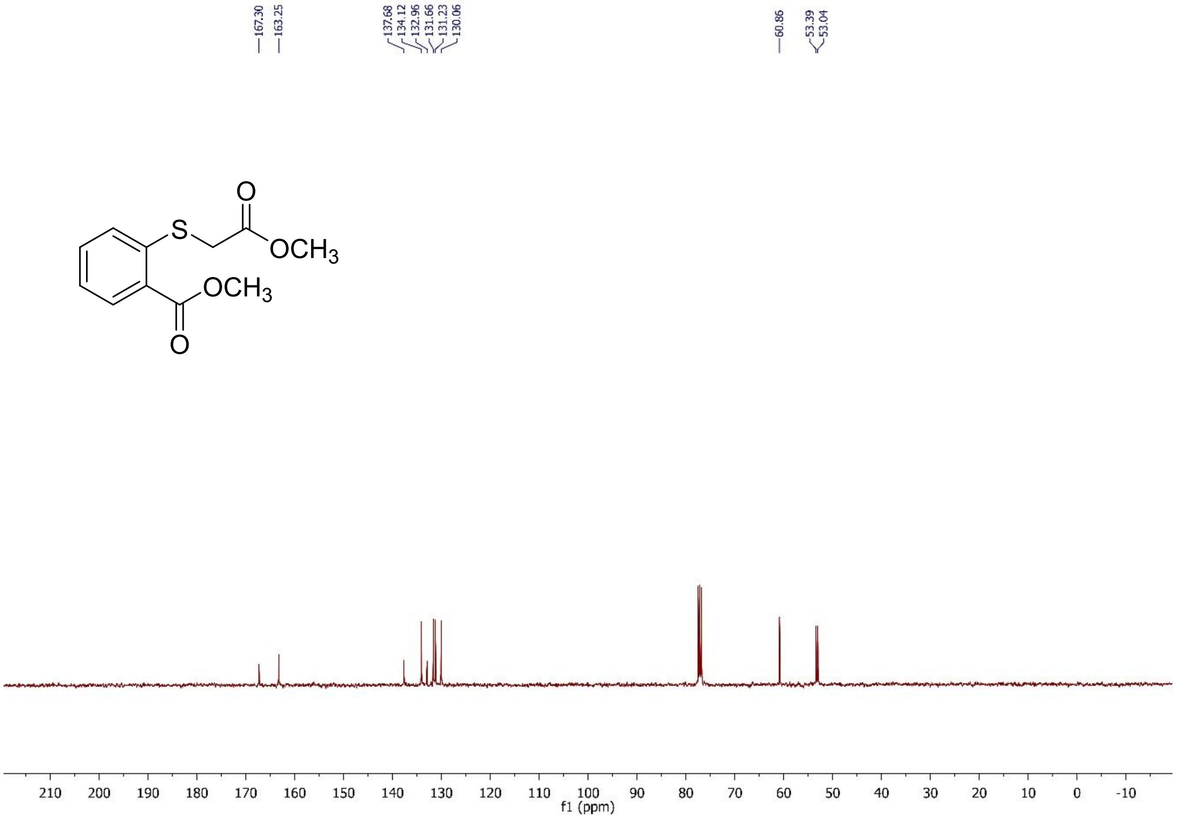
167.30
163.25
137.68
134.12
132.96
131.66
131.23
130.06
60.86
53.39
53.04
O
S
OCH3
OCH3
O
210 200 190 180 170 160 150 140 130 120 110 100 90 80 70 60 50 40 30 20 10 0 -10
f1 (ppm)

Compound 4d

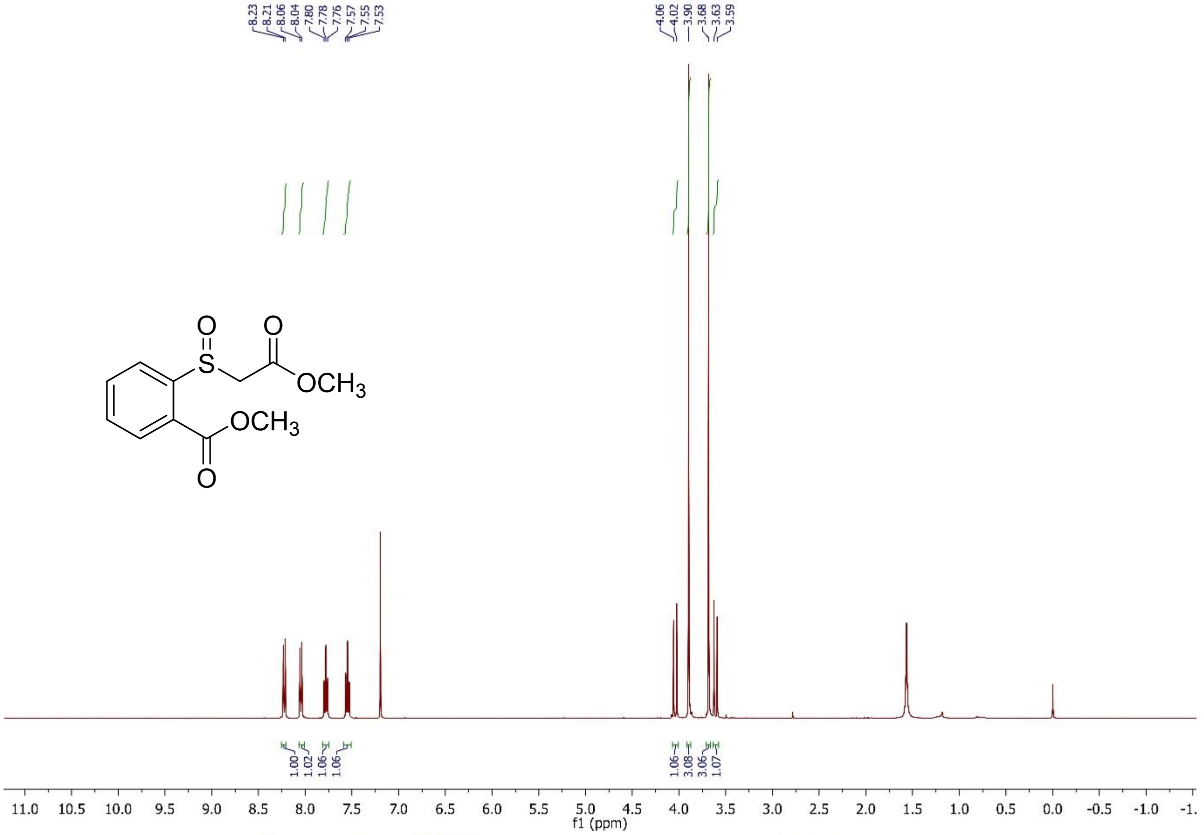
8.23
8.21
8.06
8.04
7.80
7.78
7.76
7.57
7.55
7.53
4.06
4.02
3.90
3.68
3.63
3.59
O
O
S
OCH3
OCH3
O
1.00
1.02
1.06
1.06
1.06
3.08
3.06
1.07
11.0 10.5 10.0 9.5 9.0 8.5 8.0 7.5 7.0 6.5 6.0 5.5 5.0 4.5 4.0 3.5 3.0 2.5 2.0 1.5 1.0 0.5 0.0 -0.5 -1.0
f1 (ppm)

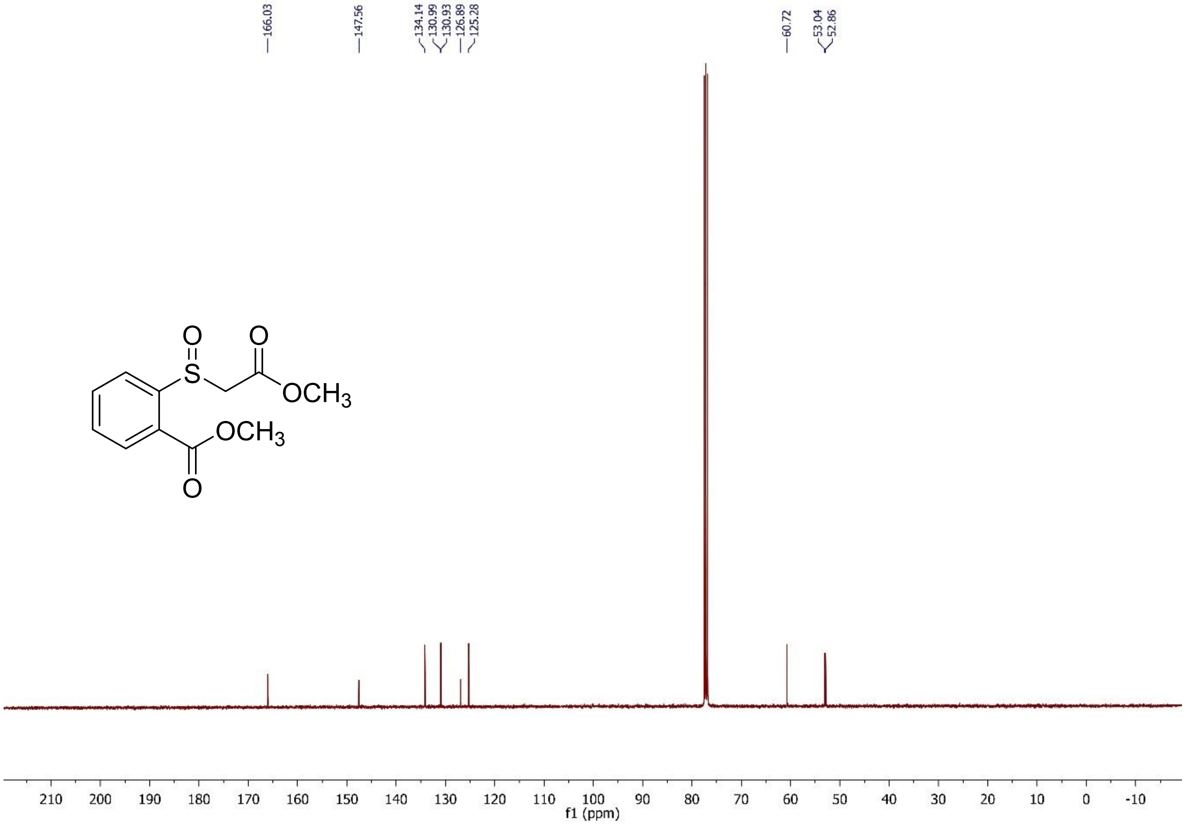
166.03
147.56
134.14
130.99
130.93
126.89
125.28
60.72
53.04
52.86
O
O
S
OCH3
OCH3
O
210 200 190 180 170 160 150 140 130 120 110 100 90 80 70 60 50 40 30 20 10 0 -10
f1 (ppm)

Compound 5d

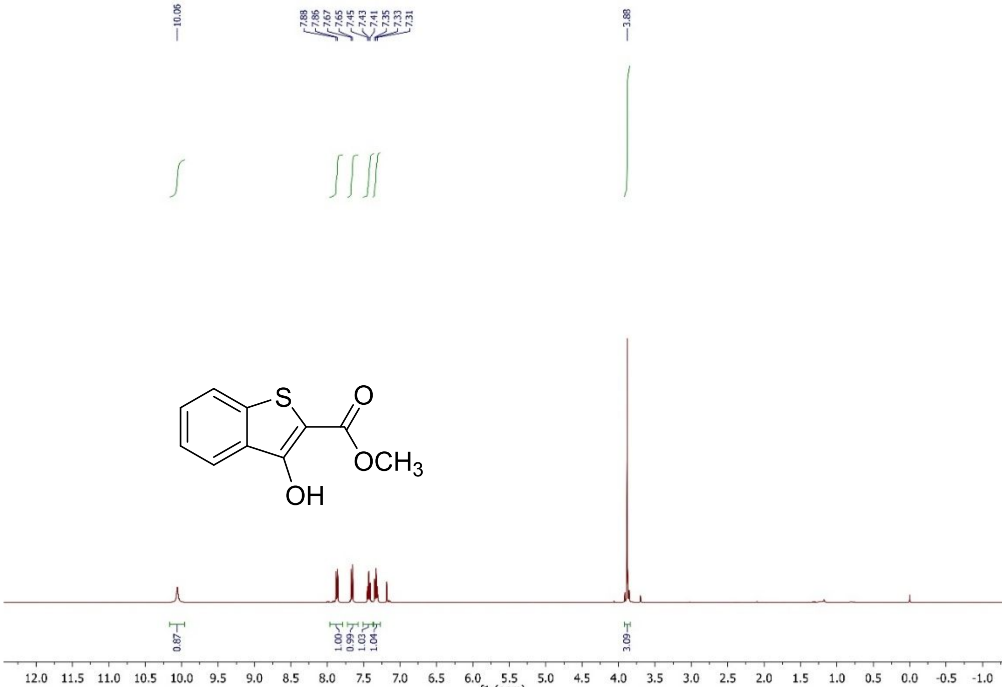

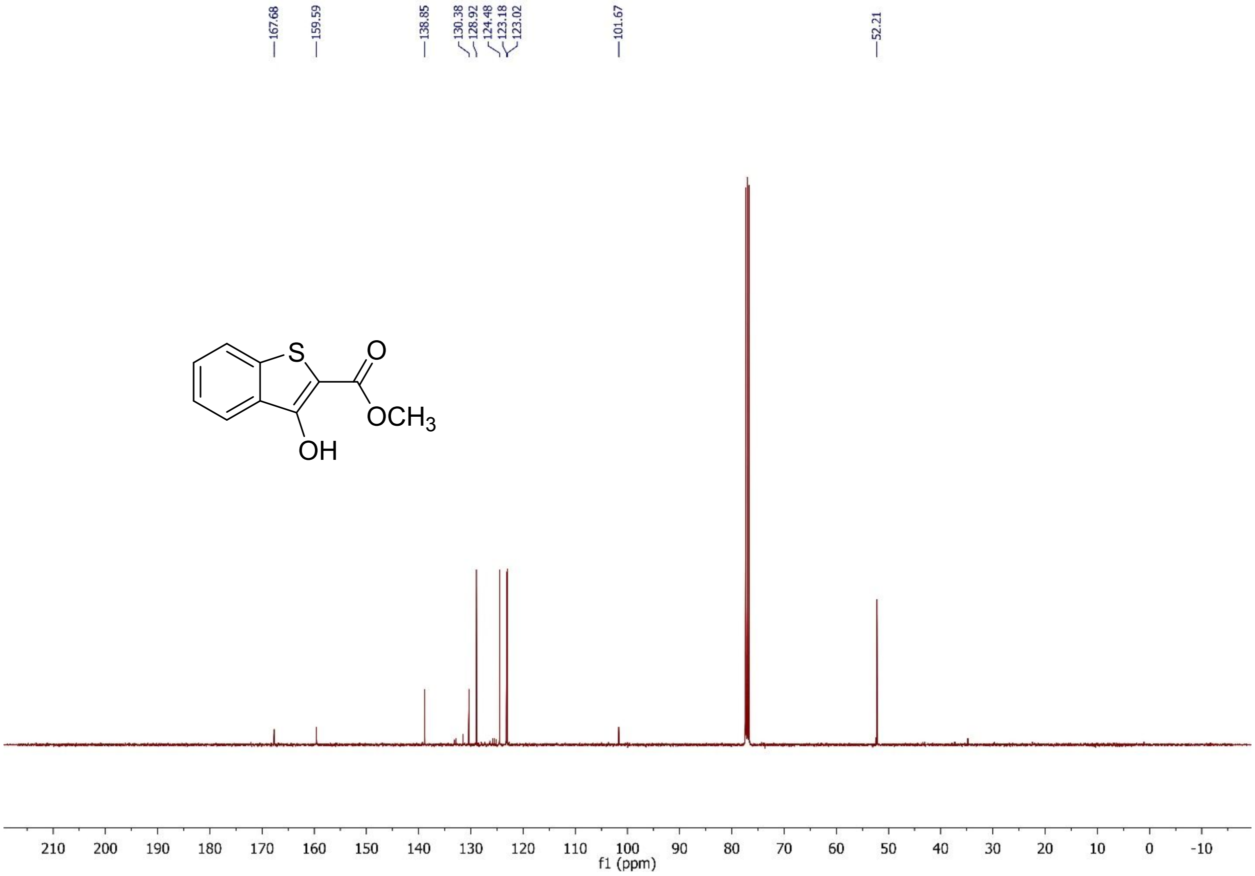

Compound 6d

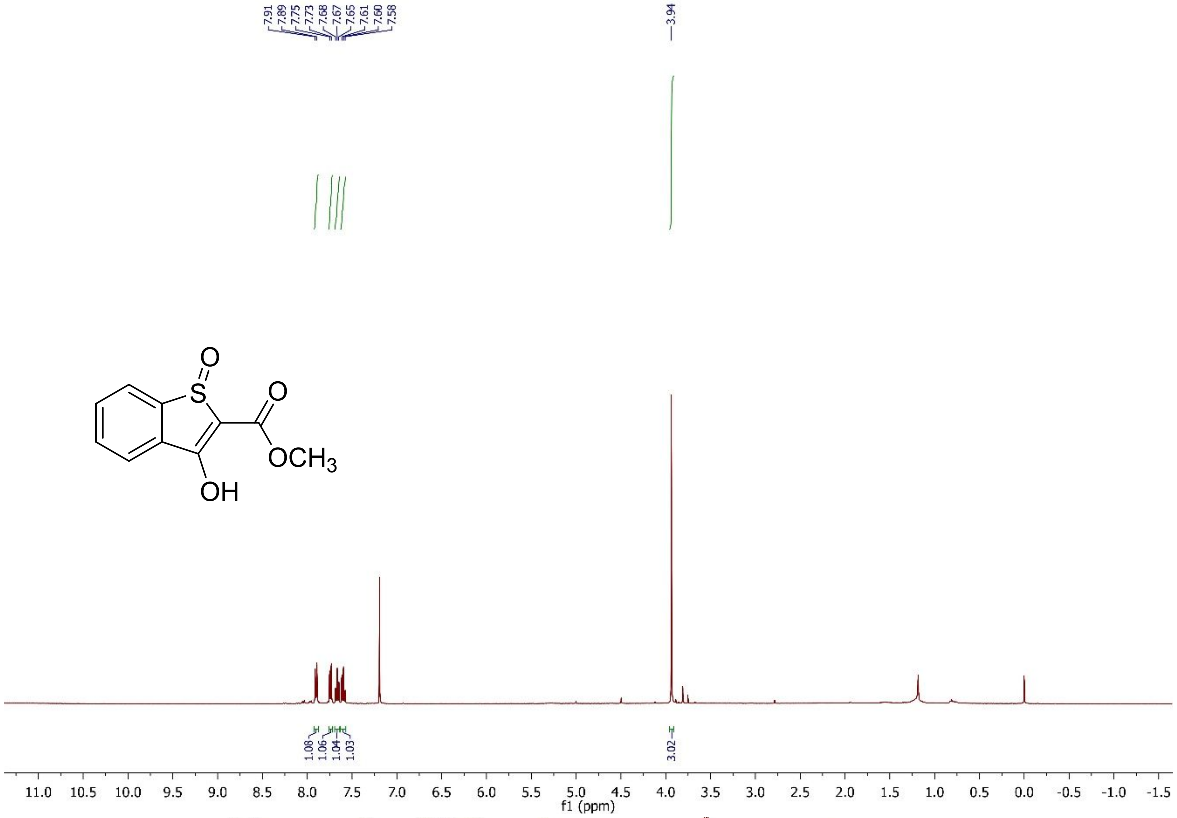
7.91
7.89
7.75
7.73
7.68
7.67
7.65
7.61
7.60
7.58
3.94
O
S
O
OCH3
OH
1.08
1.06
1.04
1.03
3.02
11.0 10.5 10.0 9.5 9.0 8.5 8.0 7.5 7.0 6.5 6.0 5.5 5.0 4.5 4.0 3.5 3.0 2.5 2.0 1.5 1.0 0.5 0.0 -0.5 -1.0 -1.5
f1 (ppm)

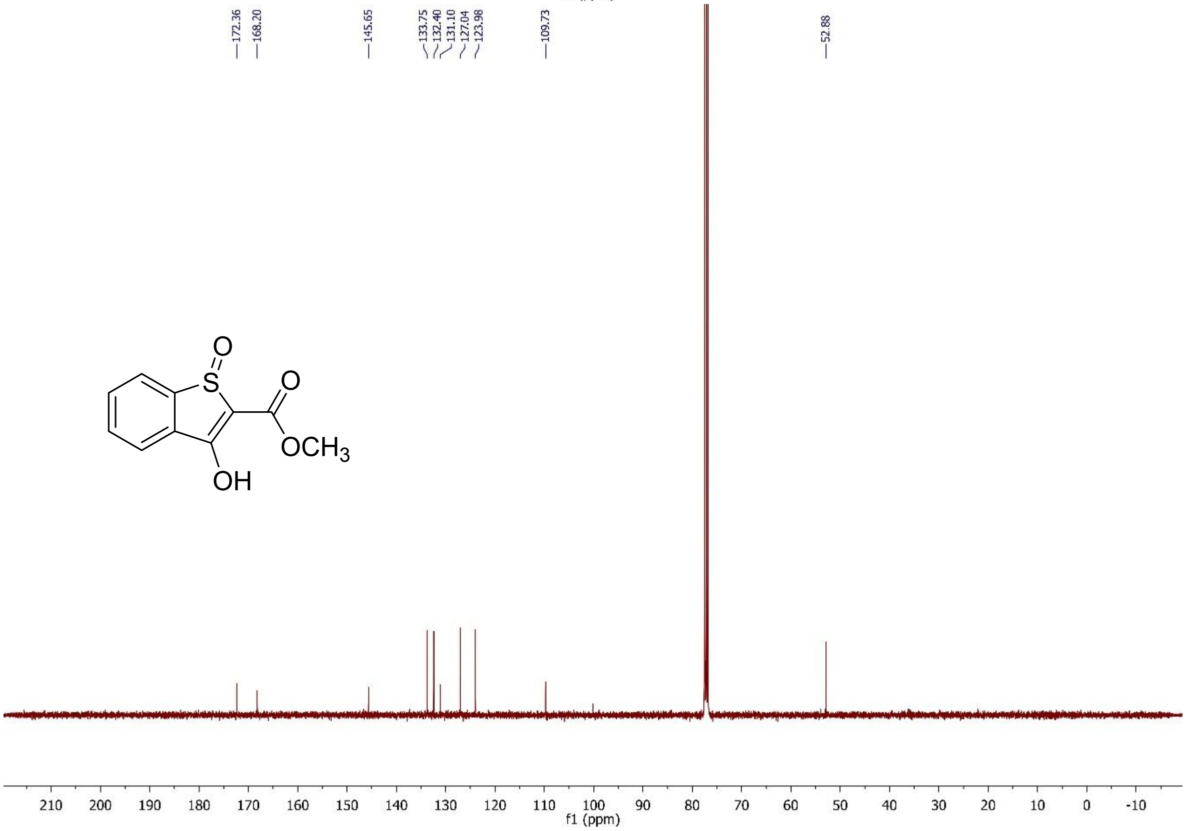
172.36
168.20
145.65
133.75
132.40
131.10
127.04
123.98
109.73
52.88
O
S
O
OCH3
OH
210 200 190 180 170 160 150 140 130 120 110 100 90 80 70 60 50 40 30 20 10 0 -10
f1 (ppm)

Compound 3e

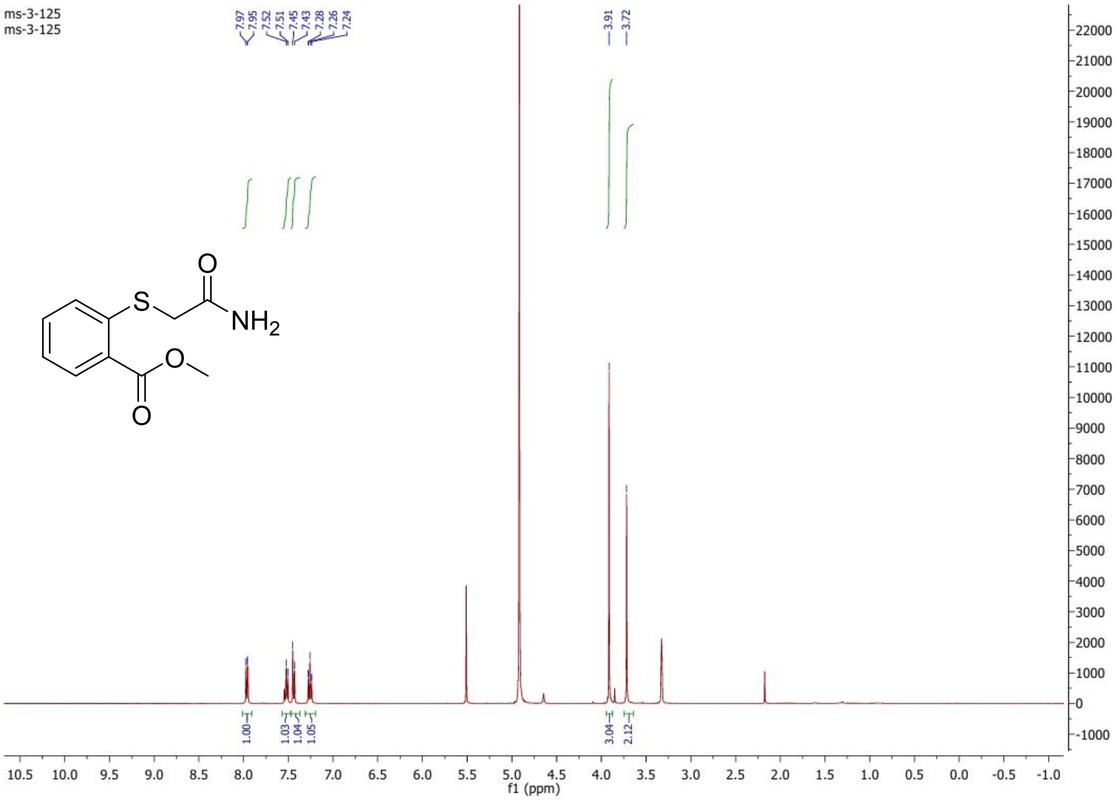
ms-3-125
ms-3-125
7.97
7.95
7.52
7.51
7.45
7.43
7.28
7.26
7.24
3.91
3.72
S
O
NH2
O
O
1.00
1.03
1.04
1.05
3.04
2.12
f1 (ppm)

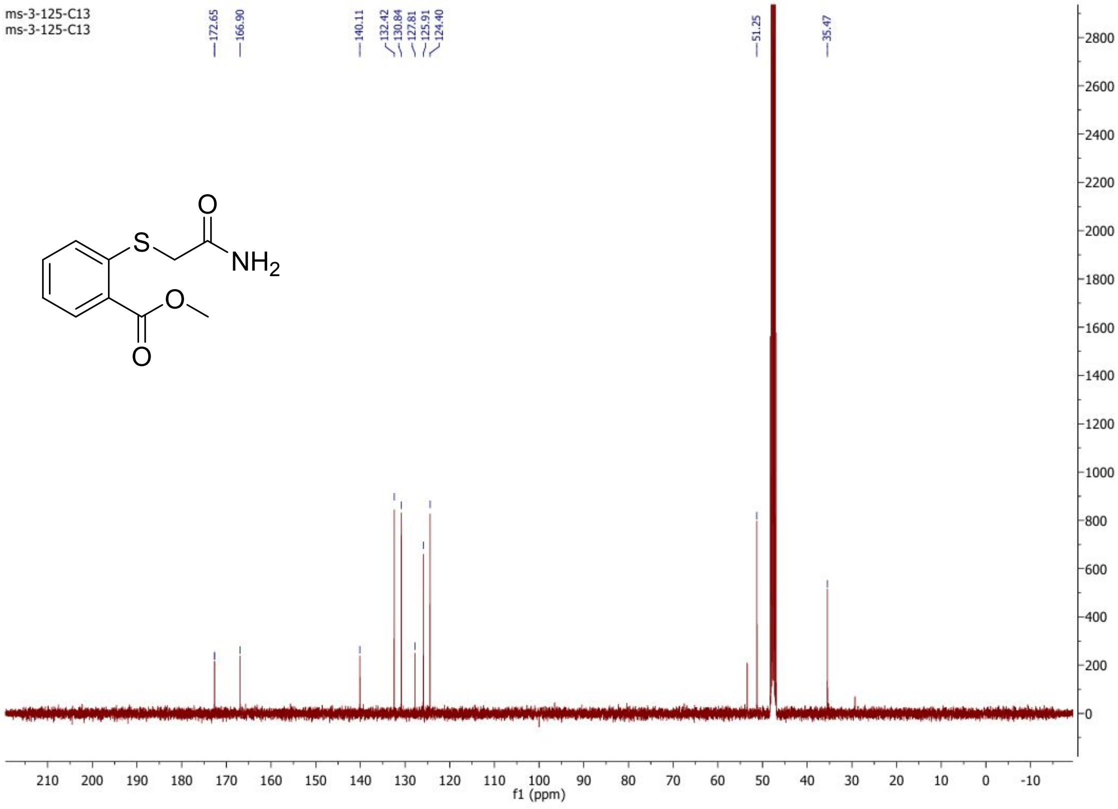
ms-3-125-C13
ms-3-125-C13
172.65
166.90
140.11
132.42
130.84
127.81
125.91
124.40
51.25
35.47
S
O
NH2
O
O
f1 (ppm)

Compound 4e

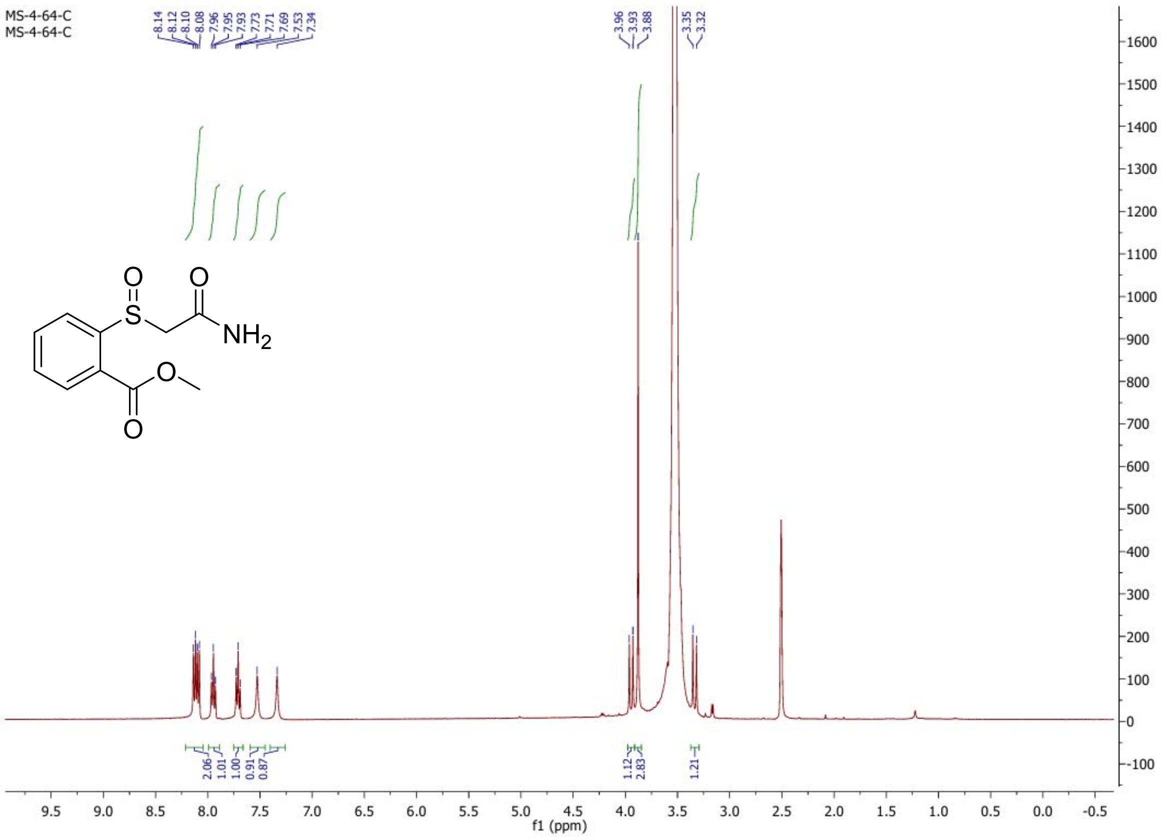
MS-4-64-C
MS-4-64-C
8.14
8.12
8.10
8.08
7.96
7.95
7.93
7.73
7.71
7.69
7.53
7.34
3.96
3.93
3.88
3.35
3.32
2.06
1.01
1.00
0.91
0.87
1.12
2.83
1.21
f1 (ppm)

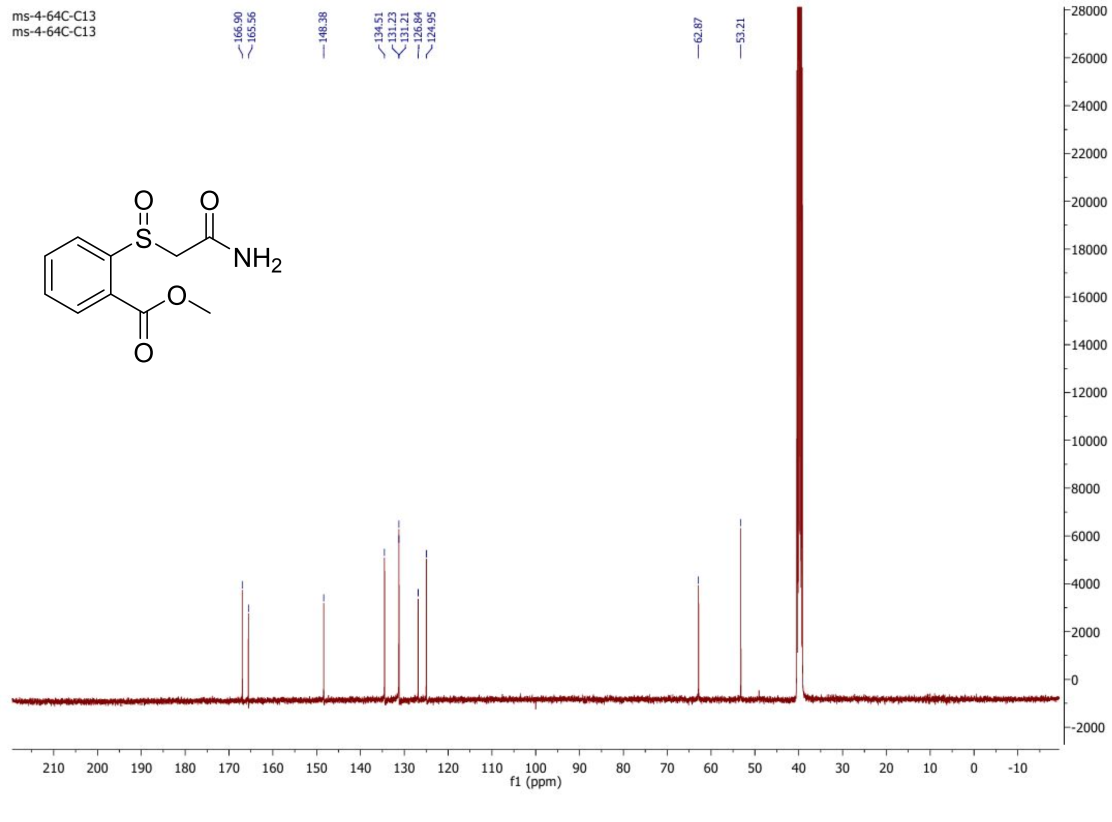
ms-4-64C-C13
ms-4-64C-C13
166.90
165.56
148.38
134.51
131.23
131.21
126.84
124.95
62.87
53.21
f1 (ppm)

Compound 5e

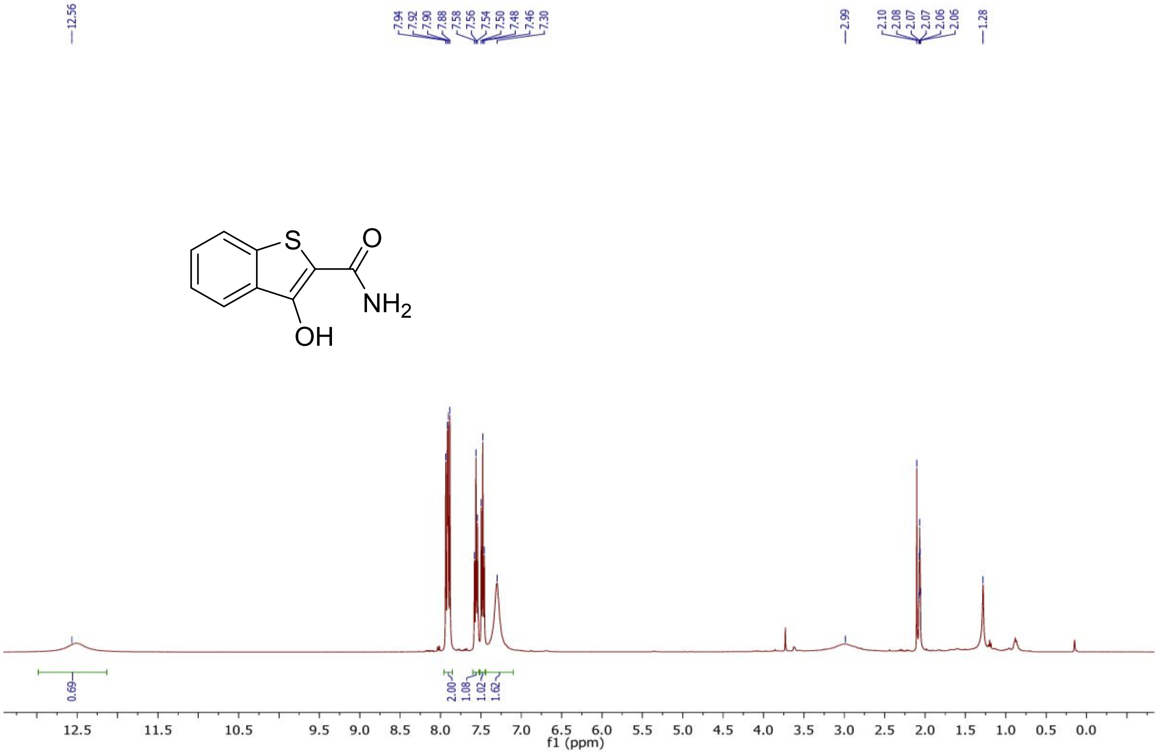

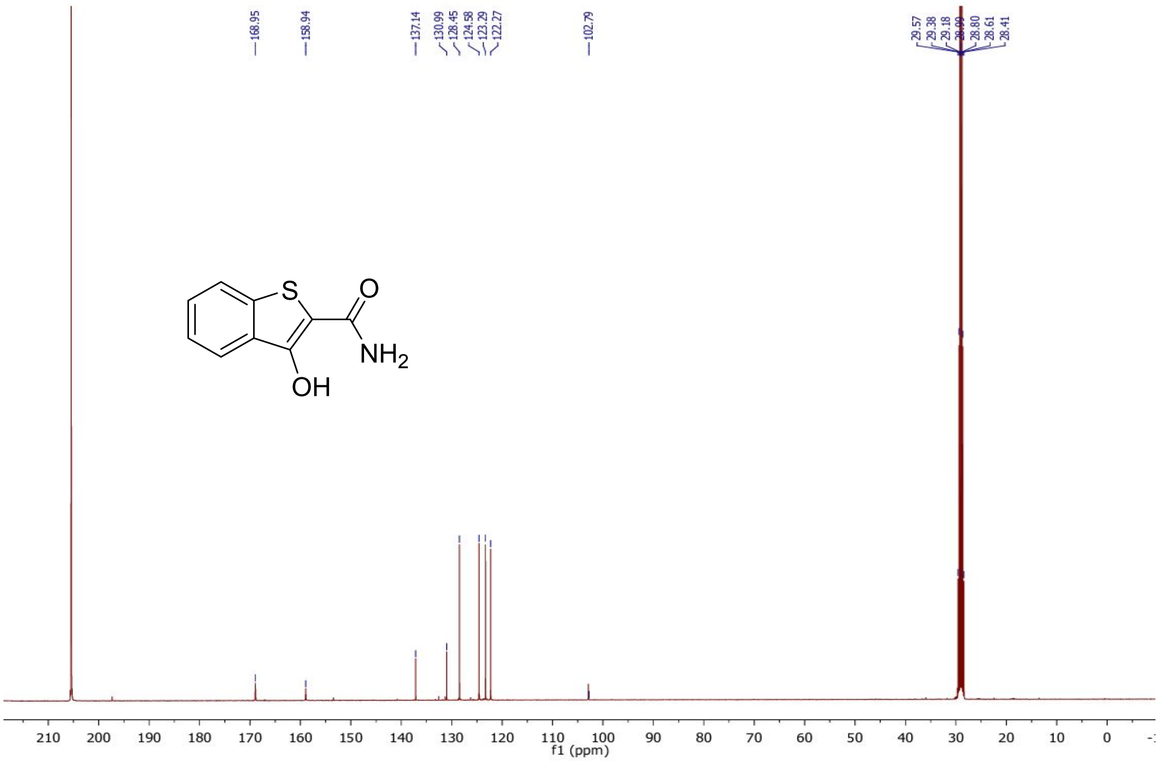

Compound 6e

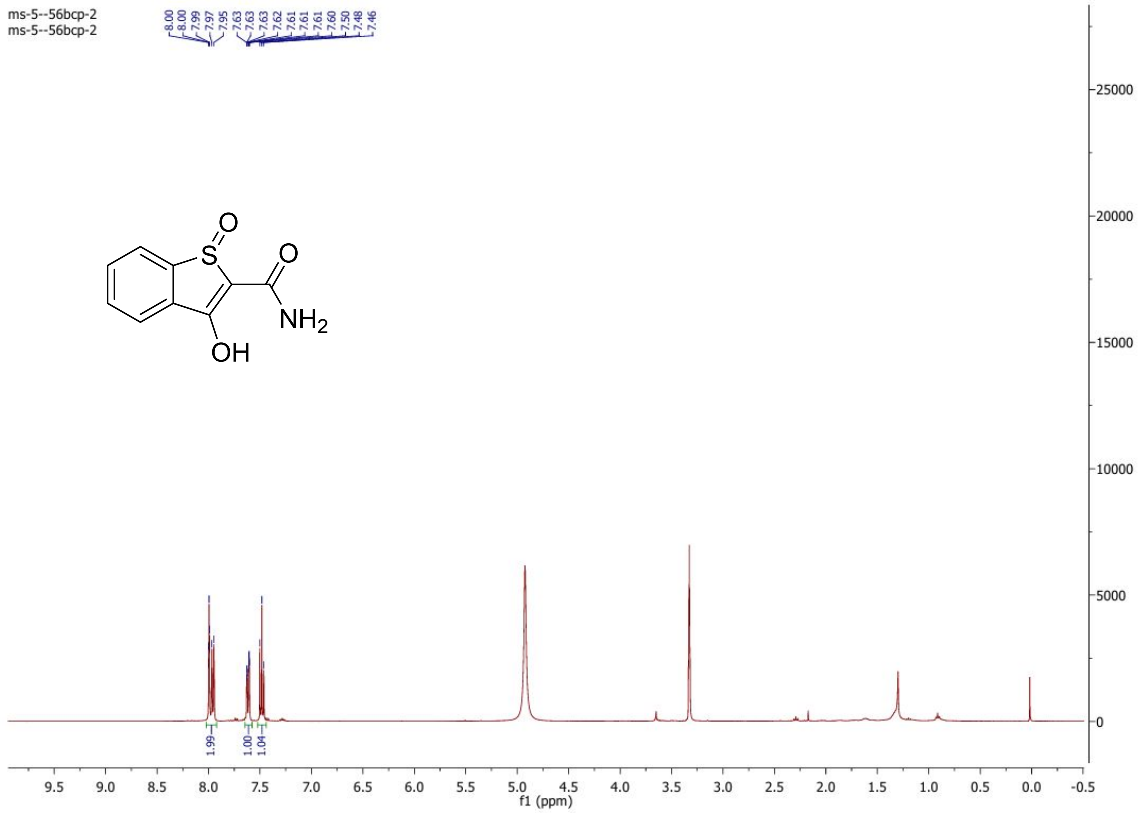
ms-5--56bcp-2
ms-5--56bcp-2
8.00
8.00
7.99
7.97
7.95
7.63
7.63
7.63
7.62
7.61
7.61
7.61
7.60
7.50
7.48
7.46
O
S
O
NH2
OH
1.99
1.00
1.04
f1 (ppm)

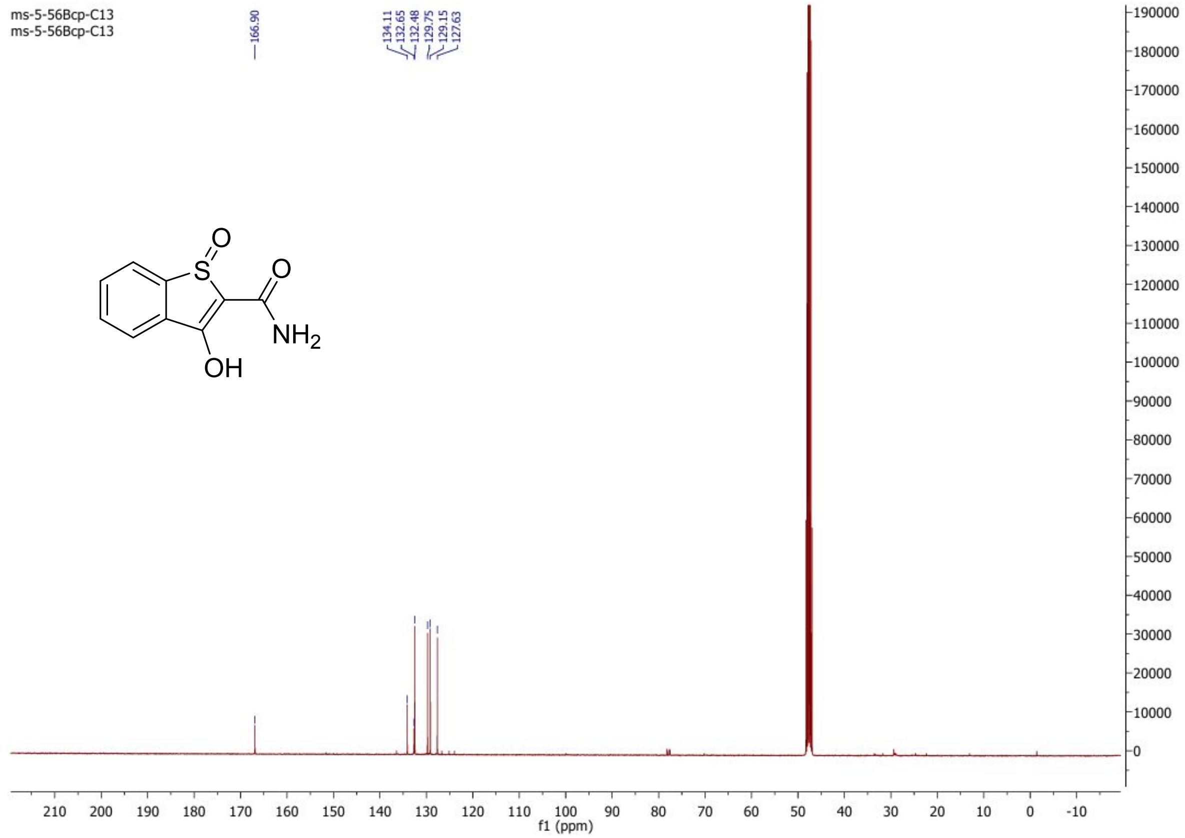
ms-5-56Bcp-C13
ms-5-56Bcp-C13
166.90
134.11
132.65
132.48
129.75
129.15
127.63
O
S
O
NH2
OH
f1 (ppm)

Compound 3f

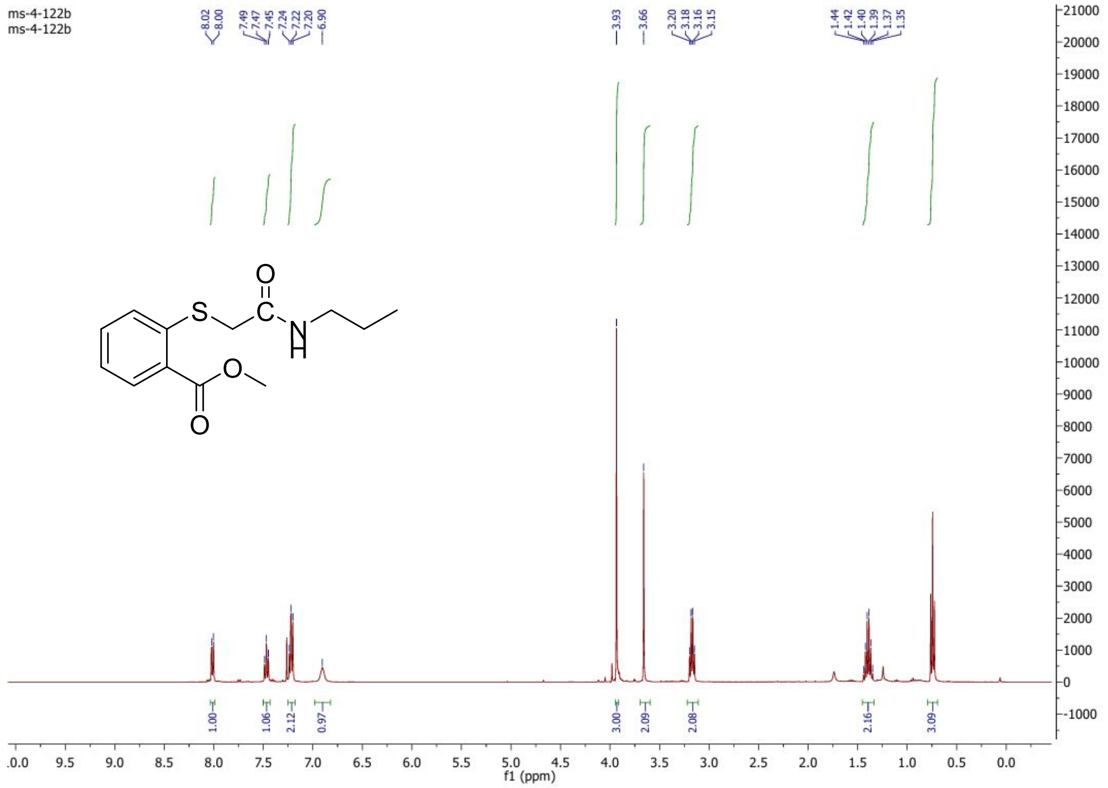

ms-4-122b
ms-4-122b
8.02
8.00
7.49
7.47
7.45
7.24
7.22
7.20
6.90
3.93
3.66
3.20
3.18
3.16
3.15
1.44
1.42
1.40
1.39
1.37
1.35
1.00
1.06
2.12
0.97
3.00
2.09
2.08
2.16
3.09
f1 (ppm)

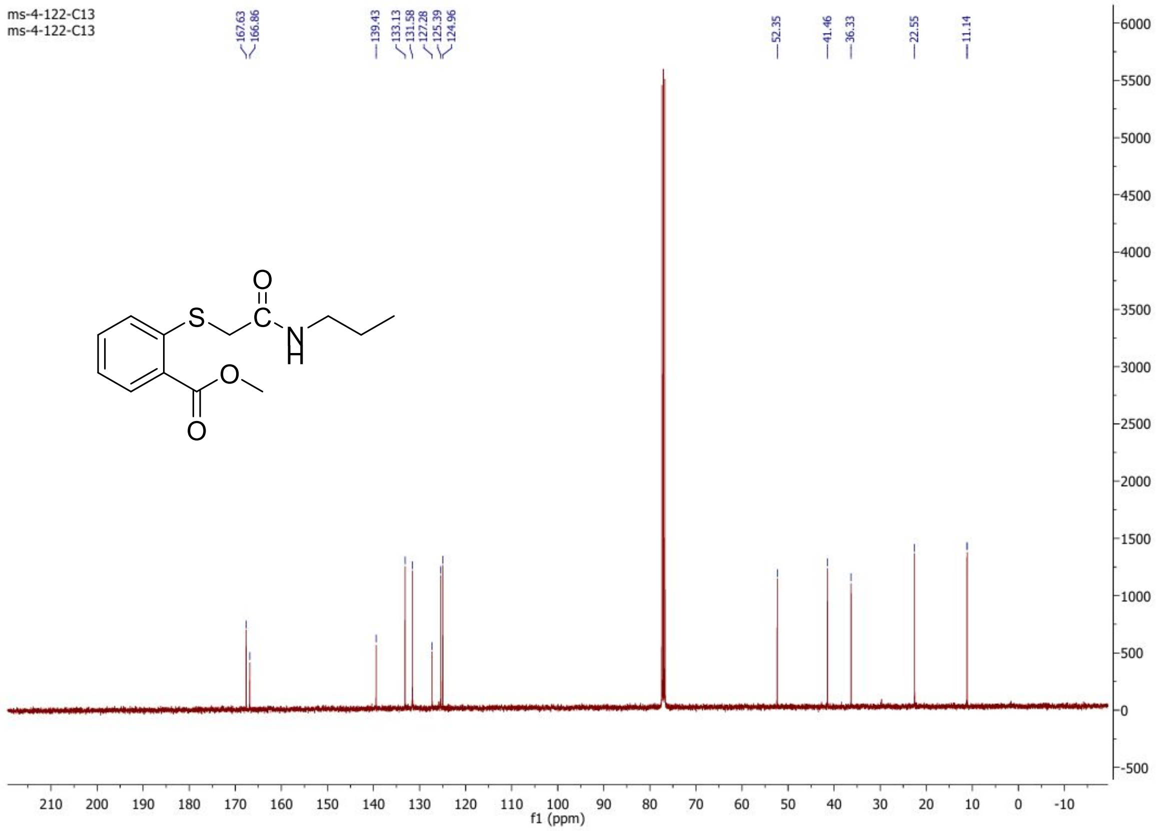

ms-4-122-C13
ms-4-122-C13
167.63
166.86
139.43
133.13
131.58
127.28
125.39
124.96
52.35
41.46
36.33
22.55
11.14
f1 (ppm)

Compound 4f

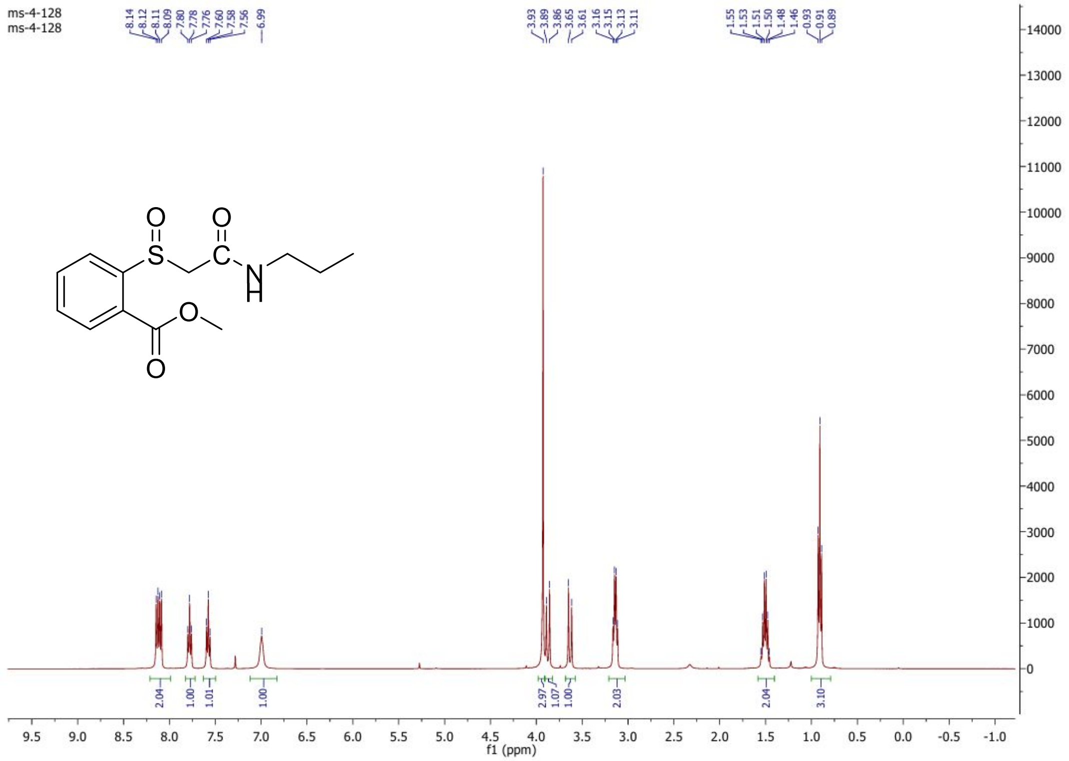

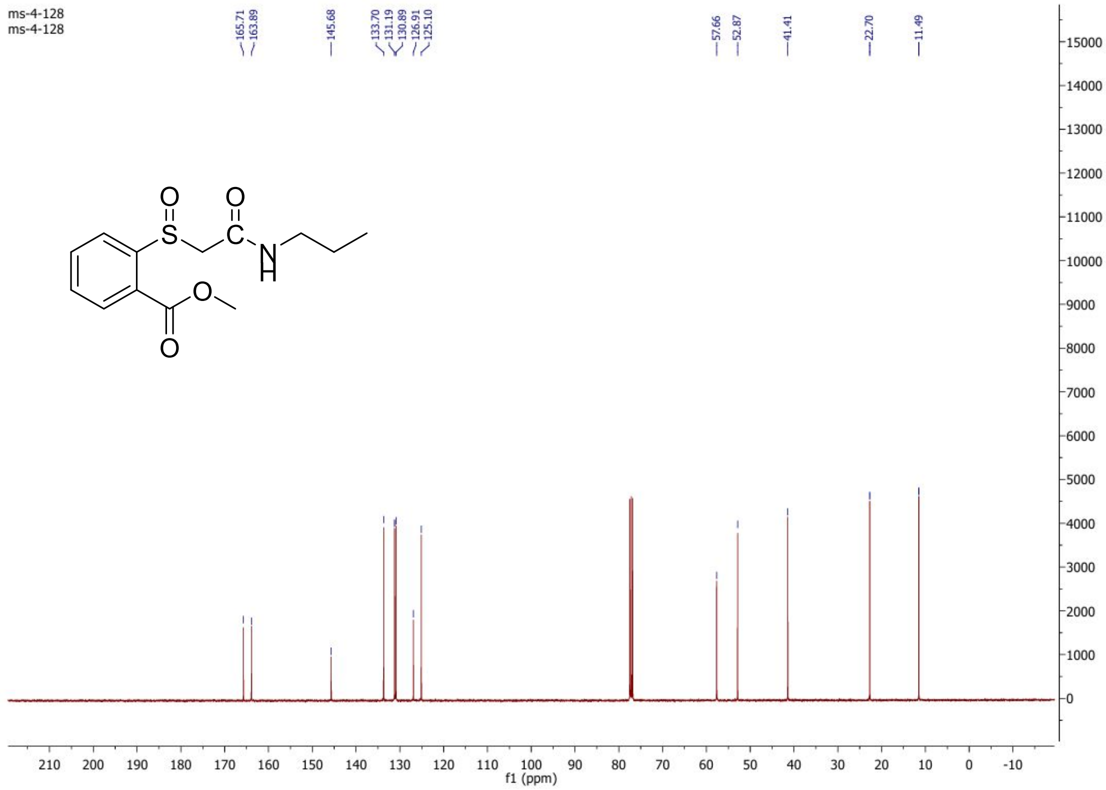

Compound 5f

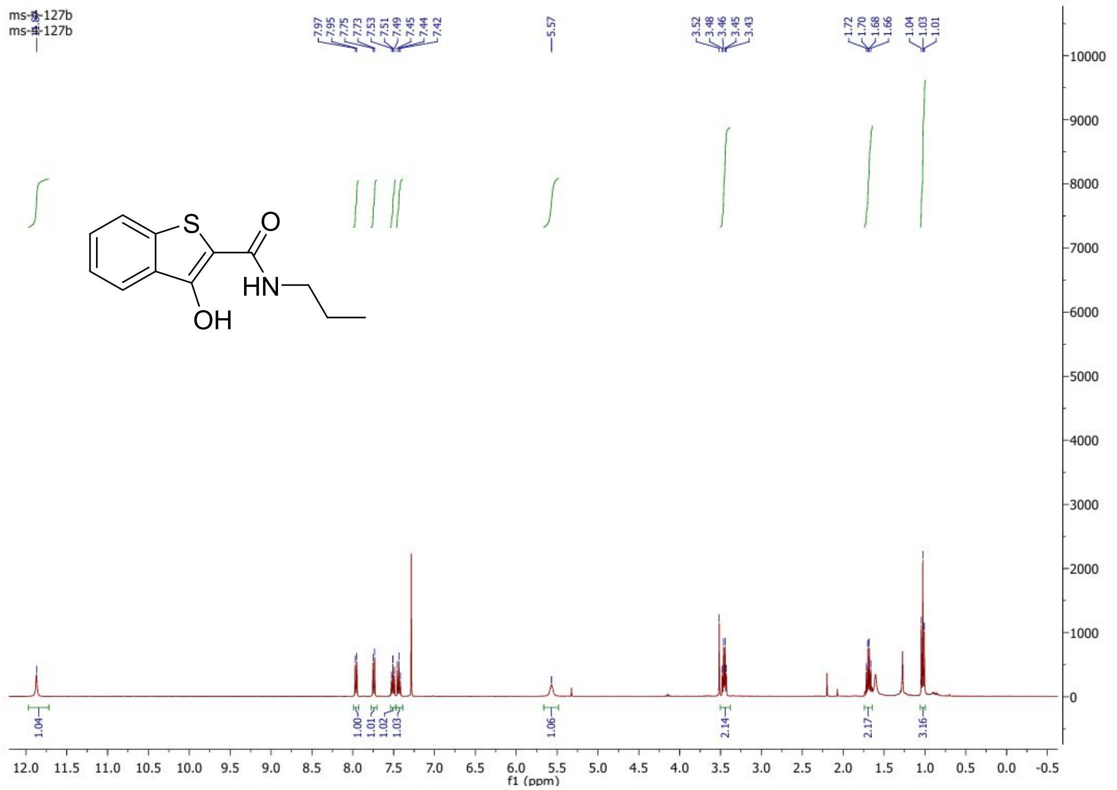
ms-4-127b
ms-4-127b
7.97 7.95 7.75 7.73 7.53 7.51 7.49 7.45 7.44 7.42
5.57
3.52 3.48 3.46 3.45 3.43
1.72 1.70 1.68 1.66 1.04 1.03 1.01
S O HN OH
1.04 1.00 1.01 1.02 1.03 1.06 2.14 2.17 3.16
f1 (ppm)

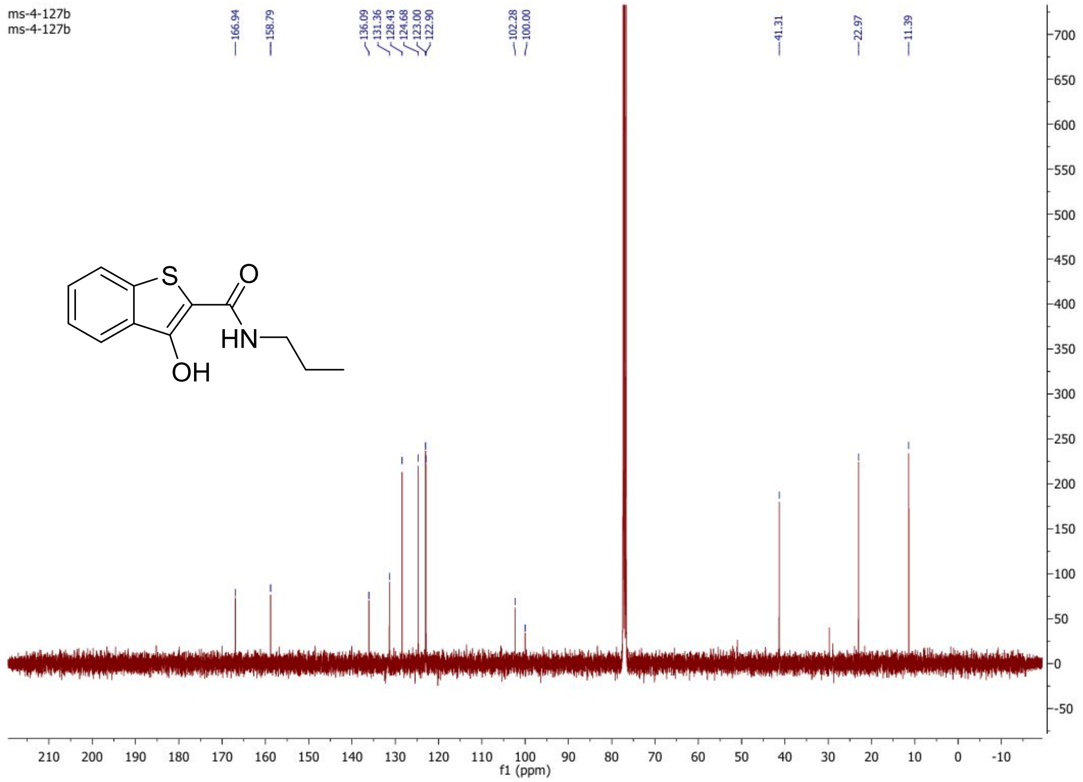
ms-4-127b
ms-4-127b
166.94 158.79
136.09 131.36 128.43 124.68 123.00 122.90
102.28 100.00
41.31 22.97 11.39
S O HN OH
f1 (ppm)

Compound 6f

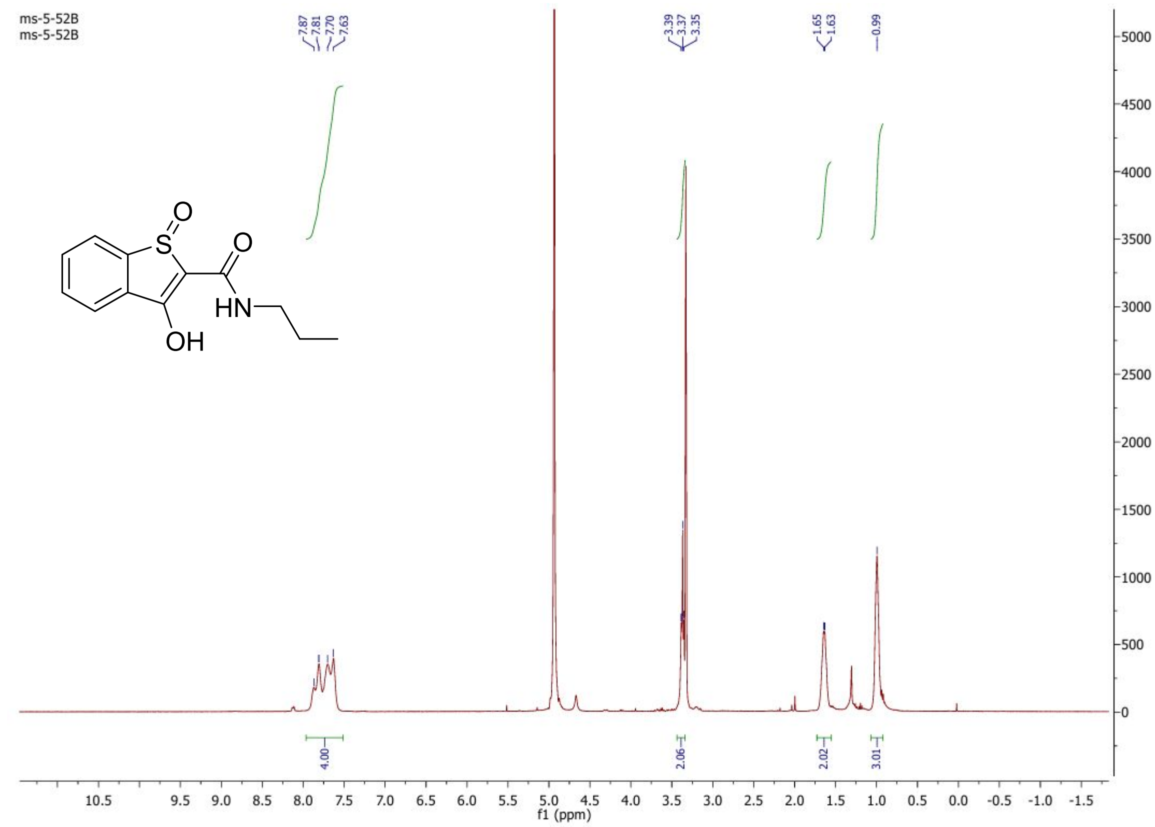
ms-5-52B
ms-5-52B
7.87
7.81
7.70
7.63
3.39
3.37
3.35
1.65
1.63
0.99
4.00
2.06
2.02
3.01
O
S
O
HN
OH
f1 (ppm)

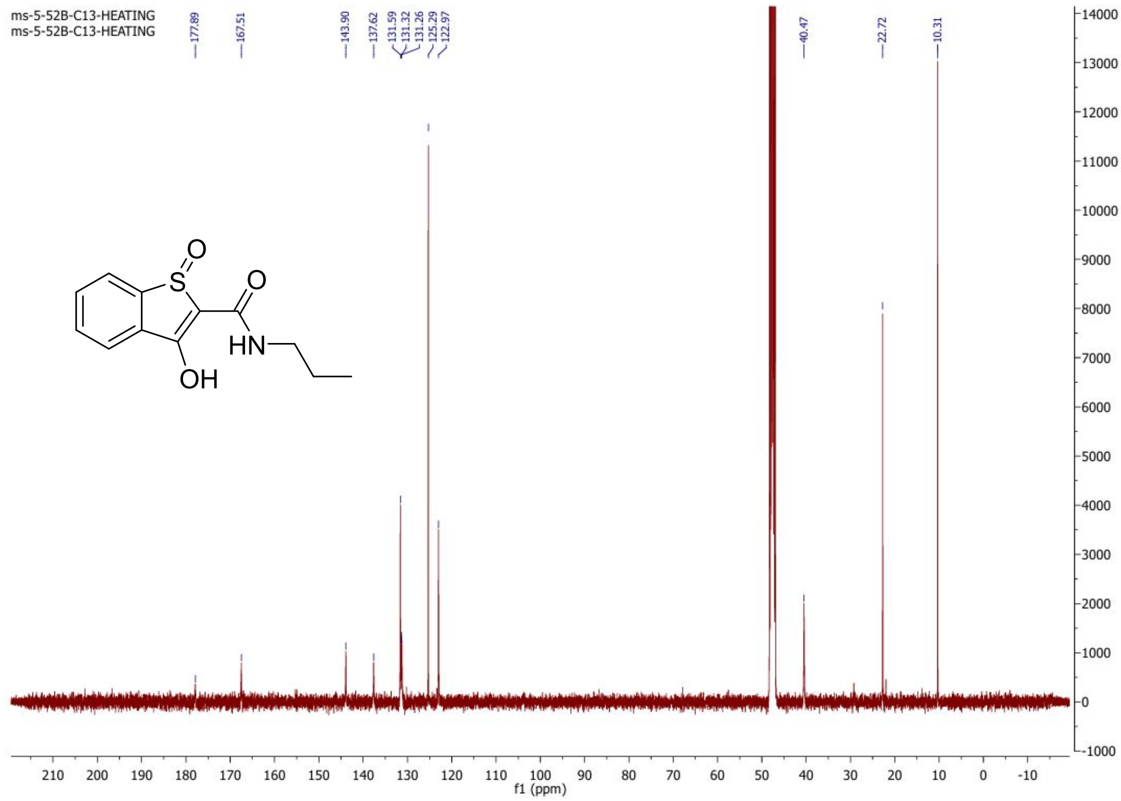
ms-5-52B-C13-HEATING
ms-5-52B-C13-HEATING
177.89
167.51
143.90
137.62
131.59
131.32
131.26
125.29
122.97
40.47
22.72
10.31
O
S
O
HN
OH
f1 (ppm)

Compound 3g

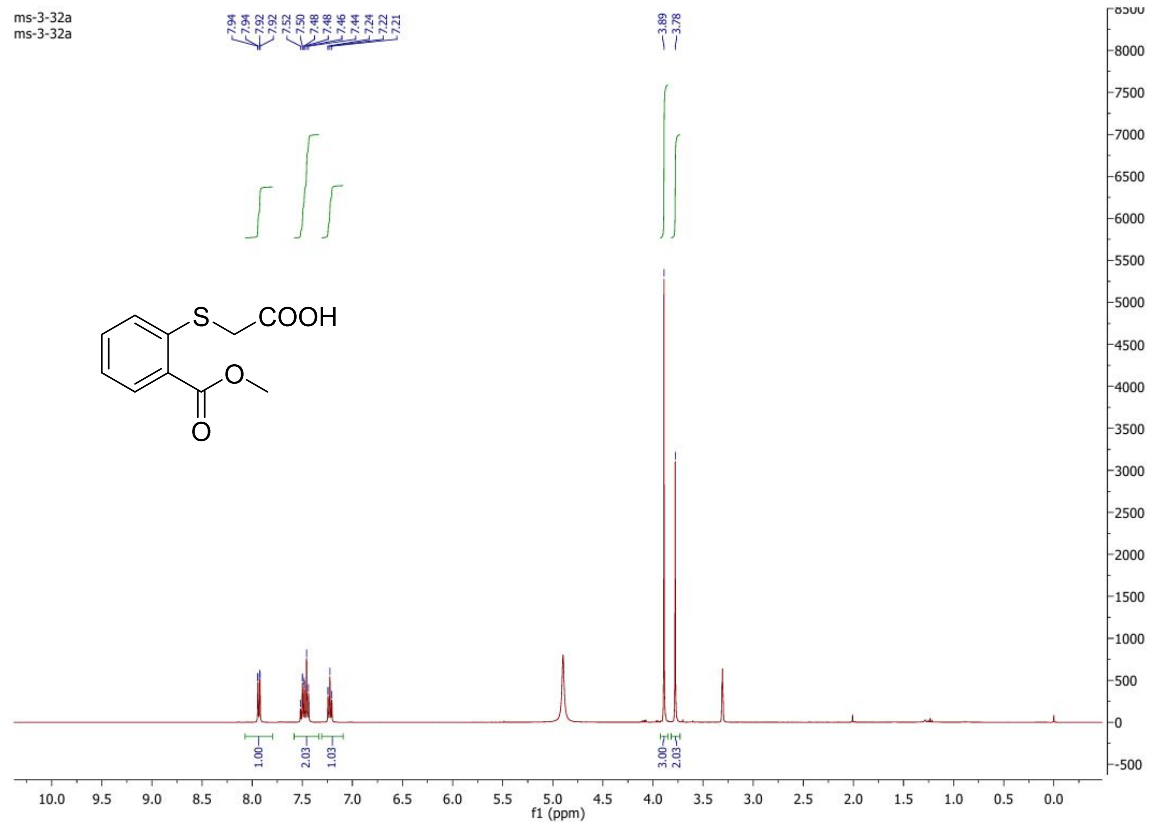
ms-3-32a
ms-3-32a
7.94
7.94
7.92
7.92
7.52
7.50
7.48
7.48
7.46
7.44
7.24
7.22
7.21
3.89
3.78
S
COOH
O
O
1.00
2.03
1.03
3.00
2.03
f1 (ppm)

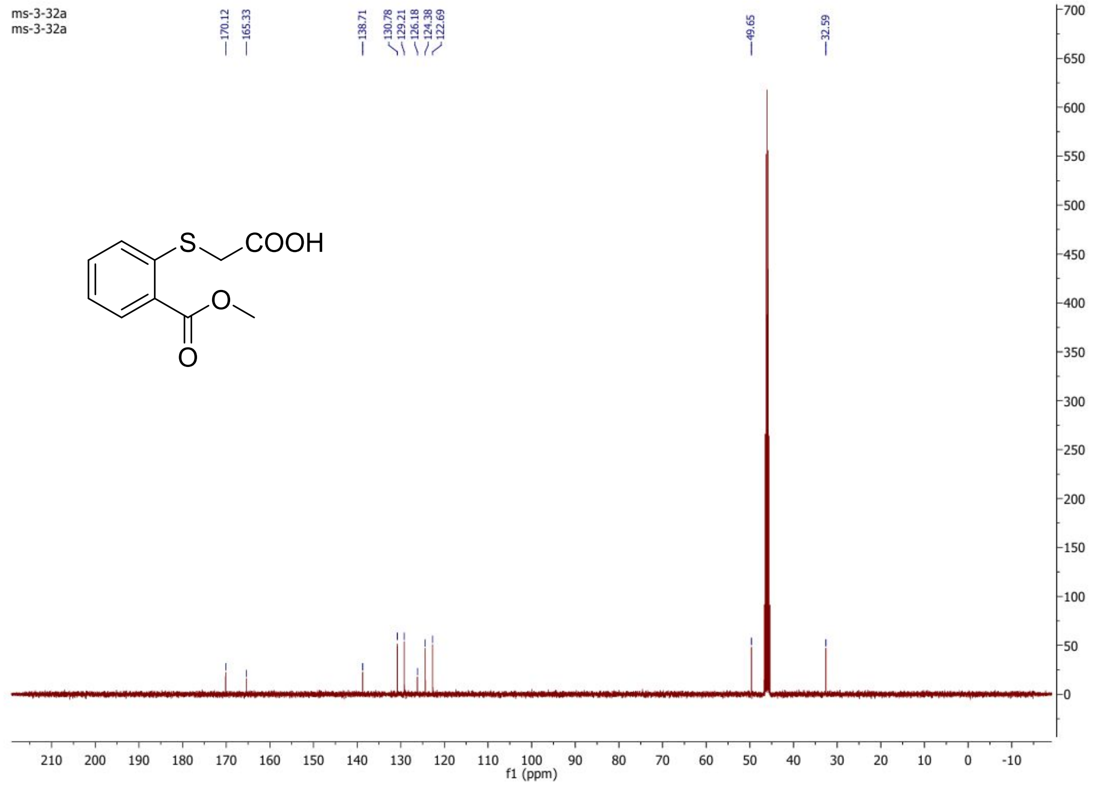
ms-3-32a
ms-3-32a
170.12
165.33
138.71
130.78
129.21
126.18
124.38
122.69
49.65
32.59
S
COOH
O
O
f1 (ppm)

Compound 4g

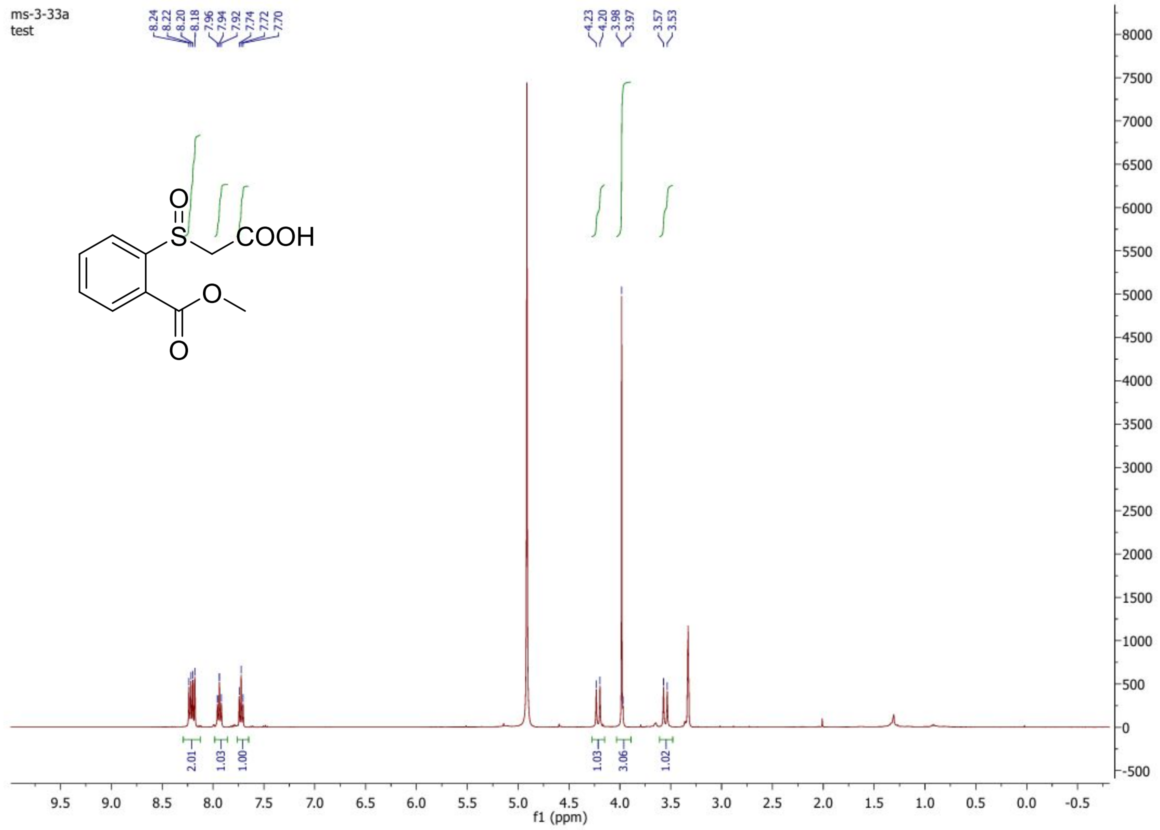

ms-3-33a
test
8.24 8.22 8.20 8.18 7.96 7.94 7.92 7.74 7.72 7.70
4.23 4.20 3.98 3.97 3.57 3.53
COOH
2.01 1.03 1.00 1.03 3.06 1.02
f1 (ppm)

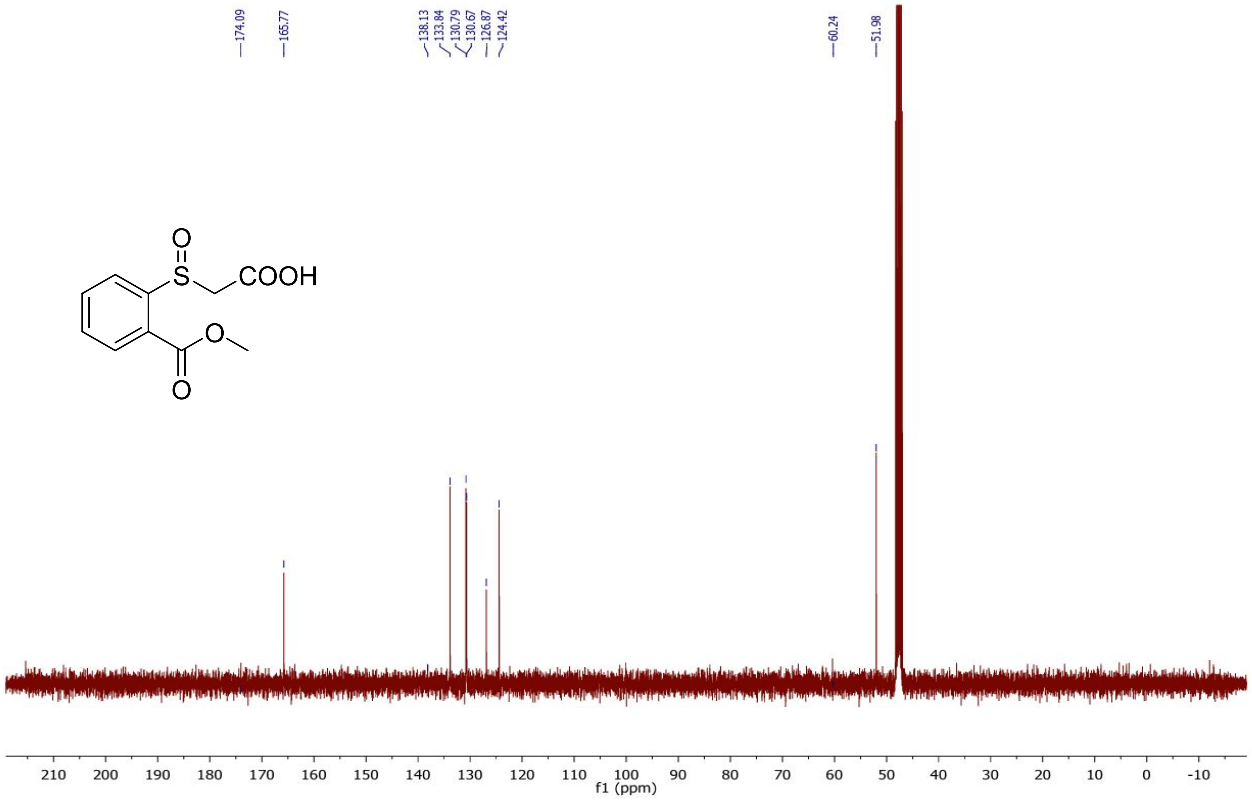

174.09 165.77
138.13 133.84 130.79 130.67 126.87 124.42
60.24 51.98
COOH
f1 (ppm)

Compound 11a

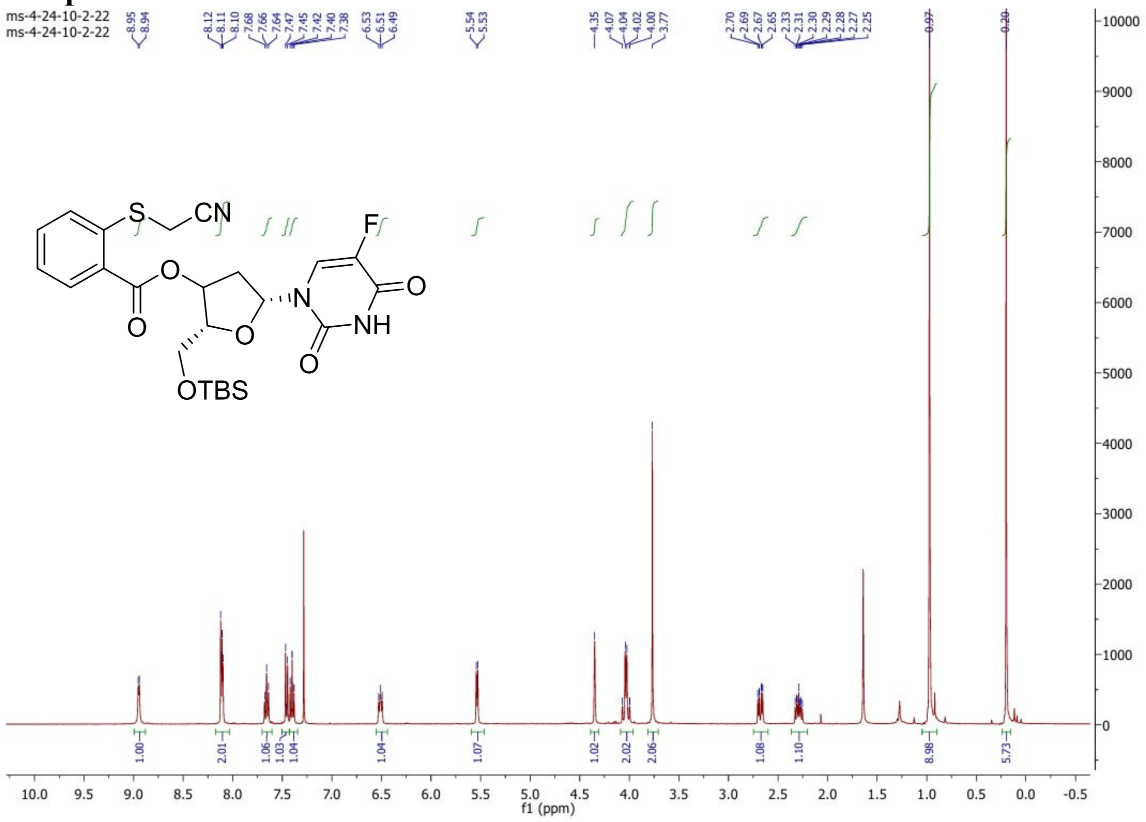

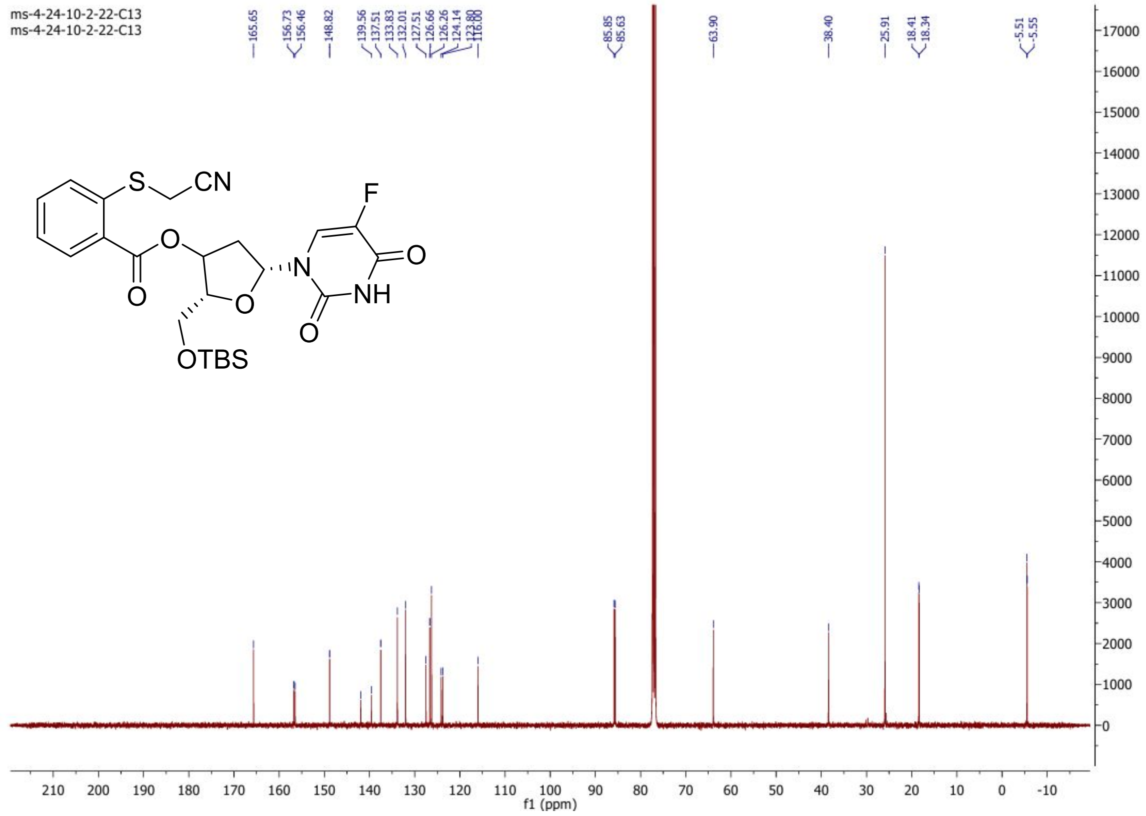

Compound 12a

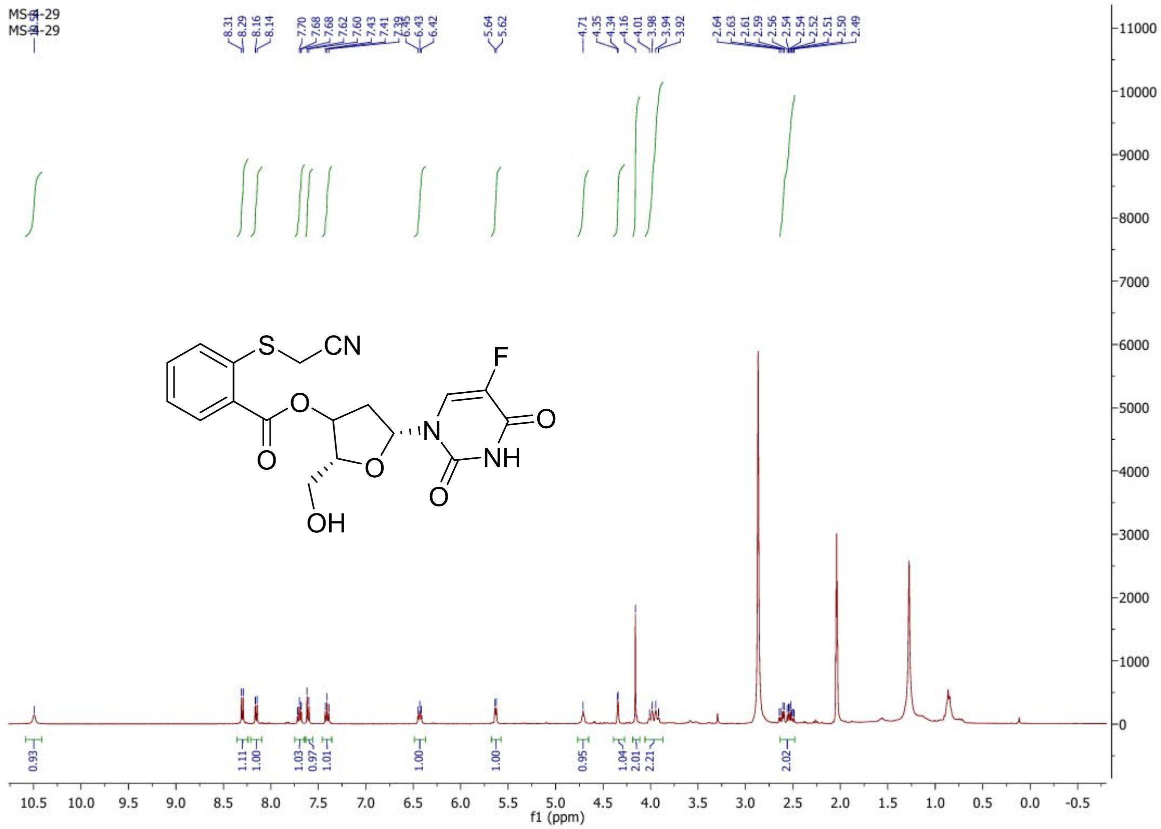

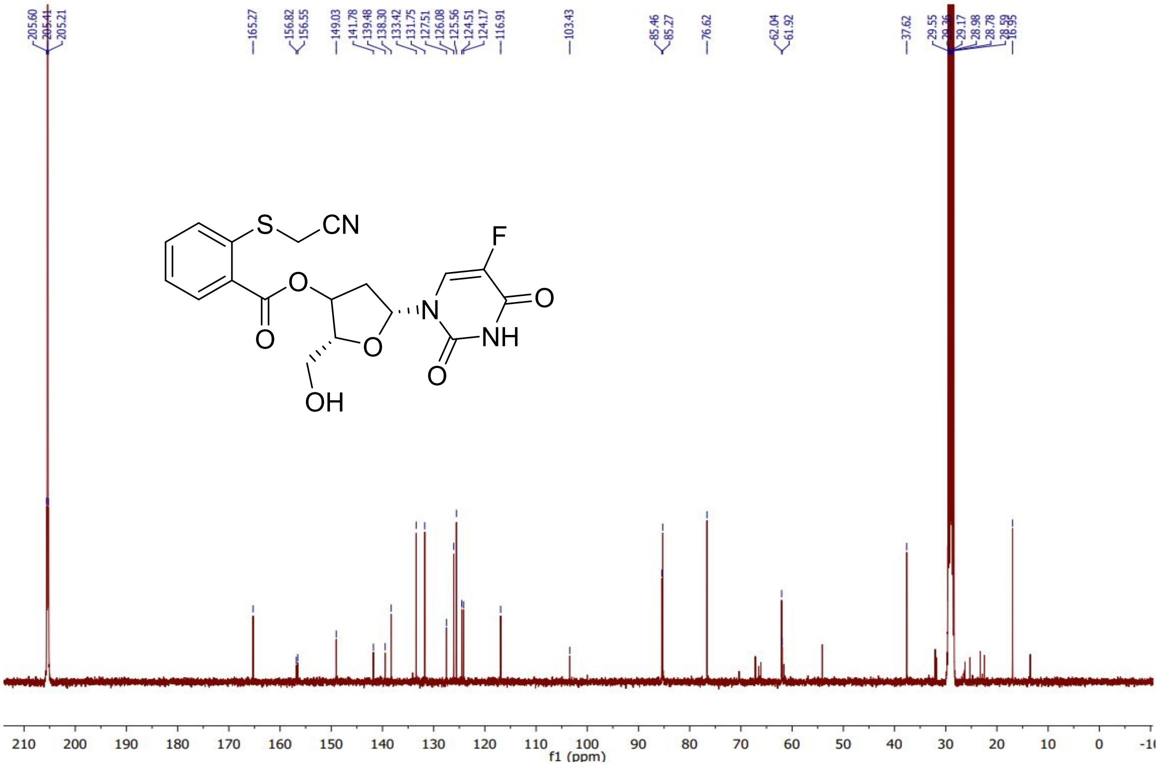

Compound 11d

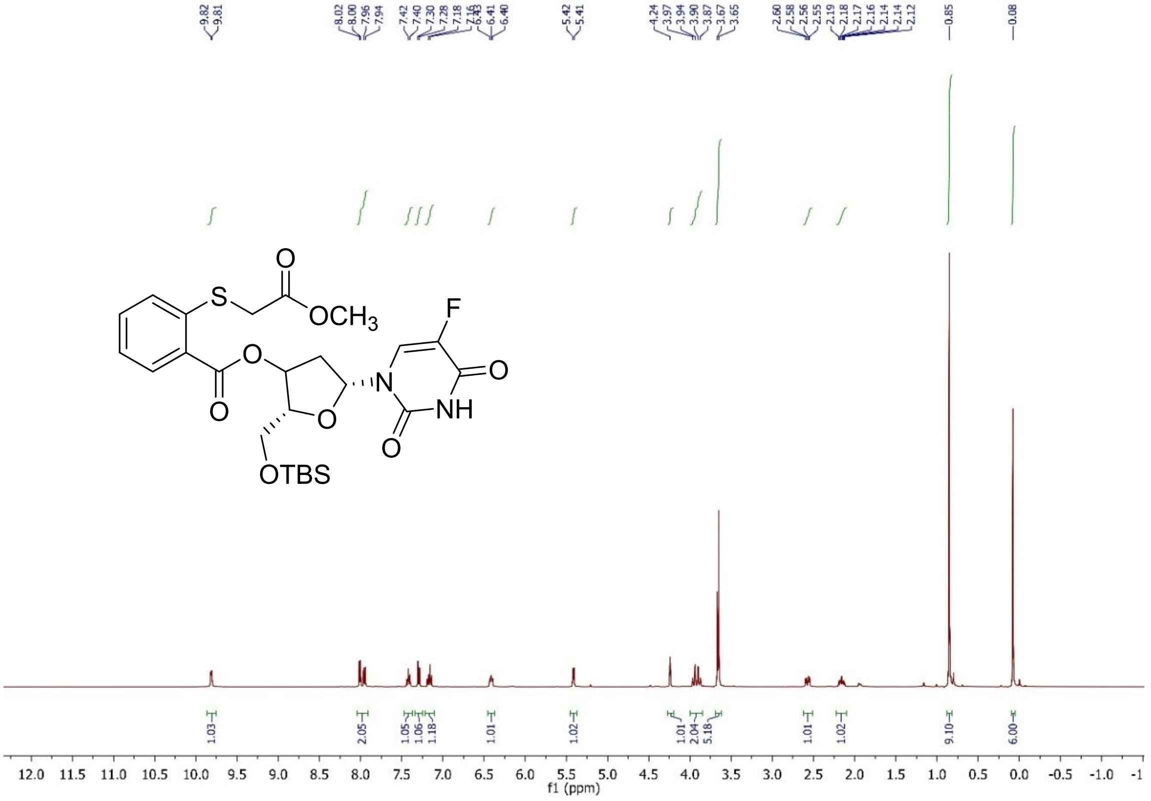

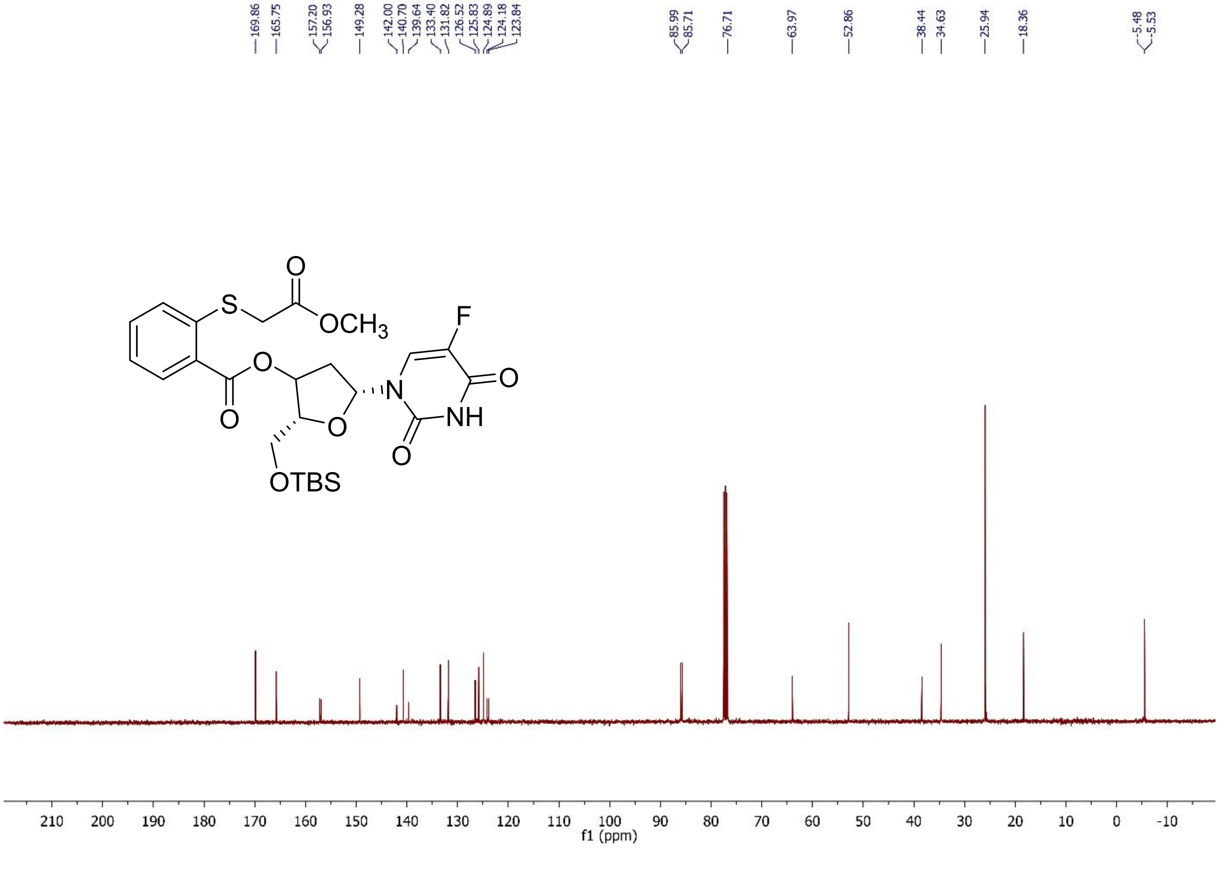

Compound 12d

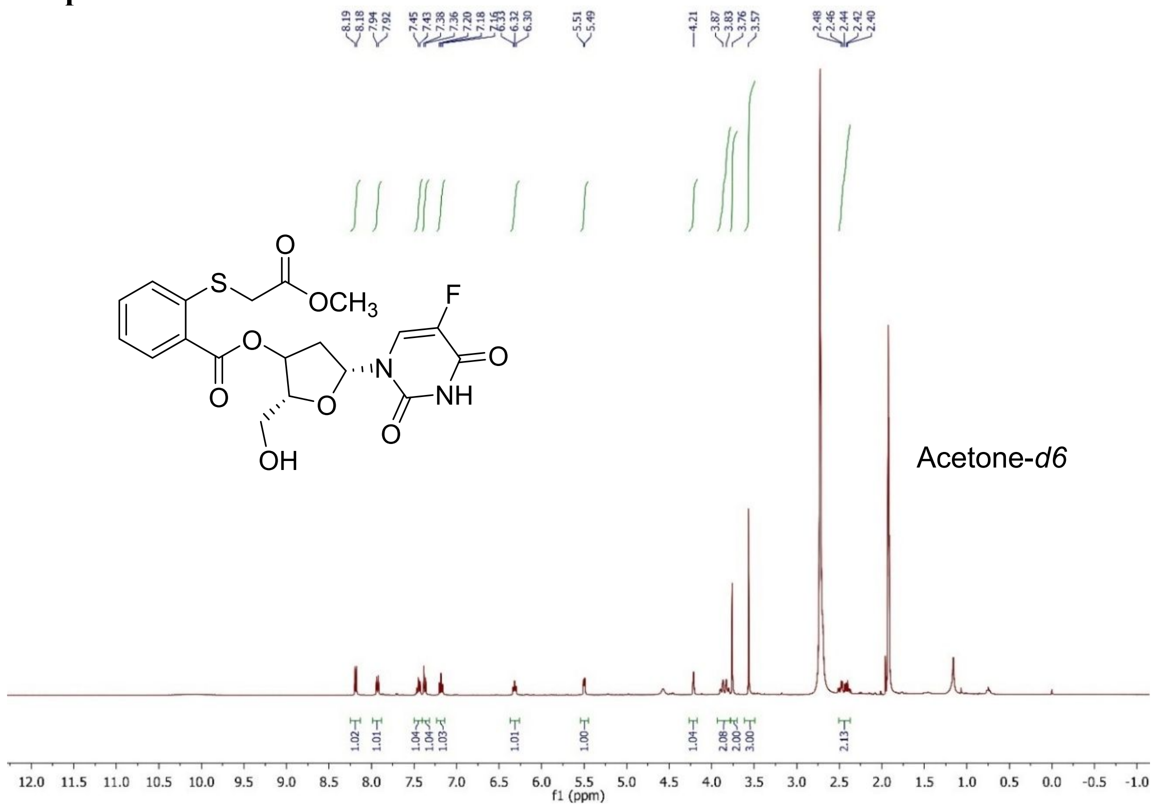

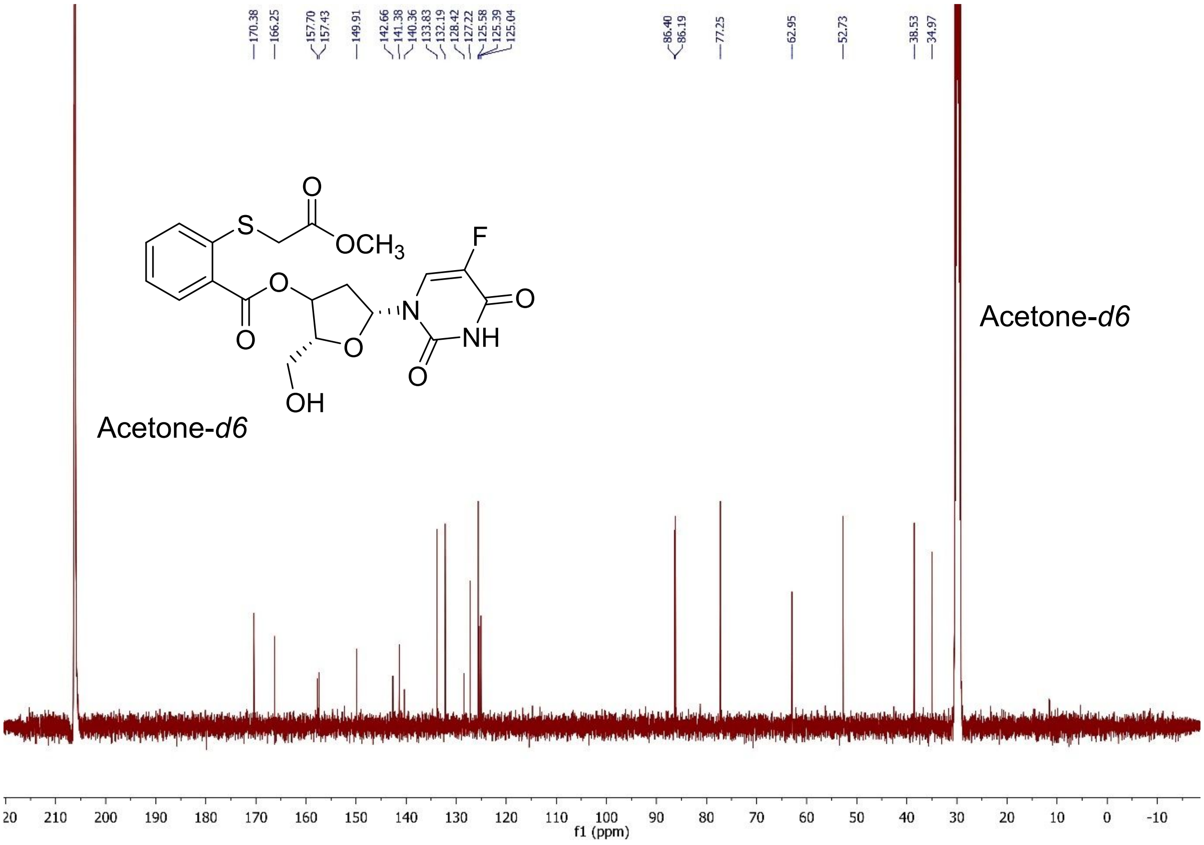

Compound 11e

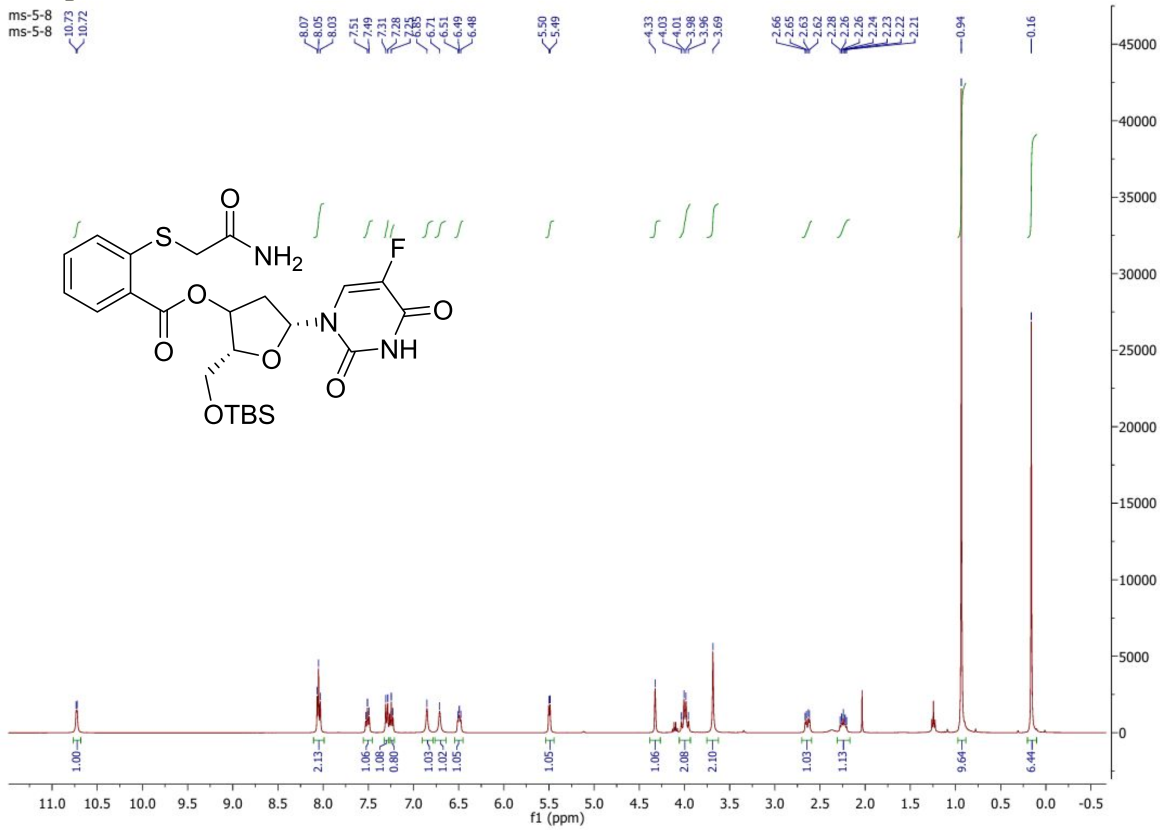

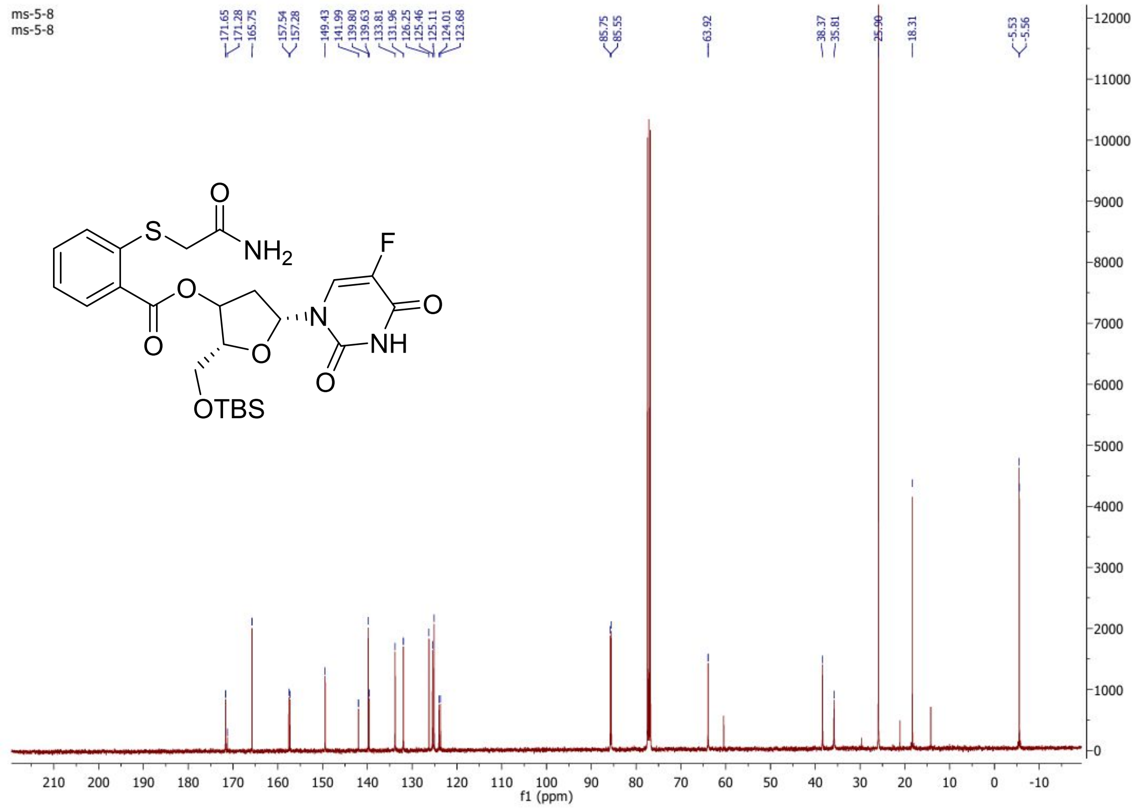

Compound 12e

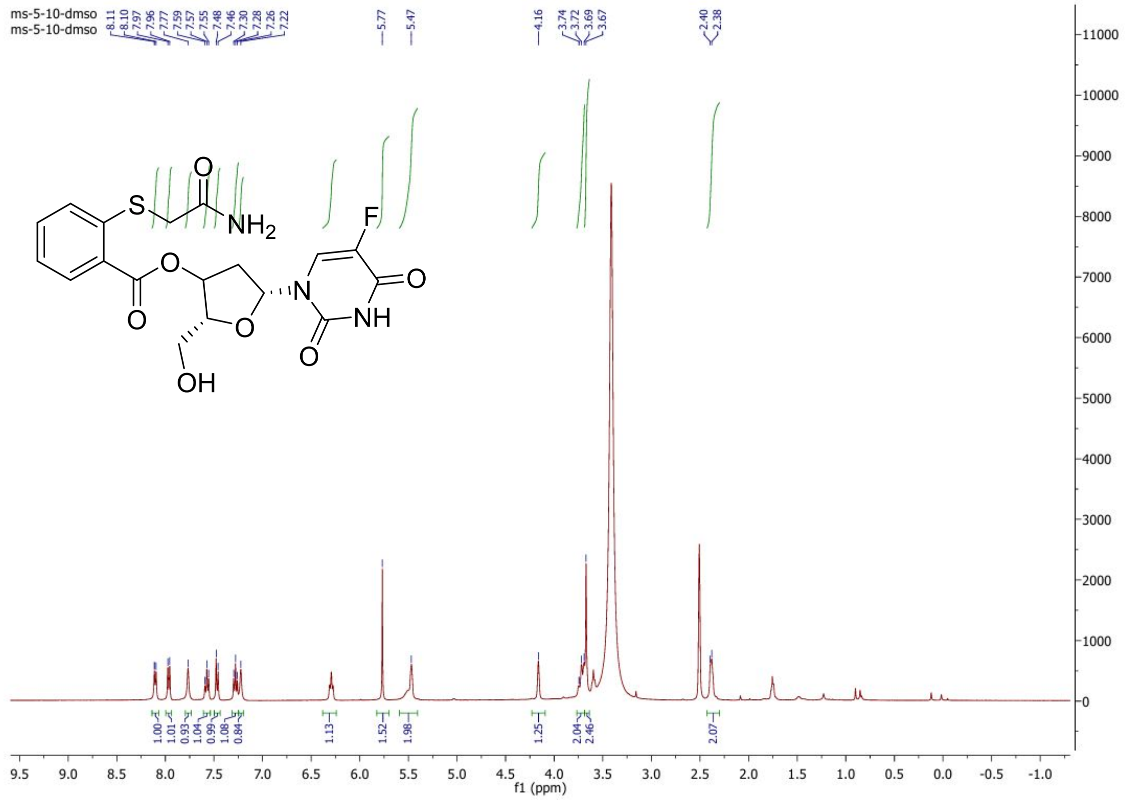

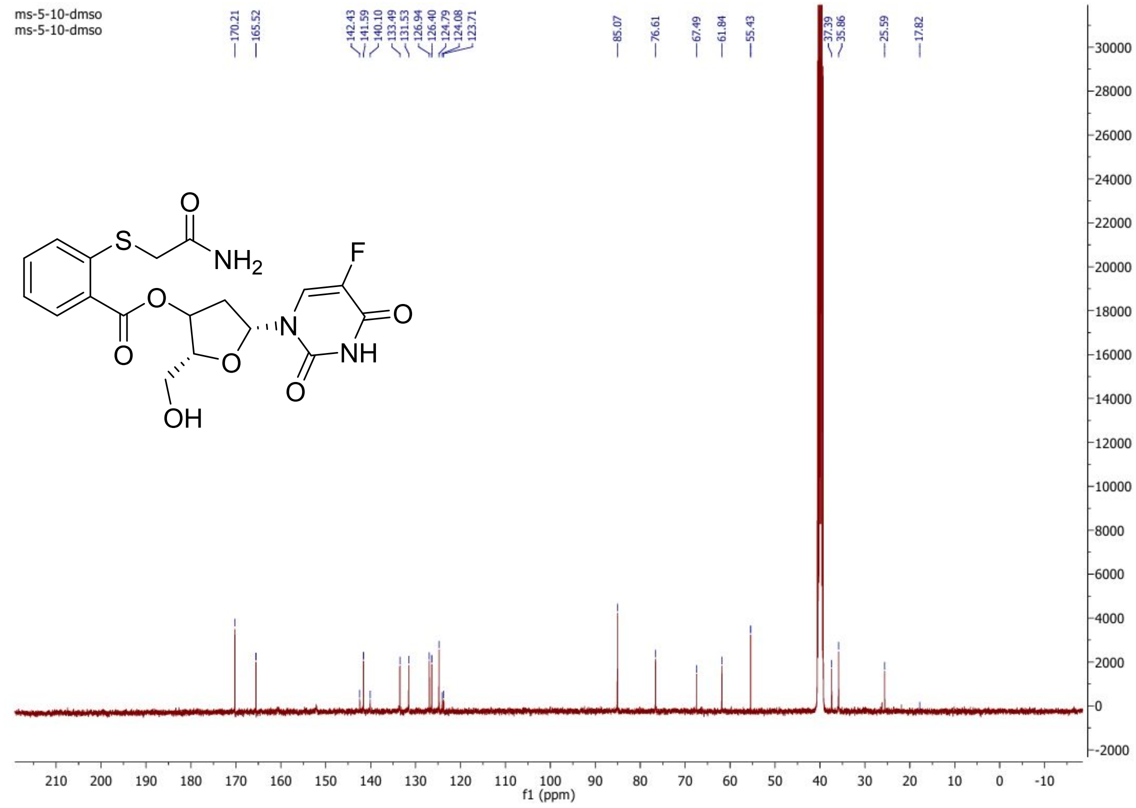

Compound 11f

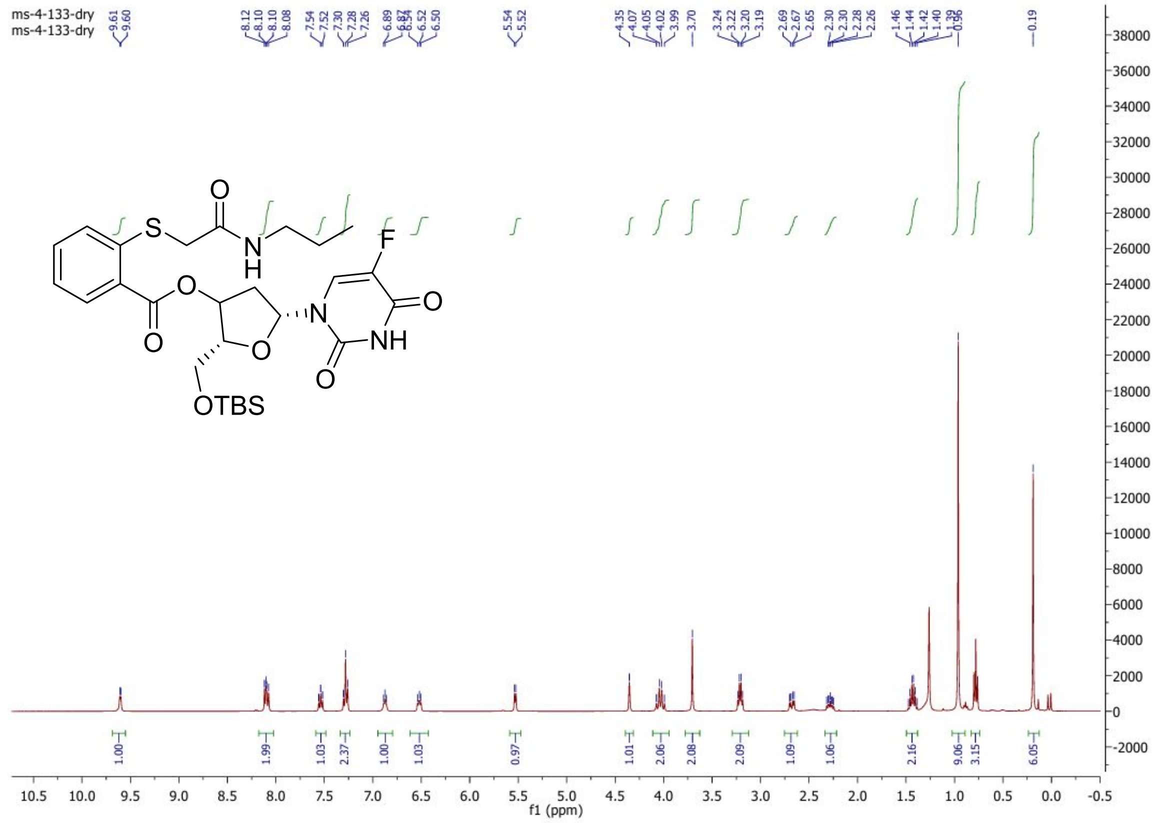

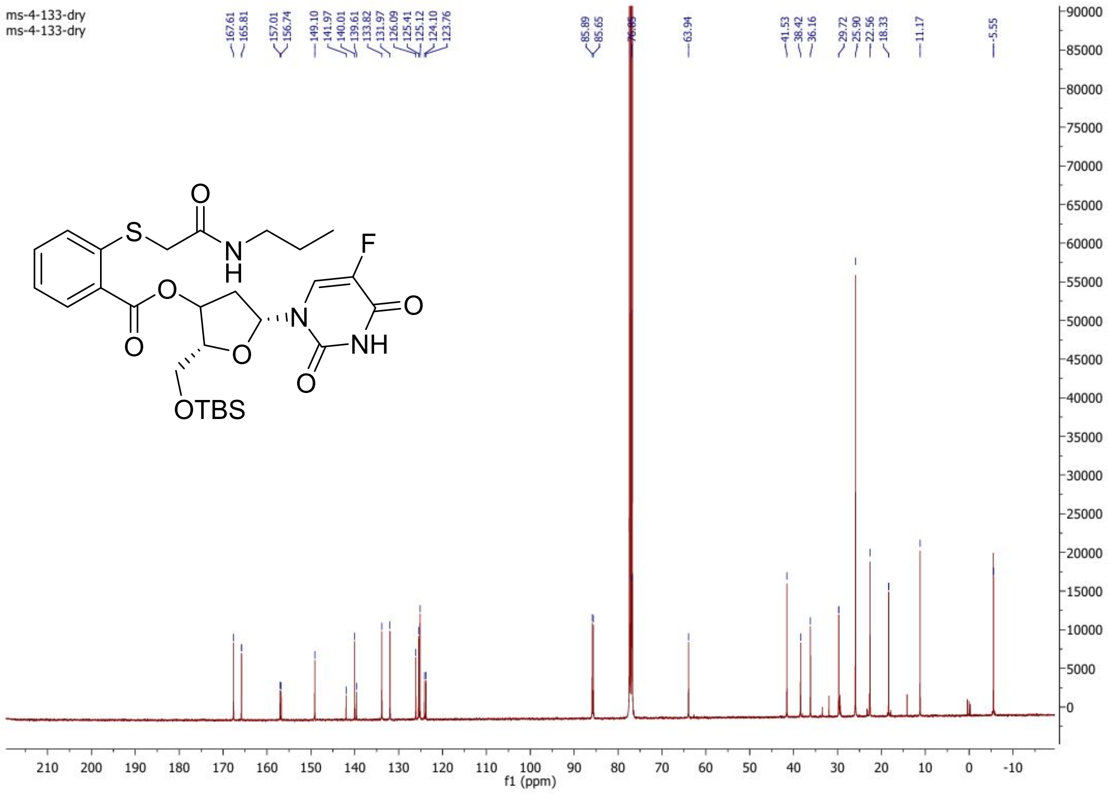

Compound 12f

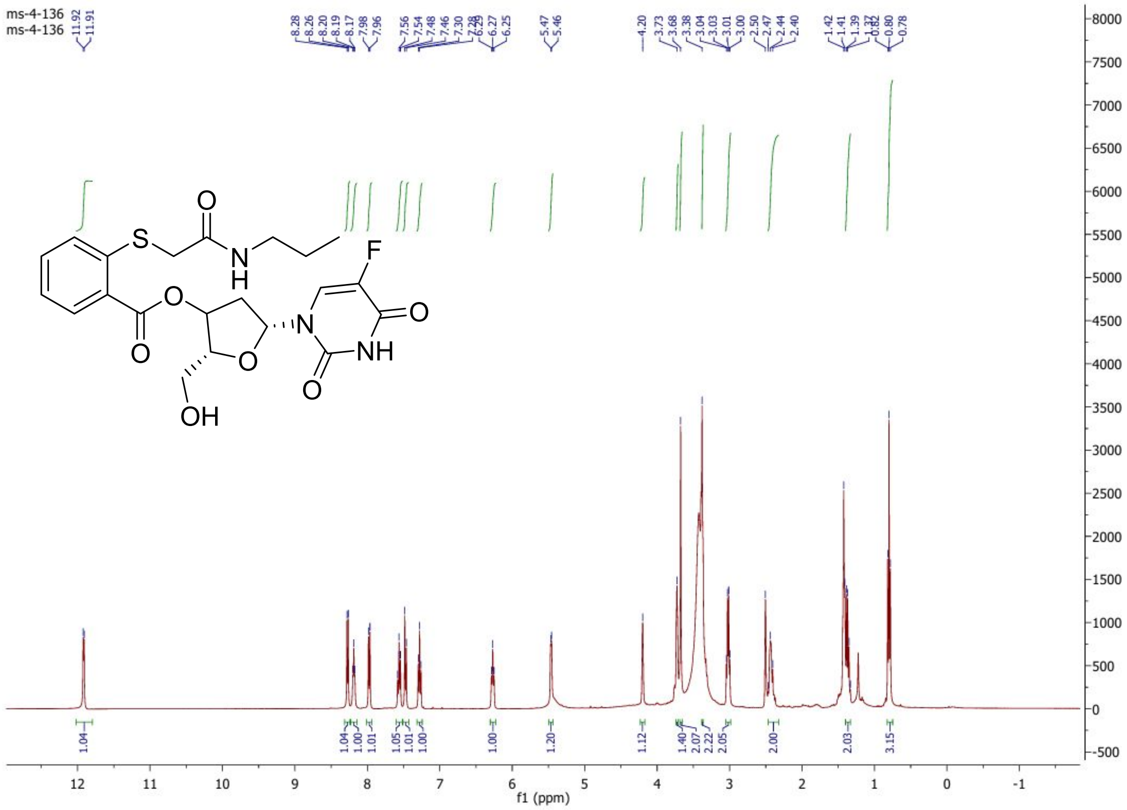

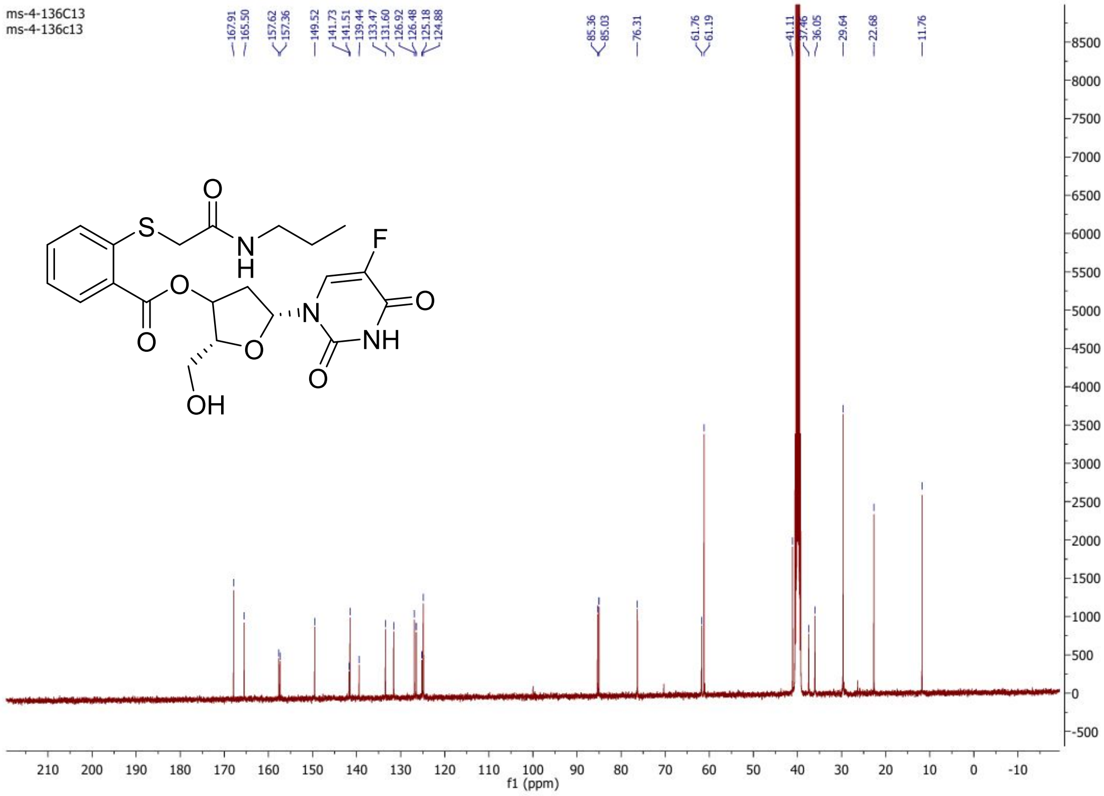

Compound 14e

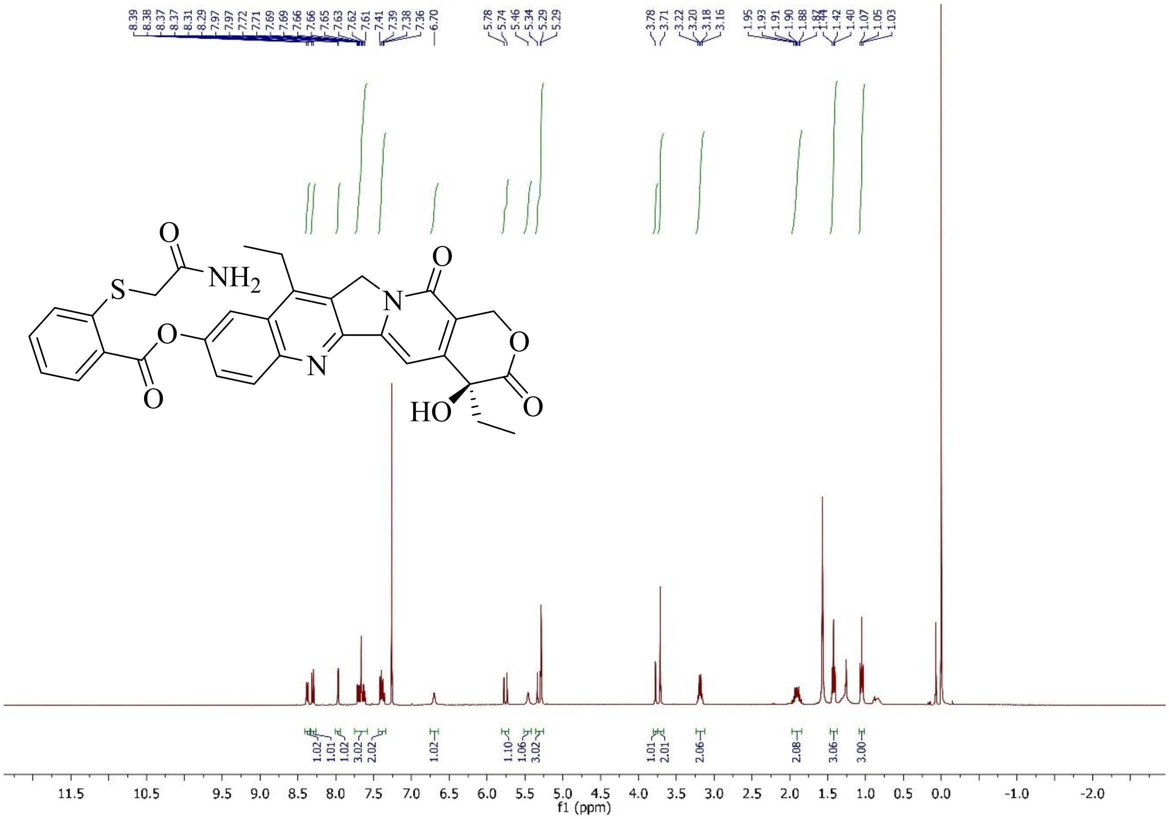

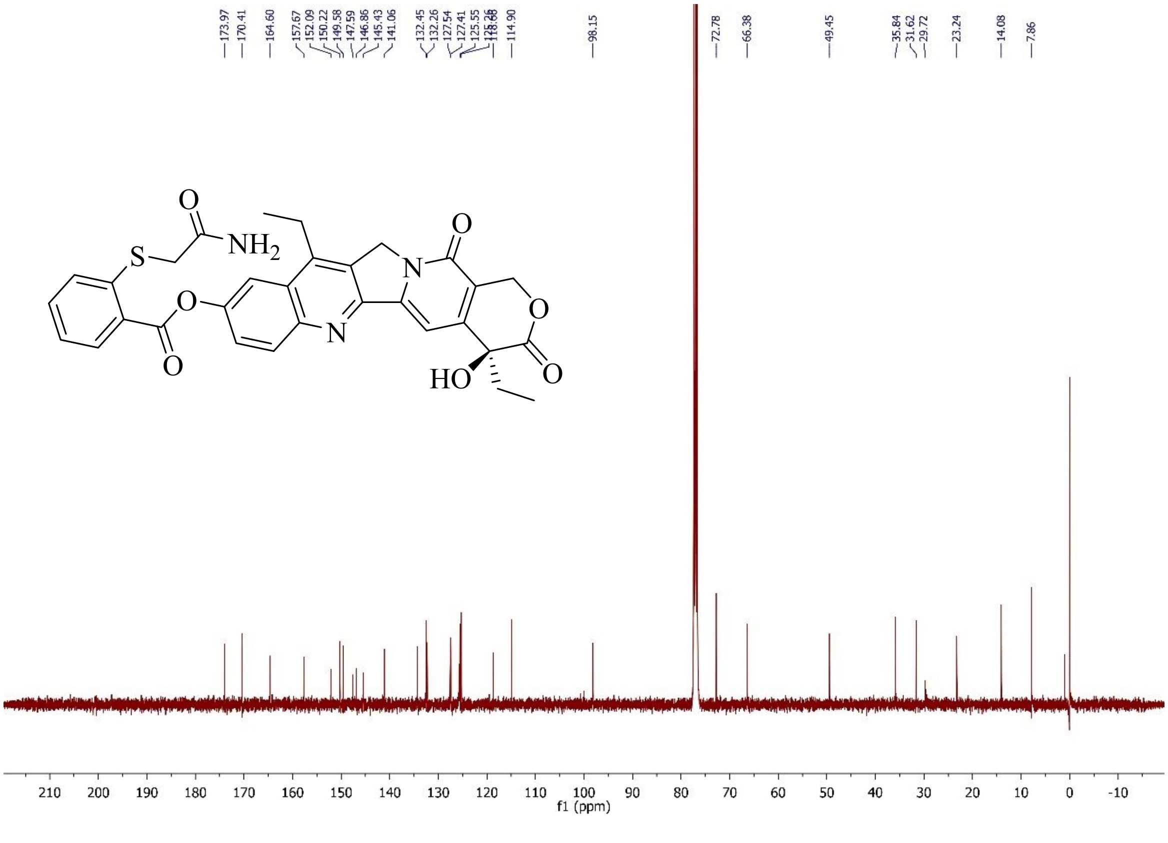

10 MS Spectra

Compound **3a**. (M+H =230.02)

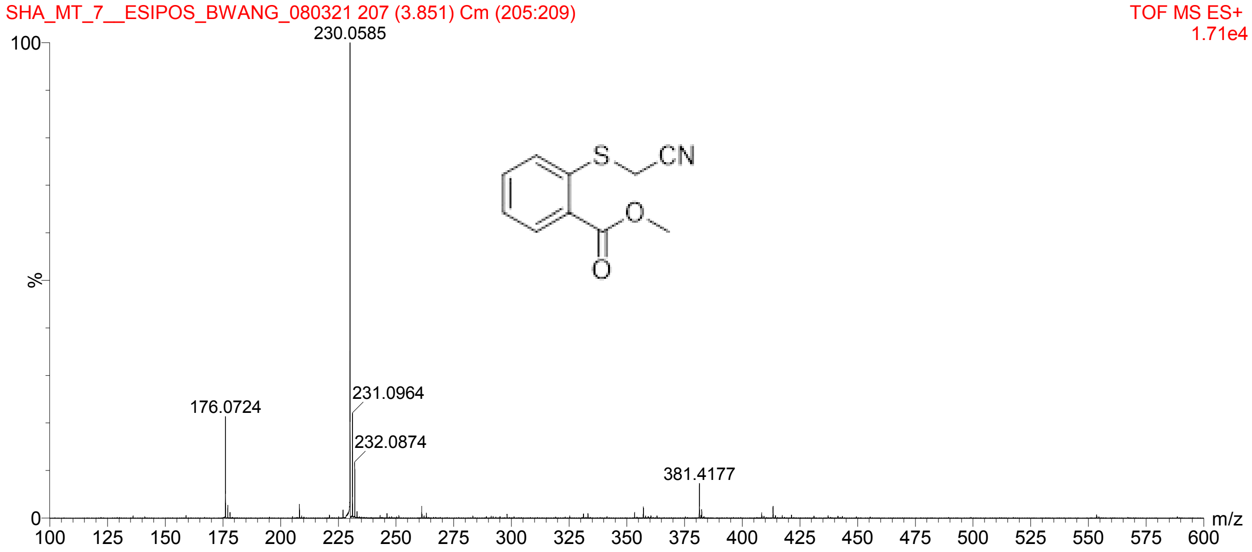

Compound **4a**. (M+H=224.0381)

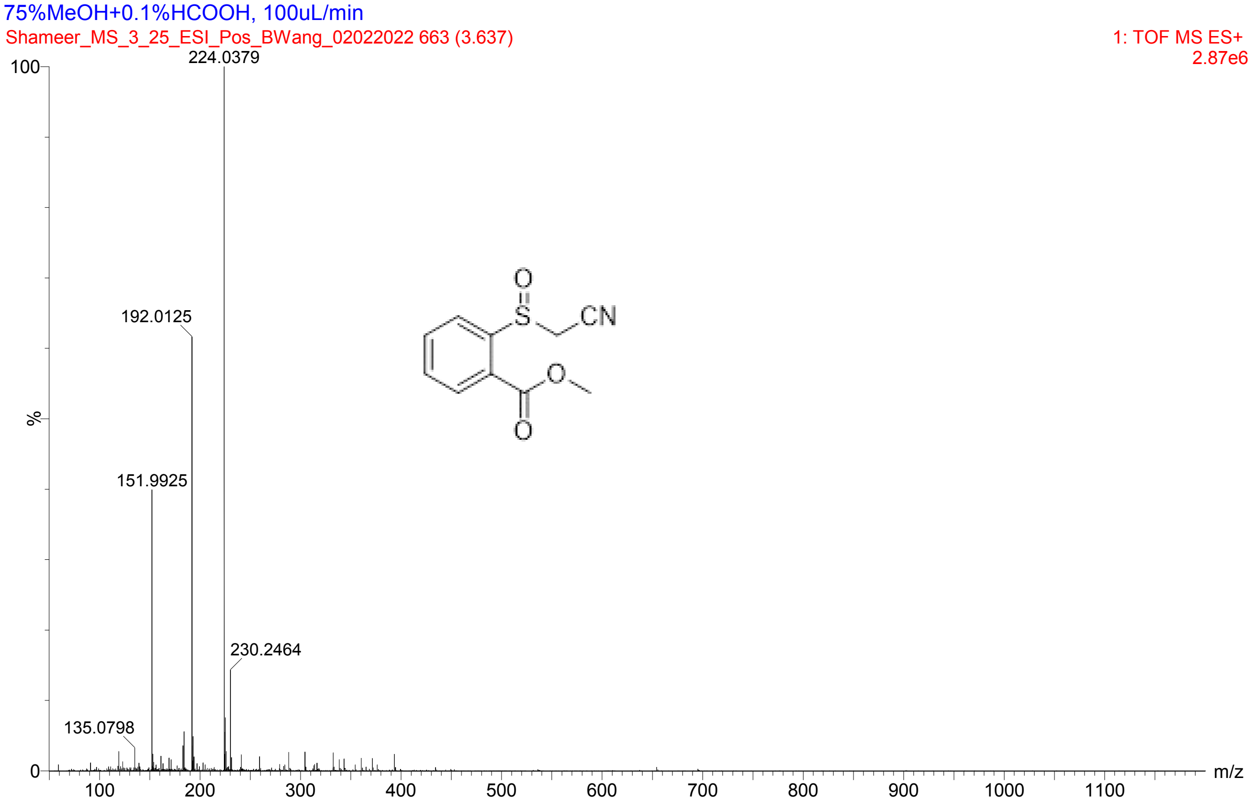

Compund **5a**. (M+H=176.0170)

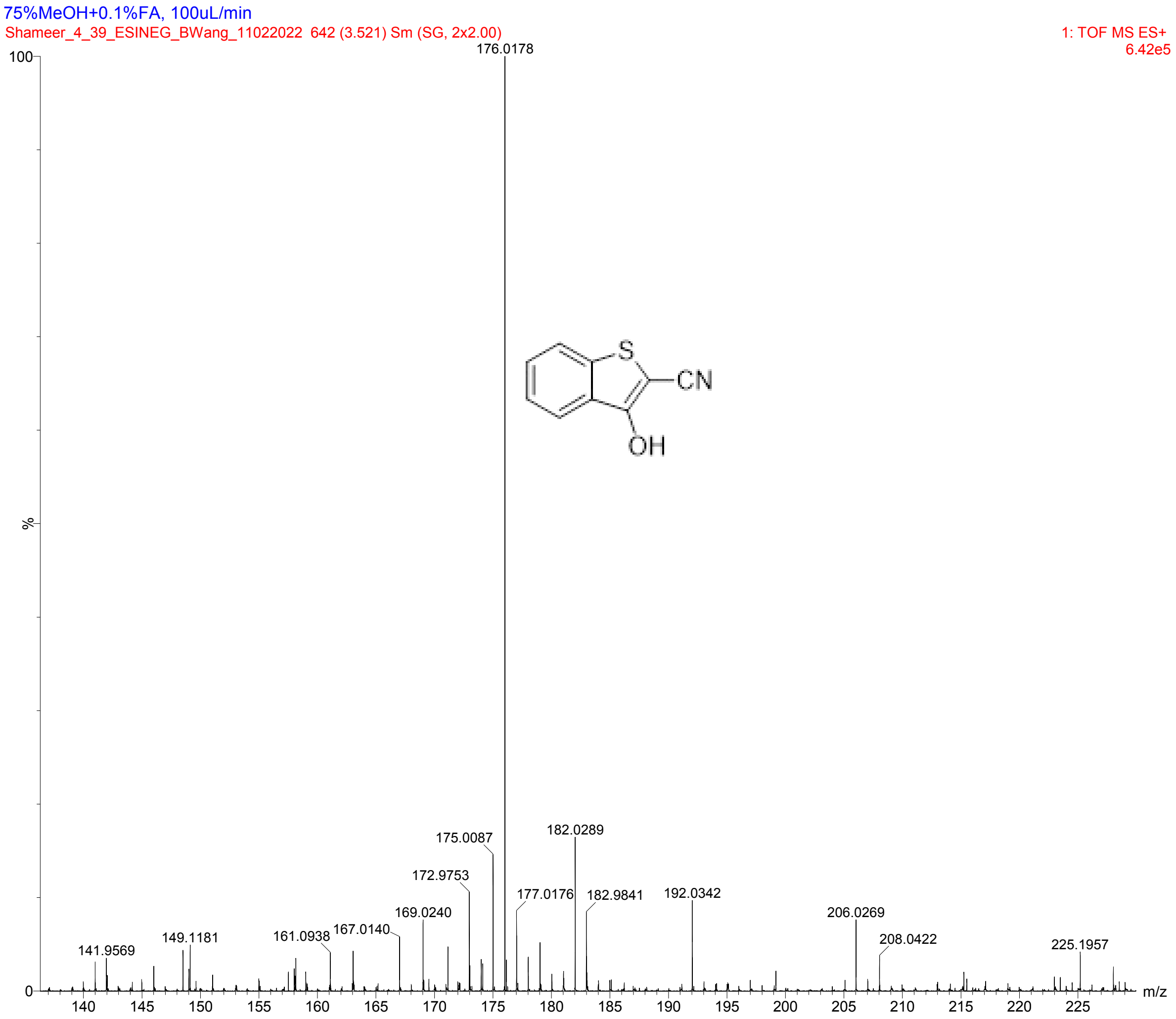

Compound **6a**. (M+H=192.0119)

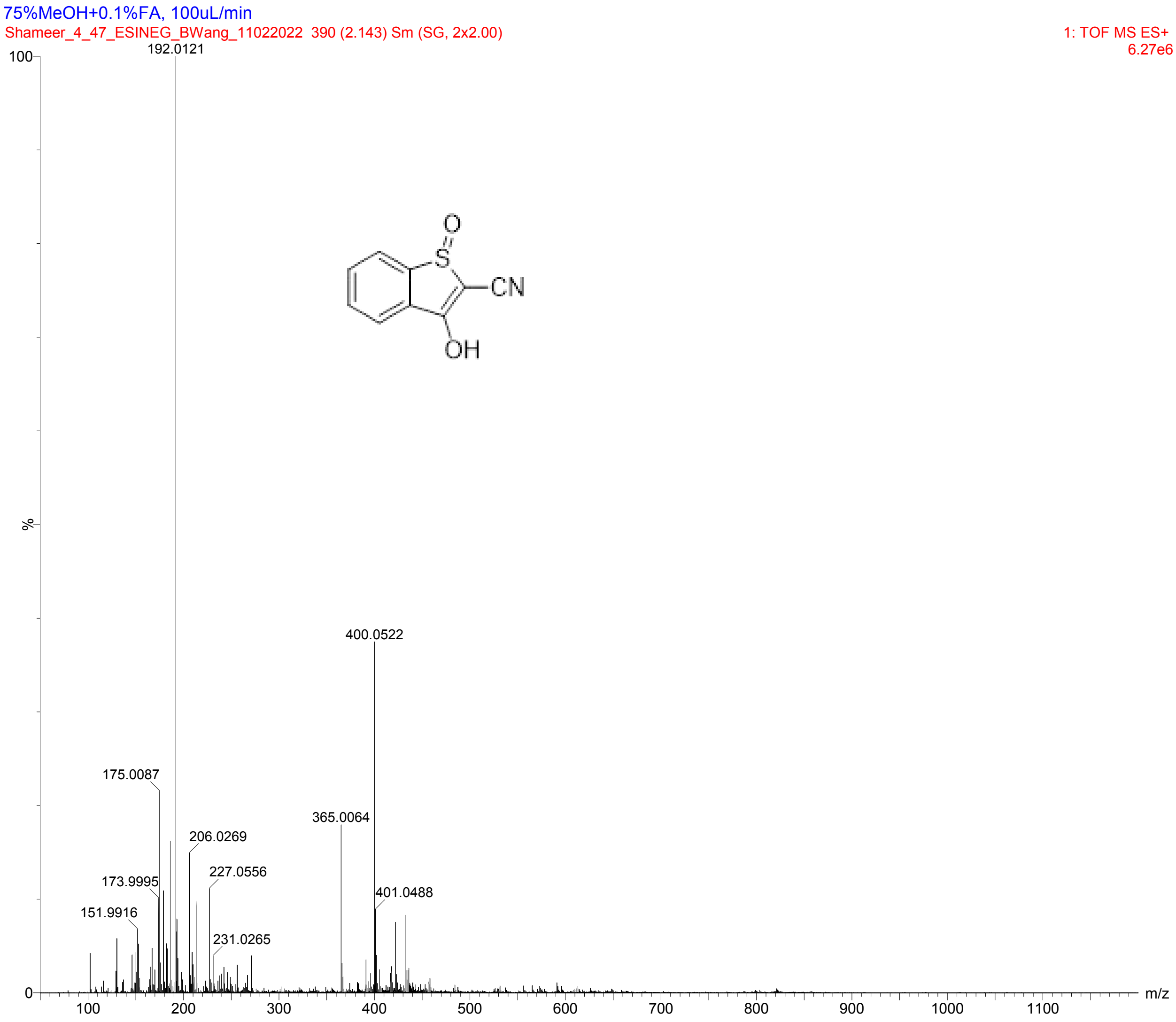

Compound **3b**. (M+H =250.0275)

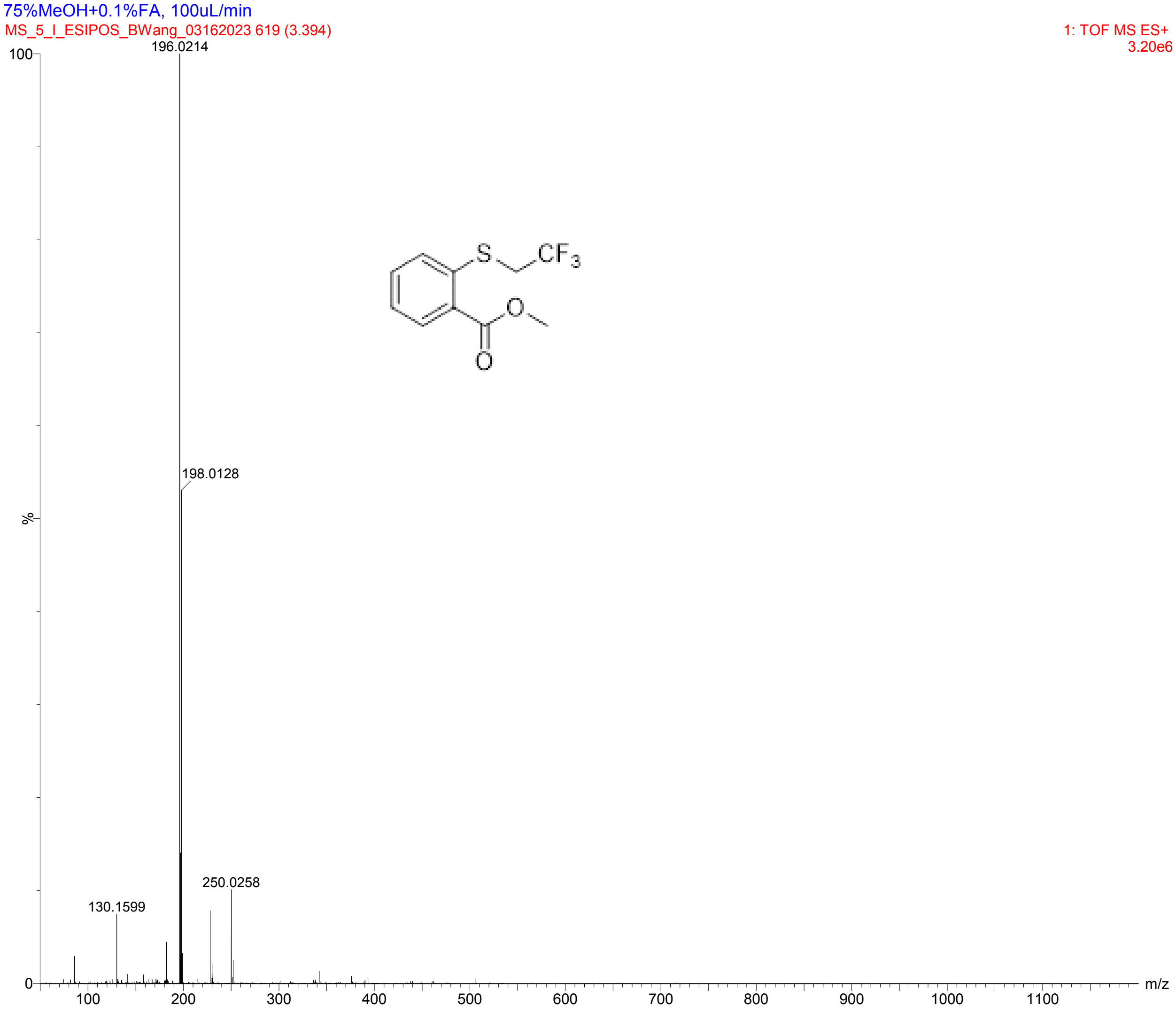

Compound **4b**. (M+H=267.0303)

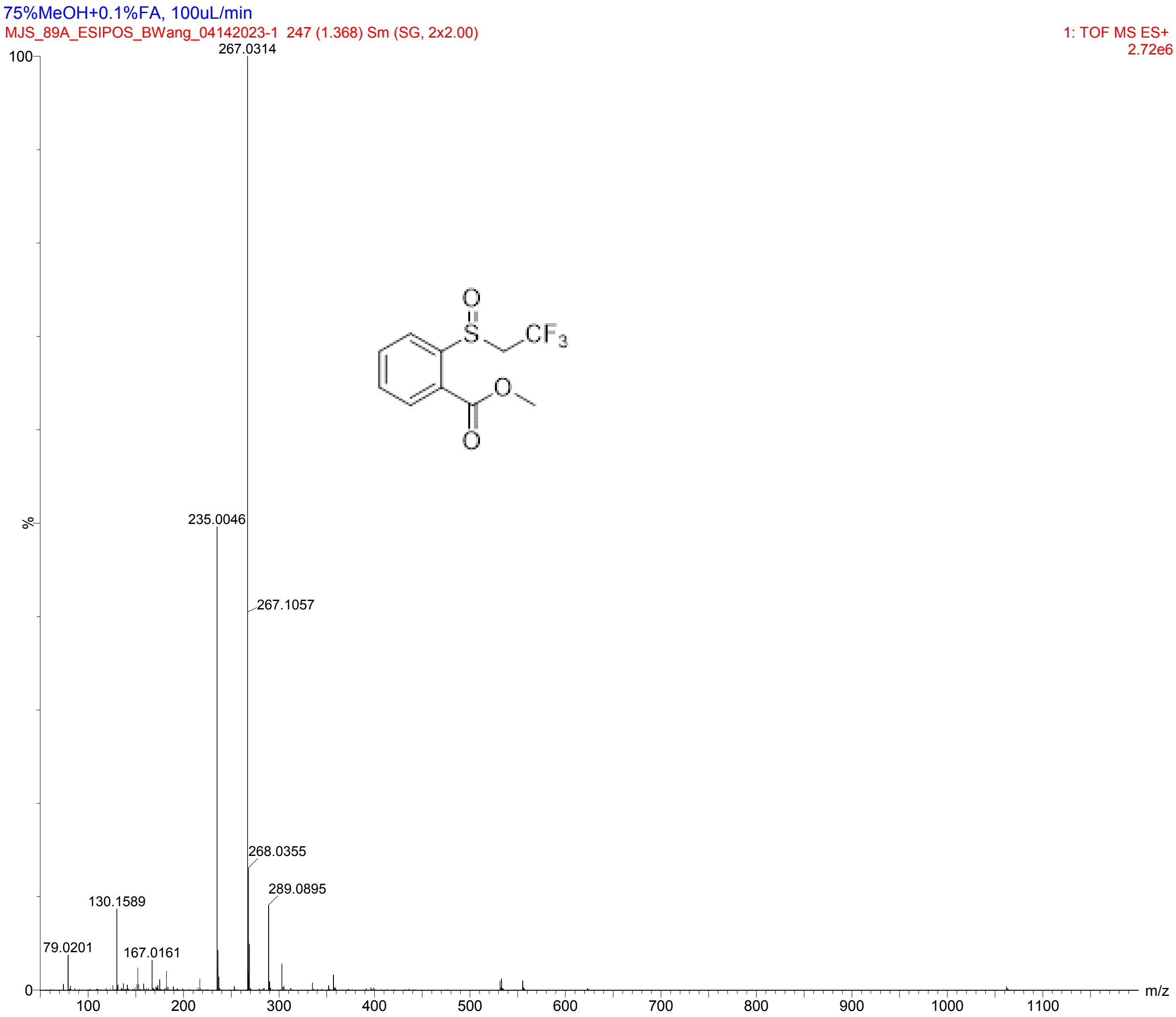

Compound **3c**. (M+H=267.1055)

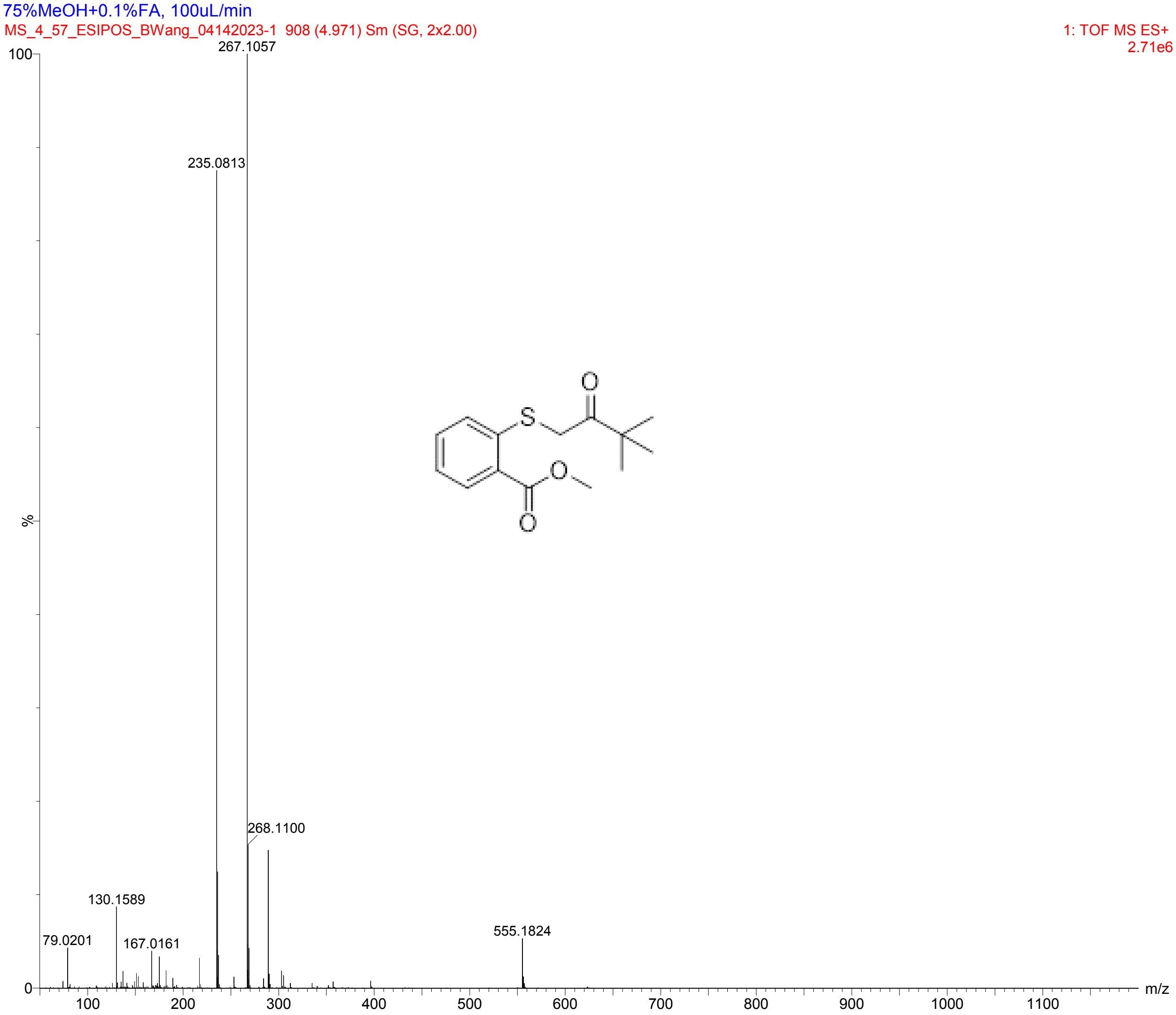

Compound **4c**. (M+H=283.1004)

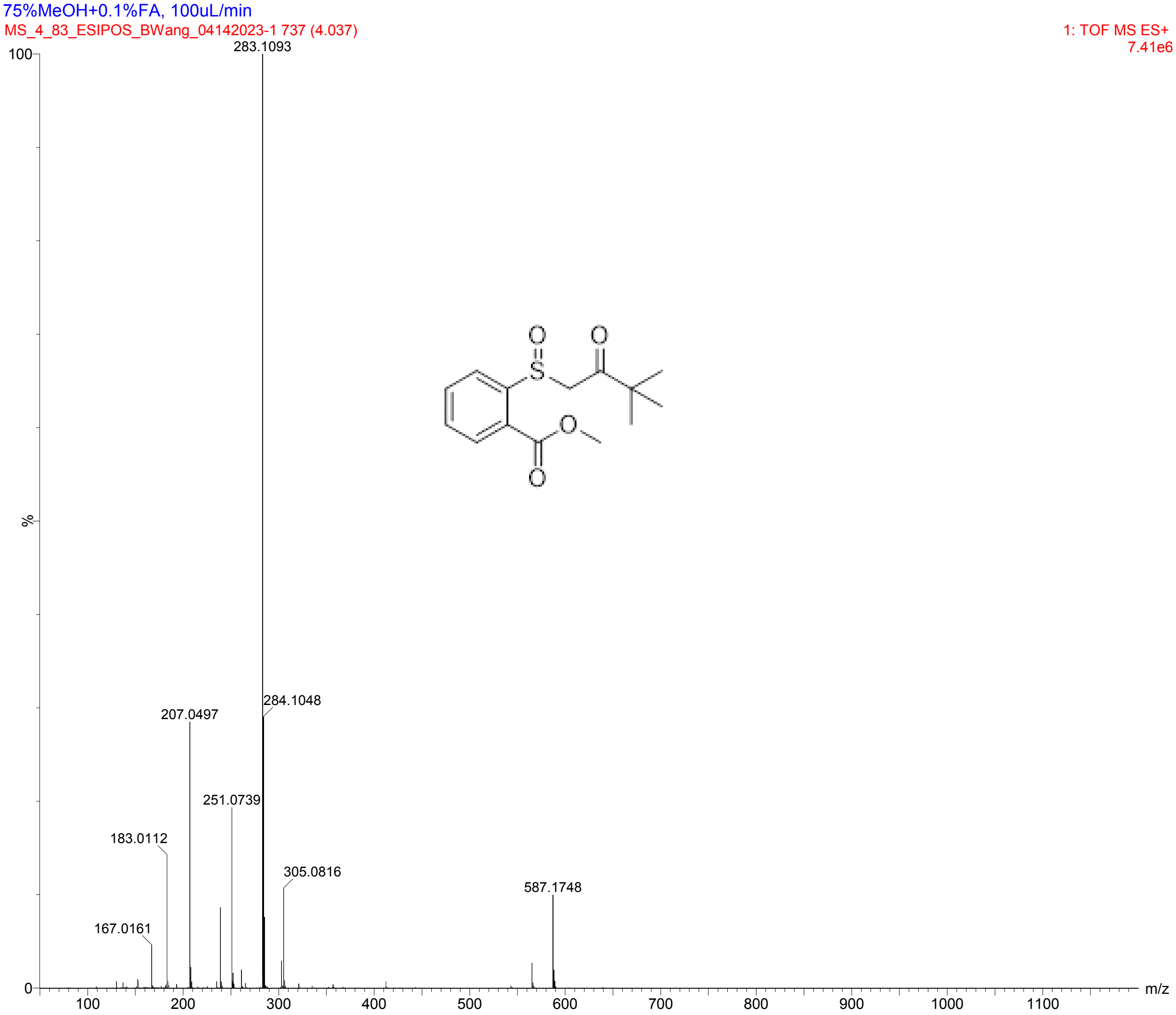

Compound **5c**. (M+H=235.0748)

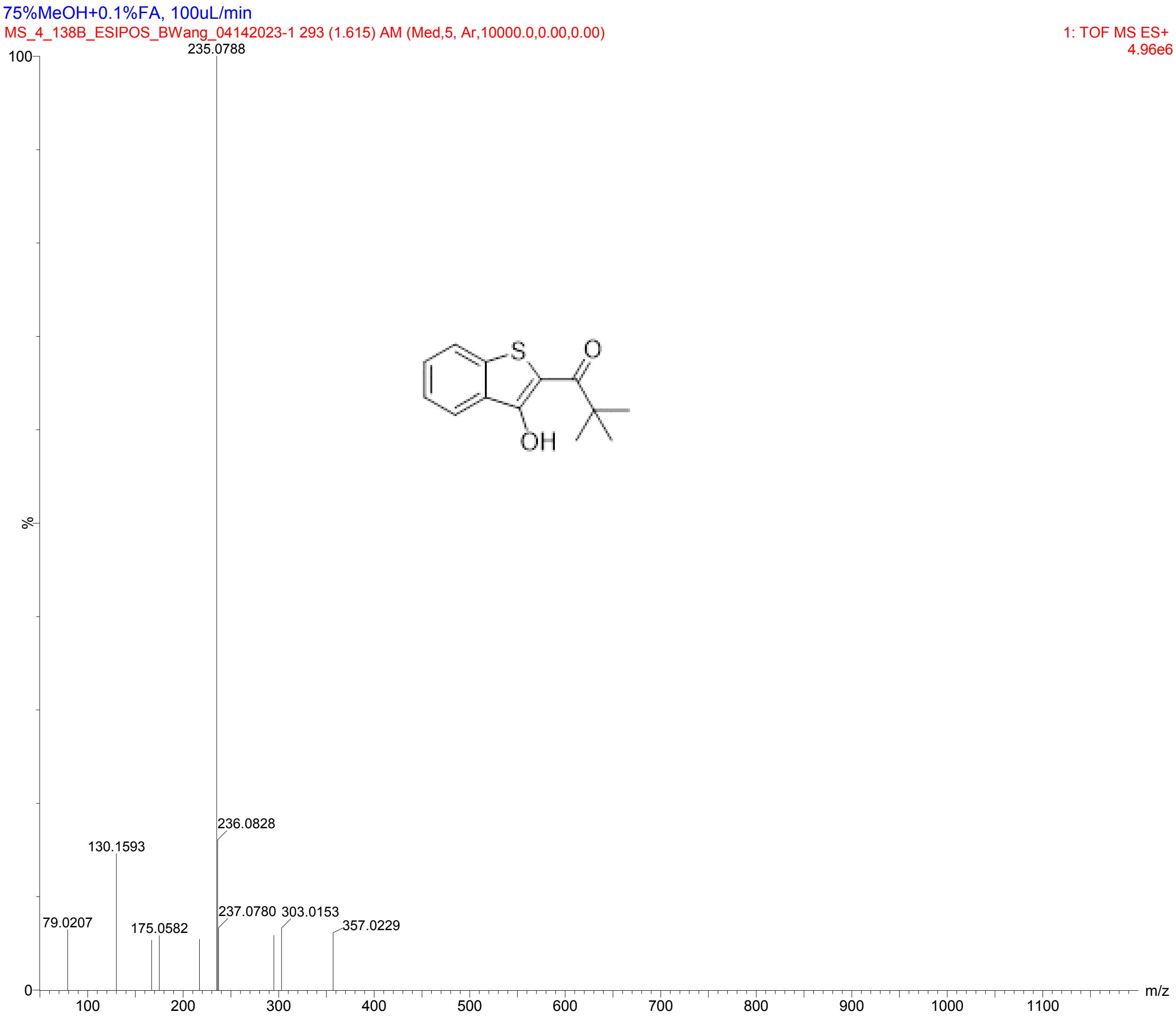

Compound **6c**. (M+H=251.0742)

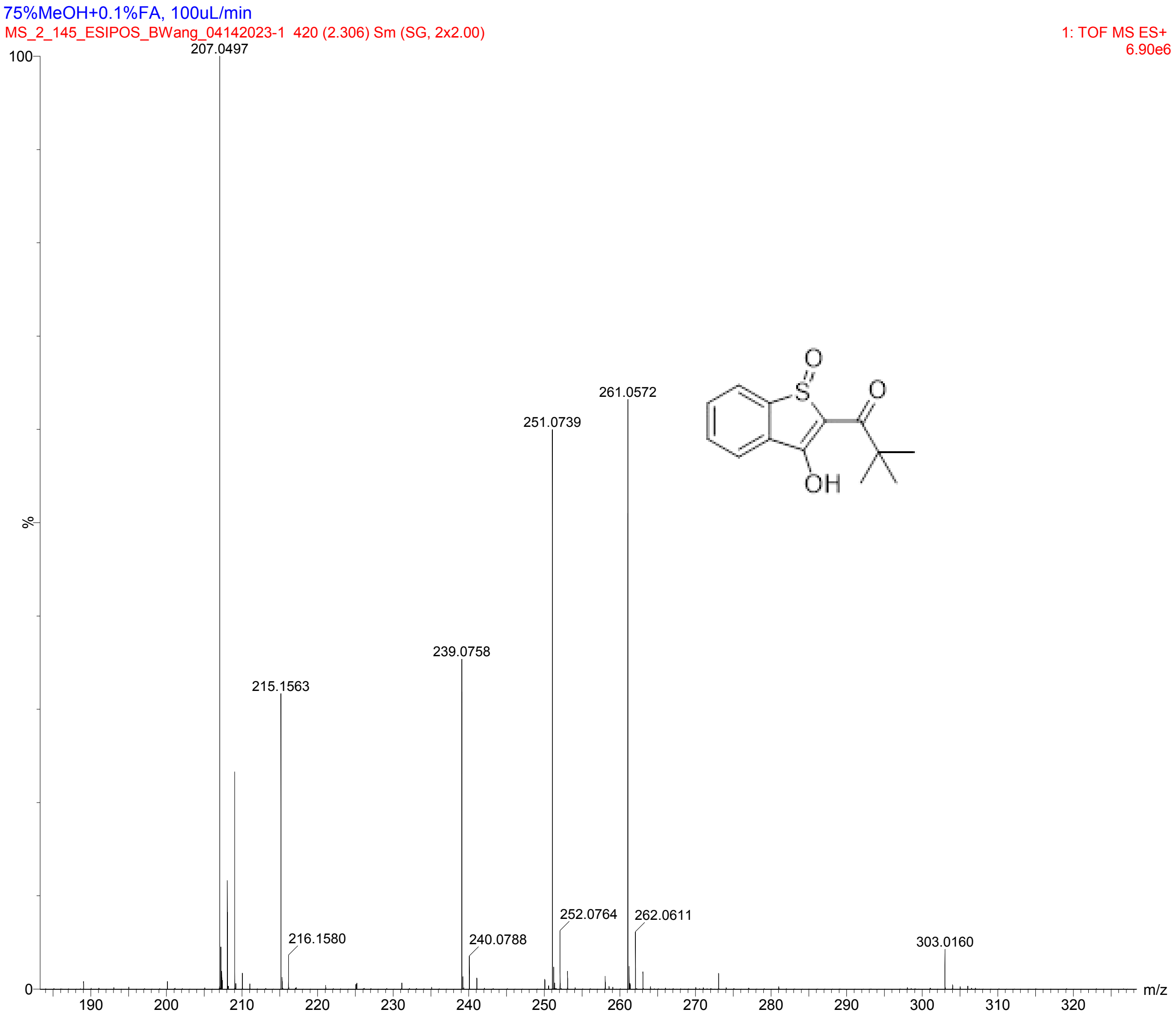

Compund **3d** (M+H= 241.0535)

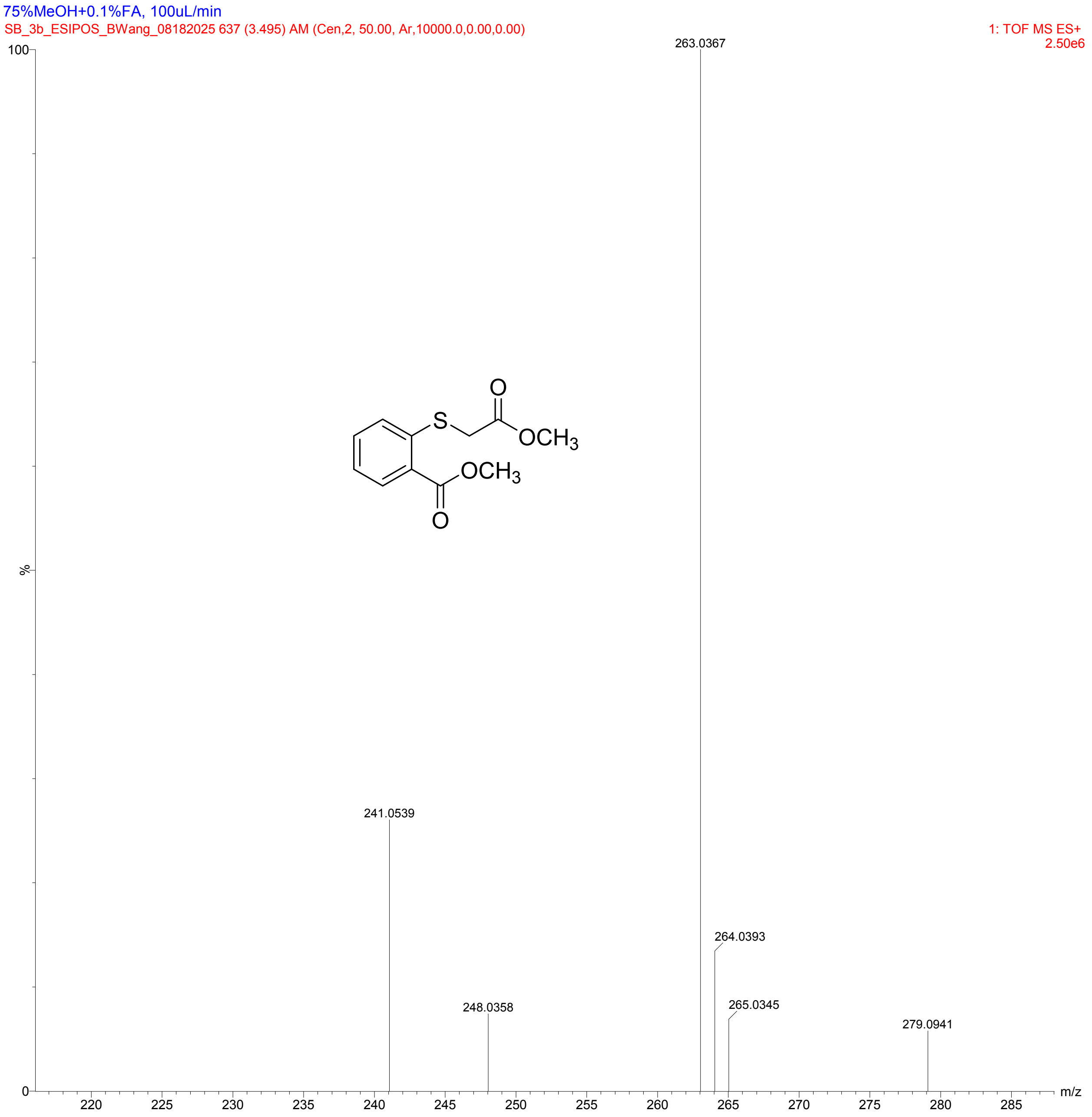

Compound **4d** (M+H=257.0484)

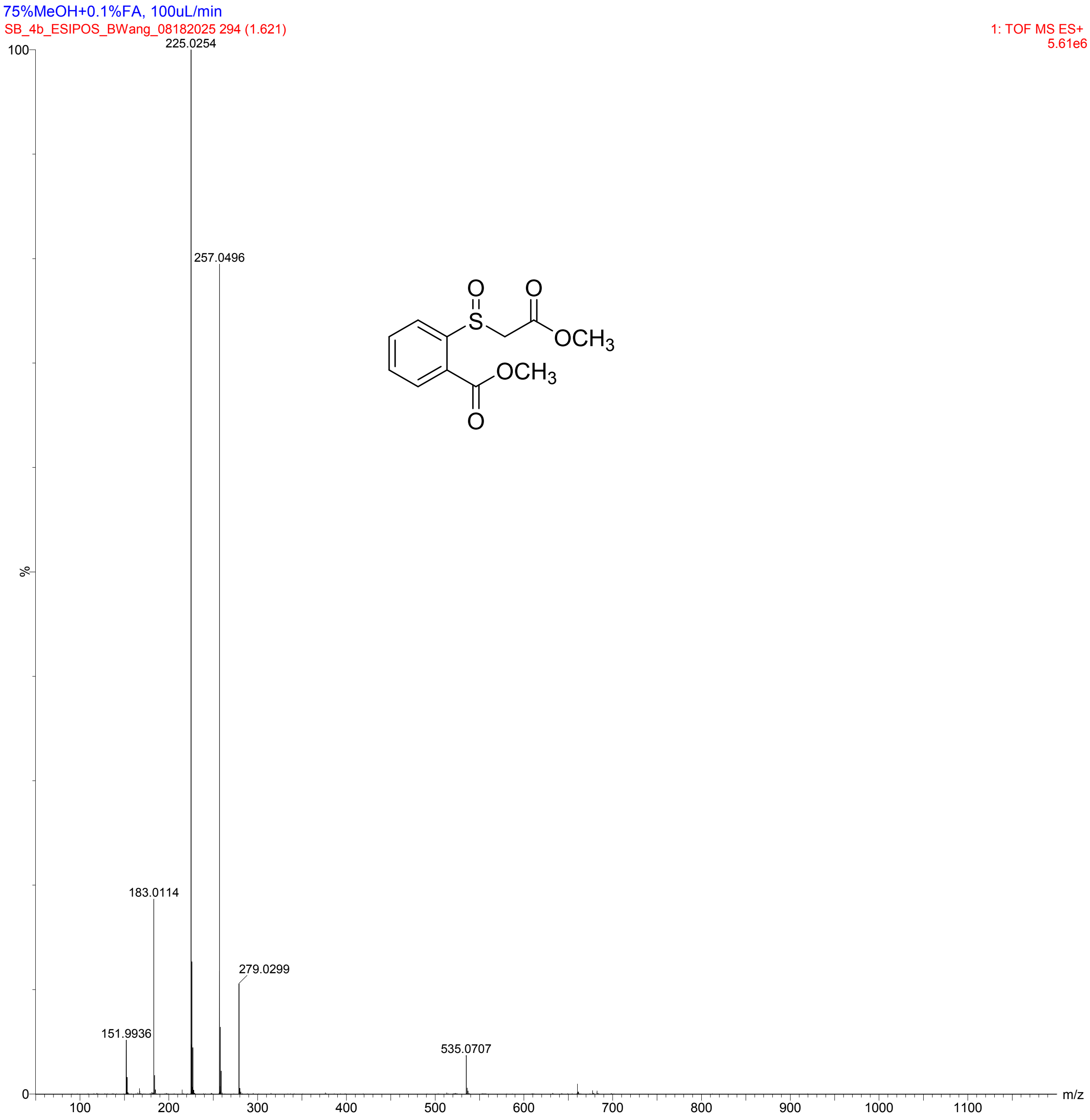

Compound **5d** (M+H= 207.0116)

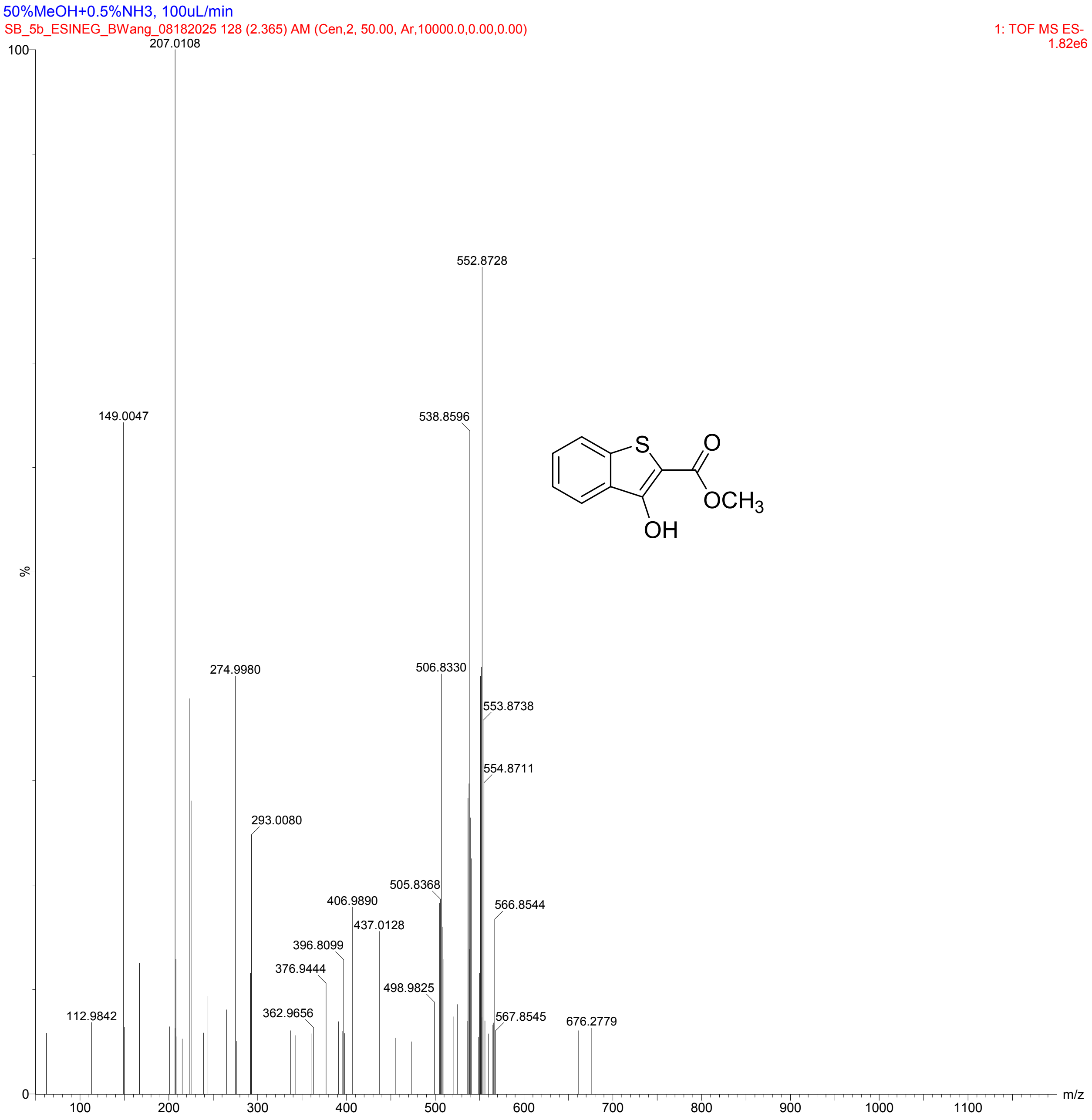

Compound **6d** (M+H= 225.0222)

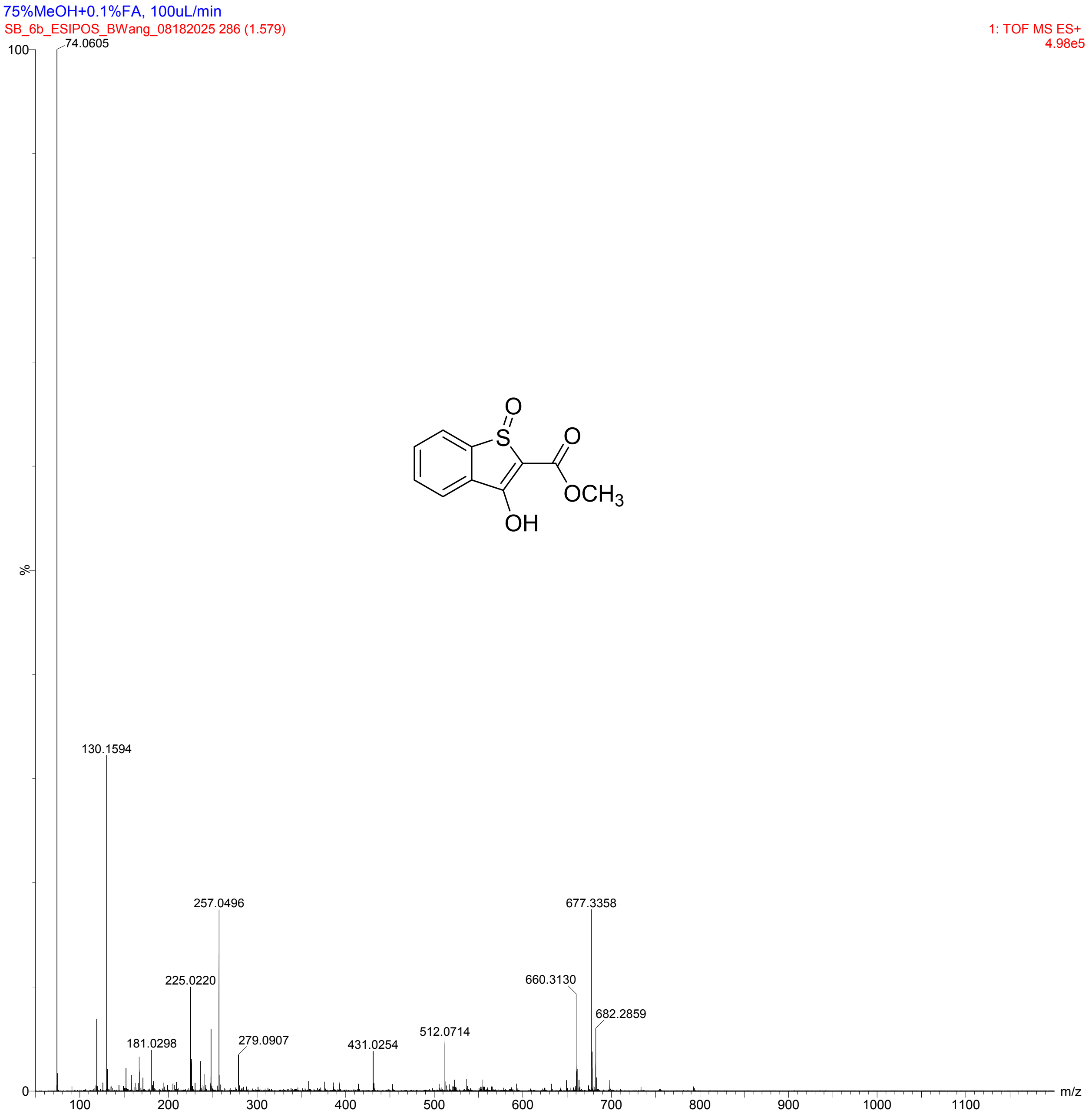

Compound **3e** (M+H = 226.0538)

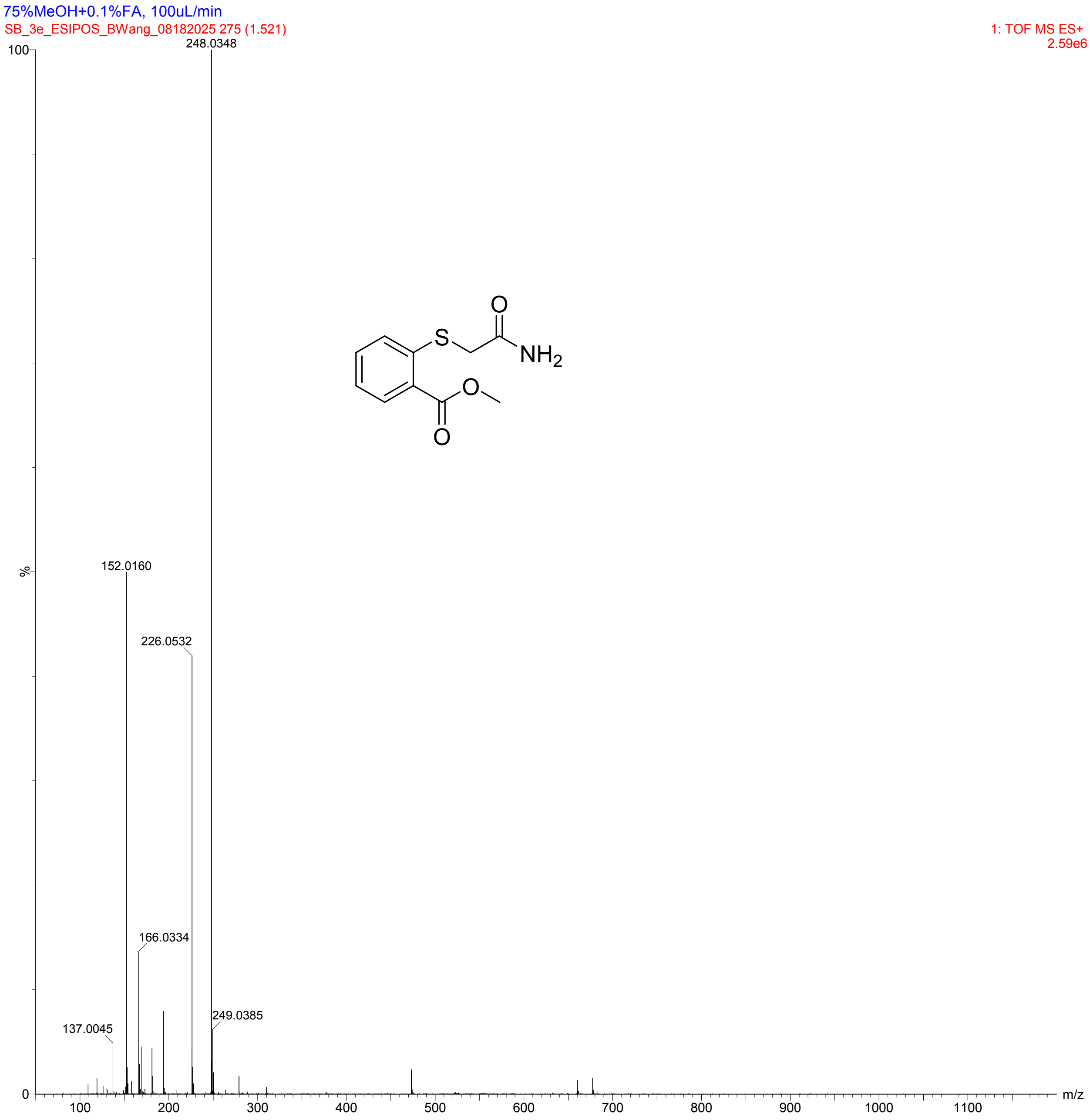

Compound **4e**. (M+H=242.0487)

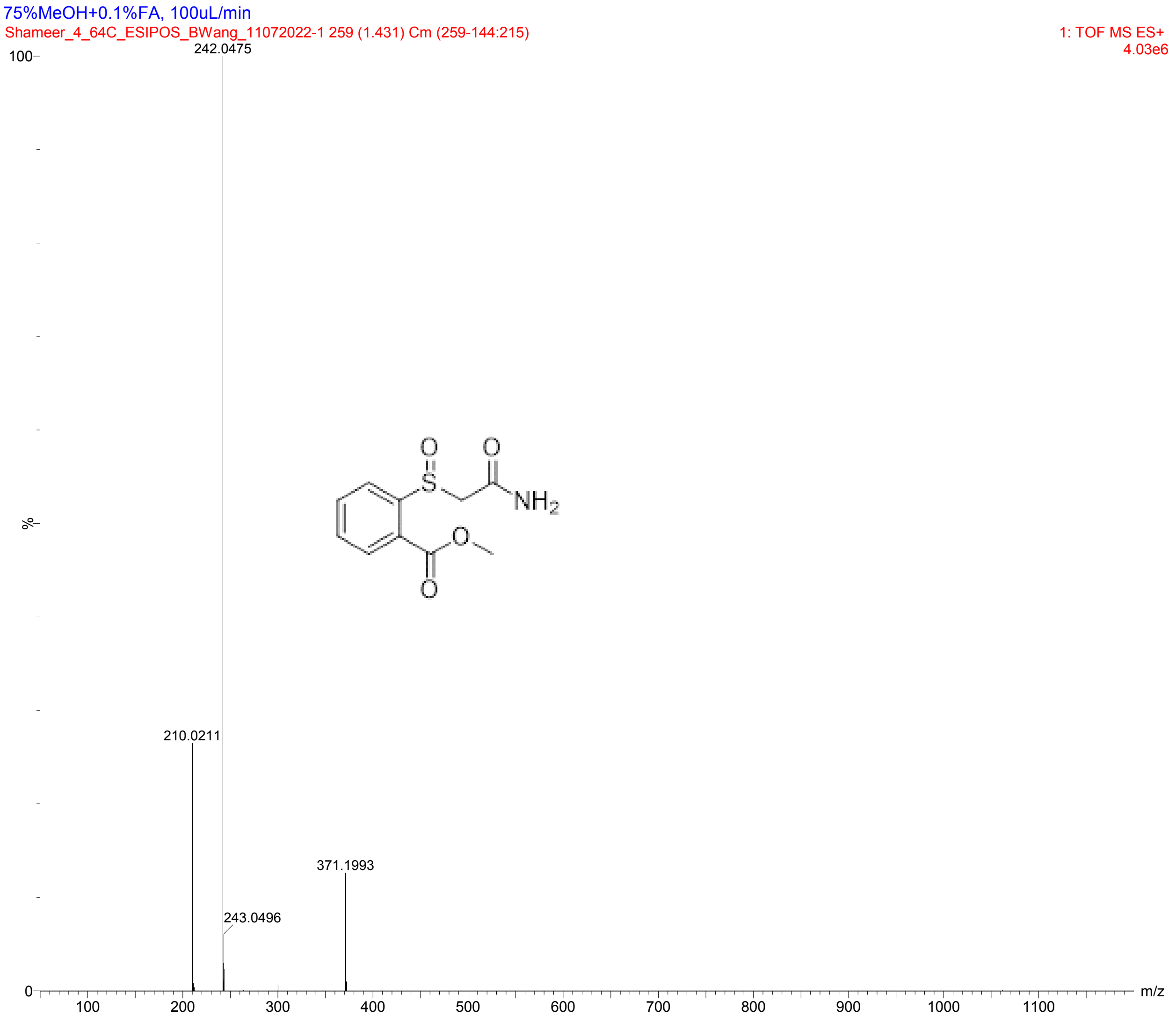

Compound **5e**. (M+H = 194.0276[])

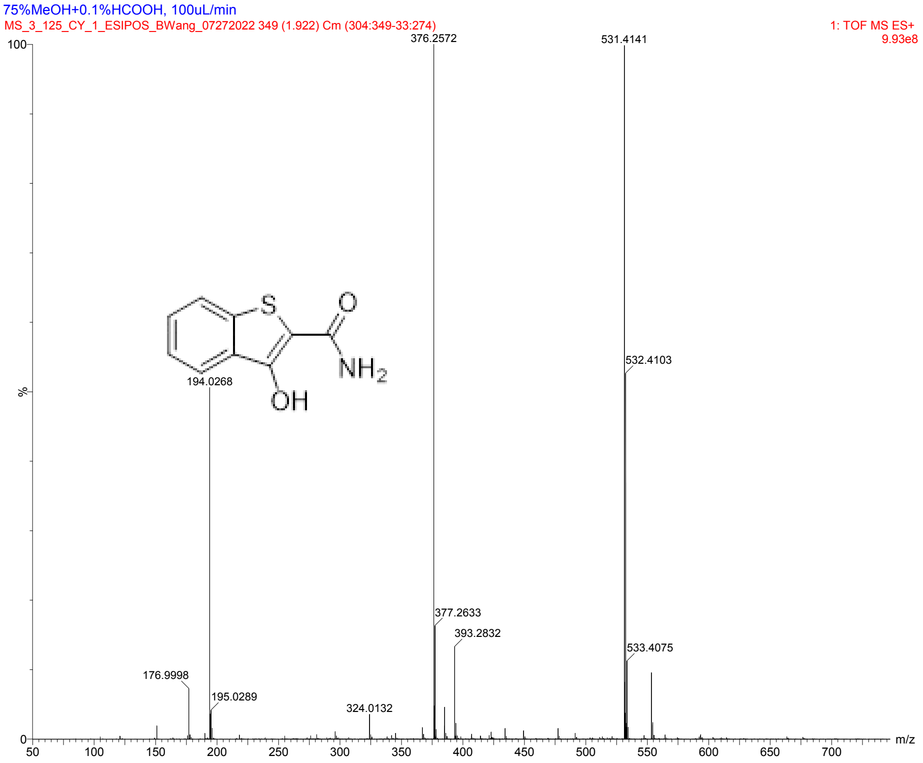

Compound **6e**. (M+H = 209.0147)

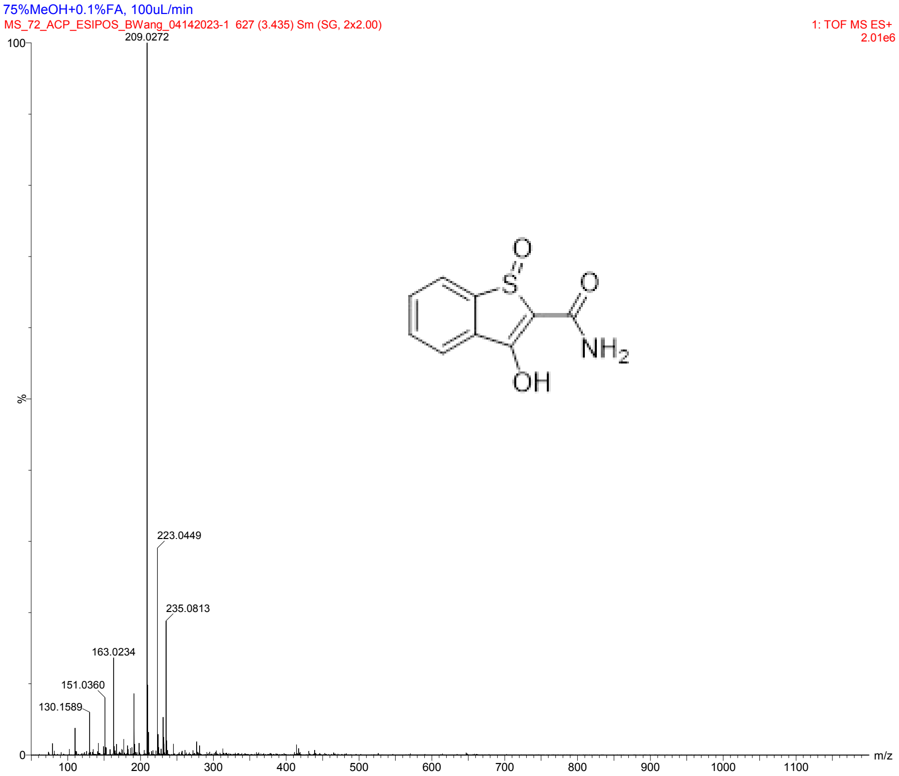

Compound **3f**. (M+H=268.1007)

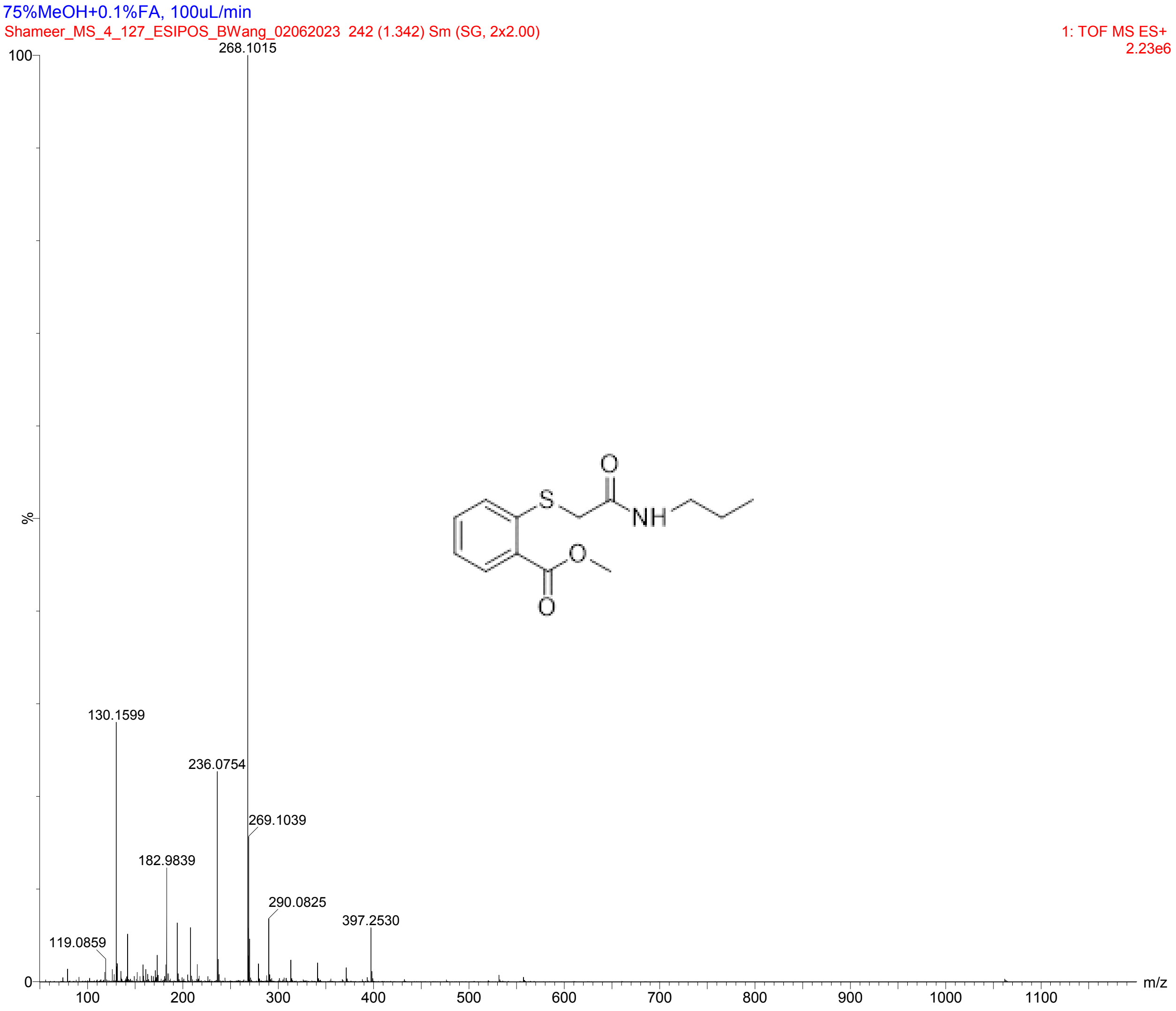

Compound 4f. (M+H=283.0878)

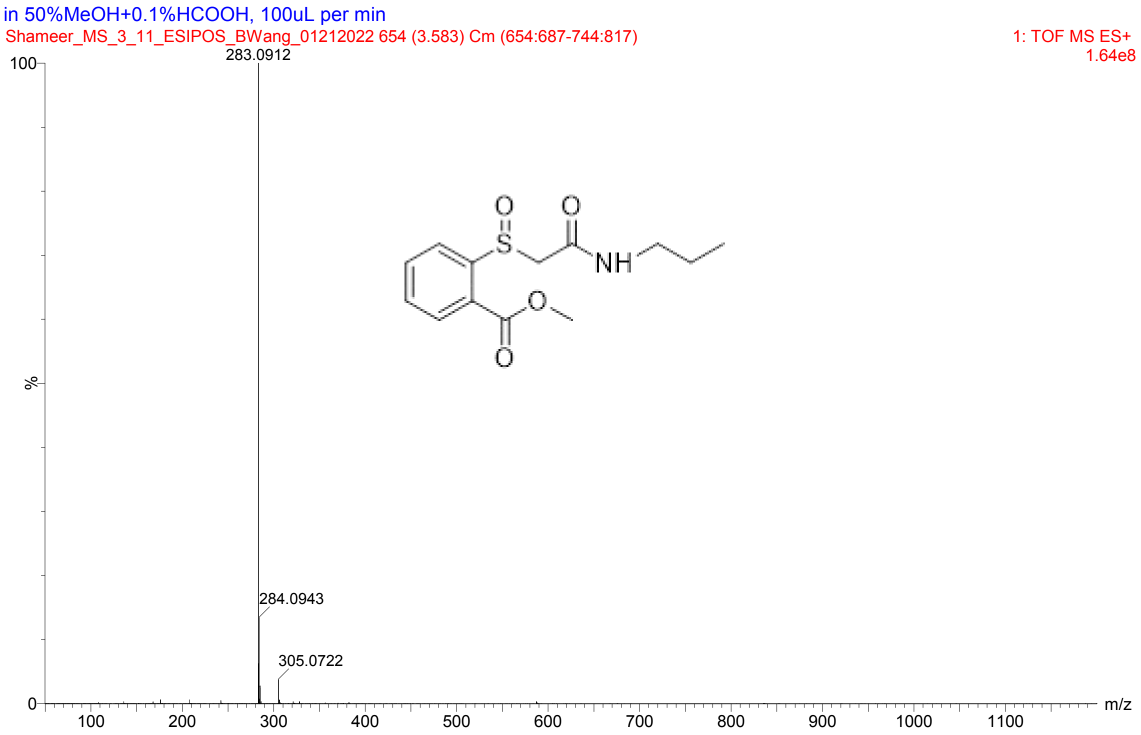

Compound **5f**. (M+H=236.0745)

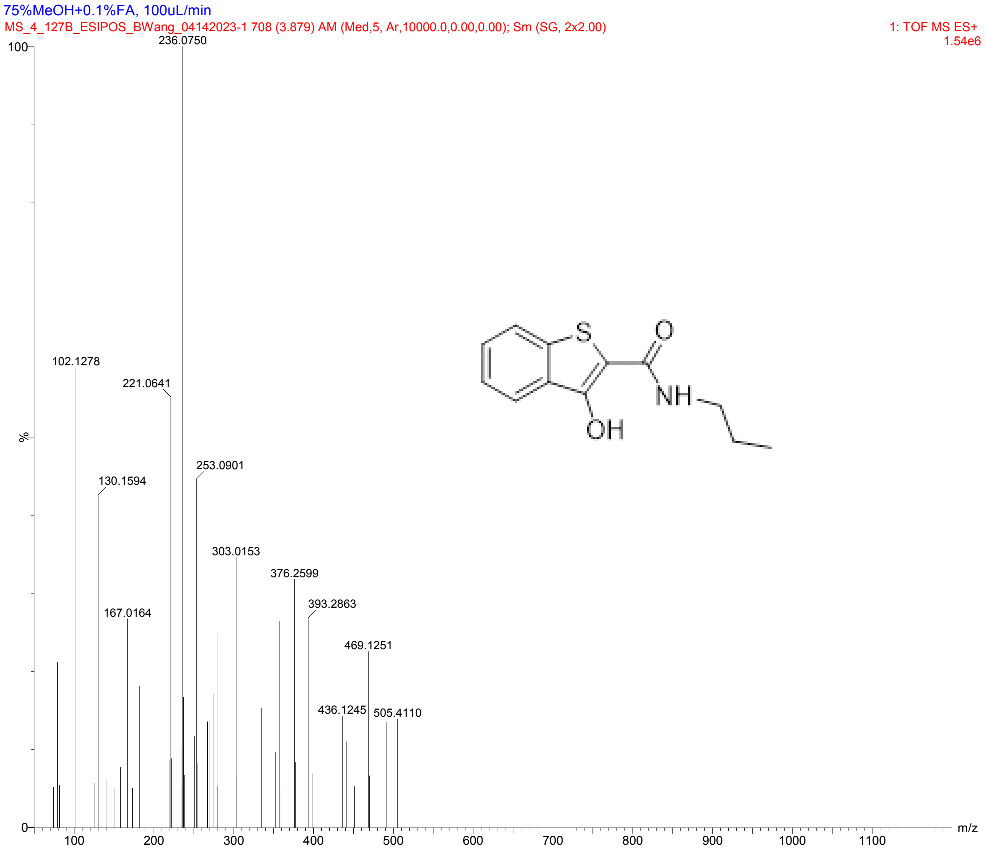

Compound **3g**. (M+H=227.0378)

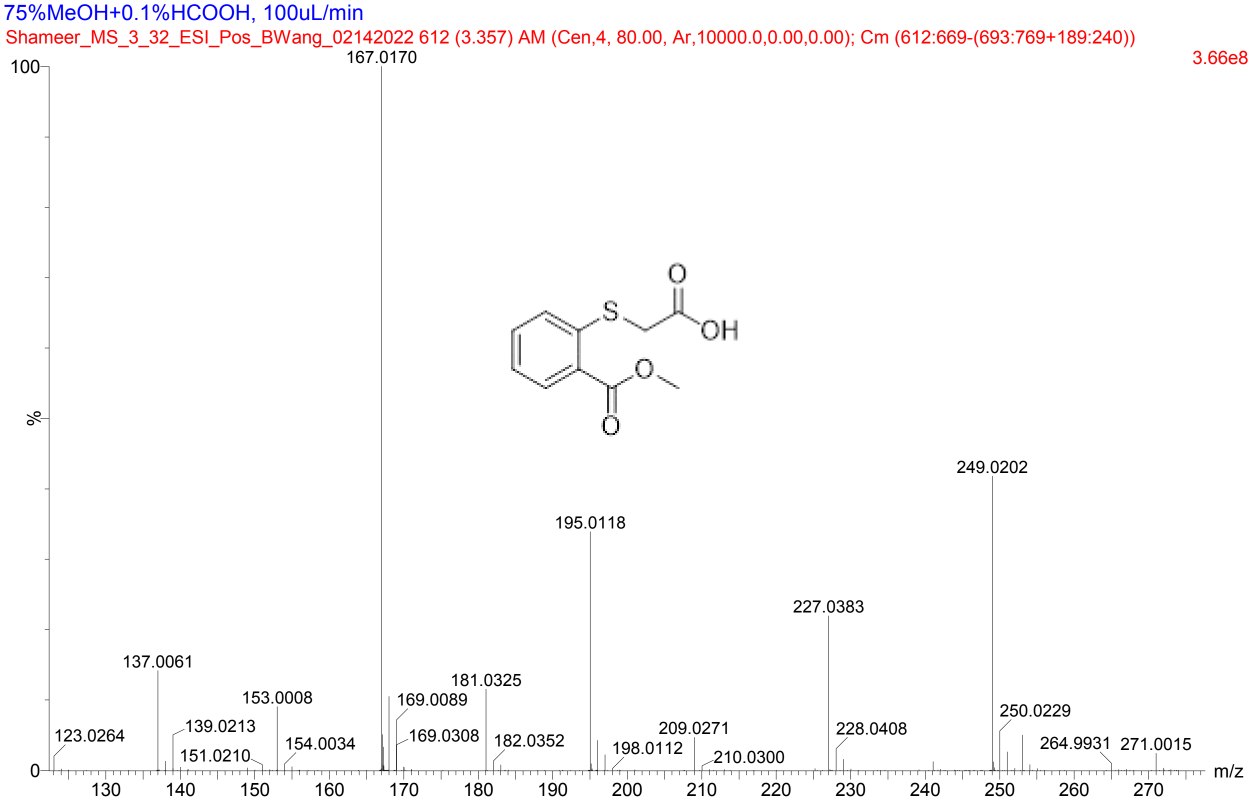

Compound **4g**. (M+H =243.0327)

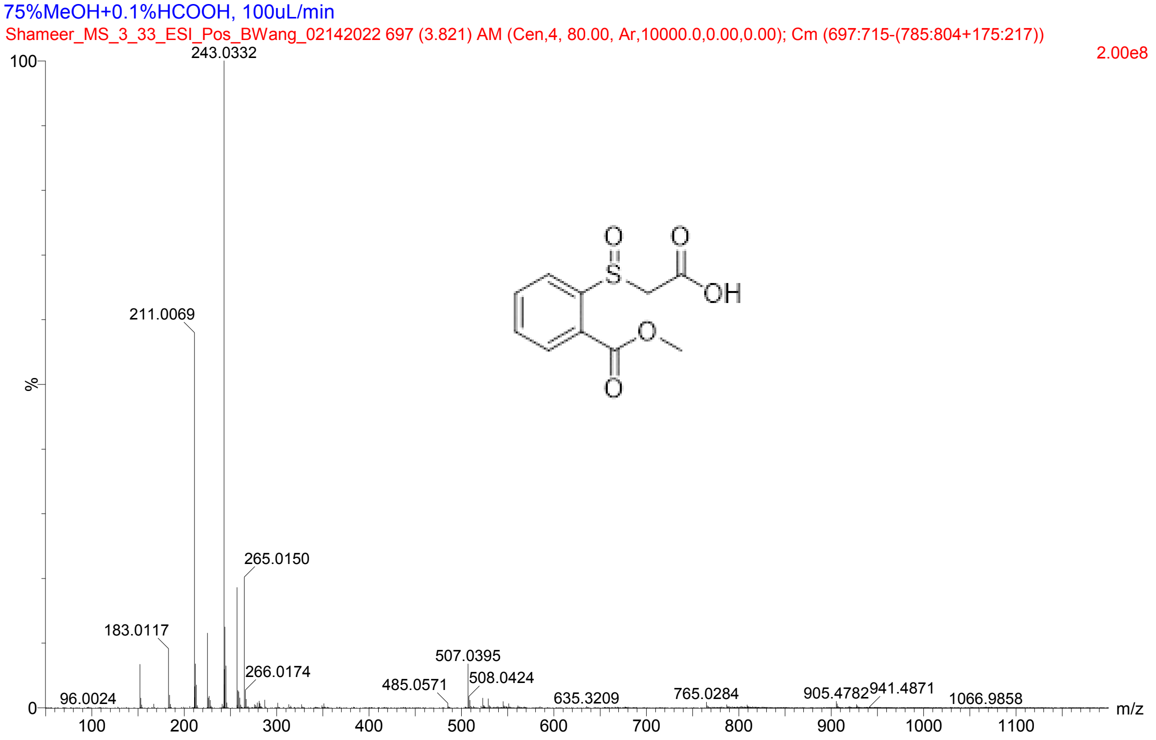

Compound **11a**. (M+H=537.1720)

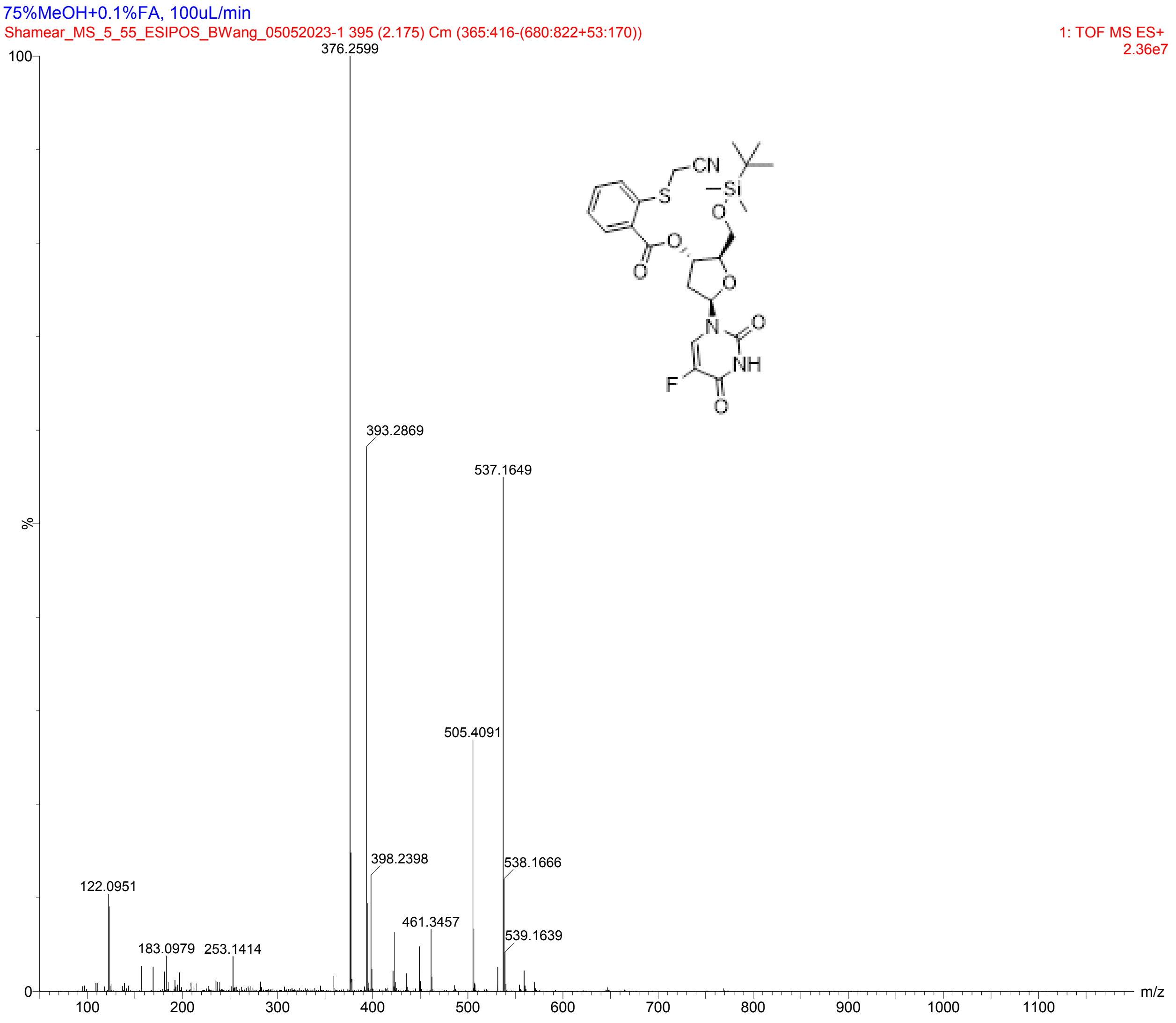

Compound **11d** (M+H= 569.1789)

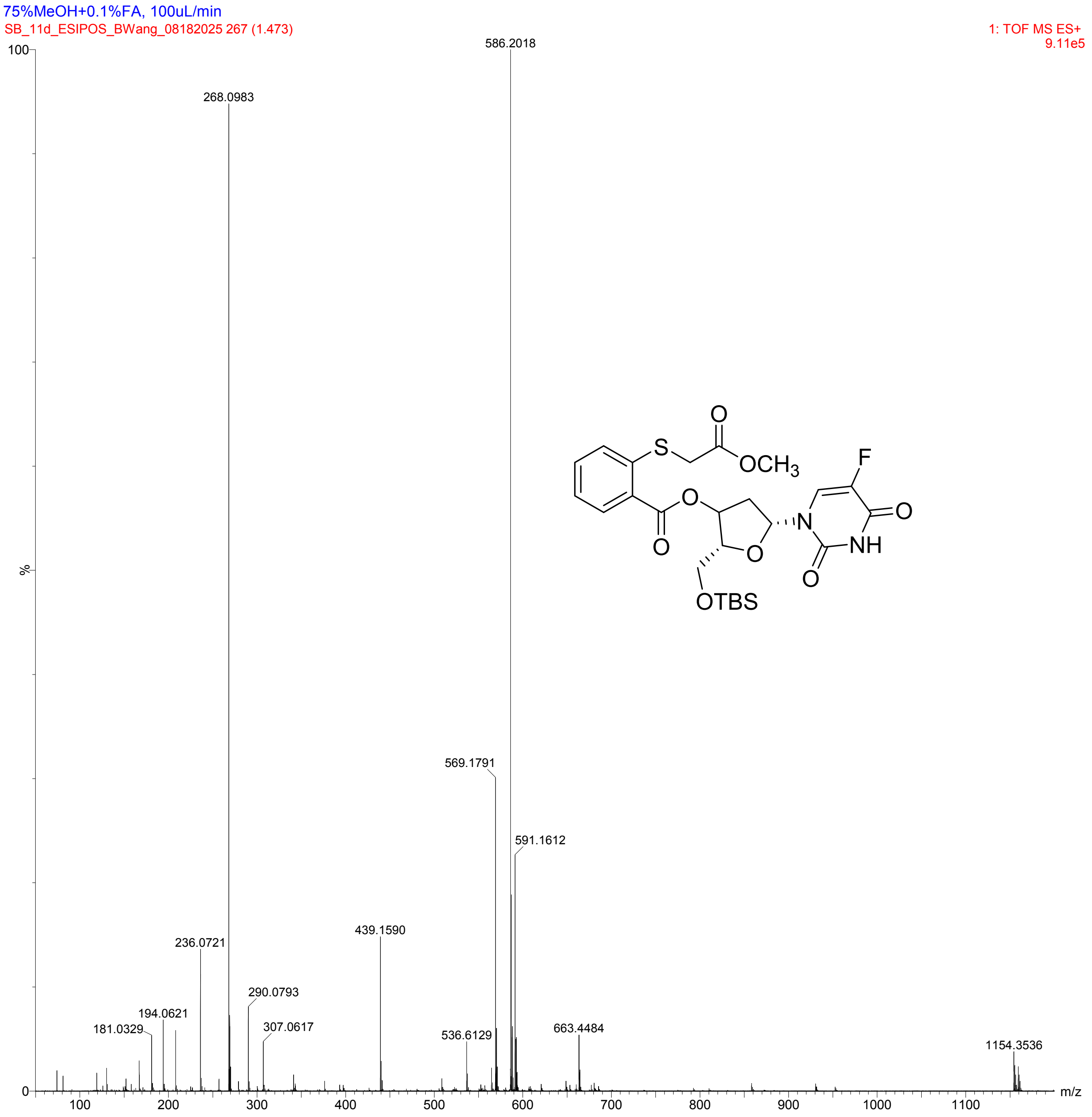

Compound **11e** (M+H = 554.1793)

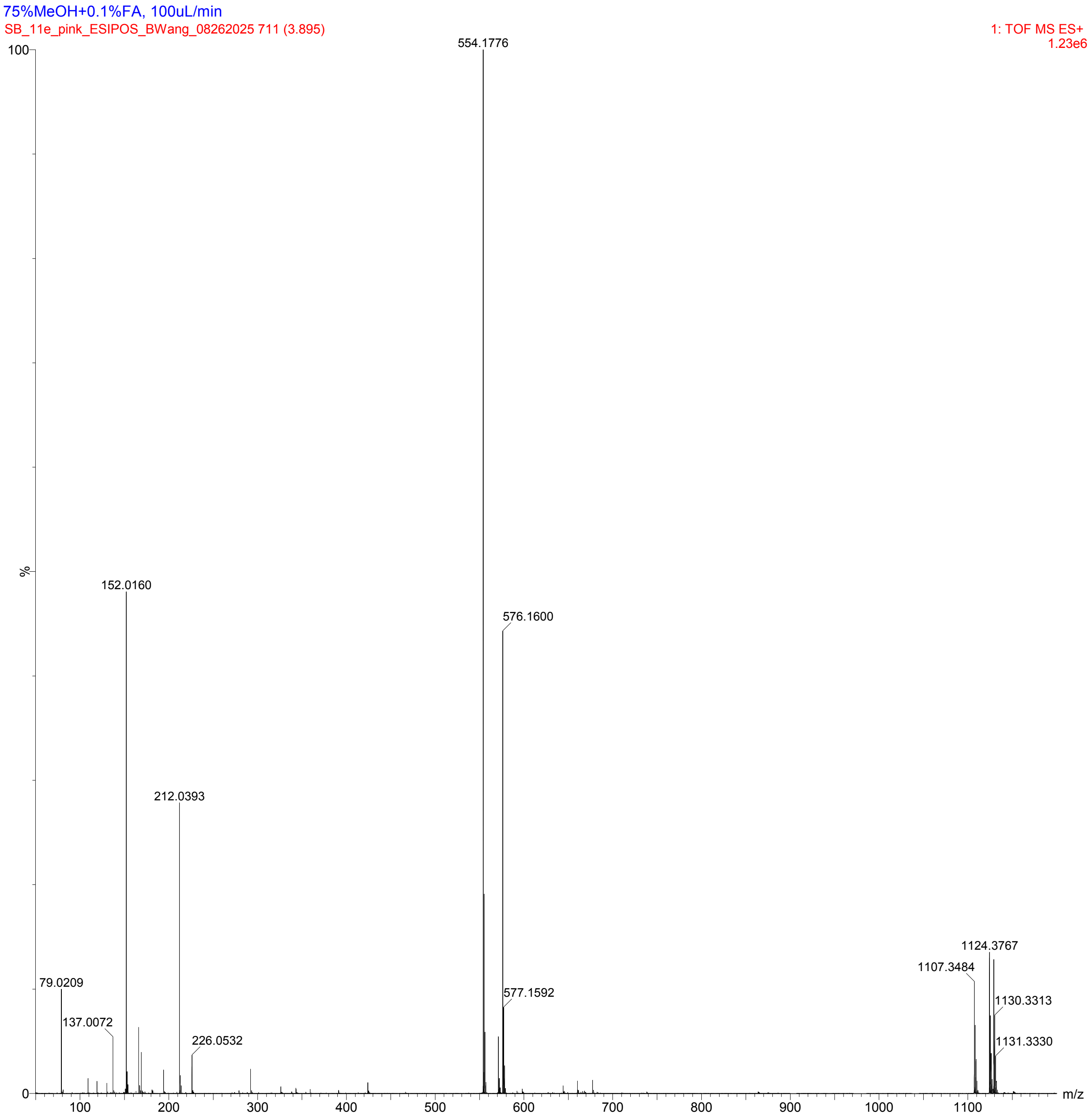

Compound **11f** (M+H= 589.2262)

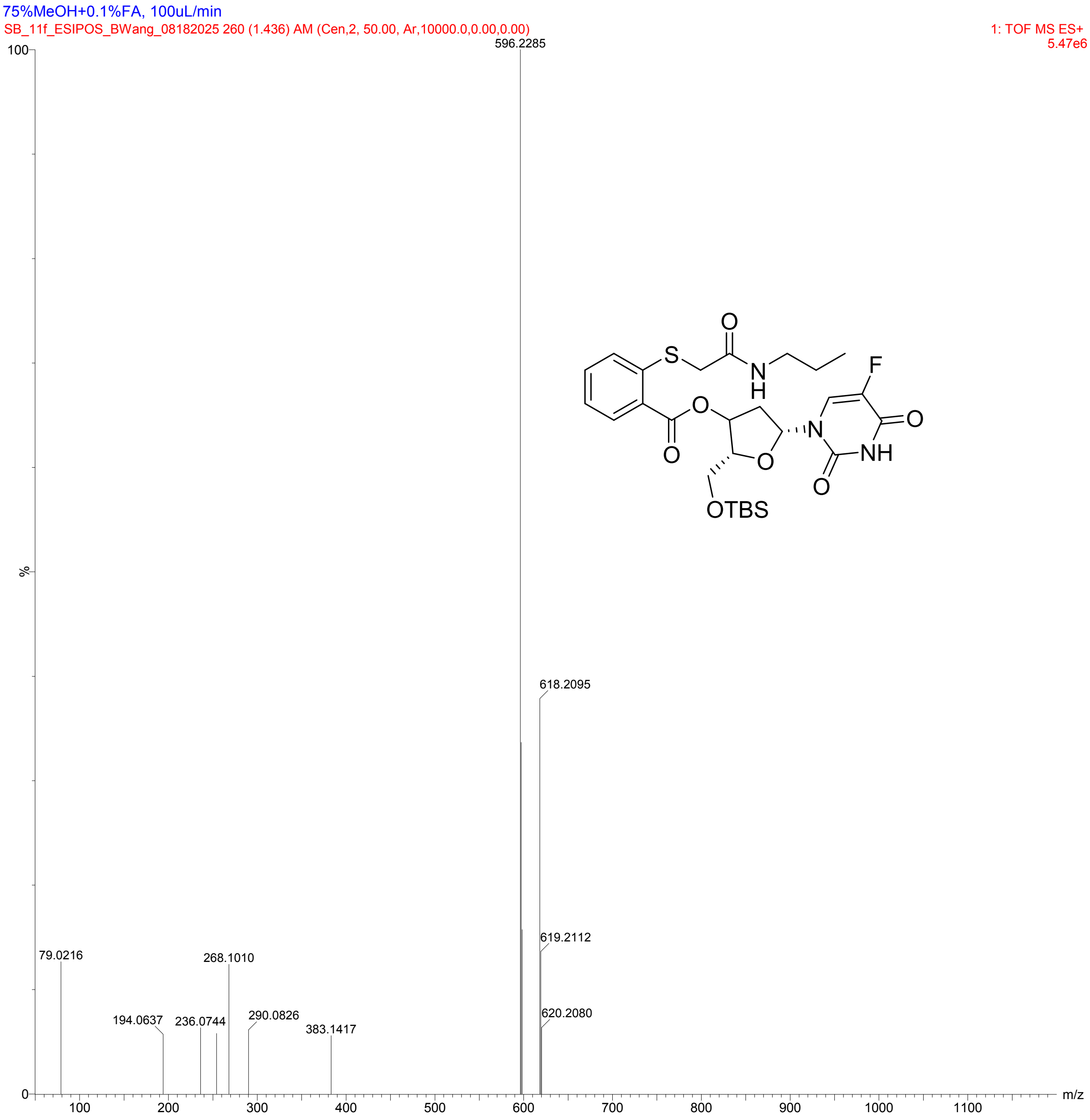

Compound **12a**.(M+H = 420.0683)

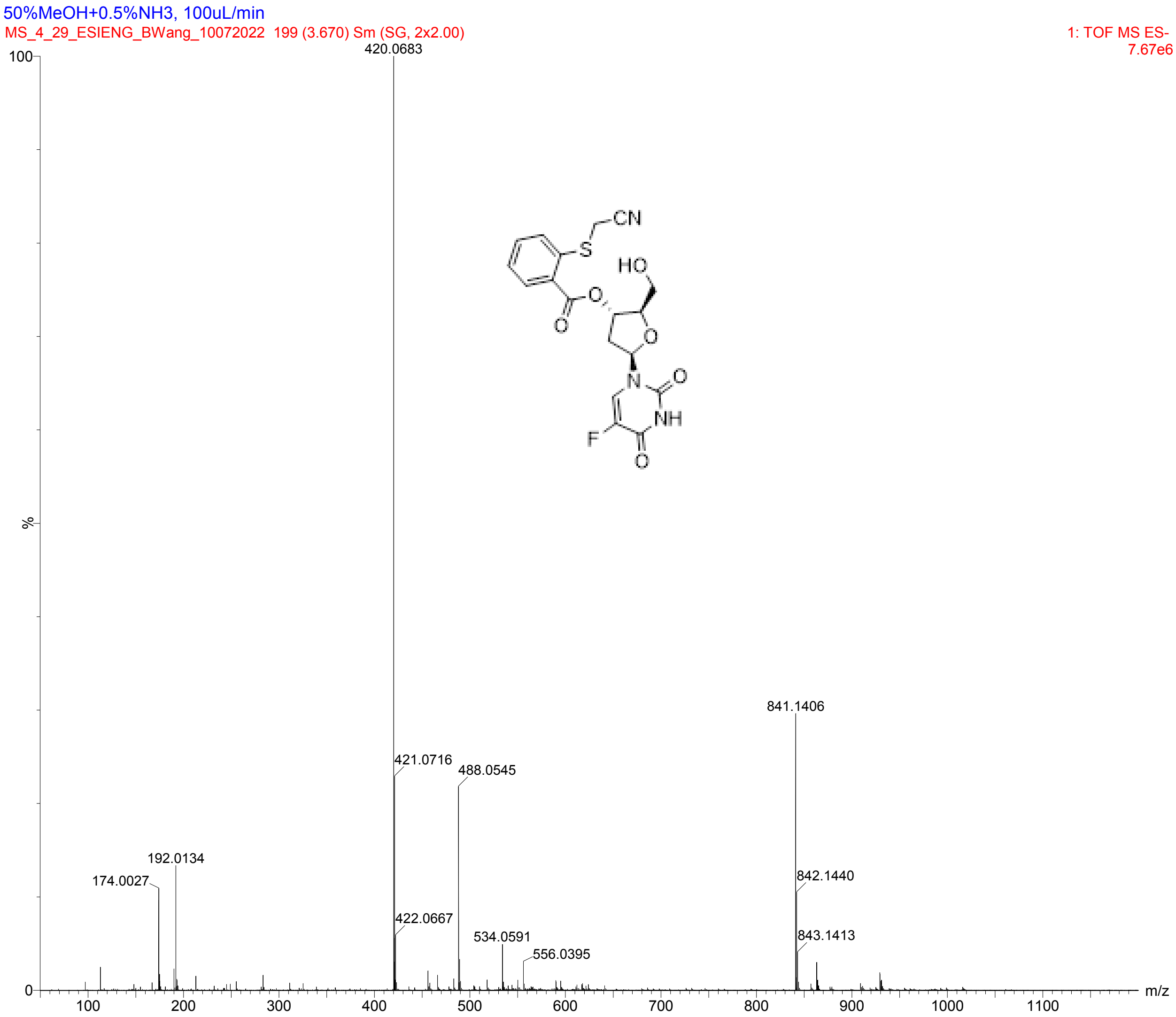

Compound **12d** (M+H= 455.0924)

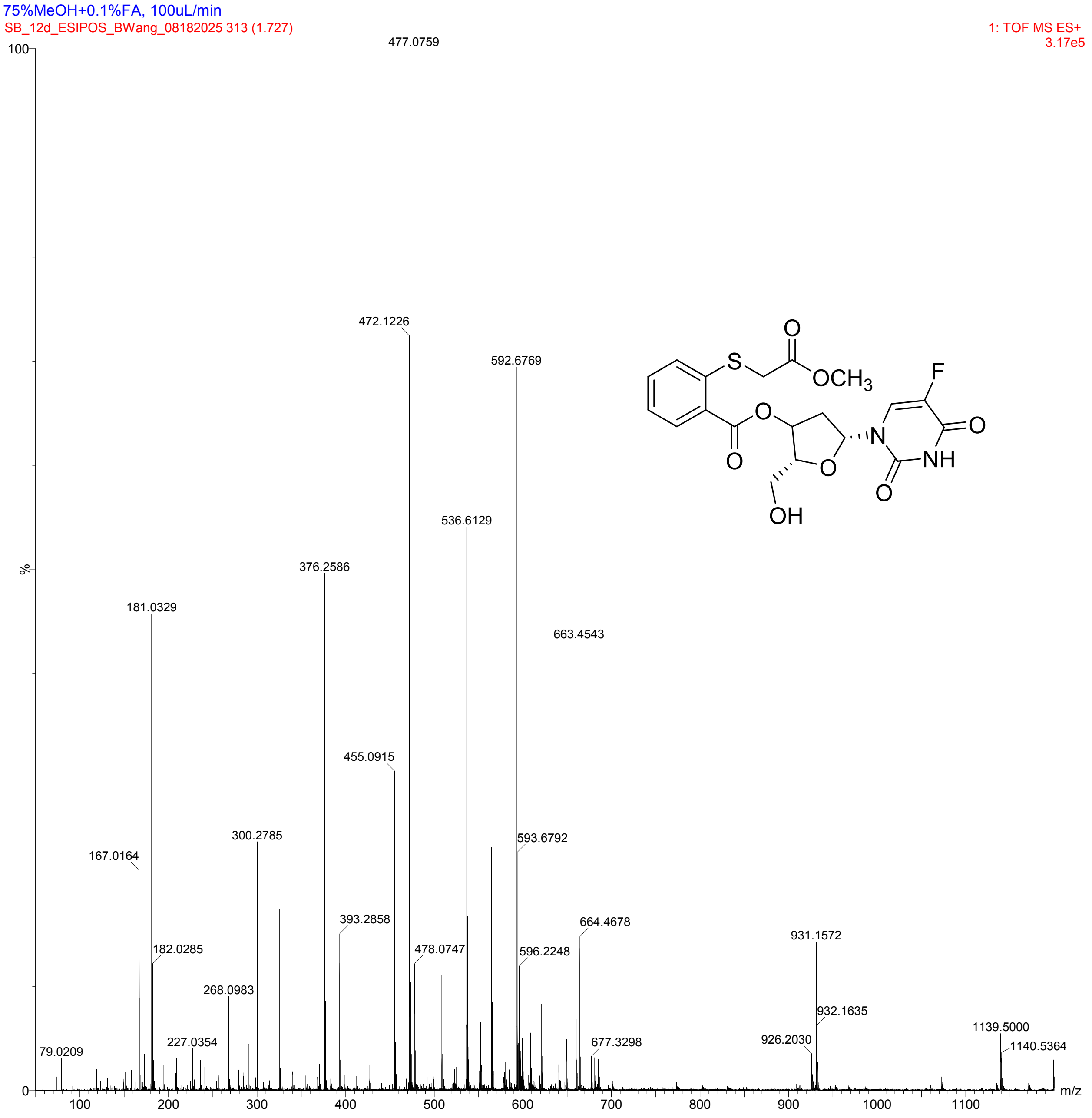

Compound **12e** (M+H= 440.0928)

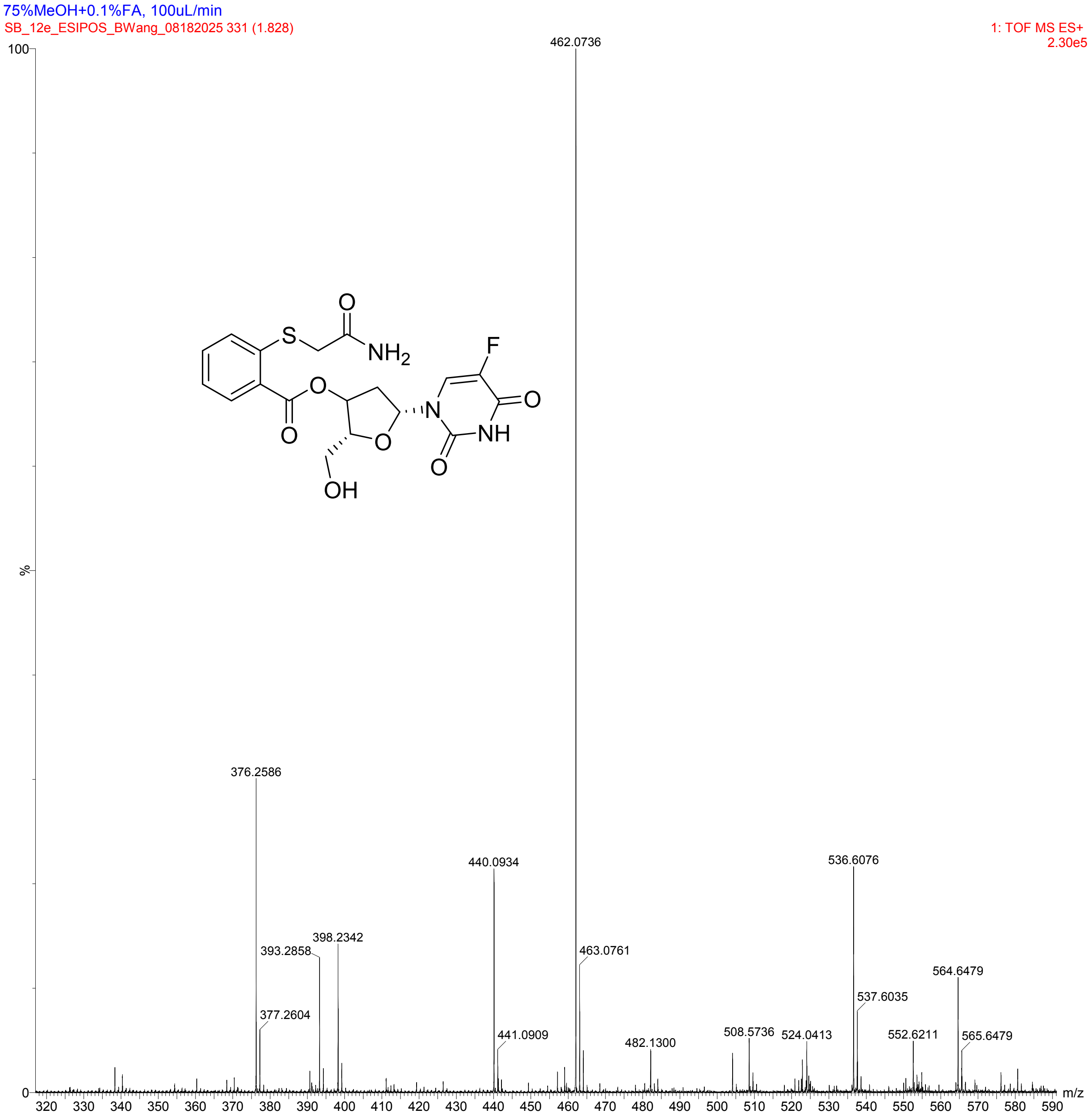

Compound **12f** (M+H= 482.1397)

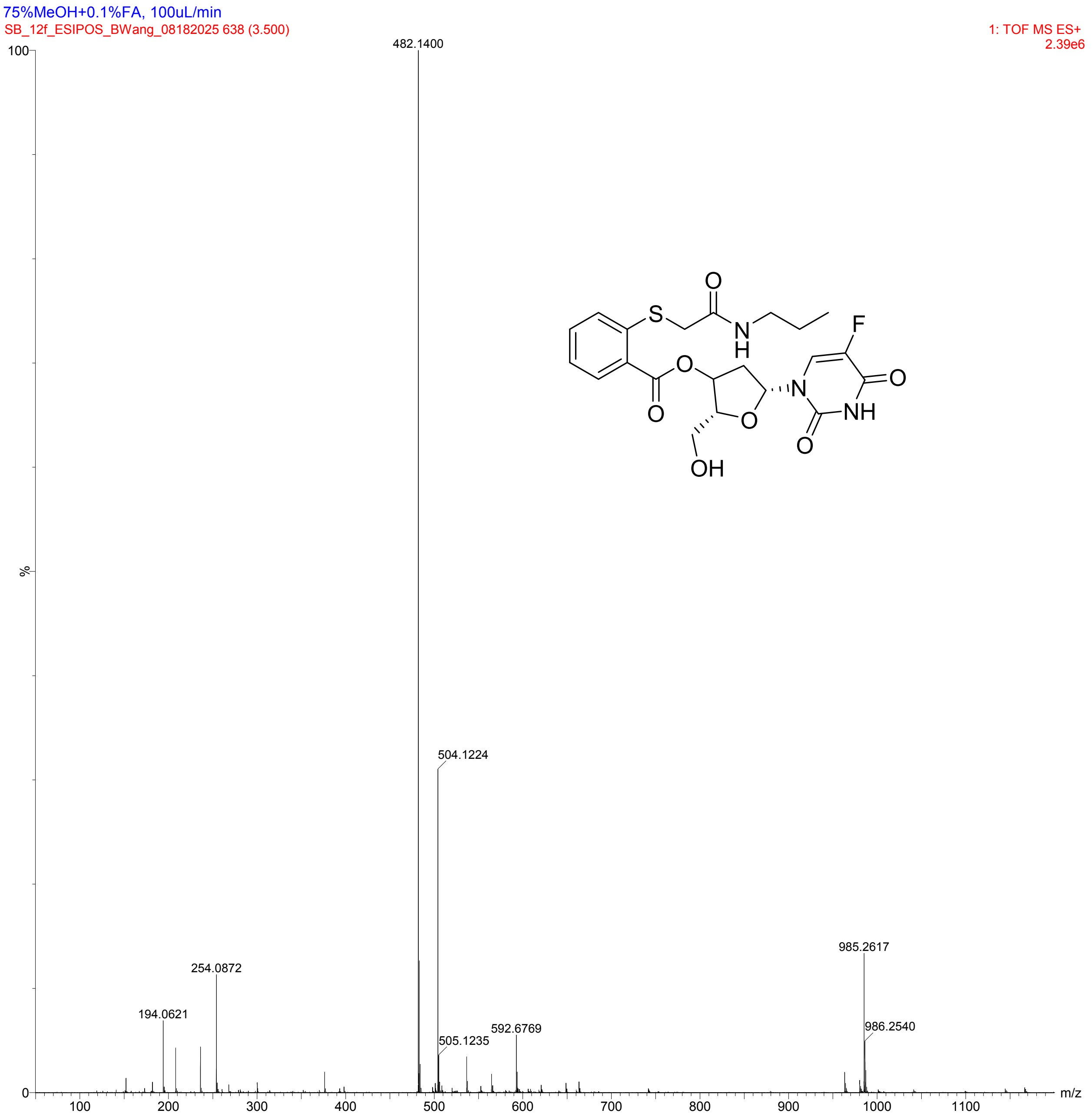

Compound **14e**. (M+H = 586.1648)

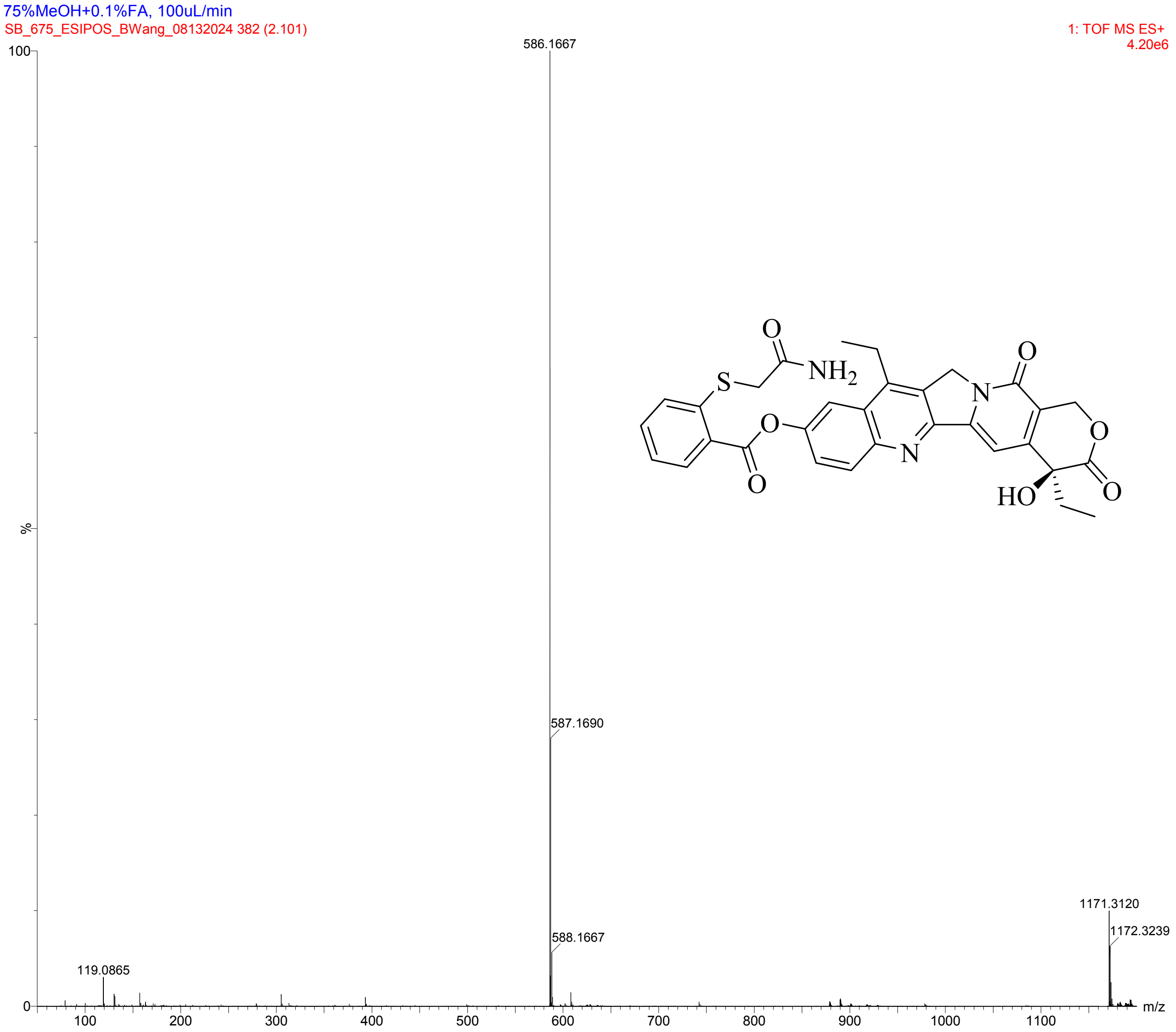

Supplement: Supplementary file 1 [file jm5c03627_si_001.pdf]
